# Increased levels of the megakaryocyte and platelet expressed cysteine proteases stefin A and cystatin A prevent thrombosis

**DOI:** 10.1038/s41598-019-45805-9

**Published:** 2019-07-03

**Authors:** Anna Mezzapesa, Delphine Bastelica, Lydie Crescence, Marjorie Poggi, Michel Grino, Franck Peiretti, Laurence Panicot-Dubois, Annabelle Dupont, René Valero, Marie Maraninchi, Jean-Claude Bordet, Marie-Christine Alessi, Christophe Dubois, Matthias Canault

**Affiliations:** 1 Aix Marseille Univ, INSERM, INRA, C2VN, Marseille, 13385 France; 2 CHU Lille, Université de Lille, Inserm U1011 – EGID, Institut Pasteur de Lille, Lille, France; 3 Laboratoire d’Hémostase, Centre de Biologie Est, Hospices Civils de Lyon, Bron, France; 4 Laboratoire de Recherche sur l’Hémophilie, UCBL1, Lyon, France

**Keywords:** Platelets, Obesity

## Abstract

Increased platelet activity occurs in type 2 diabetes mellitus (T2DM) and such platelet dysregulation likely originates from altered megakaryopoiesis. We initiated identification of dysregulated pathways in megakaryocytes in the setting of T2DM. We evaluated through transcriptomic analysis, differential gene expressions in megakaryocytes from leptin receptor-deficient mice (*db/db*), exhibiting features of human T2DM, and control mice (*db*/+). Functional gene analysis revealed an upregulation of transcripts related to calcium signaling, coagulation cascade and platelet receptors in diabetic mouse megakaryocytes. We also evidenced an upregulation (7- to 9.7-fold) of genes encoding stefin A (StfA), the human ortholog of Cystatin A (CSTA), inhibitor of cathepsin B, H and L. StfA/CSTA was present in megakaryocytes and platelets and its expression increased during obesity and diabetes in rats and humans. StfA/CSTA was primarily localized at platelet membranes and granules and was released upon agonist stimulation and clot formation through a metalloprotease-dependent mechanism. StfA/CSTA did not affect platelet aggregation, but reduced platelet accumulation on immobilized collagen from flowing whole blood (1200 s^−1^). *In-vivo*, upon laser-induced vascular injury, platelet recruitment and thrombus formation were markedly reduced in StfA1-overexpressing mice without affecting bleeding time. The presence of CA-074Me, a cathepsin B specific inhibitor significantly reduced thrombus formation *in-vitro* and *in-vivo* in human and mouse, respectively. Our study identifies StfA/CSTA as a key contributor of platelet-dependent thrombus formation in both rodents and humans.

## Introduction

Platelets originate from megakaryocytes (MKs) which are from haematopoietic stem cells differentiated through sequential stages^1^. As platelets develop, they receive their granule and organelle content as streams of individual particles transported from the MKs cell body^2^. During this differentiation, MKs migrate from the osteoblastic to the vascular niche in the bone marrow (BM). It is now recognized that the impaired BM microenvironment distinctly influences platelet phenotype and functions^3–6^. It is well known that the megakaryocyte-platelet system is turned-on during type 2 diabetes mellitus (T2DM). Indeed, Vera *et al*., demonstrated that megakaryopoiesis is altered in diabetic animals BM, giving rise to hyperactive platelets thus, contributing to an increased thrombotic risk^7^. Additionally, increased platelets activation has been reported in diabetic patients^8–11^, and is associated with increased oxidative stress, insulin resistance and inflammation^12–14^.

In an attempt to identify genes altered in MKs during T2DM, we compared, using a microarray approach, MK-derived mRNA isolated from *db/db* mice or from normal *db*/+ mice. In both groups, the patterns of gene expressions were elaborated and differentially expressed genes were identified. Following gene ontology mining and pathway analysis, we identified genes altered by T2DM, allowing to highlight several signaling pathways involved. Particularly, we identified a significant upregulation of the expression of the gene encoding for the Stefin A (Stfa), also referred to as Cystatin A (CSTA) in humans, a low molecular weight (13 kDa) Type 1 cysteine protease inhibitors^15,16^.

In this study we provide, for the first time, the evidences for the presence of Stfa/CSTA in MKs and platelets and its modulation by T2DM and the metabolic syndrome. We showed that CSTA is secreted by human platelets and we investigated its role in thrombus formation and stabilization *ex-vivo*, in a blood flow system and *in vivo* in mice. Although a precise function of platelets CSTA remains to be determined, we showed that increased circulating CSTA displays a protective effect characterized by reduced thrombus formation. These findings provide new insight into changes in platelet properties during obesity and T2DM.

## Results

### *db/db* MKs differentially express genes involved in platelet activation

**Figure 1:**
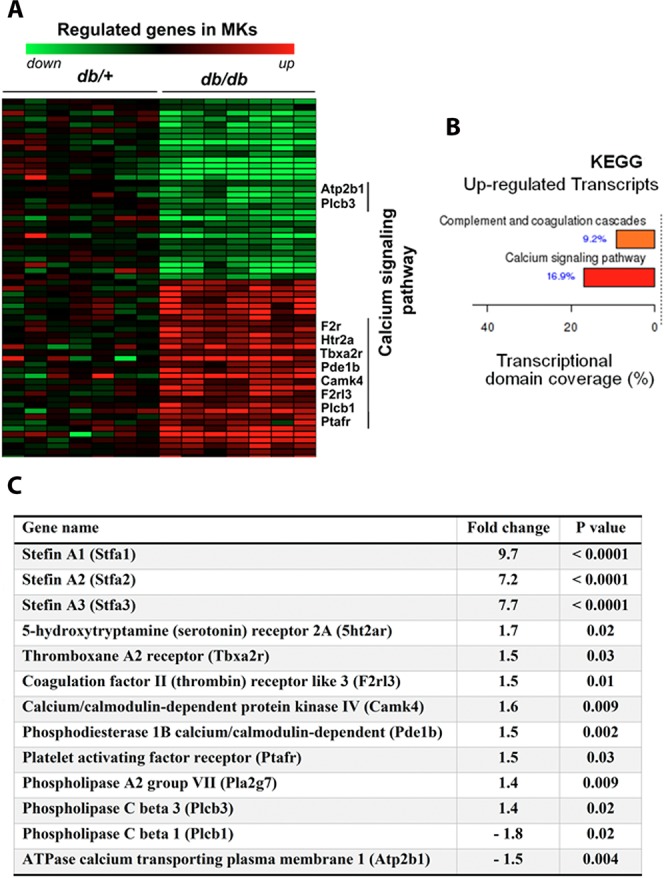
Transcriptomic signature of MKs cells in *db*/+ and *db/db* mice. (**A**) Heat map representation of the genes significantly down-regulated (green) or up-regulated (red) in mature MKs cells isolated from *db*/+ and *db/db* mice bone marrow. Data were clustered using the standard hierarchical method with linkage and the Pearson correlation. Horizontal axis displays animal samples, vertical axis displays each expressed genes by z-scores (scaled value of normalized intensity scores). (**B**) Kyoto Encyclopedia of Genes and Genomes (KEGG) enrichment analysis. KEGG enrichment was analyzed with FunNet software. KEGG Pathway database consists of graphical diagrams of biochemical pathways including most of the metabolic pathways and some of the regulatory pathways for the up-regulated genes. (**C**) List of genes involved in megakaryocytes/platelets biology.

We profiled MKs mRNA expression from diabetic *db/db* mice and *db*/+ control mice (metabolic characteristics are given in Supplementary Table S1). From 3210 probes, 1770 were filtered. Among the 893 genes differentially expressed (*p* ≤ 0.05; > 1.5-fold difference), 510 transcripts were upregulated and 383 were downregulated. Hierarchical clustering analysis showed two clusters that clearly differed between *db/db* and *db*/+ mice (Fig. 1A). Functional gene analysis, annotated with categories of the Kyoto Encyclopedia of Genes and Genomes (KEGG), revealed upregulation of transcripts related to pathways involved in calcium signaling (16.9%), coagulation cascade and platelet receptors (9.2%; Fig. 1B). The expression of key platelet activation receptor transcripts such as thrombin receptors, PAR4 (F2rl3), thromboxane A2 receptor (Tbxa2r), platelet-activating factor (Ptafr) and serotonin receptors (Htr2a) was significantly increased in *db/db* compared with *db*/+ mice (Fig. 1C). Supplementary Table S2 gathers all significantly regulated genes. These data evidence that during diabetes/obesity, modifications of MK gene expressions lead to hyperactivity/dysregulation of the produced platelets, which are associated with the disease.

### StfA is expressed in *db/db* mice MKs and in platelets from high sucrose (HS)-fed rats

**Figure 2:**
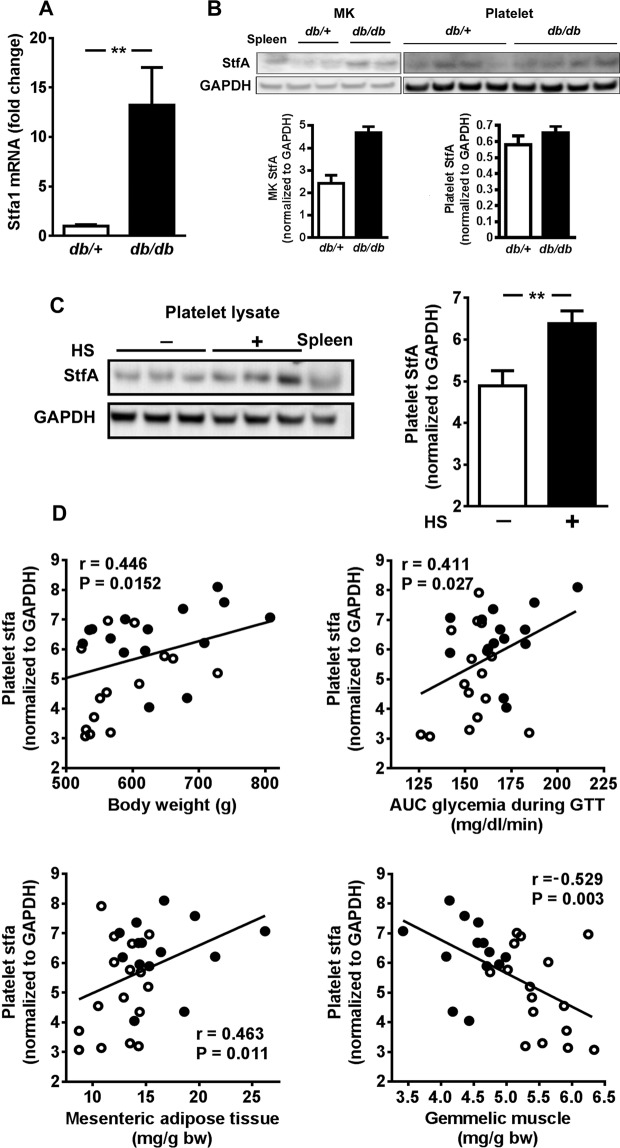
Regulation of stefin A1 (*stfa1*) mRNA and stefin A (StfA) expression in diabetic mice megacaryocytes (MK) or platelets in diabetic mice or high sucrose (HS)-fed rats. (**A**) Relative *Stfa1* mRNA levels in MK cells isolated from *db*/+ mice compared to *db/db* mice determined by real-time PCR; mRNA expression was normalized against 36B4 and *Stfa1* gene expression from *db*/+ was set as 1 (*n* = 10/group). (**B**) Representative Western blot of StfA expression of pooled (3 mice) MKs or platelets cellular lysate obtained from *db*/+ or *db/db* mice (*n* = 2 and 4/group, respectively). Results were normalized to GAPDH. The illustration resulted from a single gel. (**C**) Representative image (left panel) and quantification (histogram) of stfA immunoreactivity in platelets lysates obtained from control (**C**, *n* = 15) or HS-fed rats (*n* = 14) normalized against GAPDH. Rat spleen extract was loaded as StfA positive control. Results are mean ± SEM. **p < 0.01. (**D**) Simple linear regression analysis of rat platelet StfA protein and body weight, area under the curve (AUC) of blood glucose during glucose tolerance test (GTT), and mesenteric adipose tissue or gemmelic muscle weight obtained from control (○) or obese/glucose intolerant rats (●).

Among the significantly regulated transcripts, we highlighted a 7- to 9.7-fold increase (*p* < 0.01) in *stfa* 1, 2 and 3 mRNAs in *db/db* mice MKs compared with *db*/+ mice (Fig. 1C). Such upregulation was further confirmed by qRT-PCR, which demonstrated a 13-fold increase in StfA1 transcripts in *db/db* versus *db*/+ MKs (Fig. 2A). Immunoblot confirmed the presence of StfA in platelets and in MKs from *db*/+ *and db/db* mice (Fig. 2B). No difference in platelet counts from *db*/+ *and db/db* mice was noticed (data not shown). Similarly, StfA expression was found in rat platelets and significantly increased (*p* = 0.004) in obese and glucose intolerant rats fed a high sucrose (HS) diet compared with control animals (Fig. 2C). Then, we hypothesized that rat platelet CSTA/StfA content might be associated with some features of the metabolic syndrome. Indeed, it positively correlated with body weight (*r* = 0.446, *p* = 0.0152), fat mass (*r* = 0.463, *p* = 0.011) and glucose intolerance (*r* = 0.411, *p* = 0.027), and negatively with lean mass (*r* = 0.529, *p* = 0.003; Fig. 2D). Metabolic characteristics of HS-fed rats are shown in Supplementary Table S3.

### Evidence for the presence of CSTA in human megakaryocytes

**Figure 3:**
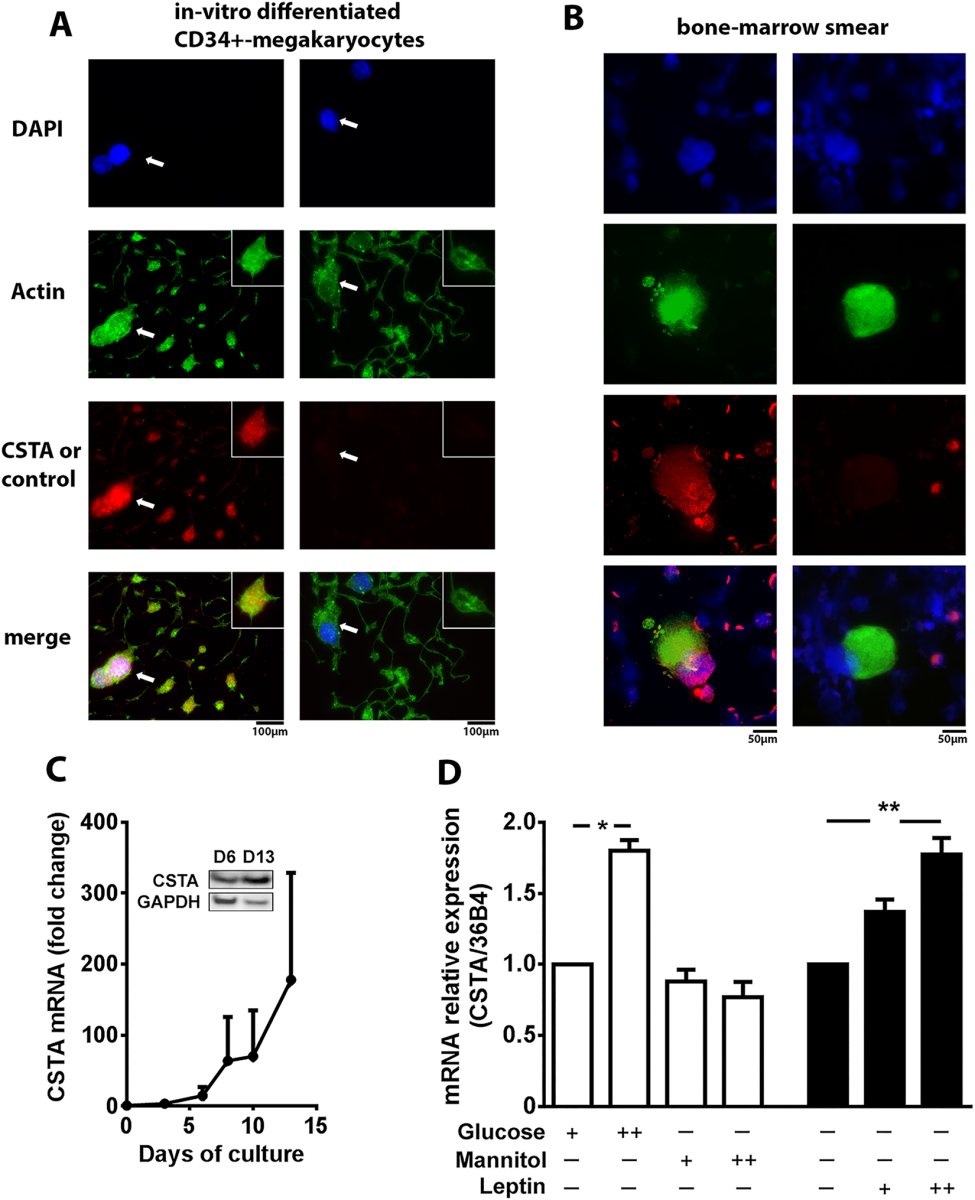
Regulation of cystatin A (CSTA) mRNA and protein expression during human megacaryocytes (MKs) differentiation and in human MKs precursors. (**A**) Representative immunofluorescence microscopy images of human CD34 + -differentiated megakaryocytes spread over fibrinogen-coated coverslips (day 13) stained with anti-CSTA antibody (left column) or without primary antibody (right column) (red), Alexa 488-coupled phalloidin for F-actin (green) and DAPI staining for nucleus (blue). The white arrow indicate the MK cellular body. An expanded view of the proplatelets is shown in the inset. (**B**) Representative immunofluorescence microscopy images of human bone marrow smear after staining for CSTA (red left column) or in absence of primary antibody (red, right column), F-actin (green) and DAPI (blue). (**C**) Time course analysis of CSTA mRNA levels during *in vitro* differentiation of peripheral blood CD34^+^ cells into MKs. mRNA expression was normalized against 36B4. Figure shows representative results from 2 independent experiments. Inset shows CSTA and GAPDH Western blot at D6 and D13. (**D**) Relative CSTA mRNA levels in human undifferentiated CMK cells incubated during 4 days with low (11 mM) or high (30 mM) concentrations of glucose or mannitol or with leptin (25 or 50 nM). mRNA expression was normalized against 36B4, and controls were set as 1. Figure shows representative results from at least three independent experiments. Results are mean ± SEM. *p < 0.05, **p < 0.005.

The human superfamily of cysteine protease inhibitors (cystatins) is divided into three families; among those the stefins (Family 1) comprises CSTA and CSTB. CSTA is the human ortholog of mouse StfA with 56–59% protein identity (Supplementary Fig. S4). They have conserved structural fold and amino acid segment involved in target enzymes binding, the cystatin motif (QXVXG). CSTA immunolocalization in proplatelet-forming CD34 + -derived MK spread over fibrinogen revealed that CSTA was concentrated in the perinuclear and cytoplasmic regions of the MK cellular body (Fig. 3A). Furthermore significant punctuate signal was detected in the released proplatelets as observed in the insert close up images. Confirmation of the presence of CSTA in megakaryocytes was obtained from human bone-marrow smear, where megakaryocytes were identified by their polyploid nucleus (DAPI staining) and the actin-positive signal (Alexa-448 labelled-phalloidin). In megakaryocytes, CSTA staining was observed all over the cytoplasm with higher levels around the nucleus (Fig. 3B). While the *CSTA* gene was not expressed in human CD34^+^ progenitor cells, its mRNA and protein increased along MK differentiation (Fig. 3C). Result analysis of the hematopoietic cell transcriptomic database, Haemopedia^17^ (version 4.9.5) revealed similar expression pattern for *stfa1 *mRNA during mouse MK differentiation. In addition, exposure of the human megakaryocytic CMK cells to high glucose or leptin concentrations, significantly (*p* < 0.05) increased relative *CSTA* mRNA expression. Substitution of glucose by equimolar concentrations of mannitol had no significant effect (Fig. 3D). Accordingly, exposure of differentiated (10 days) CD34 + -derived MK to a five time-increased glucose concentration (125 mM for high glucose vs 25 mM for control conditions) also lead to increased *CSTA* mRNA expression (1.36-fold increase vs normal glucose conditions) whereas the addition of an equimolar amount of mannitol did not (0.76-fold change vs normal glucose conditions). Altogether, these results are direct evidences for synthesis and metabolic regulation of CSTA in human MKs.

### CSTA is present in human platelets and is released during platelet activation

**Figure 4:**
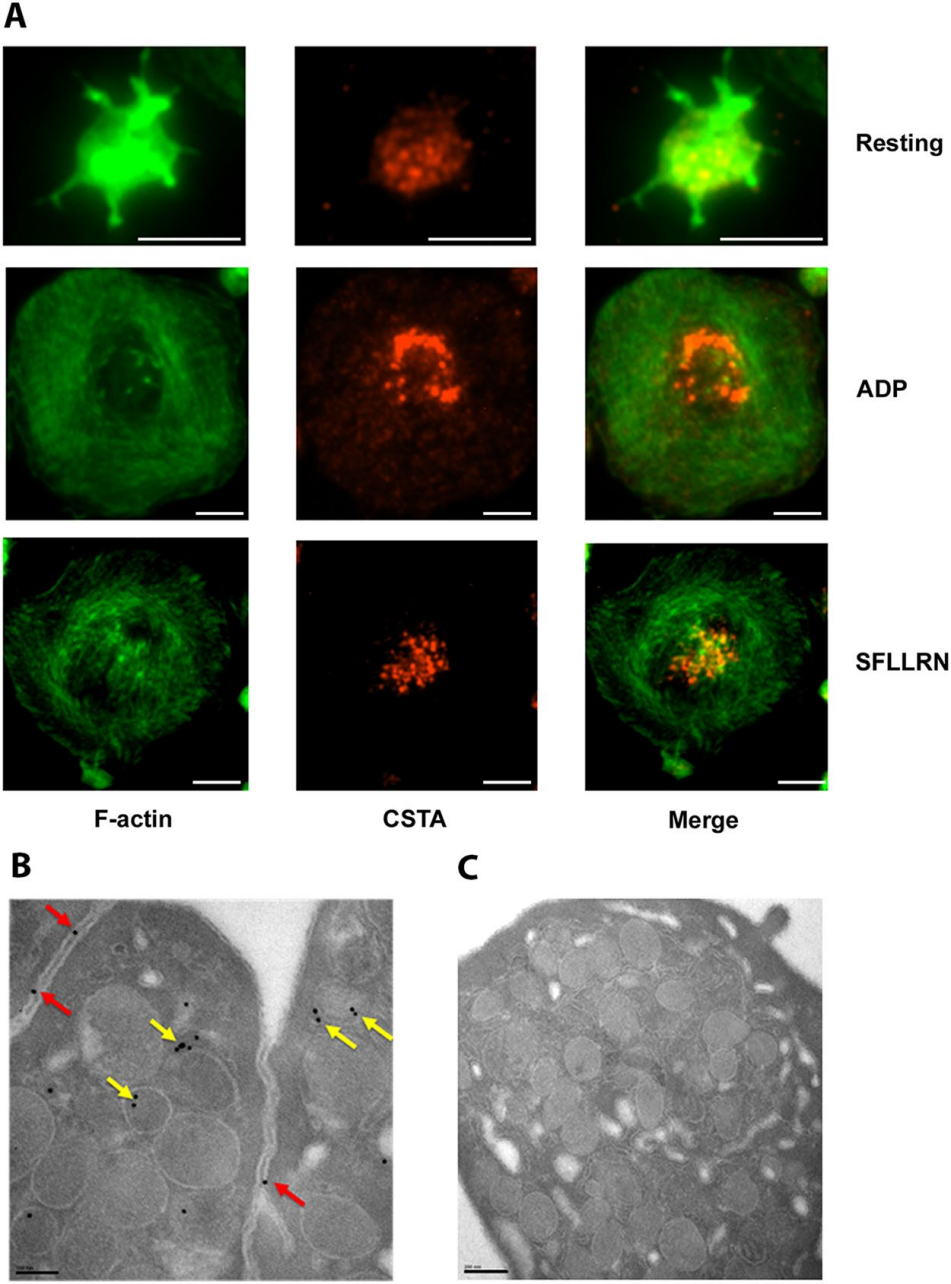
Platelets cystatin A (CSTA) localization and expression. (**A**) Immunofluorescence staining of washed platelets spread on fibrinogen-coated coverslips and incubated with or without 20 µM ADP or 10 µM SFLLRN. Platelets were double-stained for CSTA (red) and F-actin (green). Calibration bar = 4 µm. (**B**) Immunoelectron microscopy images showing resting platelets. Ultrathin platelet sections were probed for CSTA, and the bound antibody was labeled with immunogold (10 nm). CSTA appeared to be localized either in granule (yellow arrows) or at the membrane of granule; we can see also that it is present at the level of contact area within platelet membranes (red arrows). (**C**) As a control for CSTA staining, electron microscopic immunogold labeling was performed on resting platelets using isotype matched control antibody (IgGl). The results are representative of more than three independent experiments. Calibration bar = 200 nm.

**Figure 5:**
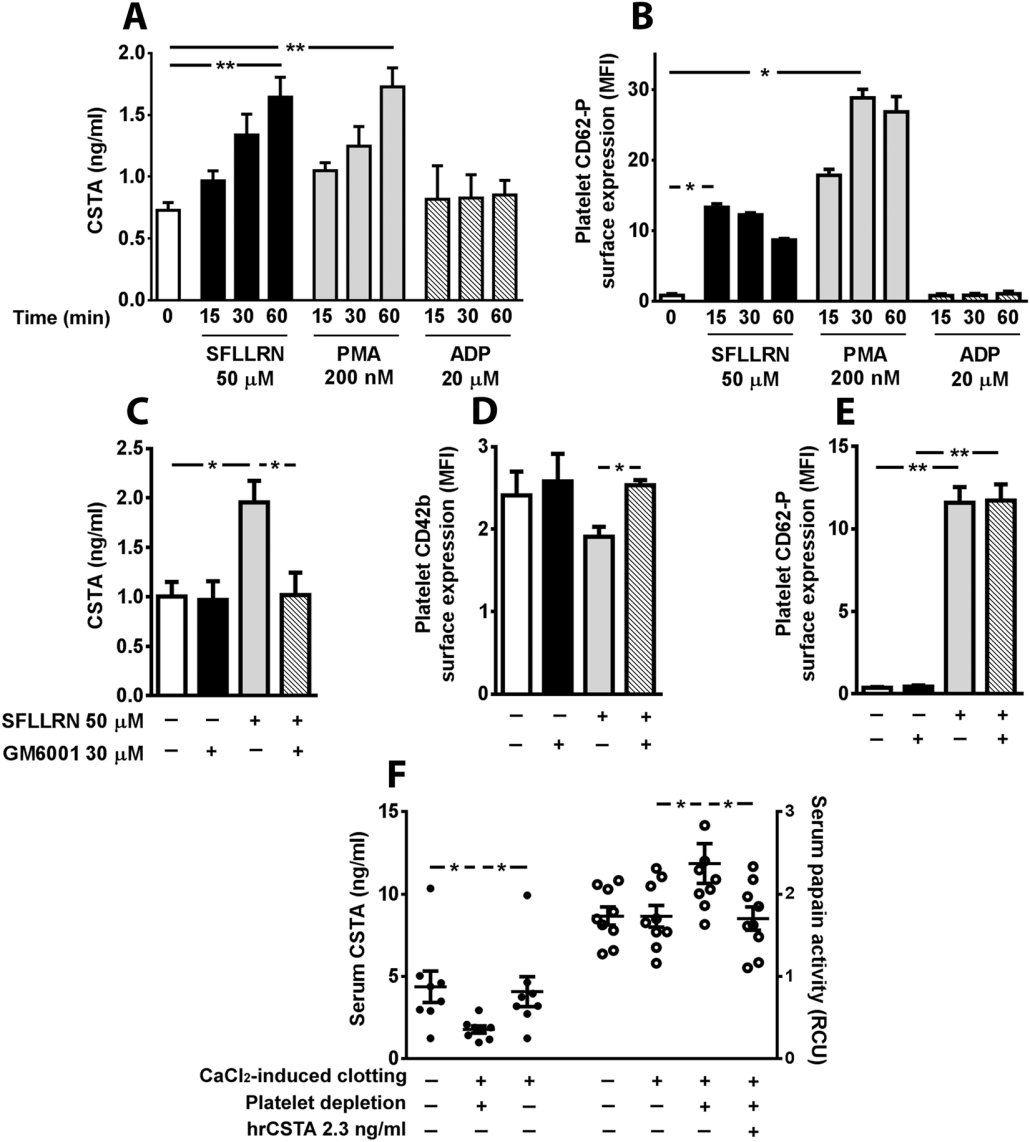
Regulation of human platelets cystatin A (CSTA) release. (**A**) Time course analysis of the effects of SFLLRN, PMA or ADP (*n* = 4 for each agonist) on CSTA release from platelet-rich plasma (PRP). (**B**) Time course analysis of CD62-P surface expression in PRP incubated with SFLLRN, PMA or ADP (n = 4 for each agonist, controls were set at 1). (**C–E**) Effects a matrix metalloproteases inhibitor (GM6001) on PRP incubated or not with SFLLRN. (**C**) Medium CSTA concentrations were determined by ELISA (*n* = 7, controls were set at 1). (**D**,**E**) Platelet CD42b (**D**) and CD62-P (**E**) surface expression was detected by flow cytometry (*n* = 6/group). F: Effects of calcium-induced blood clotting in plain or platelet-depleted serum on CSTA release and papain activity measured by ELISA or fluorescent assay, respectively. 2.3 ng/ml hrCSTA was added in platelet-depleted samples for papain activity determination. Data are expressed as mean ± SEM *p < 0.05, **p < 0.01.

Immunocytochemistry analysis revealed the presence of CSTA in human platelets spread over immobilized fibrinogen. In fully spread platelets, it was localized primarily in the platelet granulomere (Fig. 4A). Transmission electron microscopy showed the presence of immunogold-labeled CSTA at the α-granule membranes, the platelet surface and in the open canalicular system (OCS) (Fig. 4B,C). Spread platelets activated with 50 µM SFLLRN, the thrombin receptor (PAR-1)-activating peptide, showed weaker labeling than adenosine diphosphate (ADP)-stimulated (20 µM) platelets (Fig. 4A) supporting a possible SFLLRN-induced CSTA release. Indeed, SFLLRN (50 µM) and phorbol myristate acetate (PMA, 200 µM) but not ADP (20 µM), a weaker platelet agonist, induced a time-dependent release of CSTA from human platelets as evidenced by significant elevations of its concentration in the supernatants (Fig. 5A). Interestingly, CSTA accumulation kinetic upon platelet activation, differed from the kinetic of α-granule release evidenced by P-selectin (CD62P) surface expression. Indeed, maximal CD62P expression was already achieved after 15 and 30 min of SFLLRN and PMA stimulation respectively (Fig. 5B) whereas CSTA, detected in the supernatants of activated platelets as early as 15 min, showed increasing levels over the next 45 min (*p* < 0.01, Fig. 5A).

### CSTA release involves proteolysis

The CSTA release kinetic upon stimulation appeared completely different from that of classical soluble α-granule proteins^18^ and resembled to that described for CD40L^19^ involving a metalloprotease-dependent mechanism. Thus we suggest that metalloproteases participate to CSTA release. Pre-incubation with the broad-spectrum matrix metalloprotease inhibitor GM6001 fully blunted CSTA release from SFLLRN-stimulated platelets (*p* < 0.05; Fig. 5C) and, as already known^9^, efficiently (*p* < 0.05) suppressed GPIbα (CD42b) shedding (Fig. 5D) with no effect on P-selectin surface expression (Fig. 5E).

### CSTA is released during clot formation

To determine whether platelet CSTA is locally released during clot formation, we evaluated CSTA levels after calcium-induced clot formation, with or without platelet depletion. Platelet depletion did not affect leukocyte count (data not shown). CSTA levels in depleted samples were consistently reduced (1.78 ± 0.21 *vs*. 4.08 ± 0.90 ng/mL in controls, *n* = 8, *p* < 0.05, Fig. 5F), whereas papain activity, which reflects free cysteine protease activity, increased compared with the non-depleted paired samples (2.37 ± 0.24 *vs*. 1.73 ± 0.13 RFU in controls, *n* = 9, *p* < 0.05). Addition of the amount of CSTA lost following platelet depletion (2.3 ng/mL) fully recovered initial papain activity (1.70 ± 0.14 RFU, *p* < 0.05 *vs*. depleted samples). These results support significant CSTA shedding during platelet activation leading to local increase in biologically active CSTA during clot and serum formation.

### CSTA expression in obese/diabetic patients

**Figure 6:**
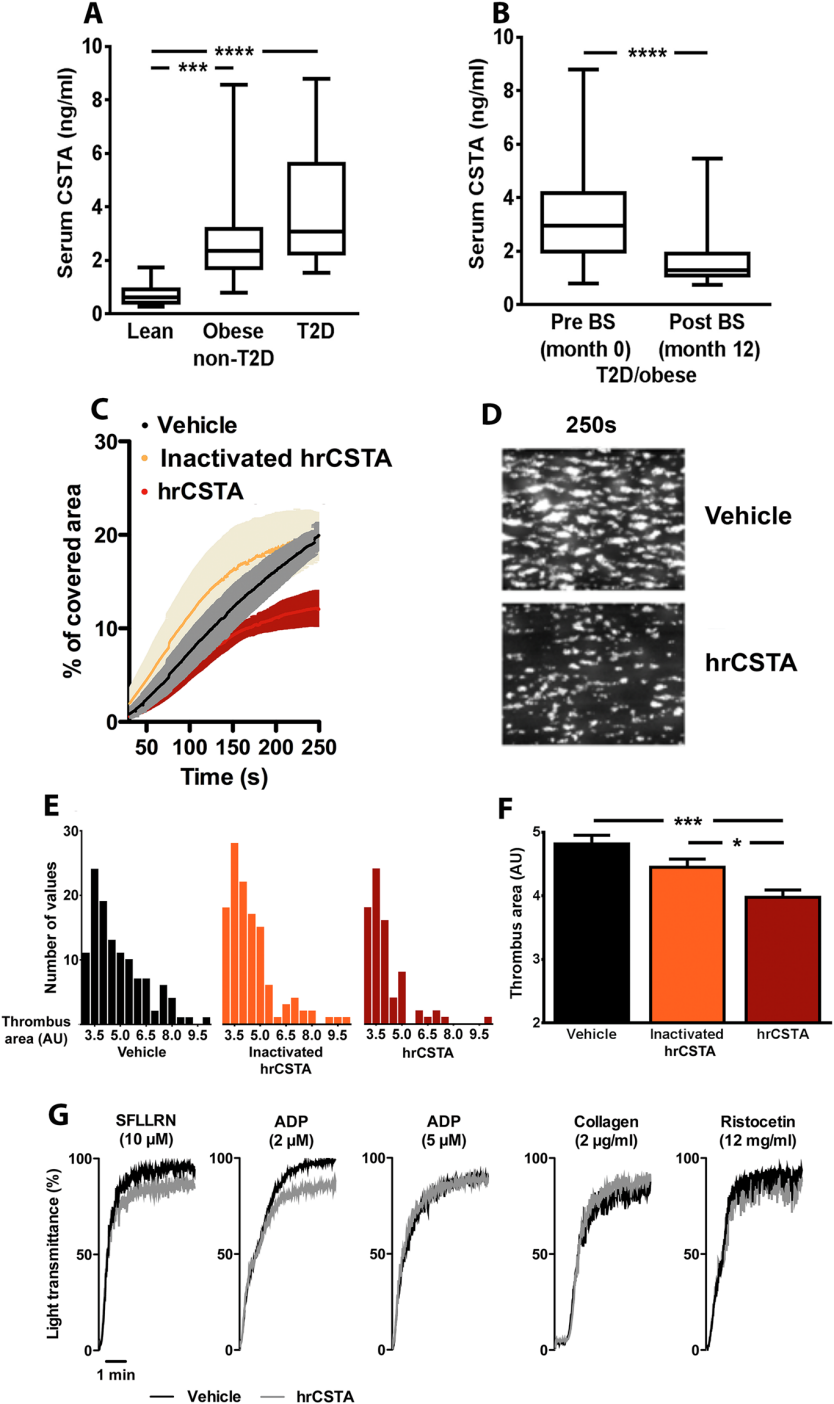
Effects of obesity/type 2 diabetes (T2D) on serum CSTA concentrations and effects of CSTA on *in-vitro* thrombus formation and platelet aggregation. (**A**) Box-and-whisker plot of serum CSTA in lean controls or obese without T2D or T2D patients (n = 10, 19 and 15, respectively). Results represent median and 2.5–97.5 percentile range. (**B**) Effect of bariatric surgery (BS) on serum CSTA in T2D/obese patients (n = 34). ***p < 0.001, ****p < 0.0001. C: Adhesion under flow (1200 s−1) on collagen of calcein-AM–labeled platelets from human whole blood pretreated with vehicle (black) or 100 ng/ml human recombinant heat-inactivated (hr)CSTA (orange) or hrCSTA (red). Percentage of covered area was assessed over 250 s. **(D**) Representative images of *in vitro* thrombus formation under arterial flow on collagen for both vehicle and hrCSTA-pretreated platelets after 250 sec. (**E**,**F**) Thrombus area distribution (**E**) and mean ± SEM (**F**) after 250 sec (n = 4 in each group). (**G**) Representative aggregation traces for control platelets in response to low-dose of SFLLRN, ADP, collagen and ristocetin with or without 100 ng/ml CSTA. *p < 0.05, ***p < 0.001.

We then determined CSTA serum levels in obese/diabetic patients. General characteristics, anthropometric and metabolic indices of the enrolled patients are shown in on line Supplementary Table S5. Significantly higher serum CSTA concentrations were found in patients with obesity or T2DM than in controls (*p* < 0.001 and *p* < 0.0001, respectively; Fig. 6A). No difference was noticed between the groups when CSTA was measured in PPP (data not shown). All obese/diabetics patients underwent bariatric surgery (BS; sleeve gastrectomy, *n* = 15, or Roux-en-Y gastric bypass, *n* = 19) and lost significant weight at 12 months. As expected, BS induced significant improvement of glucose tolerance, low-grade inflammation and insulin sensitivity indices (Supplementary Table S5). Twelve months after BS, serum CSTA levels were markedly decreased (1.64 ± 0.17 *vs*. 3.47 ± 0.36 ng/mL at baseline, *p* < 0.0001) (Fig. 6B), strongly suggesting that CSTA platelet levels increase during obesity and are further stimulated by T2DM.

### CSTA inhibits thrombus formation *in-vitro*

Because CSTA is released during clot formation, we hypothesized that it could influence thrombus formation. Whole human blood supplemented or not with hrCSTA and containing fluorescently-labeled human platelets was perfused over a collagen-coated surface at arterial shear rate (1200 s^−1^).  Ten ng/mL CSTA was ineffective, whereas 100 ng/mL significantly reduced the surface covered by platelets compared with control conditions (heat-inactivated hrCSTA or vehicle). Increasing divergence was noted after 150 s (Fig. 6C). After 250 s, thrombus area was not significantly modified by inactivated-hrCSTA treatment compared with vehicle-treated samples, but remained reduced (*p* < 0.001) in the presence of hrCSTA (Fig. 6D-F). Besides, *in-vitro* hrCSTA-treatment (100 ng/mL) had effect on neither platelet aggregation induced by all the tested agonists (Fig. 6G) nor clot formation and thrombin generation (measured by thromboelastography and calibrated automated thrombogram assay respectively, data not shown). These results reveal an antithrombotic effect of CSTA that is not directly related to platelet aggregation.

### Effect of StfA1 overexpression on thrombus formation and tail bleeding

We next investigated whether increasing circulating StfA1 levels affect *in-vivo* thrombus formation in a model of laser-induced injury of mouse cremaster arterioles. Elevated circulating StfA1 levels were obtained by systemic delivery of the StfA1 encoding vector, pLIVE * stfa1* using hydrodynamic injection. This resulted in a more than 300-fold increased liver *stfa1* mRNA concentrations (339 ± 101 *vs*. 1.0 ± 0.7 AU in pLIVE *StfA1 vs*. empty pLIVE, *p* = 0.0025) and a 3.3 fold increase in StfA circulating levels (pLIVE *StfA1 vs*. empty pLIVE: 1239 ± 194 *vs*. 379 ± 111 pg/mL, *p* < 0.01).

**Figure 7:**
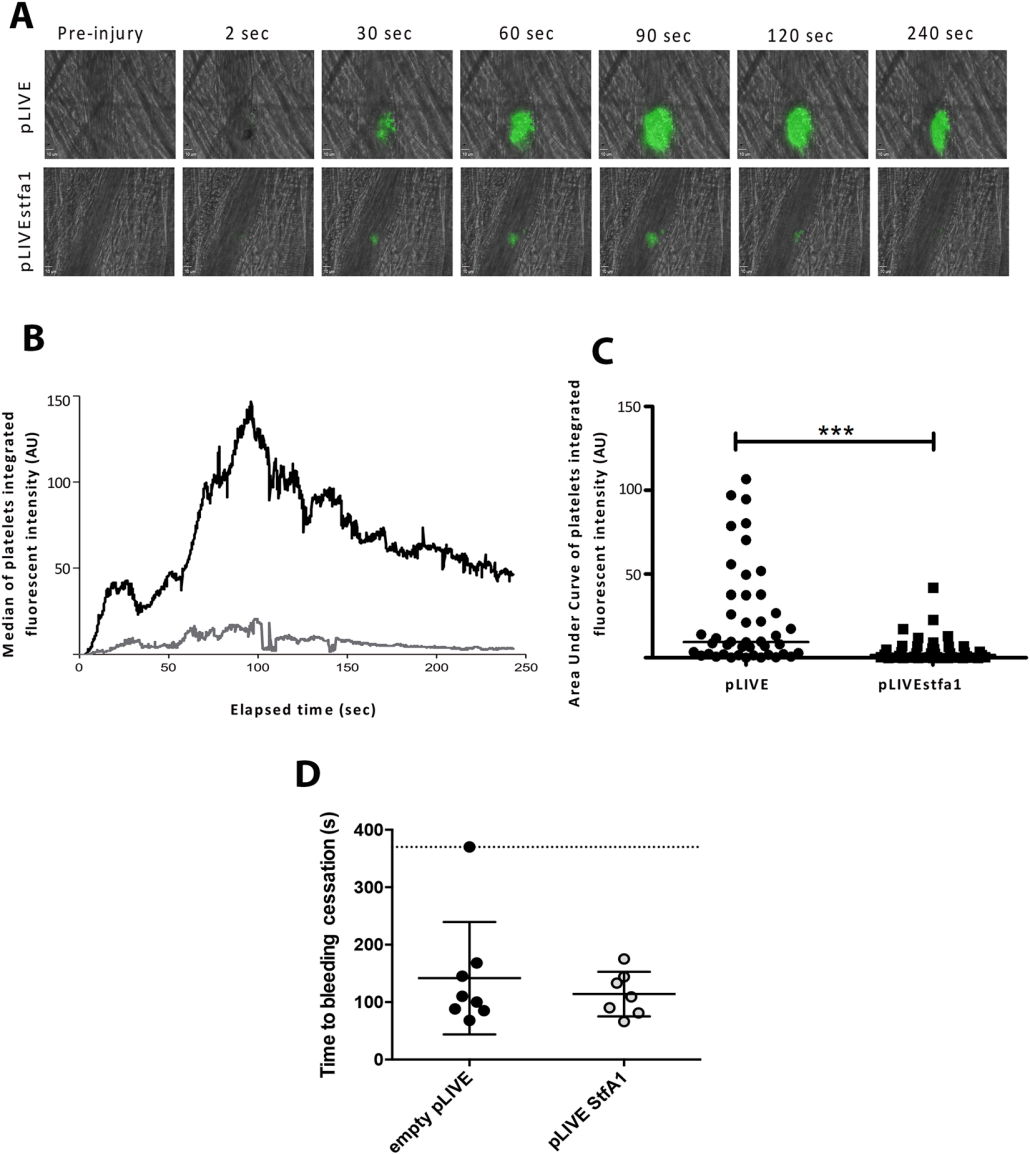
Platelet accumulation is reduced in mice overexpressing StfA1 following a laser-induced injury on the cremaster. (**A**) Representative composite images of fluorescence and brightfield data depicting thrombus formation (depicted in green) in wild- type previously injected with pLIVE (upper panel) and Wild type mice previously injected with pLIVE *stfa1* (lower panel). The antibody directed against platelets (Alexa-488 coupled anti CD41) was injected at 0.5 microgram per gram of mouse through the jugular vein before the injuries. (**B**) The median of platelet- integrated fluorescence (y-axis) based on 46 thrombi performed in 4 wild- type mice injected with pLIVE (black curve) and 43 thrombi in 4 wild-type mice injected with pLIVEstfa1 (grey curve) was represented over time. (**C**) The graph depict the distribution of the area under the curve for each thrombus in wild- type mice injected with empty pLIVE (circles) and in wild-type mice injected with pLIVE *stfa1* (squares). Horizontal lines represent the medians. ***p < 0.0005. (**D**) Measurement of the time to bleeding cessation after a 2 mm tail tip section on wild- type mice previously injected with the empty pLIVE vector (*n* = 8) or the pLIVE *stfa1* plasmid (*n* = 7). Each dot represent a single mouse. Data are also expressed as mean ± SEM.

As expected, in control mice (injected with empty pLIVE vector) platelets rapidly accumulated at the site of injury following a laser pulse and the thrombus increased in size to reach a maximum after 80–120 s, then decreased and stabilized 3–4 min later (Fig. 7A–C). When StfA1 was overexpressed, the thrombus size was markedly reduced in comparison with controls, confirming the previous results obtained *ex-vivo*.

No change was noticed in the bleeding time after a 2 mm tail tip section in pLIVE *stfa1*-injected mice compared with control mice that received an empty pLIVE vector (111 ± 17 *vs* 88 ± 8.6 s, *p* = 0.56; Fig. 7D); circulating von Willebrand factor levels also remained unchanged (132 ± 32 *vs*. 135 ± 50%, *p* = 0.95) revealing that StfA1 overexpression had no impact on hemostasis.

### Effect of cathepsin B inhibition on *in-vitro* and *in-vivo* thrombosis

**Figure 8:**
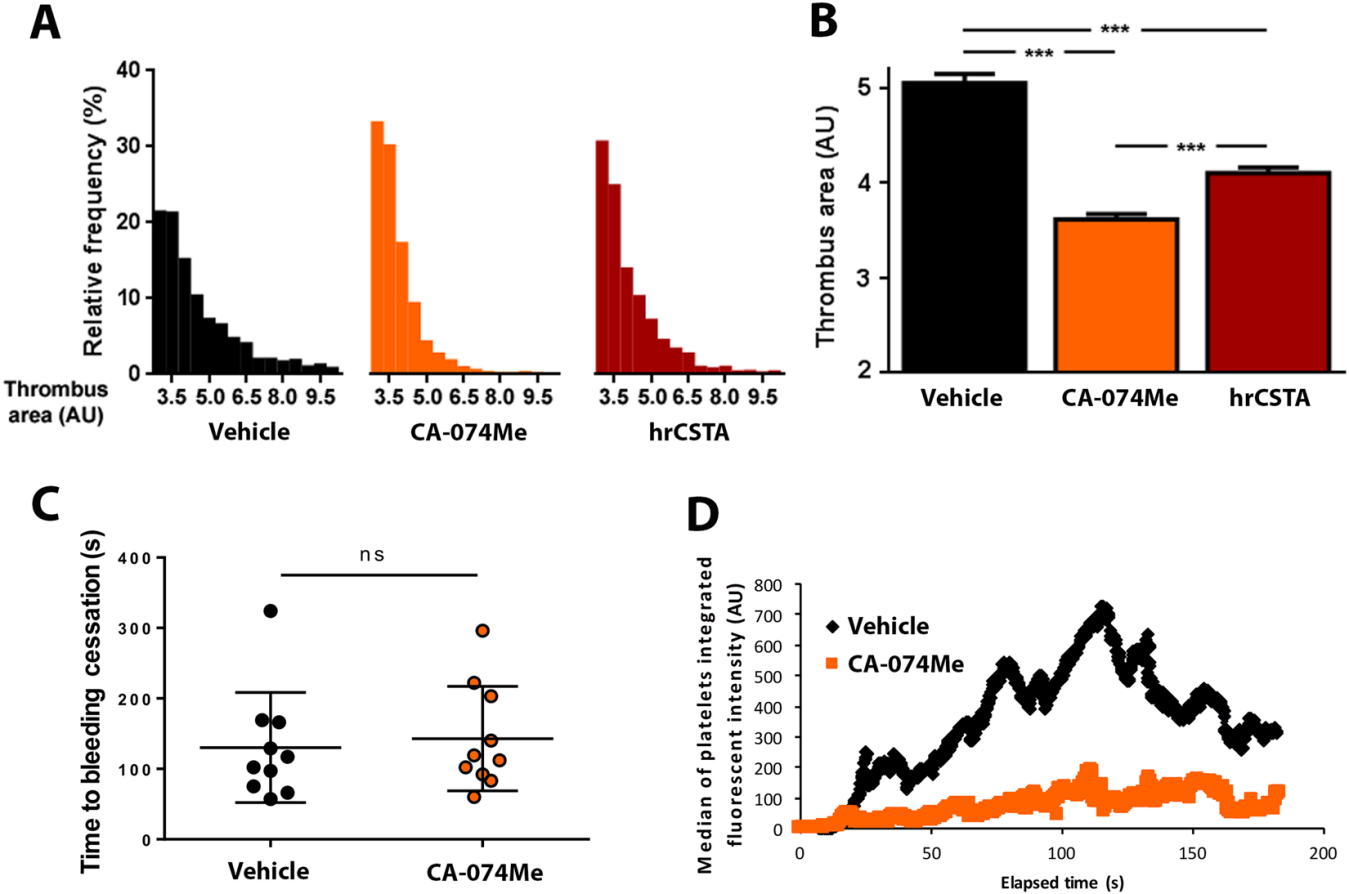
Cathepsin B inhibition by CA074-Me, reduces *in-vitro* and *in-vivo* thrombus formation. (**A**,**B**) *in-vitro* thrombus formation under arterial flow on collagen for vehicle, hrCSTA (100 ng/ml)- or CA-074Me (250 µM)-pretreated platelets. Thrombus area distribution (**A**) and mean ± SEM (**B**) after 250 sec (n = 4 in each group). (**C**) Measurement of the time to bleeding cessation after a 2 mm tail tip section on wild- type mice previously injected with vehicle or CA-074Me (12.5 mg/kg) (n = 10 in each group). Each dot represent a single mouse. Data are expressed as mean ± SEM. (**D**) The median of platelet- integrated fluorescence (y-axis) based on 42 thrombi performed in 4 wild-type mice injected with vehicle (black curve) and 41 thrombi in 4 wild-type mice injected with 250 µg CA-074Me (orange curve) was represented over time.

Finally, we asked whether the inhibitory effect on thrombosis observed in the presence of increased level of CSTA/StfA may rise from its inhibitory activity on cysteine proteases. We tested the effect on *in-vitro* and *in-vivo* thrombosis the effect of a cell-permeable irreversible inhibitor of intracellular cathepsin B^20^, CA-074Me. As compared to vehicle treated samples and similarly to hrCSTA, the presence of CA-074Me (250 µM) did not significantly impact *in-vitro* clot formation and thrombin generation (data not shown) but significantly reduced the size of the thrombi formed after 250 s of flowing human whole blood at arterial shear (1200 s^−1^) over immobilized collagen (Fig. 8A,B). Accordingly, in mice injection of 12.5 mg/kg of CA-074Me, a dose that significantly blocks cathepsin B activity in mouse liver (not shown), had no effect on bleeding time (Fig. 8C) but strongly decreased platelet accumulation at the site of laser-induced vascular injury on cremaster arterioles (Fig. 8D).

Overall, these results suggest that StfA/CSTA has no impact on hemostasis but significantly influences mechanisms controlling thrombosis.

## Discussion

Our study demonstrates that the cysteine protease inhibitor StfA was expressed in mouse MKs and platelets. MK *stfa1-3* mRNA and platelet StfA were substantially upregulated in diabetic mice or obese rats. We provide evidence that CSTA, the StfA human ortholog, was present in both human MKs and platelets, upregulated during obesity/diabetes and was released during platelet activation. Furthermore, increased StfA1 circulating levels in mice *in-vivo* and hrCSTA addition to human blood *in vitro* exerted a protective effect on thrombosis. Comparable inhibitory effects were further observed on *in-vitro* and *in-vivo* thrombosis in the presence of the cathepsin B inhibitor, CA-074Me. Thus, StfA/CSTA appears as regulator of platelet-dependent thrombus formation in both rodents and humans

Cystatins are reversible competitive inhibitors of C1 cysteine proteases. The major cysteine proteases interacting with cystatins include plant-derived papain and the mammalian cathepsins, B, H and L^21^. Cystatins are cytoplasmic inhibitors mainly regulating initiation or propagation of the lysosomal cell death pathway, such as apoptosis and necrosis^22,23^, which involves cathepsin B as a major downstream effector. To date, the role of this pathway in MK and platelet functions has not been clearly identified. Platelets contain the molecular machinery of lysosomal-dependent apoptosis among which cathepsin B^24^, and fine tuning of the apoptotic and antiapoptotic balance is described as a regulation mechanism in MK differentiation and platelet production^25^. Our results suggest the involvement of StfA/CSTA in the control of these processes during megakaryocytopoiesis and platelet life. This may be exacerbated in pathological conditions such as obesity and diabetes where platelet apoptosis has been reported^26^, but this needs further exploration.

StfA/CSTA was originally localized in the cytoplasm of squamous epithelia^27^ playing a role in desmosome-mediated cell-cell adhesion^28^. As an exception, StfA/CSTA was described in granulocytes and follicular dendritic cells^29,30^. Interestingly, Davies and Barrett^31^ found that some cells surrounding blood vessels demonstrated positive StfA/CSTA staining with granular appearance. In platelets, we show StfA/CSTA storage within α-granules and its expression at the cell membrane. Burkhart *et al*.^32^ identified CSTA at the platelet membrane level using a quantitative proteomic approach with membrane-dedicated analysis. Association of the StfA/CSTA with intracellular vesicular membranes/vesicles might explain how it becomes associated with the cell surface^33^.

The fact that StfA/CSTA was released in response to platelet activation reinforces its potential extracellular function at the site of vascular lesion. Vascular cells can mobilize cathepsin B in the extracellular space, promoting extracellular matrix degradation^34^. Our demonstration that StfA/CSTA release was reduced by a broad spectrum metalloprotease inhibitor (GM6001) suggests that metalloproteases secreted during inflammatory diseases and platelet activation could locally increase CSTA bioavailability, which may serve as a regulatory mechanism for cell adhesion and matrix degradation. The mechanisms by which StfA/CSTA release is modulated by metalloproteases will require further work.

Interestingly, the existence of StfA/CSTA was first demonstrated by reporting a factor able to inhibit the clotting activity of thiol-dependent proteases during the induction of an allergic skin reaction^35^. Many members of the StfA/CSTA superfamily were later identified^36^ and primarily explored with respect to their capacity to inhibit intracellular cysteine proteases in cancer cells. However, their potential role in the control of hemostasis and/or thrombosis was not pursued any further. Few observations support the hypothesis that lysosomal proteinases released in the extracellular milieu could play a role in thrombosis. Cathepsin B has been involved in a thrombotic tendency, inhibiting the fibrinolytic system in cultured endothelial cells^37^. Lysosomal proteinases induce thrombin formation leading to a disseminated intravascular coagulation phenotype^38^. In addition, recent results indicate that two major intracellular degradation systems, the ubiquitin-proteasome system and autophagy, must occur *in-situ*, in growing thrombi, for effective hemostasis and thrombosis^39,40^. Because StfA/CSTA is expressed in platelets and released during clot formation, we speculated that it is able to locally influence thrombosis: hrCSTA reduced the size of platelet thrombi in a human whole-blood perfusion assay under arterial shear and StfA1 overexpression prevented thrombosis following laser-induced injury in the mouse microcirculation of the cremaster muscle. Similarly, the Cathepsin B inhibitor CA-074Me reduced *in-vitro* and *in vivo* thrombus formation, suggesting that StfA/CSTA may exert control on the thrombotic process through its cysteine protease inhibitory activity. The laser-induced injury we used leads to thrombus formation after local endothelium activation, without exposure of the sub-endothelial matrix. In this model, binding of neutrophils to activated endothelial cells is the first step in thrombus formation and fibrin generation followed by tissue factor (TF)-mediated thrombin generation, which plays a central role in platelet activation^41,42^. However, neither hrCSTA nor CA-074Me influenced *in-vitro* clot formation and thrombin generation in clotting plasma. Remarkably, hrCSTA and cathepsin B inhibitor did not change bleeding time or initial thrombus formation, indicating an anti-thrombotic rather than an anti-hemostatic role. There remain to be determined the molecular and cellular targets that have to be protected by CSTA to prevent thrombosis and whether this role is retained or lost in the course of obesity and diabetes.

## Methods

### Animals

Animal experiments were performed following the European directive 2010/63/EU and the ‘Principles of laboratory animal care’ (NIH, 2015) and approved by the French Education and Research Ministry (#01585.02, #2017031413241381_v3 and 2017031413413414597_v1). Ten-week-old male BKS.Cg-m +/+ Lepr db/J genetically diabetic mice (*db/db*) and their non-diabetic lean BKS.Cg-m +/−Lepr db/J (*db*/+) littermates were purchased from Charles River Laboratories (Les Oncins, France). Male Wistar rats were in house bred using genitors from Janvier Labs (Le Genest-Saint-Isle, France). After weaning, rats were given tap (control, C) or high-sucrose (HS, 30%) water. Metabolic assessment, BM and blood collection, euthanasia and organ dissection were performed as previously described^43,44^. Blood was centrifuged at 180 *g* for 7 min at 22 °C; the supernatant was centrifuged at 150 *g* for 5 min, the platelet-rich plasma (PRP) was collected, and platelet count adjusted to 3–4 × 10^8^/mL.

### Subjects

Participants were recruited among volunteers referred to the blood sampling center (*n* = 10) and patients referred to the Department of Nutrition, Metabolic Diseases and Endocrinology at La Timone University Hospital, Marseille, France, with an indication for bariatric surgery (BS). These participants were examined and followed by a multidisciplinary and integrated medical team consisting of an endocrinologist, a bariatric surgeon, a psychiatrist, and a dietician for at least 6 months before surgery. All the subjects met the indication criteria for bariatric surgery. The choice of the surgical procedure was made by the patient and the multidisciplinary team, after a full explanation of the risks and possible benefits of each procedure. Bariatric surgery was performed by a single surgeon in the General and Endocrine Surgery Department. The clinical evaluation and laboratory tests were conducted at our Nutrition Department.Thirty-four morbidly obese patients (12 men, 22 women, 15 with T2DM) underwent BS: sleeve gastrectomy (*n* = 15) or Roux-en-Y gastric bypass (*n* = 19)^45^. All participants were free from medication or disease known to interfere with platelet function. This study was conducted in accordance with the Declaration of Helsinki, approved by the Research Ethics Board of Aix-Marseille University and all subjects gave written informed consent.

### Gene expression microarrays on megakaryocytes from mouse bone marrow

MKs from *db/db* and *db*/+ BM were enriched using Ficoll gradient (Eurobio, Courtaboeuf, France) prior selection by fluorescence-activated cell sorting (Aria III SORP, Becton Dickinson) based on CD41^+^ cells (anti-mouse-CD41-Phycoerythrin, ThermoFischer Scientific) and ≥ 8 N ploidy (Staining with 10 μg/mL Hoechst 33342, Sigma-Aldrich, Saint-Quentin-Falllavier, France). No significant difference was noticed in the number of mature MKs harvested during *db/db* and *db*/+ BM cell sorting (*db/db*: 4.64 ± 1.93% vs *db*/+: 5.31 ± 1.83%, *n* = 10 per group, *p* = 0.3).

Total RNA from 10^5^ cells was extracted using an RNeasy mini kit (Macherey-Nagel, Hoerdt, France) and RNA Clean & Concentrator (Zymo Research, Irvine, CA). RNA integrity was tested on an Agilent 2100 BioAnalyzer (Agilent Technologies, Santa Clara, CA). Reverse transcription and quantitative real time PCR (qRT-PCR) were performed as described elsewhere^31^ with StfA1 specific primers.

Total RNA was converted into complementary DNA and labeled using the Quick Amp Labeling (one color, Cy5) kit (Agilent Technologies). Hybridization was performed using SurePrint G3 Mouse Gene Expression 8 × 60 K microarrays (Agilent Technologies, G4852A). Arrays were scanned on an Agilent DNA microarray scanner and processed using the Feature Extraction Software 10.7.3.1 (Agilent Technologies). Data were processed with GeneSpring 11.5.1 (Agilent Technologies). The expression value of each sample was normalized, and baseline values of mean fluorescence intensities from the control group were subtracted from each value of the experimental group to enter them into the MeV microarray program experiment viewer (TM4, USA) and to analyze the transcriptional profiles of the group. A cluster analysis was then generated to identify the transcriptional profiles of each group. We performed a functional analysis of these gene lists using the FunNet tool^46^ (http://www.funnet.integromics.fr/).

### StfA and CSTA assays

StfA and CSTA levels were determined by ELISA (Cloud-Clone Corp., Houston, TX). The inhibitory function of hrCSTA on protease activity of papain was determined by measuring the enzymatic release of amino-methyl-coumarin (AMC) from the peptide substrate, Z-Phe-Arg-AMC according to RayBiotech’s protocol. Antipapain activity was determined by measuring the enzymatic release of amino-methyl-coumarin (AMC) from the peptide substrate, Z-Phe-Arg-AMC; hydrolysis rates were monitored for 5 min at 360 nm excitation and 450 nm emission, using a Victor^TM^ X4 reader (PerkinElmer).

### Cultures of human MKs

*In-vitro* differentiation of human CD34^+^ cells was performed as previously reported^47^. Human acute megakaryocytic leukemia cells (CMK, 5 × 10^5^/mL) were cultured for 4 days in RPMI-1640 medium with 10% fetal bovine serum (FBS), 100 IU/mL penicillin/streptomycin at 37 °C, in 5% CO_2_, and supplemented with d-glucose, d-mannitol or leptin (from Sigma-Aldrich). RNA was extracted from CD34 + and CMK cells using a NucleoSpin RNAs kit (Macherey-Nagel). Gene expression changes were measured by qRT-PCR as previously described^43^. Primers sequences are available on request.

### Platelet studies

Human platelet-poor plasma (PPP), PRP and washed platelets were prepared as previously described^43,48^. Platelet counts in PRP and washed platelets were adjusted to 3–4 × 10^8^ cells/mL using RPMI 1640. Platelet aggregation was performed as previously described^48^. CSTA release was studied in PRP and washed platelets stimulated (60 min, 37 °C) with SFLLRN (50 µM), ADP (20 µM) or PMA (200 nM). In some experiments, PRP was pre-incubated (20 minutes, 37 °C) with or without a metalloproteases broad-spectrum inhibitor (GM6001, 30 µM) prior activation. After activation, PRP samples were centrifuged (1000 × *g*, 3.5 min, 22 °C) and supernatants stored at −80 °C until CSTA determination. Granule secretion was evaluated by flow cytometry using PE-CD62P (clone AK-4; eBioscience) and FITC-CD42b (clone HIP1; eBioscience) antibodies, and analysis performed on a BD-Accuri^TM^ C6 flow cytometer (BD Biosciences). In other experiments, platelet-depleted blood was supplemented with an equal volume of PRP or PPP and CaCl_2_ (10 mM) prior incubation (120 min) at RT. After coagulation, samples were centrifuged (4500 × *g*, 10 min, 4 °C) and the sera were stored at −80 °C until CSTA content assay. The experiment was repeated with addition of 2.3 ng/mL hrCSTA in the platelet-depleted samples for papain activity determination.

### Immunoblot analysis

CSTA/StfA was detected on platelets from mice and rats and from human CD34 + derived MKs by immunoblots as previously described^47,48^ with anti-human/mouse/rat CSTA antibodies (GeneTex, Irvine, CA). Image acquisition was performed with a chemiluminescent CCD imager ImageQuant LAS 4000 (GE Healthcare, Alnay Sous Bois, France). Densitometry analysis was performed with the ImageQuant TL software. Illustrations were made using Adobe PhotoShop CC Software (Adobe Systems Incorporated).

### Epifluorescence microscopy

Platelets and CD34 + -derived differentiated MK were allowed to adhere over coated fibrinogen (100 µg/ml) for 1 hour and 3 days respectively. Platelets, MK and bone marrow smears were fixed in 1% paraformaldehyde for 10 min at room temperature. After washing, cells were permeabilized with 0.3% Triton X100 in PBS for 5 min, blocked using 3% BSA PBS for 1 hour and incubated overnight with rabbit anti-CSTA antibody (Genetex; 63944). Next, cells were incubated with anti-rabbit Alexa-546-labeled secondary antibody (Life technologies; A11010), Alexa-488-labeled Phalloidin (Life technologies; A12379). Finally, after washing steps, the slides were mounted with DAPI-containing Fluoromount and examined using an AXIO Imager M1 microscope (Carl Zeiss, Germany).

### Immunogold electron microscopy

Human platelets were fixed with 8% paraformaldehyde supplemented with 0.2% glutaraldehyde (Electron Microscopy Sciences EMS, Hatfield, PA) and 0.2% sodium metaperiodate added extemporaneously in a 0.2 M sodium cacodylate buffer pH 7.4. After centrifugation 1500 g 10 min RT, platelets were washed with 0.2 M cacodylate and 0.4 M saccharose, dehydrated in graded ethanol and embedded in pure LR-White. Sections (70 nm thick) were cut using a Reichert Ultracut ultra microtome (Leica Microsystems, Wetzlar, Germany), mounted on 200 mesh nickel grids (EMS) coated with 1:1000 polylysine. Non specific sites were blocked (1% BSA and 1% normal goat serum in 50 mM Tris-HCl pH 7.4, 20 min at RT). Antibodies incubations were carried out overnight at RT in with rabbit IgG anti-CSTA antibody (1:50) or a non-immune rabbit IgG dilution (negative control). Sections were washed (50 mM Tris-HCl; x3) and incubated at RT (45 min) with 10 nM gold particles-conjugated goat anti-rabbit Ig (Aurion, Wageningen, The Netherlands). Samples were washed (x3 in 50 mM Tris-HCl pH 7.4 and distilled water) and fixed with 4% glutaraldehyde (3 min). Sections were stained with 5% uranyl acetate and observed with a transmission electron microscope 1400 JEM (JEOL, Tokyo, Japan), equipped with an Orius 600 Gatan camera and the Digital Micrograph software (Gatan, Pleasanton, CA) (Lyon Bio Image, Centre d’Imagerie Quantitative de Lyon Est, France).

### *In-vitro* thrombus formation

Thrombus formation was studied as previously described^48^. Briefly, platelets were labeled with 1 µg/mL calcein-AM for 30 min. Reconstituted whole blood was pre-incubated for 15 min at 37 °C with 10–100 ng/mL hrCSTA, heat-inactivated hrCSTA or for 1 hour at 37 °C with 10–250 µM CA-074Me or corresponding vehicles, and injected into channels at an arteriolar shear rate of 1200 s^−1^. Images recorded over 300 s were analyzed using ImageJ software. The surface covered (%) by fluorescent platelets and the areas of thrombi were determined.

### Hydrodynamic injection of the pLIVE StfA1 vector

The coding sequence of StfA1 cDNA (NM_001082543) was introduced into the pLIVE vector (Mirus Bio, Madison, WI) driven by a liver specific promoter and amplified as previously described^49^. Six-week old C57Bl6/J male mice (Janvier Labs, France) were injected with 50 µg of either pLIVE or pLIVE *Stfa1* diluted in isotonic saline equivalent to 10% of the body weight into the tail vein within 5 seconds. Blood was collected from the retro-orbital plexus with addition of 10 µM trisodium citrate two days after injection and plasma was prepared for StefA quantification by ELISA.

### Tail bleeding time

Four days after hydrodynamic or 3 hours after CA-074Me intravenous injection, a 2 mm portion of the distal tail was removed; the tail was immersed in isotonic saline (37 °C), and the initial time to complete cessation of blood flow recorded. Bleeding times were monitored for a maximum of 10 min.

### Laser-induced injury

Intravital videomicroscopy of the cremaster muscle microcirculation was performed as previously described^50^, 5–6 days after hydrodynamic or 3 hours after CA-074Me intravenous injection. Antibodies directed against platelets were infused through the jugular vein into anesthetized mice. Vessel wall injury was induced with a nitrogen dye laser (MicroPoint; Photonics Instruments), focused through the microscope objective and aimed at the vessel wall. Image analysis was performed using SlideBook (Intelligent Imaging Innovations). Fluorescence data were analyzed as previously described to determine the median of fluorescent intensity signal over time^51^. Livers were collected after *in vivo* thrombosis experiments and total RNA extracted using NucleoSpin RNA kit for Stfa1 mRNA quantification.

### Statistical analysis

The differentially expressed genes between the two groups (microarray assays) were determined using a moderated *t*-test. A change in genetic expression (Log2 fold change) > 1.5 and *p* < 0.05 was considered as significantly different. All statistical tests were done without any correction at the 5% level of significance to increase the number of genes highlighted at the cost of losing sensitivity. All analyses were conducted using the R project for Statistical Computing software (http://www.r-project.org/).

The remainder of the data are presented as mean ± SEM and were analyzed with GraphPad Prism software. Statistical significance was determined by the unpaired *t*-test or the Mann Whitney *U*-test or the Wilcoxon test or the Kruskal-Wallis test followed as appropriate by a *post hoc* test corrected for multiple comparisons or by simple linear regression. Differences were considered significant at *p* < 0.05.

## Supplementary information

Supplementary Dataset 1

## Data Availability

The data that support the findings of this study are available from the corresponding author upon reasonable request.
