# Supplementary material for: Increased levels of the megakaryocyte and platelet expressed cysteine proteases stefin A and cystatin A prevent thrombosis
Source: Sci Rep. 2019 Jul 3;9:9631. doi: 10.1038/s41598-019-45805-9 (PMC6610149; doi:10.1038/s41598-019-45805-9)
Supplement: Supplementary file 1 — Supplementary Dataset 1 [file 41598_2019_45805_MOESM1_ESM.pdf]

Supplemental Data

Increased levels of the megakaryocyte and platelet expressed cysteine proteases stefin A and cystatin A prevent thrombosis.

Anna Mezzapesa[1], Delphine Bastelica[1], Lydie Crescence[1], Marjorie Poggi[1], Michel Grino[1], Franck Peiretti[1], Laurence Panicot-Dubois[1], Annabelle Dupont[2], René Valero[1], Marie Maraninchi[1], Jean-Claude Bordet[3,4], Marie-Christine Alessi[1,*], Christophe Dubois[1], Matthias Canault[1].

1 Aix Marseille Univ, INSERM, INRA, C2VN, Marseille, 13385, France
2 CHU Lille, Université de Lille, Inserm U1011 – EG ID, Institut Pasteur de Lille, Lille, France
3 Laboratoire d'Hémostase, Centre de Biologie Est, Hospices Civils de Lyon, Bron, France
4 Laboratoire de Recherche sur l'Hémophilie, UCBL1, Lyon, France

Supplemental Table S1: Body, fat pads and muscle weight (n = 10/group) and glucose tolerance (n = 5/group) of *db/+* and *db/db* mice.

	db/+	*db/db*	P
Body weight (g)	28.8 ± 0.6	50.8 ± 1.0	< 0.0001
Mesenteric AT (mg/g bw)	10.1 ± 0.5	64.1 ± 7.2	< 0.0001
Epididymal AT (mg/g bw)	12.2 ± 0.8	48.3 ± 1.4	< 0.0001
AUC blood glucose during GTT (mg/dL/min)	136 ± 3	569 ± 10	0.0079

bw: body weight; AT: adipose tissue; AUC: area under the curve; GTT: glucose tolerance test. Data are mean ± SEM.

Supplemental Table S2

ProbeName	P	Regulation	FC	GeneSymbol	Description	EntrezGeneID	GenbankAccession	GeneName	RefSeqAccession	UniGeneID
A_51_P130660	0.046	down	-1.172846	Mrps30	Mus musculus mitochondrial ribosomal protein S30 (Mrps30), mRNA [NM_021556]	59054	NM_021556	mitochondrial ribosomal protein S30	NM_021556	Mm.143810
A_51_P371119	0.044	down	-1.1742306	Cops4	Mus musculus COP9 (constitutive photomorphogenic) homolog, subunit 4 (Arabidopsis thaliana) (Cops4), mRNA [NM_012001]	26891	NM_012001		NM_012001	Mm.957
A_66_P101107	0.049	down	-1.1759902	Brd7	bromodomain containing 7 [Source:MGI Symbol;Acc:MGI:1349766] [ENSMUST00000034085]	26992	AK046485	bromodomain containing 7		Mm.5400
A_55_P1953899	0.048	down	-1.177086	Mccc1	Mus musculus methylcrotonoyl-Coenzyme A carboxylase 1 (alpha) (Mccc1), mRNA [NM_023644]	72039	NM_023644	methylcrotonoyl-Coenzyme A carboxylase 1 (alpha)	NM_023644	Mm.249016
A_55_P2062508	0.044	down	-1.181284	Ube2e1	Mus musculus ubiquitin-conjugating enzyme E2E 1 (Ube2e1), mRNA [NM_009455]	22194	NM_009455	ubiquitin-conjugating enzyme E2E 1	NM_009455	Mm.4429
A_55_P2066518	0.033	down	-1.185698	Akt2	Mus musculus thymoma viral proto-oncogene 2 (Akt2), transcript variant 1, mRNA [NM_001110208]	11652	NM_001110208	thymoma viral proto-oncogene 2	NM_001110208	Mm.177194
A_51_P421094	0.044	down	-1.1860728	Ascc3	Mus musculus activating signal cointegrator 1 complex subunit 3 (Ascc3), mRNA [NM_198007]	77987	NM_198007	activating signal cointegrator 1 complex subunit 3	NM_198007	Mm.222497
A_52_P636830	0.042	down	-1.1867704	G3bp2	Mus musculus GTPase activating protein (SH3 domain) binding protein 2 (G3bp2), transcript variant 1, mRNA [NM_011816]	23881	NM_011816	GTPase activating protein (SH3 domain) binding protein 2	NM_011816	Mm.290530
A_51_P267740	0.032	down	-1.1873274	Zc3h15	Mus musculus zinc finger CCCH-type containing 15 (Zc3h15), mRNA [NM_026934]	69082	NM_026934	zinc finger CCCH-type containing 15	NM_026934	Mm.27804
A_52_P114282	0.050	down	-1.1876206	Armcx5	Mus musculus armadillo repeat containing, X-linked 5 (Armcx5), mRNA [NM_001009575]	494468	NM_001009575	armadillo repeat containing, X-linked 5	NM_001009575	Mm.271980
A_55_P2064230	0.045	down	-1.1902363	Tmx3	Mus musculus thioredoxin-related transmembrane protein 3 (Tmx3), mRNA [NM_198295]	67988	NM_198295	thioredoxin-related transmembrane protein 3	NM_198295	Mm.268041
A_51_P148959	0.047	down	-1.193931	Dhx36	Mus musculus DEAH (Asp-Glu-Ala-His) box polypeptide 36 (Dhx36), mRNA [NM_028136]	72162	NM_028136	DEAH (Asp-Glu-Ala-His) box polypeptide 36	NM_028136	Mm.224233
A_51_P170619	0.038	down	-1.1939338	Ddx54	Mus musculus DEAD (Asp-Glu-Ala-Asp) box polypeptide 54 (Ddx54), mRNA [NM_028041]	71990	NM_028041	DEAD (Asp-Glu-Ala-Asp) box polypeptide 54	NM_028041	Mm.491106
A_52_P652513	0.041	down	-1.1941028	Lman1	Mus musculus lectin, mannose-binding, 1 (Lman1), transcript variant 1, mRNA [NM_027400]	70361	NM_027400	lectin, mannose-binding, 1	NM_027400	Mm.290857
A_51_P438692	0.045	down	-1.1945475	Vti1b	Mus musculus vesicle transport through interaction with t-SNAREs 1B (Vti1b), mRNA [NM_016800]	53612	NM_016800	vesicle transport through interaction with t-SNAREs 1B	NM_016800	Mm.265929
A_66_P125067	0.039	down	-1.1951663	Snrpd1	Mus musculus small nuclear ribonucleoprotein D1 (Snrpd1), mRNA [NM_009226]	20641	NM_009226	small nuclear ribonucleoprotein D1	NM_009226	Mm.603
A_55_P2048434	0.030	down	-1.1957909	Hsp90ab1	Mus musculus heat shock protein 90 alpha (cytosolic), class B member 1 (Hsp90ab1), mRNA [NM_008302]	15516	NM_008302	heat shock protein 90 alpha (cytosolic), class B member 1	NM_008302	Mm.2180
A_51_P259555	0.047	down	-1.1962941	Gpatch3	Mus musculus G patch domain containing 3 (Gpatch3), mRNA [NM_172876]	242691	NM_172876	G patch domain containing 3	NM_172876	Mm.40110
A_55_P2042773	0.049	down	-1.1969837	Ctdnep1	Mus musculus CTD nuclear envelope phosphatase 1 (Ctdnep1), mRNA [NM_026017]	67181	NM_026017	CTD nuclear envelope phosphatase 1	NM_026017	Mm.41678
A_52_P237159	0.048	down	-1.1979773	Spty2d1	Mus musculus SPT2, Suppressor of Ty, domain containing 1 (S. cerevisiae) (Spty2d1), mRNA [NM_175318]	101685	NM_175318	SPT2, Suppressor of Ty, domain containing 1 (S. cerevisiae)	NM_175318	Mm.155687
A_52_P588559	0.045	down	-1.1984832	Ube2d3	Mus musculus ubiquitin-conjugating enzyme E2D 3 (Ube2d3), mRNA [NM_025356]	66105	NM_025356	ubiquitin-conjugating enzyme E2D 3	NM_025356	Mm.49884
A_51_P446477	0.046	down	-1.199092	Cldn25	Mus musculus claudin 25 (Cldn25), transcript variant 1, mRNA [NM_171826]	224250	NM_171826	claudin 25	NM_171826	Mm.29482
A_55_P2050236	0.023	down	-1.2003849	l7Rn6	Mus musculus lethal, Chr 7, Rinchik 6 (l7Rn6), mRNA [NM_026304]	67669	NM_026304	lethal, Chr 7, Rinchik 6	NM_026304	Mm.28679
A_51_P159896	0.042	down	-1.2004471	Dnajc1	Mus musculus DnaJ (Hsp40) homolog, subfamily C, member 1 (Dnajc1), transcript variant 1, mRNA [NM_007869]	13418	NM_007869	DnaJ (Hsp40) homolog, subfamily C, member 1	NM_007869	Mm.246674
A_52_P569038	0.031	down	-1.2008796	Cnot2	Mus musculus CCR4-NOT transcription complex, subunit 2 (Cnot2), transcript variant 1, mRNA [NM_001037846]	72068	NM_001037846	CCR4-NOT transcription complex, subunit 2	NM_001037846	Mm.351553
A_51_P508349	0.045	down	-1.2015662	Cdc16	Mus musculus CDC16 cell division cycle 16 (Cdc16), mRNA [NM_027276]	69957	NM_027276	CDC16 cell division cycle 16	NM_027276	Mm.182412
A_55_P1997696	0.049	down	-1.2040595	Plbd1	Mus musculus phospholipase B domain containing 1 (Plbd1), mRNA [NM_025806]	66857	NM_025806	phospholipase B domain containing 1	NM_025806	Mm.3311
A_52_P176300	0.031	down	-1.2049828	Med1	Mus musculus mediator complex subunit 1 (Med1), transcript variant 2, mRNA [NM_134027]	19014	NM_134027	mediator complex subunit 1	NM_134027	Mm.12926
A_55_P2080850	0.044	down	-1.2052994	Ptpn2	Mus musculus protein tyrosine phosphatase, non-receptor type 2 (Ptpn2), transcript variant 2, mRNA [NM_001127177]	19255	NM_001127177	protein tyrosine phosphatase, non-receptor type 2	NM_001127177	Mm.260433
A_51_P491470	0.040	down	-1.2059742	Ddx47	Mus musculus DEAD (Asp-Glu-Ala-Asp) box polypeptide 47 (Ddx47), mRNA [NM_026360]	67755	NM_026360	DEAD (Asp-Glu-Ala-Asp) box polypeptide 47	NM_026360	Mm.166524
A_66_P135194	0.023	down	-1.2064421	Yrdc	Mus musculus yrdC domain containing (E.coli) (Yrdc), mRNA [NM_153566]	230734	NM_153566	yrdC domain containing (E.coli)	NM_153566	Mm.258568
A_52_P440445	0.044	down	-1.2068115	Polr1a	Mus musculus 2 days pregnant adult female ovary cDNA, RIKEN full-length enriched library, clone:E330019F09 product:RNA polymerase 1-4 (194 kDa subunit), full insert sequence. [AK087773]	20019	AK087773	polymerase (RNA) I polypeptide A		Mm.135581

A_55_P2036250	0.037	down	-1.2073023	Cr1l	Mus musculus complement component (3b/4b) receptor 1-like (Cr1l), mRNA [NM_013499]	12946	NM_013499	complement component (3b/4b) receptor 1-like	NM_013499	Mm.301652
A_51_P199899	0.033	down	-1.2085779	Fto	Mus musculus fat mass and obesity associated (Fto), mRNA [NM_011936]	26383	NM_011936	fat mass and obesity associated	NM_011936	Mm.4375
A_52_P445307	0.044	down	-1.208629	Cwc15	Mus musculus CWC15 homolog (S. cerevisiae) (Cwc15), mRNA [NM_023153]	66070	NM_023153	CWC15 homolog (S. cerevisiae)	NM_023153	Mm.245938
A_55_P2462377	0.047	down	-1.2088484	Nsf	Mus musculus N-ethylmaleimide sensitive fusion protein (Nsf), mRNA [NM_008740]	18195	NM_008740	N-ethylmaleimide sensitive fusion protein	NM_008740	Mm.260117
A_52_P451574	0.046	down	-1.2091117	Xpr1	Mus musculus xenotropic and polytropic retrovirus receptor 1 (Xpr1), mRNA [NM_011273]	19775	NM_011273	xenotropic and polytropic retrovirus receptor 1	NM_011273	Mm.266215
A_51_P425680	0.046	down	-1.2093762	Ivd	Mus musculus isovaleryl coenzyme A dehydrogenase (Ivd), mRNA [NM_019826]	56357	NM_019826	isovaleryl coenzyme A dehydrogenase	NM_019826	Mm.6635
A_52_P620748	0.045	down	-1.2095903	Ncl	Mus musculus nucleolin (Ncl), mRNA [NM_010880]	17975	NM_010880	nucleolin	NM_010880	Mm.154378
A_55_P1992769	0.049	down	-1.2100601	Pafah1b1	Mus musculus platelet-activating factor acetylhydrolase, isoform 1b, subunit 1 (Pafah1b1), transcript variant 1, mRNA [NM_013625]	18472	NM_013625	platelet-activating factor acetylhydrolase, isoform 1b, subunit 1	NM_013625	Mm.397111
A_55_P2119155	0.038	down	-1.2106065	Set	Mus musculus SET nuclear oncogene (Set), transcript variant 2, mRNA [NM_001204875]	56086	NM_001204875	SET nuclear oncogene	NM_001204875	Mm.335942
A_51_P455807	0.030	down	-1.2113115	Ehd4	Mus musculus EH-domain containing 4 (Ehd4), mRNA [NM_133838]	98878	NM_133838	EH-domain containing 4	NM_133838	Mm.132226
A_55_P2139748	0.036	down	-1.2116237	Phf14	Mus musculus PHD finger protein 14 (Phf14), transcript variant 2, mRNA [NM_029404]	75725	NM_029404	PHD finger protein 14	NM_029404	Mm.212411
A_66_P139250	0.043	down	-1.2119316	Kctd3	Mus musculus potassium channel tetramerisation domain containing 3 (Kctd3), mRNA [NM_172650]	226823	NM_172650	potassium channel tetramerisation domain containing 3	NM_172650	Mm.209880
A_52_P1077588	0.048	down	-1.2131993	Heatr6	Mus musculus HEAT repeat containing 6 (Heatr6), mRNA [NM_145432]	217026	NM_145432	HEAT repeat containing 6	NM_145432	Mm.117874
A_51_P260513	0.047	down	-1.2142339	Polr1d	Mus musculus polymerase (RNA) I polypeptide D (Polr1d), transcript variant 1, mRNA [NM_009087]	20018	NM_009087	polymerase (RNA) I polypeptide D	NM_009087	Mm.317557
A_51_P422629	0.039	down	-1.2147576	Tbpl1	Mus musculus TATA box binding protein-like 1 (Tbpl1), mRNA [NM_011603]	237336	NM_011603	TATA box binding protein-like 1	NM_011603	Mm.86670
A_52_P610403	0.044	down	-1.2154926	Sf1	Mus musculus splicing factor 1 (Sf1), transcript variant 1, mRNA [NM_001110791]	22668	NM_001110791	splicing factor 1	NM_001110791	Mm.256422
A_55_P2061061	0.029	down	-1.2157937	Set	Mus musculus SET nuclear oncogene (Set), transcript variant 2, mRNA [NM_001204875]	56086	NM_001204875	SET nuclear oncogene	NM_001204875	Mm.335942
A_55_P2062851	0.022	down	-1.2177261	March7	Mus musculus membrane-associated ring finger (C3HC4) 7 (March7), mRNA [NM_020575]	57438	NM_020575	membrane-associated ring finger (C3HC4) 7	NM_020575	Mm.260635
A_52_P143477	0.037	down	-1.2178077	Tgoln1	Mus musculus trans-golgi network protein (Tgoln1), mRNA [NM_009443]	22134	NM_009443	trans-golgi network protein	NM_009443	Mm.246563
A_66_P136372	0.049	down	-1.2183056	Mrpl9	Mus musculus mitochondrial ribosomal protein L9 (Mrpl9), mRNA [NM_030116]	78523	NM_030116	mitochondrial ribosomal protein L9	NM_030116	Mm.218515
A_51_P379710	0.040	down	-1.2183405	Rpn1	Mus musculus ribophorin I (Rpn1), mRNA [NM_133933]	103963	NM_133933	ribophorin I	NM_133933	Mm.188544
A_55_P2074317	0.048	down	-1.2202339	Ap2b1	Mus musculus adaptor-related protein complex 2, beta 1 subunit (Ap2b1), transcript variant 1, mRNA [NM_001035854]	71770	NM_001035854	adaptor-related protein complex 2, beta 1 subunit	NM_001035854	Mm.39053
A_52_P82510	0.041	down	-1.2204394	Nmt1	Mus musculus N-myristoyltransferase 1 (Nmt1), mRNA [NM_008707]	18107	NM_008707	N-myristoyltransferase 1	NM_008707	Mm.10265
A_52_P244964	0.038	down	-1.2206587	Akap11	Mus musculus A kinase (PRKA) anchor protein 11 (Akap11), mRNA [NM_001164503]	219181	NM_001164503	A kinase (PRKA) anchor protein 11	NM_001164503	Mm.89413
A_55_P2054310	0.040	down	-1.2222257	M6pr	Mus musculus mannose-6-phosphate receptor, cation dependent (M6pr), mRNA [NM_010749]	17113	NM_010749	mannose-6-phosphate receptor, cation dependent	NM_010749	Mm.249225
A_51_P206971	0.048	down	-1.2228074	Mnat1	Mus musculus menage a trois 1 (Mnat1), mRNA [NM_008612]	17420	NM_008612	menage a trois 1	NM_008612	Mm.246750
A_51_P504314	0.048	down	-1.223921	Prpf38b	Mus musculus PRP38 pre-mRNA processing factor 38 (yeast) domain containing B (Prpf38b), mRNA [NM_025845]	66921	NM_025845	PRP38 pre-mRNA processing factor 38 (yeast) domain containing B	NM_025845	Mm.51049
A_55_P2090535	0.027	down	-1.2247803	Thrap3	Mus musculus thyroid hormone receptor associated protein 3 (Thrap3), mRNA [NM_146153]	230753	NM_146153	thyroid hormone receptor associated protein 3	NM_146153	Mm.236211
A_52_P480939	0.025	down	-1.2261467	Tnpo1	Mus musculus transportin 1 (Tnpo1), transcript variant 1, mRNA [NM_178716]	238799	NM_178716	transportin 1	NM_178716	Mm.173286
A_55_P1957319	0.034	down	-1.2267119	Krr1	Mus musculus KRR1, small subunit (SSU) processome component, homolog (yeast) (Krr1), mRNA [NM_178610]	52705	NM_178610	KRR1, small subunit (SSU) processome component, homolog (yeast)	NM_178610	Mm.34606
A_52_P484405	0.040	down	-1.2276388	Twf1	Mus musculus twinfilin, actin-binding protein, homolog 1 (Drosophila) (Twf1), mRNA [NM_008971]	19230	NM_008971	twinfilin, actin-binding protein, homolog 1 (Drosophila)	NM_008971	Mm.490250
A_55_P2060188	0.030	down	-1.2278923	Hira	histone cell cycle regulation defective homolog A (S. cerevisiae) [Source:MGI Symbol;Acc:MGI:99430] [ENSMUST00000120532]	15260		histone cell cycle regulation defective homolog A (S. cerevisiae)		
A_51_P146303	0.036	down	-1.2288724	Mvb12a	Mus musculus multivesicular body subunit 12A (Mvb12a), mRNA [NM_028617]	73711	NM_028617	multivesicular body subunit 12A	NM_028617	Mm.307770
A_51_P333949	0.034	down	-1.2299396	Ints7	Mus musculus integrator complex subunit 7 (Ints7), mRNA [NM_178632]	77065	NM_178632	integrator complex subunit 7	NM_178632	Mm.338175

A_52_P462203	0.048	down	-1.2302846	Dclre1c	Mus musculus DNA cross-link repair 1C, PSO2 homolog (S. cerevisiae) (Dclre1c), transcript variant 1, mRNA [NM_146114]	227525	NM_146114	DNA cross-link repair 1C, PSO2 homolog (S. cerevisiae)	NM_146114	
A_55_P2088640	0.035	down	-1.231014	Trip4	Mus musculus thyroid hormone receptor interactor 4 (Trip4), transcript variant 1, mRNA [NM_019797]	56404	NM_019797	thyroid hormone receptor interactor 4	NM_019797	Mm.208379
A_51_P397375	0.047	down	-1.2310587	Pet112	Mus musculus PET112 homolog (S. cerevisiae) (Pet112), mRNA [NM_144896]	229487	NM_144896	PET112 homolog (S. cerevisiae)	NM_144896	Mm.52275
A_55_P2001342	0.038	down	-1.2314707	Cacul1	Mus musculus CDK2 associated, cullin domain 1 (Cacul1), transcript variant 1, mRNA [NM_030197]	78832	NM_030197	CDK2 associated, cullin domain 1	NM_030197	Mm.259026
A_52_P431872	0.043	down	-1.231584	Ptcd3	Mus musculus pentatricopeptide repeat domain 3 (Ptcd3), mRNA [NM_027275]	69956	NM_027275	pentatricopeptide repeat domain 3	NM_027275	Mm.30256
A_51_P430620	0.046	down	-1.2316091	Slc25a12	Mus musculus solute carrier family 25 (mitochondrial carrier, Aralar), member 12 (Slc25a12), mRNA [NM_172436]	78830	NM_172436	solute carrier family 25 (mitochondrial carrier, Aralar), member 12	NM_172436	Mm.30928
A_51_P250551	0.045	down	-1.2317815	Dhx38	Mus musculus DEAH (Asp-Glu-Ala-His) box polypeptide 38 (Dhx38), mRNA [NM_178380]	64340	NM_178380	DEAH (Asp-Glu-Ala-His) box polypeptide 38	NM_178380	Mm.23705
A_51_P298666	0.039	down	-1.2318134	Scaf4	Mus musculus SR-related CTD-associated factor 4 (Scaf4), mRNA [NM_178923]	224432	NM_178923	SR-related CTD-associated factor 4	NM_178923	Mm.439811
A_52_P185343	0.039	down	-1.2320468	Gna13	guanine nucleotide binding protein, alpha 13 [Source:MGI Symbol;Acc:MGI:95768] [ENSMUST00000106702]	14674	AK011851	guanine nucleotide binding protein, alpha 13		Mm.193925
A_55_P2052754	0.038	down	-1.2332492	Pcm1	Mus musculus pericentriolar material 1 (Pcm1), mRNA [NM_023662]	18536	NM_023662	pericentriolar material 1	NM_023662	Mm.117896
A_52_P120925	0.047	down	-1.233501	Metap1	Mus musculus methionyl aminopeptidase 1 (Metap1), mRNA [NM_175224]	75624	NM_175224	methionyl aminopeptidase 1	NM_175224	Mm.26833
A_51_P245525	0.034	down	-1.2337232	ND4	mitochondrially encoded NADH dehydrogenase 4 [Source:MGI Symbol;Acc:MGI:102498] [ENSMUST00000082414]	17719	AK144853	NADH dehydrogenase subunit 4		Mm.491081
A_55_P2184931	0.037	down	-1.2355742	Rbm19	Mus musculus RNA binding motif protein 19 (Rbm19), mRNA [NM_028762]	74111	NM_028762	RNA binding motif protein 19	NM_028762	Mm.41022
A_55_P2072736	0.034	down	-1.2359774	Gmps	Mus musculus guanine monophosphate synthetase (Gmps), mRNA [NM_001033300]	229363	NM_001033300	guanine monophosphate synthetase	NM_001033300	Mm.331051
A_52_P160936	0.045	down	-1.2360088	Gusb	glucuronidase, beta [Source:MGI Symbol;Acc:MGI:95872] [ENSMUST00000122994]	110006	BC004616	glucuronidase, beta		Mm.3317
A_52_P157274	0.044	down	-1.2361504	Abi1	Mus musculus abl-interactor 1 (Abi1), transcript variant 1, mRNA [NM_001077190]	11308	NM_001077190	abl-interactor 1	NM_001077190	Mm.205647
A_55_P2166572	0.048	down	-1.2361943	Strbp	Mus musculus spermatid perinuclear RNA binding protein (Strbp), mRNA [NM_009261]	20744	NM_009261	spermatid perinuclear RNA binding protein	NM_009261	Mm.237095
A_51_P348325	0.032	down	-1.236654	Poc1b	Mus musculus POC1 centriolar protein homolog B (Chlamydomonas) (Poc1b), mRNA [NM_027740]	382406	NM_027740	POC1 centriolar protein homolog B (Chlamydomonas)	NM_027740	Mm.440656
A_52_P207614	0.025	down	-1.237024	Csnk2a2	Mus musculus 18-day embryo whole body cDNA, RIKEN full-length enriched library, clone:1110035J23 product:unclassifiable, full insert sequence. [AK004115]	13000	AK004115	casein kinase 2, alpha prime polypeptide		Mm.440348
A_66_P110687	0.034	down	-1.2383286	Taf1a	Mus musculus TATA box binding protein (Tbp)-associated factor, RNA polymerase I, A (Taf1a), transcript variant 1, mRNA [NM_021466]	21339	NM_021466	TATA box binding protein (Tbp)-associated factor, RNA polymerase I, A	NM_021466	Mm.383556
A_55_P2043702	0.027	down	-1.2406744	Tfg	Mus musculus Trk-fused gene (Tfg), transcript variant 1, mRNA [NM_019678]	21787	NM_019678	Trk-fused gene	NM_019678	Mm.425970
A_51_P384609	0.030	down	-1.2410573	Ddx10	Mus musculus DEAD (Asp-Glu-Ala-Asp) box polypeptide 10 (Ddx10), mRNA [NM_029936]	77591	NM_029936	DEAD (Asp-Glu-Ala-Asp) box polypeptide 10	NM_029936	Mm.133560
A_55_P1958246	0.034	down	-1.2415498	Tcof1	Mus musculus Treacher Collins Franceschetti syndrome 1, homolog (Tcof1), transcript variant 1, mRNA [NM_001198984]	21453	NM_001198984	Treacher Collins Franceschetti syndrome 1, homolog	NM_001198984	Mm.486251
A_52_P273594	0.029	down	-1.2424946	Zc3h14	Mus musculus zinc finger CCCH type containing 14 (Zc3h14), transcript variant 1, mRNA [NM_029334]	75553	NM_029334	zinc finger CCCH type containing 14	NM_029334	Mm.25549
A_66_P138673	0.025	down	-1.2425474	Whamm	Mus musculus WAS protein homolog associated with actin, golgi membranes and microtubules (Whamm), mRNA [NM_001004185]	434204	NM_001004185	WAS protein homolog associated with actin, golgi membranes and microtubules	NM_001004185	Mm.181641
A_52_P436318	0.021	down	-1.2427137	Rps25	Mus musculus ribosomal protein S25 (Rps25), mRNA [NM_024266]	75617	NM_024266	ribosomal protein S25	NM_024266	Mm.292027
A_51_P275953	0.007	down	-1.2442039	Dcps	Mus musculus decapping enzyme, scavenger (Dcps), mRNA [NM_027030]	69305	NM_027030	decapping enzyme, scavenger	NM_027030	Mm.229110
A_52_P483129	0.037	down	-1.2448512	Utp23	Mus musculus UTP23, small subunit (SSU) processome component, homolog (yeast) (Utp23), mRNA [NM_030132]	78581	NM_030132	UTP23, small subunit (SSU) processome component, homolog (yeast)	NM_030132	Mm.176347
A_55_P2118268	0.042	down	-1.2452255	Chpf	Mus musculus chondroitin polymerizing factor (Chpf), transcript variant 2, mRNA [NM_001001565]	74241	NM_001001565	chondroitin polymerizing factor	NM_001001565	Mm.260577
A_52_P683306	0.048	down	-1.2452731	Fam13b	Mus musculus family with sequence similarity 13, member B (Fam13b), mRNA [NM_146084]	225358	NM_146084	family with sequence similarity 13, member B	NM_146084	Mm.226899
A_55_P1983638	0.040	down	-1.2452941	Mapk6	Mus musculus mitogen-activated protein kinase 6 (Mapk6), transcript variant 2, mRNA [NM_027418]	50772	NM_027418	mitogen-activated protein kinase 6	NM_027418	Mm.480076
A_55_P2333126	0.028	down	-1.2452985	Mgea5	Mus musculus 2 days neonate thymus thymic cells cDNA, RIKEN full-length enriched library, clone:E430019C04 product:unclassifiable, full insert sequence. [AK088506]	76055	AK088506	meningioma expressed antigen 5 (hyaluronidase)		Mm.435821

A_65_P15812	0.021	down	-1.245947	Pum1	Mus musculus pumilio 1 (Drosophila) (Pum1), transcript variant 1, mRNA [NM_030722]	80912	NM_030722	pumilio 1 (Drosophila)	NM_030722	Mm.440206
A_55_P1969396	0.038	down	-1.2470728	Tmem97	Mus musculus transmembrane protein 97, mRNA (cDNA clone MGC:25841 IMAGE:4192256), complete cds. [BC018156]	69071	BC018156	transmembrane protein 97		Mm.29431
A_51_P245546	0.035	down	-1.2474837	Synrg	Mus musculus synergin, gamma (Synrg), mRNA [NM_194341]	217030	NM_194341	synergin, gamma	NM_194341	Mm.82680
A_55_P2005055	0.016	down	-1.2479379	Pepd	Mus musculus peptidase D (Pepd), mRNA [NM_008820]	18624	NM_008820	peptidase D	NM_008820	Mm.69751
A_51_P114616	0.043	down	-1.2485156	Batf	Mus musculus basic leucine zipper transcription factor, ATF-like (Batf), mRNA [NM_016767]	53314	NM_016767	basic leucine zipper transcription factor, ATF-like	NM_016767	Mm.6672
A_55_P1954156	0.028	down	-1.2487801	Wdr43	Mus musculus WD repeat domain 43 (Wdr43), mRNA [NM_175639]	72515	NM_175639	WD repeat domain 43	NM_175639	Mm.257762
A_55_P2058359	0.047	down	-1.2492125	H2afy	Mus musculus H2A histone family, member Y (H2afy), transcript variant 4, mRNA [NM_001159515]	26914	NM_001159515	H2A histone family, member Y	NM_001159515	Mm.283802
A_55_P1982951	0.018	down	-1.2514977	Dcaf10	Mus musculus DDB1 and CUL4 associated factor 10 (Dcaf10), mRNA [NM_153167]	242418	NM_153167	DDB1 and CUL4 associated factor 10	NM_153167	Mm.317668
A_52_P556281	0.013	down	-1.2515601	G3bp2	Mus musculus GTPase activating protein (SH3 domain) binding protein 2 (G3bp2), transcript variant 3, mRNA [NM_001080795]	23881	NM_001080795	GTPase activating protein (SH3 domain) binding protein 2	NM_001080795	Mm.290530
A_51_P165165	0.042	down	-1.2528867	Hibadh	Mus musculus 3-hydroxyisobutyrate dehydrogenase (Hibadh), mRNA [NM_145567]	58875	NM_145567	3-hydroxyisobutyrate dehydrogenase	NM_145567	Mm.286458
A_51_P387235	0.036	down	-1.2535199	Nampt	Mus musculus nicotinamide phosphoribosyltransferase (Nampt), mRNA [NM_021524]	59027	NM_021524	nicotinamide phosphoribosyltransferase	NM_021524	Mm.202727
A_55_P2076772	0.039	down	-1.2541276	Hspa5	Mus musculus heat shock protein 5 (Hspa5), transcript variant 2, mRNA [NM_022310]	14828	NM_022310	heat shock protein 5	NM_022310	Mm.330160
A_51_P292030	0.033	down	-1.2552516	Nus1	Mus musculus nuclear undecaprenyl pyrophosphate synthase 1 homolog (S. cerevisiae) (Nus1), mRNA [NM_030250]	52014	NM_030250	nuclear undecaprenyl pyrophosphate synthase 1 homolog (S. cerevisiae)	NM_030250	Mm.199964
A_55_P2117579	0.045	down	-1.2554921	Ppih	Mus musculus peptidyl prolyl isomerase H (Ppih), transcript variant 2, mRNA [NM_001110129]	66101	NM_001110129	peptidyl prolyl isomerase H	NM_001110129	Mm.371613
A_51_P188073	0.040	down	-1.2558675	Dnajb11	Mus musculus DnaJ (Hsp40) homolog, subfamily B, member 11 (Dnajb11), transcript variant 2, mRNA [NM_001190804]	67838	NM_001190804	DnaJ (Hsp40) homolog, subfamily B, member 11	NM_001190804	Mm.37516
A_55_P2133225	0.049	down	-1.2562582	Mmgt2	Mus musculus membrane magnesium transporter 2 (Mmgt2), mRNA [NM_175002]	216829	NM_175002	membrane magnesium transporter 2	NM_175002	Mm.295306
A_65_P10865	0.025	down	-1.2563167	Scfd1	Mus musculus Sec1 family domain containing 1 (Scfd1), mRNA [NM_029825]	76983	NM_029825	Sec1 family domain containing 1	NM_029825	Mm.216511
A_55_P2166011	0.015	down	-1.2568095	Tmem128	transmembrane protein 128 [Source:MGI Symbol;Acc:MGI:1913559] [ENSMUST00000119047]	66309	AK076679	transmembrane protein 128		Mm.256185
A_51_P426633	0.034	down	-1.2577316	Dnttip2	Mus musculus deoxynucleotidyltransferase, terminal, interacting protein 2 (Dnttip2), mRNA [NM_153806]	99480	NM_153806	deoxynucleotidyltransferase, terminal, interacting protein 2	NM_153806	Mm.398647
A_55_P2048409	0.011	down	-1.2578512	Apool	Mus musculus apolipoprotein O-like (Apool), mRNA [NM_026565]	68117	NM_026565	apolipoprotein O-like	NM_026565	Mm.161014
A_52_P153189	0.040	down	-1.2582866	Arl2bp	Mus musculus ADP-ribosylation factor-like 2 binding protein (Arl2bp), transcript variant 2, mRNA [NM_024269]	107566	NM_024269	ADP-ribosylation factor-like 2 binding protein	NM_024269	Mm.393247
A_51_P146655	0.050	down	-1.2582974	Ptgr2	Mus musculus prostaglandin reductase 2 (Ptgr2), transcript variant 1, mRNA [NM_029880]	77219	NM_029880	prostaglandin reductase 2	NM_029880	Mm.246127
A_51_P475291	0.045	down	-1.2586273	Moap1	Mus musculus modulator of apoptosis 1 (Moap1), transcript variant 1, mRNA [NM_022323]	64113	NM_022323	modulator of apoptosis 1	NM_022323	Mm.291222
A_55_P2024773	0.024	down	-1.2587409	Rbl1	Mus musculus retinoblastoma-like 1 (p107) (Rbl1), transcript variant 2, mRNA [NM_001139516]	19650	NM_001139516	retinoblastoma-like 1 (p107)	NM_001139516	Mm.244671
A_51_P502764	0.041	down	-1.260006	Hdac10	Mus musculus histone deacetylase 10 (Hdac10), transcript variant 1, mRNA [NM_199198]	170787	NM_199198	histone deacetylase 10	NM_199198	Mm.203954
A_55_P2030736	0.030	down	-1.2604971	Scmh1	Mus musculus sex comb on midleg homolog 1 (Scmh1), transcript variant 1, mRNA [NM_013883]	29871	NM_013883	sex comb on midleg homolog 1	NM_013883	Mm.427014
A_55_P2014209	0.014	down	-1.2606729	Scyl2	Mus musculus SCY1-like 2 (S. cerevisiae) (Scyl2), mRNA [NM_198021]	213326	NM_198021	SCY1-like 2 (S. cerevisiae)	NM_198021	Mm.27651
A_55_P1958245	0.015	down	-1.2620854	Tcof1	Mus musculus Treacher Collins Franceschetti syndrome 1, homolog (Tcof1), transcript variant 1, mRNA [NM_001198984]	21453	NM_001198984	Treacher Collins Franceschetti syndrome 1, homolog	NM_001198984	Mm.486251
A_51_P315595	0.013	down	-1.2634294	CYTB	mitochondrially encoded cytochrome b [Source:MGI Symbol;Acc:MGI:102501] [ENSMUST00000082421]	17711	AK131591	cytochrome b		Mm.298256
A_55_P1994817	0.040	down	-1.2642015	Taf15	Mus musculus TAF15 RNA polymerase II, TATA box binding protein (TBP)-associated factor (Taf15), mRNA [NM_027427]	70439	NM_027427	TAF15 RNA polymerase II, TATA box binding protein (TBP)-associated factor	NM_027427	Mm.181050
A_51_P242166	0.034	down	-1.2644202	Lap3	Mus musculus leucine aminopeptidase 3 (Lap3), mRNA [NM_024434]	66988	NM_024434	leucine aminopeptidase 3	NM_024434	Mm.286830
A_52_P466090	0.021	down	-1.2654865	Samhd1	Mus musculus SAM domain and HD domain, 1 (Samhd1), transcript variant 1, mRNA [NM_018851]	56045	NM_018851	SAM domain and HD domain, 1	NM_018851	Mm.248478
A_51_P505493	0.032	down	-1.2655673	Elovl5	Mus musculus ELOVL family member 5, elongation of long chain fatty acids (yeast) (Elovl5), mRNA [NM_134255]	68801	NM_134255	ELOVL family member 5, elongation of long chain fatty acids (yeast)	NM_134255	Mm.430736

A_51_P507787	0.017	down	-1.2657769	Sergef	Mus musculus secretion regulating guanine nucleotide exchange factor (Sergef), mRNA [NM_013789]	27414	NM_013789	secretion regulating guanine nucleotide exchange factor	NM_013789	Mm.76659
A_52_P417825	0.045	down	-1.2666298	Osgin2	Mus musculus oxidative stress induced growth inhibitor family member 2 (Osgin2), mRNA [NM_145950]	209212	NM_145950	oxidative stress induced growth inhibitor family member 2	NM_145950	Mm.46455
A_55_P1966209	0.014	down	-1.2668504	Fez2	Mus musculus fasciculation and elongation protein zeta 2 (zygin II) (Fez2), transcript variant 1, mRNA [NM_001285940]	225020	NM_001285940	fasciculation and elongation protein zeta 2 (zygin II)	NM_001285940	Mm.236835
A_51_P443364	0.016	down	-1.2699469	Cbll1	Mus musculus Casitas B-lineage lymphoma-like 1 (Cbll1), transcript variant 2, mRNA [NM_134048]	104836	NM_134048	Casitas B-lineage lymphoma-like 1	NM_134048	Mm.273270
A_51_P508444	0.035	down	-1.2700126	St13	Mus musculus suppression of tumorigenicity 13 (St13), mRNA [NM_133726]	70356	NM_133726	suppression of tumorigenicity 13	NM_133726	Mm.180337
A_55_P1976839	0.041	down	-1.2705139	Spata5	Mus musculus spermatogenesis associated 5 (Spata5), transcript variant 1, mRNA [NM_001163511]	57815	NM_001163511	spermatogenesis associated 5	NM_001163511	Mm.172679
A_55_P1999213	0.021	down	-1.2709659	Tdrd7	Mus musculus tudor domain containing 7 (Tdrd7), mRNA [NM_146142]	100121	NM_146142	tudor domain containing 7	NM_146142	Mm.275413
A_52_P330694	0.019	down	-1.271298	Tle1	Mus musculus transducin-like enhancer of split 1, homolog of Drosophila E(spl) (Tle1), transcript variant 4, mRNA [NM_001285531]	21885	NM_001285531	transducin-like enhancer of split 1, homolog of Drosophila E(spl)	NM_001285531	Mm.278444
A_55_P2025660	0.015	down	-1.271729	Ranbp9	Mus musculus RAN binding protein 9 (Ranbp9), mRNA [NM_019930]	56705	NM_019930	RAN binding protein 9	NM_019930	Mm.148781
A_55_P2062727	0.011	down	-1.2725472	Nasp	Mus musculus nuclear autoantigenic sperm protein (histone-binding) (Nasp), transcript variant 2, mRNA [NM_016777]	50927	NM_016777	nuclear autoantigenic sperm protein (histone-binding)	NM_016777	Mm.257181
A_51_P481644	0.025	down	-1.2727898	Mbip	Mus musculus MAP3K12 binding inhibitory protein 1 (Mbip), mRNA [NM_145442]	217588	NM_145442	MAP3K12 binding inhibitory protein 1	NM_145442	Mm.25656
A_52_P129443	0.024	down	-1.2732165	Wdpcp	Mus musculus WD repeat containing planar cell polarity effector (Wdpcp), mRNA [NM_145425]	216560	NM_145425	WD repeat containing planar cell polarity effector	NM_145425	Mm.138090
A_55_P2129291	0.037	down	-1.2746778	Dclre1c	Mus musculus DNA cross-link repair 1C, PSO2 homolog (S. cerevisiae) (Dclre1c), transcript variant 1, mRNA [NM_146114]	227525	NM_146114	DNA cross-link repair 1C, PSO2 homolog (S. cerevisiae)	NM_146114	Mm.23483
A_65_P14571	0.027	down	-1.2747934	Wdr37	Mus musculus WD repeat domain 37 (Wdr37), transcript variant 3, mRNA [NM_001039389]	207615	NM_001039389	WD repeat domain 37	NM_001039389	Mm.284654
A_52_P510215	0.024	down	-1.2751625	Traf6	Mus musculus TNF receptor-associated factor 6 (Traf6), mRNA [NM_009424]	22034	NM_009424	TNF receptor-associated factor 6	NM_009424	Mm.292729
A_51_P233815	0.029	down	-1.2754588	Tmem126a	Mus musculus transmembrane protein 126A (Tmem126a), mRNA [NM_025460]	66271	NM_025460	transmembrane protein 126A	NM_025460	Mm.28963
A_52_P545003	0.022	down	-1.2765499	Cep72	Mus musculus centrosomal protein 72 (Cep72), mRNA [NM_028959]	74470	NM_028959	centrosomal protein 72	NM_028959	Mm.73234
A_55_P2068977	0.029	down	-1.27814	Armcx3	Mus musculus armadillo repeat containing, X-linked 3 (Armcx3), mRNA [NM_027870]	71703	NM_027870	armadillo repeat containing, X-linked 3	NM_027870	Mm.67949
A_55_P2025403	0.011	down	-1.2784274	Gphn	Mus musculus gephyrin (Gphn), transcript variant 1, mRNA [NM_145965]	268566	NM_145965	gephyrin	NM_145965	Mm.341742
A_55_P2030194	0.032	down	-1.2788895	Ifna5	Mus musculus interferon alpha 5 (Ifna5), mRNA [NM_010505]	15968	NM_010505	interferon alpha 5	NM_010505	Mm.377089
A_55_P2142451	0.038	down	-1.2791123	Echdc2	Mus musculus enoyl Coenzyme A hydratase domain containing 2 (Echdc2), transcript variant 1, mRNA [NM_026728]	52430	NM_026728	enoyl Coenzyme A hydratase domain containing 2	NM_026728	Mm.270783
A_52_P35064	0.020	down	-1.2798709	Tm7sf2	Mus musculus transmembrane 7 superfamily member 2 (Tm7sf2), mRNA [NM_028454]	73166	NM_028454	transmembrane 7 superfamily member 2	NM_028454	Mm.489613
A_52_P79385	0.004	down	-1.2803761	Magt1	Mus musculus magnesium transporter 1 (Magt1), transcript variant 2, mRNA [NM_025952]	67075	NM_025952	magnesium transporter 1	NM_025952	Mm.275943
A_52_P77332	0.025	down	-1.2811247	Ireb2	Mus musculus iron responsive element binding protein 2 (Ireb2), mRNA [NM_022655]	64602	NM_022655	iron responsive element binding protein 2	NM_022655	Mm.208991
A_51_P389988	0.013	down	-1.282542	Slc40a1	Mus musculus solute carrier family 40 (iron-regulated transporter), member 1 (Slc40a1), mRNA [NM_016917]	53945	NM_016917	solute carrier family 40 (iron-regulated transporter), member 1	NM_016917	Mm.28756
A_55_P2039416	0.048	down	-1.2825705	Sgk1	Mus musculus serum/glucocorticoid regulated kinase 1 (Sgk1), transcript variant 1, mRNA [NM_001161845]	20393	NM_001161845	serum/glucocorticoid regulated kinase 1	NM_001161845	Mm.28405
A_55_P2001773	0.043	down	-1.2826418	Copg2	Mus musculus coatomer protein complex, subunit gamma 2 (Copg2), mRNA [NM_017478]	54160	NM_017478	coatomer protein complex, subunit gamma 2	NM_017478	Mm.381860
A_55_P2138366	0.031	down	-1.2828982	Xpo4	Mus musculus exportin 4 (Xpo4), mRNA [NM_020506]	57258	NM_020506	exportin 4	NM_020506	Mm.202747
A_52_P625808	0.030	down	-1.2829347	Sf3b1	Mus musculus splicing factor 3b, subunit 1 (Sf3b1), mRNA [NM_031179]	81898	NM_031179	splicing factor 3b, subunit 1	NM_031179	Mm.279736
A_52_P515094	0.036	down	-1.2833941	Tmem176a	Mus musculus transmembrane protein 176A (Tmem176a), transcript variant 1, mRNA [NM_025326]	66058	NM_025326	transmembrane protein 176A	NM_025326	Mm.27061
A_55_P2008918	0.007	down	-1.2833995	Cbll1	Mus musculus Casitas B-lineage lymphoma-like 1 (Cbll1), transcript variant 1, mRNA [NM_001253847]	104836	NM_001253847	Casitas B-lineage lymphoma-like 1	NM_001253847	Mm.273270
A_52_P179729	0.024	down	-1.2836008	Txnl4a	Mus musculus thioredoxin-like 4A (Txnl4a), transcript variant 1, mRNA [NM_025299]	27366	NM_025299	thioredoxin-like 4A	NM_025299	Mm.172411
A_55_P2143075	0.027	down	-1.2843671	Ubc	Mus musculus ubiquitin C (Ubc), mRNA [NM_019639]	22190	NM_019639	ubiquitin C	NM_019639	Mm.419997

A_52_P218537	0.039	down	-1.2851918	Hnrnpc	Mus musculus heterogeneous nuclear ribonucleoprotein C (Hnrnpc), transcript variant 1, mRNA [NM_016884]	15381	NM_016884	heterogeneous nuclear ribonucleoprotein C	NM_016884	Mm.427321
A_51_P438805	0.046	down	-1.2861092	Txnip	Mus musculus thioredoxin interacting protein (Txnip), transcript variant 1, mRNA [NM_001009935]	56338	NM_001009935	thioredoxin interacting protein	NM_001009935	Mm.410189
A_52_P490272	0.036	down	-1.286296	Nup98	Mus musculus nucleoporin 98 (Nup98), mRNA [NM_022979]	269966	NM_022979	nucleoporin 98	NM_022979	Mm.486276
A_55_P2023081	0.013	down	-1.2863218	Dnaja2	Mus musculus DnaJ (Hsp40) homolog, subfamily A, member 2 (Dnaja2), mRNA [NM_019794]	56445	NM_019794	DnaJ (Hsp40) homolog, subfamily A, member 2	NM_019794	Mm.475573
A_55_P2022693	0.018	down	-1.2889149	Tcerg1	Mus musculus transcription elongation regulator 1 (CA150) (Tcerg1), mRNA [NM_001039474]	56070	NM_001039474	transcription elongation regulator 1 (CA150)	NM_001039474	Mm.270511
A_55_P2058152	0.013	down	-1.2892914	Smek2	Mus musculus SMEK homolog 2, suppressor of mek1 (Dictyostelium) (Smek2), mRNA [NM_134034]	104570	NM_134034	SMEK homolog 2, suppressor of mek1 (Dictyostelium)	NM_134034	Mm.276386
A_52_P12001	0.005	down	-1.289346	Erp44	Mus musculus endoplasmic reticulum protein 44 (Erp44), mRNA [NM_029572]	76299	NM_029572	endoplasmic reticulum protein 44	NM_029572	Mm.317701
A_51_P101858	0.039	down	-1.2898729	Dlg1	Mus musculus discs, large homolog 1 (Drosophila) (Dlg1), transcript variant 1, mRNA [NM_007862]	13383	NM_007862	discs, large homolog 1 (Drosophila)	NM_007862	Mm.382
A_51_P321150	0.028	down	-1.2909188	Lyz2	Mus musculus lysozyme 2 (Lyz2), mRNA [NM_017372]	17105	NM_017372	lysozyme 2	NM_017372	Mm.45436
A_65_P02958	0.040	down	-1.2913251	Rnf13	Mus musculus ring finger protein 13 (Rnf13), transcript variant 1, mRNA [NM_001113413]	24017	NM_001113413	ring finger protein 13	NM_001113413	Mm.274360
A_52_P506271	0.037	down	-1.2924666	Cmtm4	Mus musculus CKLF-like MARVEL transmembrane domain containing 4 (Cmtm4), mRNA [NM_153582]	97487	NM_153582	CKLF-like MARVEL transmembrane domain containing 4	NM_153582	Mm.29658
A_55_P2056236	0.044	down	-1.2926846	Pdlim5	Mus musculus PDZ and LIM domain 5 (Pdlim5), transcript variant 2, mRNA [NM_019809]	56376	NM_019809	PDZ and LIM domain 5	NM_019809	Mm.117709
A_52_P283910	0.023	down	-1.2928461	Hnrnpdl	Mus musculus heterogeneous nuclear ribonucleoprotein D-like (Hnrnpdl), mRNA [NM_016690]	50926	NM_016690	heterogeneous nuclear ribonucleoprotein D-like	NM_016690	Mm.389579
A_55_P2088070	0.037	down	-1.2930325	Spata5	Mus musculus spermatogenesis associated 5 (Spata5), transcript variant 1, mRNA [NM_001163511]	57815	NM_001163511	spermatogenesis associated 5	NM_001163511	Mm.172679
A_51_P479808	0.021	down	-1.2934847	Mapk6	Mus musculus mitogen-activated protein kinase 6 (Mapk6), transcript variant 2, mRNA [NM_027418]	50772	NM_027418	mitogen-activated protein kinase 6	NM_027418	Mm.480076
A_55_P1975714	0.022	down	-1.2941424	Tor1aip1	Mus musculus torsin A interacting protein 1 (Tor1aip1), transcript variant 1, mRNA [NM_001160018]	208263	NM_001160018	torsin A interacting protein 1	NM_001160018	Mm.211654
A_52_P434052	0.021	down	-1.2950248	Wdr37	Mus musculus WD repeat domain 37 (Wdr37), transcript variant 2, mRNA [NM_001039388]	207615	NM_001039388	WD repeat domain 37	NM_001039388	Mm.284654
A_55_P1952414	0.011	down	-1.2952554	Odf2	Mus musculus outer dense fiber of sperm tails 2 (Odf2), transcript variant 2, mRNA [NM_001113214]	18286	NM_001113214	outer dense fiber of sperm tails 2	NM_001113214	Mm.330116
A_55_P2165239	0.021	down	-1.2953731	Abl1	Mus musculus c-abl oncogene 1, non-receptor tyrosine kinase (Abl1), transcript variant 4, mRNA [NM_001283046]	11350	NM_001283046	c-abl oncogene 1, non-receptor tyrosine kinase	NM_001283046	Mm.1318
A_55_P2037156	0.045	down	-1.2958089	Pja1	Mus musculus praja1, RING-H2 motif containing (Pja1), transcript variant 1, mRNA [NM_001083110]	18744	NM_001083110	praja1, RING-H2 motif containing	NM_001083110	Mm.8211
A_55_P2185323	0.027	down	-1.2963611	Gmps	Mus musculus guanine monophosphate synthetase (Gmps), mRNA [NM_001033300]	229363	NM_001033300	guanine monophosphate synthetase	NM_001033300	Mm.331051
A_55_P2093418	0.042	down	-1.2973139	Zmpste24	Mus musculus zinc metallopeptidase, STE24 (Zmpste24), mRNA [NM_172700]	230709	NM_172700	zinc metallopeptidase, STE24	NM_172700	Mm.34399
A_55_P2021792	0.016	down	-1.2974977	Dyrk1b	Mus musculus dual-specificity tyrosine-(Y)-phosphorylation regulated kinase 1b (Dyrk1b), transcript variant 1, mRNA [NM_001037957]	13549	NM_001037957	dual-specificity tyrosine-(Y)-phosphorylation regulated kinase 1b	NM_001037957	Mm.57249
A_66_P111987	0.018	down	-1.2975738	Brwd3	Mus musculus bromodomain and WD repeat domain containing 3 (Brwd3), mRNA [NM_001081477]	382236	NM_001081477	bromodomain and WD repeat domain containing 3	NM_001081477	Mm.426127
A_55_P1998401	0.037	down	-1.2983924	Eif2ak4	eukaryotic translation initiation factor 2 alpha kinase 4 [Source:MGI Symbol;Acc:MGI:1353427] [ENSMUST00000110869]	27103	AK077199	eukaryotic translation initiation factor 2 alpha kinase 4		
A_52_P244496	0.033	down	-1.2986863	U2surp	Mus musculus U2 snRNP-associated SURP domain containing (U2surp), transcript variant 1, mRNA [NM_001114977]	67958	NM_001114977	U2 snRNP-associated SURP domain containing	NM_001114977	Mm.292742
A_52_P649285	0.030	down	-1.2998847	Cdc26	cell division cycle 26 [Source:MGI Symbol;Acc:MGI:1913690] [ENSMUST00000134727]	66440	AK008199	cell division cycle 26		Mm.109930
A_51_P433127	0.022	down	-1.3008988	Rdm1	Mus musculus RAD52 motif 1 (Rdm1), mRNA [NM_025654]	66599	NM_025654	RAD52 motif 1	NM_025654	Mm.41449
A_55_P2174704	0.022	down	-1.3011662	Nuf2	Mus musculus NUF2, NDC80 kinetochore complex component, homolog (S. cerevisiae) (Nuf2), mRNA [NM_023284]	66977	NM_023284	NUF2, NDC80 kinetochore complex component, homolog (S. cerevisiae)	NM_023284	Mm.151315
A_51_P332580	0.006	down	-1.3017826	Polr3f	Mus musculus polymerase (RNA) III (DNA directed) polypeptide F (Polr3f), mRNA [NM_029763]	70408	NM_029763	polymerase (RNA) III (DNA directed) polypeptide F	NM_029763	Mm.389351
A_55_P2133562	0.023	down	-1.3023288	Ppih	Mus musculus peptidyl prolyl isomerase H (Ppih), transcript variant 2, mRNA [NM_001110129]	66101	NM_001110129	peptidyl prolyl isomerase H	NM_001110129	Mm.371613
A_51_P402994	0.033	down	-1.303378	Ddx3y	Mus musculus DEAD (Asp-Glu-Ala-Asp) box polypeptide 3, Y-linked (Ddx3y), mRNA [NM_012008]	26900	NM_012008	DEAD (Asp-Glu-Ala-Asp) box polypeptide 3, Y-linked	NM_012008	Mm.302938

A_51_P375431	0.008	down	-1.3036602	Phip	Mus musculus pleckstrin homology domain interacting protein (Phip), mRNA [NM_001081216]	83946	NM_001081216	pleckstrin homology domain interacting protein	NM_001081216	Mm.221688
A_66_P137102	0.007	down	-1.3041172	Spryd7	Mus musculus SPRY domain containing 7 (Spryd7), mRNA [NM_025697]	66674	NM_025697	SPRY domain containing 7	NM_025697	Mm.282706
A_52_P243599	0.011	down	-1.3042574	Hsd17b12	Mus musculus hydroxysteroid (17-beta) dehydrogenase 12 (Hsd17b12), mRNA [NM_019657]	56348	NM_019657	hydroxysteroid (17-beta) dehydrogenase 12	NM_019657	Mm.22505
A_66_P105244	0.004	down	-1.3044014	Tor1aip1	Mus musculus torsin A interacting protein 1 (Tor1aip1), transcript variant 2, mRNA [NM_144791]	208263	NM_144791	torsin A interacting protein 1	NM_144791	Mm.211654
A_51_P509098	0.031	down	-1.3045149	Asah1	Mus musculus N-acylsphingosine amidohydrolase 1 (Asah1), mRNA [NM_019734]	11886	NM_019734	N-acylsphingosine amidohydrolase 1	NM_019734	Mm.22547
A_55_P2044458	0.025	down	-1.3051924	Pramef8	Mus musculus PRAME family member 8 (Pramef8), mRNA [NM_172877]	242736	NM_172877	PRAME family member 8	NM_172877	Mm.275953
A_55_P2017759	0.023	down	-1.3053412	Evi2a	Mus musculus ecotropic viral integration site 2a (Evi2a), transcript variant 1, mRNA [NM_001033711]	14017	NM_001033711	ecotropic viral integration site 2a	NM_001033711	Mm.439665
A_55_P2057946	0.047	down	-1.3058091	Hsp90aa1	Mus musculus heat shock protein 90, alpha (cytosolic), class A member 1 (Hsp90aa1), mRNA [NM_010480]	15519	NM_010480	heat shock protein 90, alpha (cytosolic), class A member 1	NM_010480	Mm.341186
A_55_P2183493	0.010	down	-1.3060491	Spcs3	Mus musculus signal peptidase complex subunit 3 homolog (S. cerevisiae) (Spcs3), mRNA [NM_029701]	76687	NM_029701	signal peptidase complex subunit 3 homolog (S. cerevisiae)	NM_029701	Mm.86437
A_52_P78922	0.029	down	-1.3062689	Ythdc2	Mus musculus YTH domain containing 2 (Ythdc2), mRNA [NM_001163013]	240255	NM_001163013	YTH domain containing 2	NM_001163013	Mm.244482
A_55_P2020378	0.011	down	-1.3076345	Ehd4	Mus musculus EH-domain containing 4 (Ehd4), mRNA [NM_133838]	98878	NM_133838	EH-domain containing 4	NM_133838	Mm.132226
A_51_P500507	0.039	down	-1.3080754	Tmed7	Mus musculus transmembrane emp24 protein transport domain containing 7 (Tmed7), mRNA [NM_025698]	66676	NM_025698	transmembrane emp24 protein transport domain containing 7	NM_025698	Mm.296043
A_52_P276794	0.034	down	-1.3084158	Tox4	Mus musculus TOX high mobility group box family member 4 (Tox4), mRNA [NM_023434]	268741	NM_023434	TOX high mobility group box family member 4	NM_023434	Mm.246237
A_55_P2022277	0.042	down	-1.3084514	Nfe2l1	Mus musculus nuclear factor, erythroid derived 2,-like 1 (Nfe2l1), transcript variant 1, mRNA [NM_008686]	18023	NM_008686	nuclear factor, erythroid derived 2,-like 1	NM_008686	Mm.6743
A_55_P2095933	0.018	down	-1.3092077	Zranb1	Mus musculus zinc finger, RAN-binding domain containing 1 (Zranb1), mRNA [NM_207302]	360216	NM_207302	zinc finger, RAN-binding domain containing 1	NM_207302	Mm.491225
A_55_P1981205	0.041	down	-1.3097954	Xrcc4	Mus musculus X-ray repair complementing defective repair in Chinese hamster cells 4 (Xrcc4), mRNA [NM_028012]	108138	NM_028012	X-ray repair complementing defective repair in Chinese hamster cells 4	NM_028012	Mm.37531
A_52_P47645	0.016	down	-1.309832	Cul3	Mus musculus cullin 3 (Cul3), mRNA [NM_016716]	26554	NM_016716	cullin 3	NM_016716	Mm.12665
A_51_P504522	0.003	down	-1.310216	Derl1	Mus musculus Der1-like domain family, member 1 (Derl1), mRNA [NM_024207]	67819	NM_024207	Der1-like domain family, member 1	NM_024207	Mm.289387
A_51_P383218	0.024	down	-1.3102267	Tmem161a	Mus musculus transmembrane protein 161A (Tmem161a), mRNA [NM_145597]	234371	NM_145597	transmembrane protein 161A	NM_145597	Mm.23488
A_55_P2132147	0.008	down	-1.3109884	ND3	mitochondrially encoded NADH dehydrogenase 3 [Source:MGI Symbol;Acc:MGI:102499] [ENSMUST00000082411]	17718	AK139029	NADH dehydrogenase subunit 3		Mm.491158
A_55_P2019058	0.004	down	-1.3110088	Acaca	Mus musculus acetyl-Coenzyme A carboxylase alpha (Acaca), mRNA [NM_133360]	107476	NM_133360	acetyl-Coenzyme A carboxylase alpha	NM_133360	Mm.31374
A_55_P1965655	0.047	down	-1.311621	Mtmr14	Mus musculus myotubularin related protein 14 (Mtmr14), mRNA [NM_026849]	97287	NM_026849	myotubularin related protein 14	NM_026849	Mm.197816
A_51_P112237	0.029	down	-1.3118405	Angel1	Mus musculus angel homolog 1 (Drosophila) (Angel1), mRNA [NM_144524]	68737	NM_144524	angel homolog 1 (Drosophila)	NM_144524	Mm.63979
A_55_P1967928	0.013	down	-1.3120685	Srfbp1	Mus musculus serum response factor binding protein 1 (Srfbp1), mRNA [NM_026040]	67222	NM_026040	serum response factor binding protein 1	NM_026040	Mm.100617
A_51_P179498	0.003	down	-1.3122882	Fam92a	Mus musculus family with sequence similarity 92, member A (Fam92a), mRNA [NM_026558]	68099	NM_026558	family with sequence similarity 92, member A	NM_026558	Mm.25340
A_52_P299832	0.044	down	-1.3123324	Plxnc1	Mus musculus plexin C1 (Plxnc1), mRNA [NM_018797]	54712	NM_018797	plexin C1	NM_018797	Mm.256712
A_55_P2050192	0.013	down	-1.3127128	ND5	mitochondrially encoded NADH dehydrogenase 5 [Source:MGI Symbol;Acc:MGI:102496] [ENSMUST00000082418]	17721	AK142726	NADH dehydrogenase subunit 5		Mm.466752
A_51_P130757	0.025	down	-1.3127551	Acer3	Mus musculus alkaline ceramidase 3 (Acer3), mRNA [NM_025408]	66190	NM_025408	alkaline ceramidase 3	NM_025408	Mm.334041
A_55_P2158873	0.028	down	-1.3141794	Ppid	Mus musculus peptidylprolyl isomerase D (cyclophilin D) (Ppid), mRNA [NM_026352]	67738	NM_026352	peptidylprolyl isomerase D (cyclophilin D)	NM_026352	Mm.295252
A_52_P48777	0.018	down	-1.314586		Mus musculus coatomer protein complex, subunit gamma 2 (Copg2), mRNA [NM_017478]	54160	NM_017478	coatomer protein complex, subunit gamma 2	NM_017478	Mm.381860
A_55_P2166488	0.038	down	-1.315218	Cd44	Mus musculus CD44 antigen (Cd44), transcript variant 1, mRNA [NM_009851]	12505	NM_009851	CD44 antigen	NM_009851	Mm.423621
A_52_P630493	0.017	down	-1.3161464	Dnajb6	Mus musculus DnaJ (Hsp40) homolog, subfamily B, member 6 (Dnajb6), transcript variant 2, mRNA [NM_001037941]	23950	NM_001037941	DnaJ (Hsp40) homolog, subfamily B, member 6	NM_001037941	Mm.290110
A_55_P2111741	0.022	down	-1.3165975	Cbx6	Mus musculus chromobox 6 (Cbx6), mRNA [NM_028763]	494448	NM_028763	chromobox 6	NM_028763	Mm.38438
A_52_P109403	0.015	down	-1.3170265	Cebpz	Mus musculus CCAAT/enhancer binding protein zeta (Cebpz), mRNA [NM_001024806]	12607	NM_001024806	CCAAT/enhancer binding protein zeta	NM_001024806	Mm.24169
A_52_P479500	0.026	down	-1.3177739	Npepps	Mus musculus aminopeptidase puromycin sensitive (Npepps), mRNA [NM_008942]	19155	NM_008942	aminopeptidase puromycin sensitive	NM_008942	Mm.29824

A_66_P104190	0.036	down	-1.3178185	Gsk3b	Mus musculus glycogen synthase kinase 3 beta (Gsk3b), mRNA [NM_019827]	56637	NM_019827	glycogen synthase kinase 3 beta	NM_019827	Mm.394930
A_55_P2091836	0.042	down	-1.3181379	Fnip1	Mus musculus folliculin interacting protein 1 (Fnip1), mRNA [NM_173753]	216742	NM_173753	folliculin interacting protein 1	NM_173753	Mm.34087
A_55_P2149269	0.024	down	-1.3188034	Galt	Mus musculus galactose-1-phosphate uridyl transferase (Galt), mRNA [NM_016658]	14430	NM_016658	galactose-1-phosphate uridyl transferase	NM_016658	Mm.439669
A_51_P113058	0.043	down	-1.3195893	Olfr1131	Mus musculus olfactory receptor 1131 (Olfr1131), mRNA [NM_146658]	258652	NM_146658	olfactory receptor 1131	NM_146658	Mm.377615
A_52_P311148	0.018	down	-1.320005	Trappc13	Mus musculus trafficking protein particle complex 13 (Trappc13), transcript variant 4, non-coding RNA [NR_003546]	66975	NR_003546	trafficking protein particle complex 13	NR_003546	Mm.272541
A_55_P2083949	0.027	down	-1.3222601	Scfd1	Mus musculus mRNA for mKIAA0917 protein. [AK129238]	76983	AK129238	Sec1 family domain containing 1		Mm.216511
A_55_P2061025	0.049	down	-1.3225853	Hspa4	Mus musculus heat shock protein 4 (Hspa4), mRNA [NM_008300]	15525	NM_008300	heat shock protein 4	NM_008300	Mm.239865
A_55_P1983544	0.006	down	-1.3228303	Tmed5	Mus musculus transmembrane emp24 protein transport domain containing 5 (Tmed5), mRNA [NM_028876]	73130	NM_028876	transmembrane emp24 protein transport domain containing 5	NM_028876	Mm.363960
A_52_P520564	0.039	down	-1.3243117	Wasl	Mus musculus Wiskott-Aldrich syndrome-like (human) (Wasl), transcript variant 1, mRNA [NM_028459]	73178	NM_028459	Wiskott-Aldrich syndrome-like (human)	NM_028459	Mm.1574
A_55_P1961769	0.031	down	-1.3264326	Zfand5	Mus musculus zinc finger, AN1-type domain 5 (Zfand5), mRNA [NM_009551]	22682	NM_009551	zinc finger, AN1-type domain 5	NM_009551	Mm.292405
A_55_P1975380	0.003	down	-1.3268456	Nol11	Mus musculus nucleolar protein 11 (Nol11), transcript variant 1, mRNA [NM_133702]	68979	NM_133702	nucleolar protein 11	NM_133702	Mm.294617
A_55_P2141301	0.035	down	-1.3269678	Synj2bp	Mus musculus synaptojanin 2 binding protein (Synj2bp), mRNA [NM_025292]	24071	NM_025292	synaptojanin 2 binding protein	NM_025292	Mm.279603
A_66_P112006	0.048	down	-1.3273697	H2-T10	Mus musculus histocompatibility 2, T region locus 10 (H2-T10), mRNA [NM_010395]	15024	NM_010395	histocompatibility 2, T region locus 10	NM_010395	Mm.426031
A_55_P1960581	0.041	down	-1.3276006	Kdm5b	Mus musculus adult male corpora quadrigemina cDNA, RIKEN full-length enriched library, clone:B230217H22 product:jumonji, AT rich interactive domain 1B (Rbp2 like), full insert sequence. [AK163481]	75605	AK163481	lysine (K)-specific demethylase 5B		Mm.28995
A_66_P105074	0.048	down	-1.3285493	Enpp5	Mus musculus ectonucleotide pyrophosphatase/phosphodiesterase 5 (Enpp5), transcript variant 1, mRNA [NM_032003]	83965	NM_032003	ectonucleotide pyrophosphatase/phosphodiesterase 5	NM_032003	Mm.30145
A_51_P438701	0.023	down	-1.3291045	Atpaf2	Mus musculus ATP synthase mitochondrial F1 complex assembly factor 2 (Atpaf2), mRNA [NM_145427]	246782	NM_145427	ATP synthase mitochondrial F1 complex assembly factor 2	NM_145427	Mm.41651
A_55_P2175742	0.045	down	-1.3293447	Prdm10	PR domain containing 10 [Source:MGI Symbol;Acc:MGI:2682952] [ENSMUST00000136144]	382066	AK139221	PR domain containing 10		Mm.335039
A_66_P104390	0.036	down	-1.3295484	Arl3	Mus musculus ADP-ribosylation factor-like 3 (Arl3), mRNA [NM_019718]	56350	NM_019718	ADP-ribosylation factor-like 3	NM_019718	Mm.22085
A_51_P411264	0.038	down	-1.3297099	Txndc16	Mus musculus thioredoxin domain containing 16 (Txndc16), mRNA [NM_172597]	70561	NM_172597	thioredoxin domain containing 16	NM_172597	Mm.28129
A_51_P328652	0.033	down	-1.3304602	Acot7	Mus musculus acyl-CoA thioesterase 7 (Acot7), transcript variant 2, mRNA [NM_133348]	70025	NM_133348	acyl-CoA thioesterase 7	NM_133348	Mm.296191
A_52_P399717	0.037	down	-1.3306797	Ccdc50	Mus musculus coiled-coil domain containing 50 (Ccdc50), transcript variant 1, mRNA [NM_026202]	67501	NM_026202	coiled-coil domain containing 50	NM_026202	Mm.258985
A_52_P634122	0.045	down	-1.3309567	Rexo1	Mus musculus REX1, RNA exonuclease 1 homolog (S. cerevisiae) (Rexo1), mRNA [NM_025852]	66932	NM_025852	REX1, RNA exonuclease 1 homolog (S. cerevisiae)	NM_025852	Mm.1116
A_55_P2046509	0.020	down	-1.3318607	Amot	Mus musculus angiomotin (Amot), mRNA [NM_153319]	27494	NM_153319	angiomotin	NM_153319	Mm.100068
A_55_P2182433	0.046	down	-1.3328959	Atg10	Mus musculus autophagy related 10 (Atg10), mRNA [NM_025770]	66795	NM_025770	autophagy related 10	NM_025770	Mm.235385
A_52_P74427	0.009	down	-1.3332816	Mms19	Mus musculus MMS19 (MET18 S. cerevisiae) (Mms19), mRNA [NM_028152]	72199	NM_028152	MMS19 (MET18 S. cerevisiae)	NM_028152	Mm.218940
A_55_P2183438	0.022	down	-1.3343605	Runx1	Mus musculus runt related transcription factor 1 (Runx1), transcript variant 2, mRNA [NM_001111022]	12394	NM_001111022	runt related transcription factor 1	NM_001111022	Mm.4081
A_55_P2075636	0.010	down	-1.3360058	Epc2	Mus musculus enhancer of polycomb homolog 2 (Drosophila) (Epc2), mRNA [NM_172663]	227867	NM_172663	enhancer of polycomb homolog 2 (Drosophila)	NM_172663	Mm.29167
A_52_P965502	0.009	down	-1.3372289	Ssr1	Mus musculus signal sequence receptor, alpha (Ssr1), mRNA [NM_025965]	107513	NM_025965	signal sequence receptor, alpha	NM_025965	Mm.490298
A_55_P2073825	0.033	down	-1.3381276	Ocm	Mus musculus oncomodulin (Ocm), mRNA [NM_033039]	18261	NM_033039	oncomodulin	NM_033039	Mm.270533
A_55_P2078115	0.024	down	-1.3386972	Rps6kc1	Mus musculus ribosomal protein S6 kinase polypeptide 1 (Rps6kc1), mRNA [NM_178775]	320119	NM_178775	ribosomal protein S6 kinase polypeptide 1	NM_178775	Mm.220912
A_66_P124136	0.044	down	-1.3392084	Prkx	Mus musculus adult male hypothalamus cDNA, RIKEN full-length enriched library, clone:A230094I17 product:putative serine/threonine kinase, full insert sequence. [AK039088]	19108	AK039088	protein kinase, X-linked		
A_52_P228005	0.018	down	-1.3397056	Ipmk	inositol polyphosphate multikinase [Source:MGI Symbol;Acc:MGI:1916968] [ENSMUST00000118381]	69718	AK172246	inositol polyphosphate multikinase		Mm.245867
A_55_P2088028	0.049	down	-1.3406328	Mtss1	Mus musculus metastasis suppressor 1 (Mtss1), transcript variant 1, mRNA [NM_144800]	211401	NM_144800	metastasis suppressor 1	NM_144800	Mm.215481

A_55_P2377527	0.043	down	-1.3422759	Gbf1	Mus musculus golgi-specific brefeldin A-resistance factor 1 (Gbf1), mRNA [NM_178930]	107338	NM_178930	golgi-specific brefeldin A-resistance factor 1	NM_178930	Mm.271620
A_55_P2115127	0.012	down	-1.34267	Mphosph10	Mus musculus M-phase phosphoprotein 10 (U3 small nucleolar ribonucleoprotein) (Mphosph10), mRNA [NM_026483]	67973	NM_026483	M-phase phosphoprotein 10 (U3 small nucleolar ribonucleoprotein)	NM_026483	Mm.26973
A_55_P2121195	0.031	down	-1.3436837	Lcorl	Mus musculus ligand dependent nuclear receptor corepressor-like (Lcorl), transcript variant 2, mRNA [NM_172153]	209707	NM_172153	ligand dependent nuclear receptor corepressor-like	NM_172153	Mm.71498
A_55_P2017090	0.030	down	-1.3439064	Ezh1	enhancer of zeste homolog 1 (Drosophila) [Source:MGI Symbol;Acc:MGI:1097695] [ENSMUST00000100417]	14055	AK045374	enhancer of zeste homolog 1 (Drosophila)		
A_52_P43848	0.027	down	-1.3439429	Sco1	Mus musculus SCO cytochrome oxidase deficient homolog 1 (yeast) (Sco1), mRNA [NM_001040026]	52892	NM_001040026	SCO cytochrome oxidase deficient homolog 1 (yeast)	NM_001040026	Mm.129731
A_52_P59318	0.023	down	-1.3449366	Rdh10	Mus musculus retinol dehydrogenase 10 (all-trans) (Rdh10), mRNA [NM_133832]	98711	NM_133832	retinol dehydrogenase 10 (all-trans)	NM_133832	Mm.274376
A_55_P2122285	0.009	down	-1.3458835	Top3a	Mus musculus 12 days embryo eyeball cDNA, RIKEN full-length enriched library, clone:D230033L04 product:topoisomerase (DNA) III alpha, full insert sequence. [AK164443]	21975	AK164443	topoisomerase (DNA) III alpha		Mm.477819
A_51_P156849	0.015	down	-1.3464117	Paqr3	Mus musculus progestin and adipoQ receptor family member III (Paqr3), mRNA [NM_198422]	231474	NM_198422	progestin and adipoQ receptor family member III	NM_198422	Mm.332505
A_55_P2102936	0.008	down	-1.3465405	Pnrc1	Mus musculus proline-rich nuclear receptor coactivator 1 (Pnrc1), mRNA [NM_001033225]	108767	NM_001033225	proline-rich nuclear receptor coactivator 1	NM_001033225	Mm.27769
A_51_P174854	0.047	down	-1.3466494	Maged1	Mus musculus melanoma antigen, family D, 1 (Maged1), mRNA [NM_019791]	94275	NM_019791	melanoma antigen, family D, 1	NM_019791	Mm.27578
A_55_P2179020	0.014	down	-1.346697	Mrpl15	Mus musculus mitochondrial ribosomal protein L15 (Mrpl15), transcript variant 1, mRNA [NM_001177658]	27395	NM_001177658	mitochondrial ribosomal protein L15	NM_001177658	Mm.182927
A_55_P2121985	0.040	down	-1.3470703	Plcb4	Mus musculus phospholipase C, beta 4 (Plcb4), mRNA [NM_013829]	18798	NM_013829	phospholipase C, beta 4	NM_013829	Mm.38009
A_65_P05358	0.019	down	-1.3479507	ND1	mitochondrially encoded NADH dehydrogenase 1 [Source:MGI Symbol;Acc:MGI:101787] [ENSMUST00000082392]	17716	AK131578	NADH dehydrogenase subunit 1		Mm.455354
A_55_P2023607	0.046	down	-1.3483474	Ikzf1	IKAROS family zinc finger 1 [Source:MGI Symbol;Acc:MGI:1342540] [ENSMUST00000048122]	22778	AK154175	IKAROS family zinc finger 1		Mm.103545
A_55_P2164714	0.031	down	-1.3484863	C7	Mus musculus complement component 7 (C7), mRNA [NM_001243837]	109828	NM_001243837	complement component 7	NM_001243837	Mm.208034
A_55_P2022369	0.020	down	-1.3485986	Lbh	Mus musculus limb-bud and heart (Lbh), mRNA [NM_029999]	77889	NM_029999	limb-bud and heart	NM_029999	Mm.154109
A_66_P123824	0.042	down	-1.3488758	Sorl1	sortilin-related receptor, LDLR class A repeats-containing [Source:MGI Symbol;Acc:MGI:1202296] [ENSMUST00000060989]	20660	AK162665	sortilin-related receptor, LDLR class A repeats-containing		Mm.121920
A_52_P191537	0.035	down	-1.3505671	Sec24b	Sec24 related gene family, member B (S. cerevisiae) [Source:MGI Symbol;Acc:MGI:2139764] [ENSMUST00000001079]	99683	AK089573	Sec24 related gene family, member B (S. cerevisiae)		Mm.192303
A_51_P216702	0.047	down	-1.352199	Eogt	Mus musculus EGF domain-specific O-linked N-acetylglucosamine (GlcNAc) transferase (Eogt), mRNA [NM_175313]	101351	NM_175313	EGF domain-specific O-linked N-acetylglucosamine (GlcNAc) transferase	NM_175313	Mm.486365
A_51_P431772	0.009	down	-1.3533696	Ndufa3	NADH dehydrogenase (ubiquinone) 1 alpha subcomplex, 3 [Source:MGI Symbol;Acc:MGI:1913341] [ENSMUST00000150497]	66091	AK006243	NADH dehydrogenase (ubiquinone) 1 alpha subcomplex, 3		Mm.17851
A_51_P172853	0.036	down	-1.3546504	Cd14	Mus musculus CD14 antigen (Cd14), mRNA [NM_009841]	12475	NM_009841	CD14 antigen	NM_009841	Mm.3460
A_51_P272563	0.012	down	-1.3551615	Naa25	Mus musculus N(alpha)-acetyltransferase 25, NatB auxiliary subunit (Naa25), mRNA [NM_172722]	231713	NM_172722	N(alpha)-acetyltransferase 25, NatB auxiliary subunit	NM_172722	Mm.33919
A_55_P2024095	0.015	down	-1.3559334	Med1	Mus musculus mediator complex subunit 1 (Med1), transcript variant 3, mRNA [NM_001080118]	19014	NM_001080118	mediator complex subunit 1	NM_001080118	Mm.12926
A_55_P2105362	0.006	down	-1.3559908	Tmed5	Mus musculus transmembrane emp24 protein transport domain containing 5 (Tmed5), mRNA [NM_028876]	73130	NM_028876	transmembrane emp24 protein transport domain containing 5	NM_028876	Mm.363960
A_52_P237048	0.012	down	-1.355997	Prmt6	Mus musculus protein arginine N-methyltransferase 6 (Prmt6), non-coding RNA [NR_024139]	99890	NR_024139	protein arginine N-methyltransferase 6	NR_024139	Mm.36115
A_51_P306067	0.032	down	-1.3560897	Lpp	Mus musculus LIM domain containing preferred translocation partner in lipoma (Lpp), transcript variant 1, mRNA [NM_178665]	210126	NM_178665	LIM domain containing preferred translocation partner in lipoma	NM_178665	Mm.209385
A_55_P2142430	0.033	down	-1.3561776	Bank1	Mus musculus B cell scaffold protein with ankyrin repeats 1 (Bank1), mRNA [NM_001033350]	242248	NM_001033350	B cell scaffold protein with ankyrin repeats 1	NM_001033350	Mm.30832
A_55_P2003576	0.007	down	-1.3569655	Tpx2	Mus musculus TPX2, microtubule-associated protein homolog (Xenopus laevis) (Tpx2), transcript variant 1, mRNA [NM_001141977]	72119	NM_001141977	TPX2, microtubule-associated protein homolog (Xenopus laevis)	NM_001141977	Mm.407737
A_66_P124677	0.049	down	-1.3570216	Ipcef1	Mus musculus interaction protein for cytohesin exchange factors 1 (Ipcef1), transcript variant 2, mRNA [NM_001170801]	320495	NM_001170801	interaction protein for cytohesin exchange factors 1	NM_001170801	Mm.331674

A_55_P2046793	0.017	down	-1.357423	Eogt	Mus musculus EGF domain-specific O-linked N-acetylglucosamine (GlcNAc) transferase (Eogt), mRNA [NM_175313]	101351	NM_175313	EGF domain-specific O-linked N-acetylglucosamine (GlcNAc) transferase	NM_175313	Mm.486365
A_52_P513347	0.024	down	-1.3578322	Phkb	Mus musculus phosphorylase kinase beta (Phkb), mRNA [NM_199446]	102093	NM_199446	phosphorylase kinase beta	NM_199446	Mm.237296
A_55_P2101231	0.023	down	-1.3580443	Aldh7a1	Mus musculus aldehyde dehydrogenase family 7, member A1 (Aldh7a1), transcript variant 2, mRNA [NM_001127338]	110695	NM_001127338	aldehyde dehydrogenase family 7, member A1	NM_001127338	Mm.30250
A_66_P132307	0.049	down	-1.3586489	Crnkl1	Mus musculus Crn, crooked neck-like 1 (Drosophila) (Crnkl1), mRNA [NM_025820]	66877	NM_025820	Crn, crooked neck-like 1 (Drosophila)	NM_025820	Mm.248755
A_51_P482633	0.017	down	-1.3587973	Rps6ka4	Mus musculus ribosomal protein S6 kinase, polypeptide 4 (Rps6ka4), mRNA [NM_019924]	56613	NM_019924	ribosomal protein S6 kinase, polypeptide 4	NM_019924	Mm.20914
A_55_P2002054	0.034	down	-1.3597351	Phf8	Mus musculus PHD finger protein 8 (Phf8), transcript variant 1, mRNA [NM_177201]	320595	NM_177201	PHD finger protein 8	NM_177201	Mm.17156
A_51_P371174	0.006	down	-1.3613844	Bag3	Mus musculus BCL2-associated athanogene 3 (Bag3), mRNA [NM_013863]	29810	NM_013863	BCL2-associated athanogene 3	NM_013863	Mm.84073
A_55_P1978061	0.013	down	-1.3628026	Hnrnpa3	Mus musculus heterogeneous nuclear ribonucleoprotein A3 (Hnrnpa3), transcript variant b, mRNA [NM_146130]	229279	NM_146130	heterogeneous nuclear ribonucleoprotein A3	NM_146130	Mm.379375
A_55_P1962621	0.010	down	-1.363669	Chi3l7	PREDICTED: Mus musculus chitinase 3-like 7, transcript variant 1 (Chi3l7), mRNA [XM_003084535]	229687	XM_003084535	chitinase 3-like 7	XM_003084535	Mm.39224
A_55_P2328920	0.038	down	-1.3641952	Tle4	Mus musculus transducin-like enhancer of split 4, homolog of Drosophila E(spl) (Tle4), mRNA [NM_011600]	21888	NM_011600	transducin-like enhancer of split 4, homolog of Drosophila E(spl)	NM_011600	Mm.103638
A_51_P472217	0.023	down	-1.3642371	Sapcd2	Mus musculus suppressor APC domain containing 2 (Sapcd2), mRNA [NM_001081085]	72080	NM_001081085	suppressor APC domain containing 2	NM_001081085	Mm.250293
A_55_P1953894	0.036	down	-1.364248	Mccc2	Mus musculus methylcrotonoyl-Coenzyme A carboxylase 2 (beta) (Mccc2), mRNA [NM_030026]	78038	NM_030026	methylcrotonoyl-Coenzyme A carboxylase 2 (beta)	NM_030026	Mm.137327
A_51_P284608	0.048	down	-1.3663781	Cd74	Mus musculus CD74 antigen (invariant polypeptide of major histocompatibility complex, class II antigen-associated) (Cd74), transcript variant 1, mRNA [NM_001042605]	16149	NM_001042605	CD74 antigen (invariant polypeptide of major histocompatibility complex, class II antigen-associated)	NM_001042605	Mm.439737
A_55_P2243367	0.010	down	-1.3670925	Ap1g1	Mus musculus adaptor protein complex AP-1, gamma 1 subunit (Ap1g1), mRNA [NM_009677]	11765	NM_009677	adaptor protein complex AP-1, gamma 1 subunit	NM_009677	Mm.37210
A_52_P302463	0.037	down	-1.367927	Arl5a	Mus musculus ADP-ribosylation factor-like 5A (Arl5a), mRNA [NM_182994]	75423	NM_182994	ADP-ribosylation factor-like 5A	NM_182994	Mm.314378
A_55_P2001148	0.014	down	-1.3683622	Pdlim5	Mus musculus PDZ and LIM domain 5 (Pdlim5), transcript variant 2, mRNA [NM_019809]	56376	NM_019809	PDZ and LIM domain 5	NM_019809	Mm.117709
A_55_P2026218	0.019	down	-1.3683659	Igsf8	Mus musculus immunoglobulin superfamily, member 8 (Igsf8), mRNA [NM_080419]	140559	NM_080419	immunoglobulin superfamily, member 8	NM_080419	Mm.271717
A_55_P2095488	0.003	down	-1.3687825	Syncrip	Mus musculus synaptotagmin binding, cytoplasmic RNA interacting protein (Syncrip), transcript variant 1, mRNA [NM_019666]	56403	NM_019666	synaptotagmin binding, cytoplasmic RNA interacting protein	NM_019666	Mm.260545
A_66_P138976	0.010	down	-1.3688191	Lpin2	Mus musculus lipin 2 (Lpin2), transcript variant 1, mRNA [NM_001164885]	64898	NM_001164885	lipin 2	NM_001164885	Mm.227924
A_55_P2116993	0.002	down	-1.3688639	Hnrnpdl	Mus musculus heterogeneous nuclear ribonucleoprotein D-like (Hnrnpdl), mRNA [NM_016690]	50926	NM_016690	heterogeneous nuclear ribonucleoprotein D-like	NM_016690	Mm.389579
A_55_P1959480	0.015	down	-1.3690761	Urgcp	Mus musculus upregulator of cell proliferation (Urgcp), transcript variant 2, mRNA [NM_001077661]	72046	NM_001077661	upregulator of cell proliferation	NM_001077661	Mm.271657
A_55_P2016249	0.029	down	-1.369284	Ppid	Mus musculus peptidylprolyl isomerase D (cyclophilin D) (Ppid), mRNA [NM_026352]	67738	NM_026352	peptidylprolyl isomerase D (cyclophilin D)	NM_026352	Mm.295252
A_65_P09031	0.007	down	-1.3698071	Cdc42se2	Mus musculus CDC42 small effector 2 (Cdc42se2), mRNA [NM_178626]	72729	NM_178626	CDC42 small effector 2	NM_178626	Mm.29476
A_55_P2091350	0.003	down	-1.369894	ND4L	mitochondrially encoded NADH dehydrogenase 4L [Source:MGI Symbol;Acc:MGI:102497] [ENSMUST00000084013]	17720	AK140187	NADH dehydrogenase subunit 4L		Mm.491081
A_51_P292116	0.012	down	-1.3709943	Ica1	Mus musculus islet cell autoantigen 1 (Ica1), transcript variant 1, mRNA [NM_010492]	15893	NM_010492	islet cell autoantigen 1	NM_010492	Mm.275683
A_51_P157976	0.017	down	-1.3712808	Mmab	Mus musculus methylmalonic aciduria (cobalamin deficiency) type B homolog (human) (Mmab), mRNA [NM_029956]	77697	NM_029956	methylmalonic aciduria (cobalamin deficiency) type B homolog (human)	NM_029956	Mm.105182
A_55_P2050019	0.030	down	-1.3718792	Scml2	Mus musculus sex comb on midleg-like 2 (Drosophila) (Scml2), mRNA [NM_133194]	107815	NM_133194	sex comb on midleg-like 2 (Drosophila)	NM_133194	Mm.159173
A_52_P93422	0.044	down	-1.3719609	Cabin1	Mus musculus calcineurin binding protein 1 (Cabin1), mRNA [NM_172549]	104248	NM_172549	calcineurin binding protein 1	NM_172549	Mm.257404
A_55_P2130814	0.038	down	-1.372693	Zxdc	Mus musculus ZXD family zinc finger C (Zxdc), transcript variant 1, mRNA [NM_030260]	80292	NM_030260	ZXD family zinc finger C	NM_030260	Mm.100116
A_66_P121495	0.050	down	-1.3727292	Psat1	Mus musculus phosphoserine aminotransferase 1 (Psat1), transcript variant 2, mRNA [NM_001205339]	107272	NM_001205339	phosphoserine aminotransferase 1	NM_001205339	Mm.289936
A_51_P323180	0.025	down	-1.3729439	Gbp9	Mus musculus guanylate-binding protein 9 (Gbp9), mRNA [NM_172777]	236573	NM_172777	guanylate-binding protein 9	NM_172777	Mm.425261

A_55_P2251082	0.008	down	-1.3732936	Qk	Mus musculus 15 days embryo male testis cDNA, RIKEN full-length enriched library, clone:8030484F22 product:RIKEN cDNA 1110003F05 gene, full insert sequence. [AK033279]	19317	AK033279	quaking		Mm.130694
A_65_P10351	0.004	down	-1.3749474	Atp2b1	Mus musculus ATPase, Ca++ transporting, plasma membrane 1 (Atp2b1), mRNA [NM_026482]	67972	NM_026482	ATPase, Ca++ transporting, plasma membrane 1	NM_026482	Mm.166944
A_55_P2045855	0.009	down	-1.3752521	Zfp944	Mus musculus zinc finger protein 944 (Zfp944), mRNA [NM_176962]	319615	NM_176962	zinc finger protein 944	NM_176962	Mm.442162
A_51_P514712	0.037	down	-1.3766261	Parp14	Mus musculus poly (ADP-ribose) polymerase family, member 14 (Parp14), mRNA [NM_001039530]	547253	NM_001039530	poly (ADP-ribose) polymerase family, member 14	NM_001039530	Mm.244406
A_66_P129730	0.017	down	-1.3770076	Pdia3	Mus musculus protein disulfide isomerase associated 3 (Pdia3), mRNA [NM_007952]	14827	NM_007952	protein disulfide isomerase associated 3	NM_007952	Mm.263177
A_52_P243102	0.019	down	-1.3771131	Kctd3	Mus musculus potassium channel tetramerisation domain containing 3 (Kctd3), mRNA [NM_172650]	226823	NM_172650	potassium channel tetramerisation domain containing 3	NM_172650	Mm.209880
A_65_P19780	0.023	down	-1.3781765	Klf9	Mus musculus Kruppel-like factor 9 (Klf9), mRNA [NM_010638]	16601	NM_010638	Kruppel-like factor 9	NM_010638	Mm.291595
A_55_P2156761	0.006	down	-1.378928	Slc10a7	Mus musculus solute carrier family 10 (sodium/bile acid cotransporter family), member 7 (Slc10a7), transcript variant a, mRNA [NM_029736]	76775	NM_029736	solute carrier family 10 (sodium/bile acid cotransporter family), member 7	NM_029736	Mm.334631
A_55_P2118183	0.010	down	-1.3791059	Tnks2	Mus musculus tankyrase, TRF1-interacting ankyrin-related ADP-ribose polymerase 2 (Tnks2), mRNA [NM_001163635]	74493	NM_001163635	tankyrase, TRF1-interacting ankyrin-related ADP-ribose polymerase 2	NM_001163635	Mm.249310
A_51_P174736	0.008	down	-1.379411	Krr1	Mus musculus KRR1, small subunit (SSU) processome component, homolog (yeast) (Krr1), mRNA [NM_178610]	52705	NM_178610	KRR1, small subunit (SSU) processome component, homolog (yeast)	NM_178610	Mm.34606
A_51_P209413	0.048	down	-1.3803025	Akap3	Mus musculus A kinase (PRKA) anchor protein 3 (Akap3), mRNA [NM_009650]	11642	NM_009650	A kinase (PRKA) anchor protein 3	NM_009650	Mm.87748
A_55_P2013058	0.038	down	-1.3808817	Shb	Mus musculus src homology 2 domain-containing transforming protein B (Shb), mRNA [NM_001033306]	230126	NM_001033306	src homology 2 domain-containing transforming protein B	NM_001033306	Mm.251716
A_51_P235440	0.007	down	-1.3818688	Pelp1	Mus musculus adult male testis cDNA, RIKEN full-length enriched library, clone:4930563C04 product:hypothetical protein, full insert sequence. [AK016197]	75273	AK016197	proline, glutamic acid and leucine rich protein 1		Mm.340601
A_66_P122053	0.011	down	-1.3819252	Kcnq5	Mus musculus potassium voltage-gated channel, subfamily Q, member 5 (Kcnq5), transcript variant 1, mRNA [NM_001160139]	226922	NM_001160139	potassium voltage-gated channel, subfamily Q, member 5	NM_001160139	Mm.336519
A_51_P441286	0.020	down	-1.3839438	Ttc5	Mus musculus tetratricopeptide repeat domain 5 (Ttc5), transcript variant 2, mRNA [NM_177625]	219022	NM_177625	tetratricopeptide repeat domain 5	NM_177625	Mm.276362
A_51_P263220	0.022	down	-1.3840712	Taf5	Mus musculus TAF5 RNA polymerase II, TATA box binding protein (TBP)-associated factor (Taf5), mRNA [NM_177342]	226182	NM_177342	TAF5 RNA polymerase II, TATA box binding protein (TBP)-associated factor	NM_177342	Mm.301522
A_55_P2023191	0.012	down	-1.38412	Polg2	Mus musculus polymerase (DNA directed), gamma 2, accessory subunit (Polg2), transcript variant 1, mRNA [NM_015810]	50776	NM_015810	polymerase (DNA directed), gamma 2, accessory subunit	NM_015810	Mm.859
A_66_P123643	0.007	down	-1.3848946	Ptbp2	Mus musculus polypyrimidine tract binding protein 2 (Ptbp2), mRNA [NM_019550]	56195	NM_019550	polypyrimidine tract binding protein 2	NM_019550	Mm.29966
A_55_P1955587	0.013	down	-1.3856962	Hspd1	Mus musculus heat shock protein 1 (chaperonin) (Hspd1), mRNA [NM_010477]	15510	NM_010477	heat shock protein 1 (chaperonin)	NM_010477	Mm.1777
A_52_P443544	0.004	down	-1.3872896	Gdpgp1	Mus musculus GDP-D-glucose phosphorylase 1 (Gdpgp1), mRNA [NM_178752]	269952	NM_178752	GDP-D-glucose phosphorylase 1	NM_178752	Mm.187733
A_55_P2117699	0.019	down	-1.3873272	Pak7	Mus musculus p21 protein (Cdc42/Rac)-activated kinase 7 (Pak7), mRNA [NM_172858]	241656	NM_172858	p21 protein (Cdc42/Rac)-activated kinase 7	NM_172858	Mm.131572
A_51_P177092	0.012	down	-1.3874998	Stat4	Mus musculus signal transducer and activator of transcription 4 (Stat4), mRNA [NM_011487]	20849	NM_011487	signal transducer and activator of transcription 4	NM_011487	Mm.1550
A_51_P410744	0.030	down	-1.3876765	Dgke	Mus musculus diacylglycerol kinase, epsilon (Dgke), mRNA [NM_019505]	56077	NM_019505	diacylglycerol kinase, epsilon	NM_019505	Mm.153695
A_52_P520607	0.042	down	-1.3878212	Ankrd22	Mus musculus ankyrin repeat domain 22 (Ankrd22), mRNA [NM_024204]	52024	NM_024204	ankyrin repeat domain 22	NM_024204	Mm.183030
A_55_P2091461	0.046	down	-1.3882496	Casp4	Mus musculus caspase 4, apoptosis-related cysteine peptidase (Casp4), mRNA [NM_007609]	12363	NM_007609	caspase 4, apoptosis-related cysteine peptidase	NM_007609	Mm.1569
A_55_P2176087	0.031	down	-1.3911257	Cox11	cytochrome c oxidase assembly protein 11 [Source:MGI Symbol;Acc:MGI:1917052] [ENSMUST00000099960]	69802		cytochrome c oxidase assembly protein 11		
A_55_P2112205	0.027	down	-1.3911382	Cep110	centrosomal protein 110 [Source:MGI Symbol;Acc:MGI:1889576] [ENSMUST00000123884]	26920	BC060146	centrosomal protein 110		Mm.231332
A_55_P2033586	0.013	down	-1.3921851	Mmab	Mus musculus methylmalonic aciduria (cobalamin deficiency) type B homolog (human) (Mmab), mRNA [NM_029956]	77697	NM_029956	methylmalonic aciduria (cobalamin deficiency) type B homolog (human)	NM_029956	Mm.105182
A_51_P129803	0.015	down	-1.3923694	Cacybp	Mus musculus calcyclin binding protein (Cacybp), mRNA [NM_009786]	12301	NM_009786	calcyclin binding protein	NM_009786	Mm.10702
A_55_P2095345	0.037	down	-1.3926655	Rara	Mus musculus retinoic acid receptor, alpha (Rara), transcript variant 2, mRNA [NM_001177302]	19401	NM_001177302	retinoic acid receptor, alpha	NM_001177302	Mm.439744

A_55_P1988975	0.034	down	-1.3942444	Ms4a4b	membrane-spanning 4-domains, subfamily A, member 4B [Source:MGI Symbol;Acc:MGI:1913083] [ENSMUST00000142135]	60361	AK080934	membrane-spanning 4-domains, subfamily A, member 4B		Mm.33957
A_52_P398989	0.009	down	-1.3956825	Cytip	Mus musculus cytohesin 1 interacting protein (Cytip), mRNA [NM_139200]	227929	NM_139200	cytohesin 1 interacting protein	NM_139200	Mm.273905
A_52_P388359	0.040	down	-1.397864	Prdx6	Mus musculus peroxiredoxin 6 (Prdx6), mRNA [NM_007453]	11758	NM_007453	peroxiredoxin 6	NM_007453	Mm.186185
A_55_P2058864	0.026	down	-1.3981634	Mvk	Mus musculus mevalonate kinase (Mvk), mRNA [NM_023556]	17855	NM_023556	mevalonate kinase	NM_023556	Mm.28088
A_52_P660922	0.009	down	-1.3982254	Dock1	Mus musculus dedicator of cytokinesis 1 (Dock1), mRNA [NM_001033420]	330662	NM_001033420	dedicator of cytokinesis 1	NM_001033420	Mm.436572
A_55_P2053491	0.007	down	-1.3982843	Pdia6	Mus musculus protein disulfide isomerase associated 6 (Pdia6), mRNA [NM_027959]	71853	NM_027959	protein disulfide isomerase associated 6	NM_027959	Mm.222825
A_55_P2147200	0.011	down	-1.4036276	Ttc37	Mus musculus tetratricopeptide repeat domain 37 (Ttc37), mRNA [NM_001081352]	218343	NM_001081352	tetratricopeptide repeat domain 37	NM_001081352	Mm.277964
A_52_P264902	0.003	down	-1.4053743	Dnajc3	Mus musculus DnaJ (Hsp40) homolog, subfamily C, member 3 (Dnajc3), mRNA [NM_008929]	1E+08	NM_008929	DnaJ (Hsp40) homolog, subfamily C, member 3	NM_008929	Mm.12616
A_51_P159352	0.014	down	-1.4056232	Sub1	Mus musculus SUB1 homolog (S. cerevisiae) (Sub1), mRNA [NM_011294]	20024	NM_011294	SUB1 homolog (S. cerevisiae)	NM_011294	Mm.41746
A_52_P127459	0.011	down	-1.4066272	Caprin1	Mus musculus cell cycle associated protein 1 (Caprin1), transcript variant 4, mRNA [NM_001111291]	53872	NM_001111291	cell cycle associated protein 1	NM_001111291	Mm.427589
A_51_P228817	0.016	down	-1.4066877	Stau2	staufen (RNA binding protein) homolog 2 (Drosophila) [Source:MGI Symbol;Acc:MGI:1352508] [ENSMUST00000127420]	29819	AK171076	staufen (RNA binding protein) homolog 2 (Drosophila)		Mm.216257
A_51_P345836	0.002	down	-1.4070926	Prkra	Mus musculus protein kinase, interferon inducible double stranded RNA dependent activator (Prkra), mRNA [NM_011871]	23992	NM_011871	protein kinase, interferon inducible double stranded RNA dependent activator	NM_011871	Mm.277250
A_52_P618807	0.004	down	-1.4104921	Crk	Mus musculus v-crk sarcoma virus CT10 oncogene homolog (avian) (Crk), transcript variant 2, mRNA [NM_133656]	12928	NM_133656	v-crk sarcoma virus CT10 oncogene homolog (avian)	NM_133656	Mm.280125
A_55_P1983828	0.014	down	-1.4117908	Zbtb11	Mus musculus zinc finger and BTB domain containing 11 (Zbtb11), mRNA [NM_173026]	271377	NM_173026	zinc finger and BTB domain containing 11	NM_173026	Mm.282814
A_52_P80567	0.017	down	-1.4121714	Nbas	Mus musculus neuroblastoma amplified sequence (Nbas), mRNA [NM_027706]	71169	NM_027706	neuroblastoma amplified sequence	NM_027706	Mm.177520
A_55_P2064862	0.015	down	-1.4123824	Ica1	Mus musculus islet cell autoantigen 1 (Ica1), transcript variant 2, mRNA [NM_001252266]	15893	NM_001252266	islet cell autoantigen 1	NM_001252266	Mm.275683
A_55_P1975505	0.042	down	-1.4146577	Chi3l7	PREDICTED: Mus musculus chitinase 3-like 7, transcript variant 1 (Chi3l7), mRNA [XM_003084535]	229687	XM_003084535	chitinase 3-like 7	XM_003084535	Mm.39224
A_55_P1994997	0.018	down	-1.4153621	Arhgap11a	Mus musculus Rho GTPase activating protein 11A (Arhgap11a), mRNA [NM_181416]	228482	NM_181416	Rho GTPase activating protein 11A	NM_181416	Mm.491249
A_51_P275591	0.009	down	-1.4177175	Zfp292	Mus musculus zinc finger protein 292 (Zfp292), mRNA [NM_013889]	30046	NM_013889	zinc finger protein 292	NM_013889	Mm.38193
A_51_P164393	0.048	down	-1.4180589	Txndc2	Mus musculus thioredoxin domain containing 2 (spermatozoa) (Txndc2), transcript variant 2, mRNA [NM_153519]	213272	NM_153519	thioredoxin domain containing 2 (spermatozoa)	NM_153519	Mm.255732
A_55_P2148844	0.043	down	-1.4183394	Bend4	Mus musculus BEN domain containing 4 (Bend4), mRNA [NM_001164806]	666938	NM_001164806	BEN domain containing 4	NM_001164806	Mm.101339
A_52_P569348	0.046	down	-1.4187428	Dbt	Mus musculus dihydrolipoamide branched chain transacylase E2 (Dbt), mRNA [NM_010022]	13171	NM_010022	dihydrolipoamide branched chain transacylase E2	NM_010022	Mm.3636
A_51_P342871	0.042	down	-1.4208354	S100a10	Mus musculus S100 calcium binding protein A10 (calpactin) (S100a10), mRNA [NM_009112]	20194	NM_009112	S100 calcium binding protein A10 (calpactin)	NM_009112	Mm.1
A_55_P2067971	0.026	down	-1.4225783	Prss54	Mus musculus protease, serine, 54 (Prss54), mRNA [NM_027640]	70993	NM_027640	protease, serine, 54	NM_027640	Mm.486561
A_51_P122246	0.011	down	-1.4241581	Creld2	Mus musculus cysteine-rich with EGF-like domains 2 (Creld2), mRNA [NM_029720]	76737	NM_029720	cysteine-rich with EGF-like domains 2	NM_029720	Mm.292567
A_51_P188772	0.014	down	-1.4268419	Arhgef28	Mus musculus Rho guanine nucleotide exchange factor (GEF) 28 (Arhgef28), mRNA [NM_012026]	110596	NM_012026	Rho guanine nucleotide exchange factor (GEF) 28	NM_012026	Mm.252718
A_52_P134639	0.001	down	-1.427827	Tgoln2	Mus musculus trans-golgi network protein 2 (Tgoln2), mRNA [NM_009444]	22135	NM_009444	trans-golgi network protein 2	NM_009444	Mm.246563
A_55_P2100705	0.010	down	-1.4281346	Auh	AU RNA binding protein/enoyl-coenzyme A hydratase [Source:MGI Symbol;Acc:MGI:1338011] [ENSMUST00000120535]	11992	AK019978	AU RNA binding protein/enoyl-coenzyme A hydratase		
A_51_P265219	0.038	down	-1.428797	Ppm1k	Mus musculus protein phosphatase 1K (PP2C domain containing) (Ppm1k), mRNA [NM_175523]	243382	NM_175523	protein phosphatase 1K (PP2C domain containing)	NM_175523	Mm.396893
A_55_P2135466	0.043	down	-1.4296662	Srsf12	serine/arginine-rich splicing factor 12 [Source:MGI Symbol;Acc:MGI:2661424] [ENSMUST00000108159]	272009		serine/arginine-rich splicing factor 12		
A_51_P362066	0.005	down	-1.4303567	Chi3l1	Mus musculus chitinase 3-like 1 (Chi3l1), mRNA [NM_007695]	12654	NM_007695	chitinase 3-like 1	NM_007695	Mm.38274
A_55_P1985890	0.009	down	-1.4311517	Tiparp	Mus musculus TCDD-inducible poly(ADP-ribose) polymerase (Tiparp), mRNA [NM_178892]	99929	NM_178892	TCDD-inducible poly(ADP-ribose) polymerase	NM_178892	Mm.246398
A_52_P384574	0.007	down	-1.4328103	Stard4	Mus musculus StAR-related lipid transfer (START) domain containing 4 (Stard4), mRNA [NM_133774]	170459	NM_133774	StAR-related lipid transfer (START) domain containing 4	NM_133774	Mm.127058

A_52_P269158	0.029	down	-1.4333317	Pid1	Mus musculus phosphotyrosine interaction domain containing 1 (Pid1), mRNA [NM_001003948]	98496	NM_001003948	phosphotyrosine interaction domain containing 1	NM_001003948	Mm.187470
A_52_P627816	0.045	down	-1.4333432	Tgm1	Mus musculus transglutaminase 1, K polypeptide (Tgm1), transcript variant 2, mRNA [NM_019984]	21816	NM_019984	transglutaminase 1, K polypeptide	NM_019984	Mm.41964
A_52_P117294	0.009	down	-1.4343919	Fryl	furry homolog-like (Drosophila) [Source:MGI Symbol;Acc:MGI:1919563] [ENSMUST00000153923]	72313	AK036035	furry homolog-like (Drosophila)		
A_52_P507578	0.043	down	-1.4347504	Slamf6	Mus musculus SLAM family member 6 (Slamf6), mRNA [NM_030710]	30925	NM_030710	SLAM family member 6	NM_030710	Mm.486404
A_52_P231170	0.011	down	-1.4382974	Megf9	Mus musculus multiple EGF-like-domains 9 (Megf9), mRNA [NM_172694]	230316	NM_172694	multiple EGF-like-domains 9	NM_172694	Mm.251188
A_55_P1997415	0.007	down	-1.4396964	Exoc6b	Mus musculus exocyst complex component 6B (Exoc6b), mRNA [NM_177077]	75914	NM_177077	exocyst complex component 6B	NM_177077	Mm.159621
A_51_P510663	0.013	down	-1.4407116	Supt3	Mus musculus suppressor of Ty 3 (Supt3), mRNA [NM_178652]	109115	NM_178652	suppressor of Ty 3	NM_178652	Mm.431327
A_55_P2181381	0.045	down	-1.4411551	Dfnb59	Mus musculus deafness, autosomal recessive 59 (human) (Dfnb59), mRNA [NM_001080711]	381375	NM_001080711	deafness, autosomal recessive 59 (human)	NM_001080711	Mm.330312
A_52_P510107	0.038	down	-1.4419802	Nudt15	Mus musculus nudix (nucleoside diphosphate linked moiety X)-type motif 15 (Nudt15), mRNA [NM_172527]	214254	NM_172527	nudix (nucleoside diphosphate linked moiety X)-type motif 15	NM_172527	Mm.386852
A_55_P2002846	0.023	down	-1.4425011	Cystm1	Mus musculus cysteine-rich transmembrane module containing 1 (Cystm1), mRNA [NM_001081365]	66060	NM_001081365	cysteine-rich transmembrane module containing 1	NM_001081365	Mm.272527
A_55_P2139107	0.009	down	-1.4430538	Rab6a	Mus musculus adult male hippocampus cDNA, RIKEN full-length enriched library, clone:C630031P05 product:RAB6, member RAS oncogene family, full insert sequence. [AK083262]	19346	AK083262	RAB6A, member RAS oncogene family		Mm.28650
A_52_P368650	0.017	down	-1.4455712	Zswim4	Mus musculus zinc finger SWIM-type containing 4 (Zswim4), mRNA [NM_172503]	212168	NM_172503	zinc finger SWIM-type containing 4	NM_172503	Mm.117094
A_52_P217875	0.039	down	-1.4470721	H3f3b	Mus musculus H3 histone, family 3B (H3f3b), mRNA [NM_008211]	15081	NM_008211	H3 histone, family 3B	NM_008211	Mm.371563
A_55_P1953387	0.030	down	-1.4470842	Fabp5	Mus musculus fatty acid binding protein 5, epidermal (Fabp5), transcript variant 2, mRNA [NM_001272097]	16592	NM_001272097	fatty acid binding protein 5, epidermal	NM_001272097	Mm.741
A_55_P2165324	0.017	down	-1.4473218	Acsl3	Mus musculus acyl-CoA synthetase long-chain family member 3 (Acsl3), transcript variant 1, mRNA [NM_028817]	74205	NM_028817	acyl-CoA synthetase long-chain family member 3	NM_028817	Mm.276016
A_55_P2120089	0.007	down	-1.4476309	Zmynd11	Mus musculus zinc finger, MYND domain containing 11 (Zmynd11), transcript variant 3, mRNA [NM_001199141]	66505	NM_001199141	zinc finger, MYND domain containing 11	NM_001199141	Mm.397307
A_66_P106133	0.029	down	-1.4477448	Magi3	Mus musculus membrane associated guanylate kinase, WW and PDZ domain containing 3 (Magi3), transcript variant 2, mRNA [NM_133853]	99470	NM_133853	membrane associated guanylate kinase, WW and PDZ domain containing 3	NM_133853	Mm.264849
A_55_P1973339	0.039	down	-1.4512143	Mapk1	Mus musculus mitogen-activated protein kinase 1 (Mapk1), transcript variant 2, mRNA [NM_001038663]	26413	NM_001038663	mitogen-activated protein kinase 1	NM_001038663	Mm.196581
A_52_P640386	0.007	down	-1.4517717	Usp53	Mus musculus ubiquitin specific peptidase 53 (Usp53), mRNA [NM_133857]	99526	NM_133857	ubiquitin specific peptidase 53	NM_133857	Mm.491248
A_55_P1983508	0.039	down	-1.4523387	Nr4a2	Mus musculus nuclear receptor subfamily 4, group A, member 2 (Nr4a2), transcript variant 1, mRNA [NM_013613]	18227	NM_013613	nuclear receptor subfamily 4, group A, member 2	NM_013613	Mm.3507
A_55_P2090924	0.048	down	-1.453549	Tbc1d8b	Mus musculus TBC1 domain family, member 8B (Tbc1d8b), mRNA [NM_001081499]	245638	NM_001081499	TBC1 domain family, member 8B	NM_001081499	Mm.275869
A_52_P579517	0.008	down	-1.4538085	Cox10	Mus musculus cytochrome c oxidase assembly protein 10 (Cox10), mRNA [NM_178379]	70383	NM_178379	cytochrome c oxidase assembly protein 10	NM_178379	Mm.340211
A_52_P37681	0.010	down	-1.4547851	Fkbp9	Mus musculus FK506 binding protein 9 (Fkbp9), mRNA [NM_012056]	27055	NM_012056	FK506 binding protein 9	NM_012056	Mm.20943
A_55_P2083317	0.000	down	-1.4548528	Calml3	Mus musculus calmodulin-like 3 (Calml3), mRNA [NM_027416]	70405	NM_027416	calmodulin-like 3	NM_027416	Mm.20079
A_52_P494622	0.031	down	-1.455083	Nr4a2	Mus musculus nuclear receptor subfamily 4, group A, member 2 (Nr4a2), transcript variant 1, mRNA [NM_013613]	18227	NM_013613	nuclear receptor subfamily 4, group A, member 2	NM_013613	Mm.3507
A_55_P2160286	0.043	down	-1.4550873	Olfm1	Mus musculus olfactomedin 1 (Olfm1), transcript variant 2, mRNA [NM_001038612]	56177	NM_001038612	olfactomedin 1	NM_001038612	Mm.43278
A_52_P211208	0.035	down	-1.4552486	Aar2	Mus musculus AAR2 splicing factor homolog (S. cerevisiae) (Aar2), transcript variant 2, mRNA [NM_026661]	68295	NM_026661	AAR2 splicing factor homolog (S. cerevisiae)	NM_026661	Mm.253648
A_51_P469968	0.013	down	-1.4561074	H2-M3	Mus musculus histocompatibility 2, M region locus 3 (H2-M3), mRNA [NM_013819]	14991	NM_013819	histocompatibility 2, M region locus 3	NM_013819	Mm.14437
A_55_P2054420	0.008	down	-1.4618329	Tcf4	Mus musculus transcription factor 4 (Tcf4), transcript variant 1, mRNA [NM_013685]	21413	NM_013685	transcription factor 4	NM_013685	Mm.4269
A_55_P2151616	0.013	down	-1.4626148	Hbs1l	Mus musculus Hbs1-like (S. cerevisiae) (Hbs1l), transcript variant 3, mRNA [NM_001145209]	56422	NM_001145209	Hbs1-like (S. cerevisiae)	NM_001145209	Mm.25527
A_51_P129464	0.015	down	-1.4634042	Scd2	Mus musculus stearoyl-Coenzyme A desaturase 2 (Scd2), mRNA [NM_009128]	20250	NM_009128	stearoyl-Coenzyme A desaturase 2	NM_009128	Mm.487021
A_52_P177373	0.031	down	-1.4665991	Intu	Mus musculus inturned planar cell polarity effector homolog (Drosophila) (Intu), mRNA [NM_175515]	380614	NM_175515	inturned planar cell polarity effector homolog (Drosophila)	NM_175515	Mm.432345
A_52_P199633	0.030	down	-1.4674801	Trim30d	Mus musculus tripartite motif-containing 30D (Trim30d), transcript variant 1, mRNA [NM_199146]	209387	NM_199146	tripartite motif-containing 30D	NM_199146	Mm.277377

A_66_P140185	0.013	down	-1.468543	Rgs9	Mus musculus regulator of G-protein signaling 9 (Rgs9), transcript variant 2, mRNA [NM_001165934]	19739	NM_001165934	regulator of G-protein signaling 9	NM_001165934	Mm.38548
A_52_P479269	0.011	down	-1.4685664	Sdc1	Mus musculus syndecan 1 (Sdc1), mRNA [NM_011519]	20969	NM_011519	syndecan 1	NM_011519	Mm.2580
A_52_P624149	0.001	down	-1.4691756	Hsp90b1	Mus musculus heat shock protein 90, beta (Grp94), member 1 (Hsp90b1), mRNA [NM_011631]	22027	NM_011631	heat shock protein 90, beta (Grp94), member 1	NM_011631	Mm.87773
A_51_P408363	0.003	down	-1.4710954	Cfp	Mus musculus complement factor properdin (Cfp), mRNA [NM_008823]	18636	NM_008823	complement factor properdin	NM_008823	Mm.3064
A_55_P2121682	0.002	down	-1.4721872	Alox12b	Mus musculus arachidonate 12-lipoxygenase, 12R type (Alox12b), mRNA [NM_009659]	11686	NM_009659	arachidonate 12-lipoxygenase, 12R type	NM_009659	Mm.340329
A_55_P2045109	0.032	down	-1.4723727	Tm7sf3	Mus musculus transmembrane 7 superfamily member 3 (Tm7sf3), mRNA [NM_026281]	67623	NM_026281	transmembrane 7 superfamily member 3	NM_026281	Mm.278037
A_55_P1952547	0.024	down	-1.4728951	Sh3rf1	Mus musculus SH3 domain containing ring finger 1 (Sh3rf1), mRNA [NM_021506]	59009	NM_021506	SH3 domain containing ring finger 1	NM_021506	Mm.27949
A_55_P1968395	0.044	down	-1.4747863	Cradd	Mus musculus CASP2 and RIPK1 domain containing adaptor with death domain (Cradd), mRNA [NM_009950]	12905	NM_009950	CASP2 and RIPK1 domain containing adaptor with death domain	NM_009950	Mm.473739
A_55_P2122020	0.035	down	-1.4753872	Klf4	Mus musculus Kruppel-like factor 4 (gut) (Klf4), mRNA [NM_010637]	16600	NM_010637	Kruppel-like factor 4 (gut)	NM_010637	Mm.4325
A_55_P2071176	0.043	down	-1.4757532	Il1f9	Mus musculus interleukin 1 family, member 9 (Il1f9), mRNA [NM_153511]	215257	NM_153511	interleukin 1 family, member 9	NM_153511	Mm.249379
A_52_P373798	0.003	down	-1.4765037	Zdhhc3	Mus musculus zinc finger, DHHC domain containing 3 (Zdhhc3), mRNA [NM_026917]	69035	NM_026917	zinc finger, DHHC domain containing 3	NM_026917	Mm.28300
A_55_P2156062	0.038	down	-1.4768679	Pick1	Mus musculus 15 days embryo brain cDNA, RIKEN full-length enriched library, clone:G630007M13 product:protein that interacts with C kinase 1, full insert sequence. [AK090155]	18693	AK090155	protein interacting with C kinase 1		Mm.259464
A_51_P300867	0.037	down	-1.4778166	Map3k9	Mus musculus mitogen-activated protein kinase kinase kinase 9 (Map3k9), transcript variant 2, mRNA [NM_177395]	338372	NM_177395	mitogen-activated protein kinase kinase kinase 9	NM_177395	Mm.35284
A_52_P584293	0.004	down	-1.4790355	Atrnl1	Mus musculus attractin like 1 (Atrnl1), mRNA [NM_181415]	226255	NM_181415	attractin like 1	NM_181415	Mm.245340
A_55_P2003004	0.013	down	-1.479551	Capza1	Mus musculus capping protein (actin filament) muscle Z-line, alpha 1 (Capza1), mRNA [NM_009797]	12340	NM_009797	capping protein (actin filament) muscle Z-line, alpha 1	NM_009797	Mm.19142
A_51_P216605	0.010	down	-1.4817821	Hbp1	Mus musculus high mobility group box transcription factor 1 (Hbp1), transcript variant 2, mRNA [NM_177993]	73389	NM_177993	high mobility group box transcription factor 1	NM_177993	Mm.390461
A_51_P446469	0.003	down	-1.4854897	Dok2	Mus musculus docking protein 2 (Dok2), mRNA [NM_010071]	13449	NM_010071	docking protein 2	NM_010071	Mm.243323
A_52_P625912	0.012	down	-1.4855278	Klrb1f	Mus musculus killer cell lectin-like receptor subfamily B member 1F (Klrb1f), transcript variant 1, mRNA [NM_153094]	232408	NM_153094	killer cell lectin-like receptor subfamily B member 1F	NM_153094	Mm.259262
A_51_P471791	0.043	down	-1.489531	St8sia6	Mus musculus ST8 alpha-N-acetyl-neuraminide alpha-2,8-sialyltransferase 6 (St8sia6), mRNA [NM_145838]	241230	NM_145838	ST8 alpha-N-acetyl-neuraminide alpha-2,8-sialyltransferase 6	NM_145838	Mm.330004
A_65_P17869	0.000	down	-1.4910008	Rpf1	Mus musculus ribosome production factor 1 homolog (S. cerevisiae) (Rpf1), transcript variant 1, mRNA [NM_027332]	70285	NM_027332	ribosome production factor 1 homolog (S. cerevisiae)	NM_027332	Mm.28128
A_51_P382369	0.004	down	-1.4914929	Zfp608	Mus musculus zinc finger protein 608 (Zfp608), mRNA [NM_175751]	269023	NM_175751	zinc finger protein 608	NM_175751	Mm.192984
A_51_P437289	0.020	down	-1.4917359	Smco4	Mus musculus single-pass membrane protein with coiled-coil domains 4 (Smco4), mRNA [NM_133214]	170748	NM_133214	single-pass membrane protein with coiled-coil domains 4	NM_133214	Mm.86572
A_51_P502614	0.047	down	-1.4920377	Dusp6	Mus musculus dual specificity phosphatase 6 (Dusp6), mRNA [NM_026268]	67603	NM_026268	dual specificity phosphatase 6	NM_026268	Mm.1791
A_55_P2116332	0.021	down	-1.4924753	Gfm1	Mus musculus 15 days embryo male testis cDNA, RIKEN full-length enriched library, clone:8030465C11 product:G elongation factor, full insert sequence. [AK033214]	28030	AK033214	G elongation factor, mitochondrial 1		
A_51_P185175	0.005	down	-1.4926925	Fkbp4	Mus musculus FK506 binding protein 4 (Fkbp4), mRNA [NM_010219]	14228	NM_010219	FK506 binding protein 4	NM_010219	Mm.12758
A_52_P95930	0.017	down	-1.4956816	Tbx19	Mus musculus T-box 19 (Tbx19), mRNA [NM_032005]	83993	NM_032005	T-box 19	NM_032005	Mm.190649
A_51_P237834	0.008	down	-1.4963912	Tmcc1	Mus musculus transmembrane and coiled coil domains 1 (Tmcc1), mRNA [NM_177412]	330401	NM_177412	transmembrane and coiled coil domains 1	NM_177412	Mm.425352
A_55_P2077618	0.043	down	-1.4969035	Csgalnact1	Mus musculus chondroitin sulfate N-acetylgalactosaminyltransferase 1 (Csgalnact1), transcript variant 1, mRNA [NM_001252623]	234356	NM_001252623	chondroitin sulfate N-acetylgalactosaminyltransferase 1	NM_001252623	Mm.489712
A_51_P193146	0.028	down	-1.4974586	Ms4a6c	Mus musculus membrane-spanning 4-domains, subfamily A, member 6C (Ms4a6c), transcript variant 1, mRNA [NM_028595]	73656	NM_028595	membrane-spanning 4-domains, subfamily A, member 6C	NM_028595	Mm.440755
A_51_P188271	0.041	down	-1.4977477	Cd248	Mus musculus CD248 antigen, endosialin (Cd248), mRNA [NM_054042]	70445	NM_054042	CD248 antigen, endosialin	NM_054042	Mm.29597
A_52_P617327	0.004	down	-1.4993169	Rcan1	Mus musculus regulator of calcineurin 1 (Rcan1), transcript variant 2, mRNA [NM_019466]	54720	NM_019466	regulator of calcineurin 1	NM_019466	Mm.265744
A_51_P498442	0.041	down	-1.5005128	Slc34a1	Mus musculus solute carrier family 34 (sodium phosphate), member 1 (Slc34a1), mRNA [NM_011392]	20505	NM_011392	solute carrier family 34 (sodium phosphate), member 1	NM_011392	Mm.276355

A_51_P214269	0.045	down	-1.5008422	Epha1	Mus musculus Eph receptor A1 (Epha1), mRNA [NM_023580]	13835	NM_023580	Eph receptor A1	NM_023580	Mm.133330
A_51_P147284	0.019	down	-1.5018356	Slain1	Mus musculus SLAIN motif family, member 1 (Slain1), mRNA [NM_198014]	105439	NM_198014	SLAIN motif family, member 1	NM_198014	Mm.27548
A_66_P105526	0.040	down	-1.504027	Mamld1	Mus musculus mastermind-like domain containing 1 (Mamld1), transcript variant 1, mRNA [NM_001081354]	333639	NM_001081354	mastermind-like domain containing 1	NM_001081354	Mm.371730
A_55_P2093849	0.021	down	-1.504388	Nol4	Mus musculus nucleolar protein 4 (Nol4), transcript variant 2, mRNA [NM_199024]	319211	NM_199024	nucleolar protein 4	NM_199024	Mm.209896
A_52_P423247	0.030	down	-1.5052768	Pde4b	Mus musculus phosphodiesterase 4B, cAMP specific (Pde4b), transcript variant 1, mRNA [NM_019840]	18578	NM_019840	phosphodiesterase 4B, cAMP specific	NM_019840	Mm.20181
A_55_P2113081	0.028	down	-1.5057561	Tmem132a	Mus musculus transmembrane protein 132A (Tmem132a), mRNA [NM_133804]	98170	NM_133804	transmembrane protein 132A	NM_133804	Mm.490254
A_51_P225793	0.017	down	-1.506252	Prr5l	Mus musculus proline rich 5 like (Prr5l), transcript variant 2, mRNA [NM_175181]	72446	NM_175181	proline rich 5 like	NM_175181	Mm.24138
A_51_P460420	0.005	down	-1.508135	Rsf1	Mus musculus remodeling and spacing factor 1 (Rsf1), mRNA [NM_001081267]	233532	NM_001081267	remodeling and spacing factor 1	NM_001081267	Mm.43977
A_51_P482711	0.006	down	-1.5084455	Dhcr24	Mus musculus 24-dehydrocholesterol reductase (Dhcr24), mRNA [NM_053272]	74754	NM_053272	24-dehydrocholesterol reductase	NM_053272	Mm.133370
A_55_P1952251	0.001	down	-1.5089066	Spry2	Mus musculus sprouty homolog 2 (Drosophila) (Spry2), mRNA [NM_011897]	24064	NM_011897	sprouty homolog 2 (Drosophila)	NM_011897	Mm.89982
A_55_P2154450	0.020	down	-1.5093926	Xkr6	Mus musculus X Kell blood group precursor related family member 6 homolog (Xkr6), mRNA [NM_173393]	219149	NM_173393	X Kell blood group precursor related family member 6 homolog	NM_173393	Mm.450437
A_52_P592195	0.018	down	-1.5107868	Zkscan8	Mus musculus zinc finger with KRAB and SCAN domains 8 (Zkscan8), transcript variant 3, mRNA [NM_001251834]	93681	NM_001251834	zinc finger with KRAB and SCAN domains 8	NM_001251834	Mm.489857
A_52_P340073	0.014	down	-1.5116426	Efnb2	Mus musculus ephrin B2 (Efnb2), mRNA [NM_010111]	13642	NM_010111	ephrin B2	NM_010111	Mm.209813
A_55_P2119388	0.037	down	-1.5121777	Zeb2	zinc finger E-box binding homeobox 2 [Source:MGI Symbol;Acc:MGI:1344407] [ENSMUST00000028229]	24136	AJ535783	zinc finger E-box binding homeobox 2		Mm.440702
A_55_P2153907	0.004	down	-1.5128024	Mlxip	Mus musculus MLX interacting protein (Mlxip), transcript variant 1, mRNA [NM_177582]	208104	NM_177582	MLX interacting protein	NM_177582	Mm.83277
A_51_P469285	0.028	down	-1.5134826	Nrp1	Mus musculus neuropilin 1 (Nrp1), mRNA [NM_008737]	18186	NM_008737	neuropilin 1	NM_008737	Mm.271745
A_55_P2019138	0.019	down	-1.5134852	Fmn1	Mus musculus formin 1 (Fmn1), transcript variant 2, mRNA [NM_001043322]	14260	NM_001043322	formin 1	NM_001043322	Mm.4938
A_52_P284821	0.004	down	-1.5141393	Slco4c1	Mus musculus solute carrier organic anion transporter family, member 4C1 (Slco4c1), mRNA [NM_172658]	227394	NM_172658	solute carrier organic anion transporter family, member 4C1	NM_172658	Mm.11662
A_55_P2024530	0.023	down	-1.514867	Siah2	Mus musculus seven in absentia 2 (Siah2), mRNA [NM_009174]	20439	NM_009174	seven in absentia 2	NM_009174	Mm.2847
A_51_P384113	0.015	down	-1.5150241	Acad12	Mus musculus acyl-Coenzyme A dehydrogenase family, member 12 (Acad12), mRNA [NM_178799]	338350	NM_178799	acyl-Coenzyme A dehydrogenase family, member 12	NM_178799	Mm.482918
A_51_P463846	0.019	down	-1.5151551	Gbp7	Mus musculus guanylate binding protein 7 (Gbp7), transcript variant 1, mRNA [NM_145545]	229900	NM_145545	guanylate binding protein 7	NM_145545	Mm.45740
A_55_P2058122	0.002	down	-1.516149	Ccbl2	Mus musculus cysteine conjugate-beta lyase 2 (Ccbl2), mRNA [NM_173763]	229905	NM_173763	cysteine conjugate-beta lyase 2	NM_173763	Mm.289643
A_52_P561205	0.000	down	-1.5164688	Stard4	Mus musculus StAR-related lipid transfer (START) domain containing 4 (Stard4), mRNA [NM_133774]	170459	NM_133774	StAR-related lipid transfer (START) domain containing 4	NM_133774	Mm.127058
A_51_P317031	0.041	down	-1.5177583	Ccdc109b	Mus musculus coiled-coil domain containing 109B (Ccdc109b), mRNA [NM_025779]	66815	NM_025779	coiled-coil domain containing 109B	NM_025779	Mm.31056
A_55_P2035946	0.011	down	-1.5183877	Penk	Mus musculus preproenkephalin (Penk), mRNA [NM_001002927]	18619	NM_001002927	preproenkephalin	NM_001002927	Mm.475097
A_51_P355434	0.017	down	-1.5189803	Trib1	tribbles homolog 1 (Drosophila) [Source:MGI Symbol;Acc:MGI:2443397] [ENSMUST00000118228]	211770	AK041212	tribbles homolog 1 (Drosophila)		Mm.40298
A_55_P2090833	0.037	down	-1.5215826	Macrod2	Mus musculus MACRO domain containing 2 (Macrod2), transcript variant 1, mRNA [NM_001013802]	72899	NM_001013802	MACRO domain containing 2	NM_001013802	Mm.229466
A_55_P2074801	0.047	down	-1.5223825	Ptpn14	Mus musculus protein tyrosine phosphatase, non-receptor type 14 (Ptpn14), mRNA [NM_008976]	19250	NM_008976	protein tyrosine phosphatase, non-receptor type 14	NM_008976	Mm.4498
A_51_P275454	0.017	down	-1.5232669	Trim30a	Mus musculus tripartite motif-containing 30A (Trim30a), mRNA [NM_009099]	20128	NM_009099	tripartite motif-containing 30A	NM_009099	Mm.295578
A_55_P2105239	0.018	down	-1.5239105	Defb47	Mus musculus defensin beta 47 (Defb47), mRNA [NM_001039125]	654465	NM_001039125	defensin beta 47	NM_001039125	Mm.424929
A_51_P211854	0.000	down	-1.5247644	Selp	Mus musculus selectin, platelet (Selp), mRNA [NM_011347]	20344	NM_011347	selectin, platelet	NM_011347	Mm.3337
A_55_P2182867	0.001	down	-1.5252261	Sgcb	Mus musculus sarcoglycan, beta (dystrophin-associated glycoprotein) (Sgcb), mRNA [NM_011890]	24051	NM_011890	sarcoglycan, beta (dystrophin-associated glycoprotein)	NM_011890	Mm.89310
A_52_P18922	0.012	down	-1.5272751	Chid1	Mus musculus chitinase domain containing 1 (Chid1), transcript variant 2, mRNA [NM_026522]	68038	NM_026522	chitinase domain containing 1	NM_026522	Mm.245355
A_51_P497985	0.018	down	-1.5276253	C2	Mus musculus complement component 2 (within H-2S) (C2), mRNA [NM_013484]	12263	NM_013484	complement component 2 (within H-2S)	NM_013484	Mm.283217

A_51_P468464	0.032	down	-1.5281864	Gdpd5	Mus musculus glycerophosphodiester phosphodiesterase domain containing 5 (Gdpd5), mRNA [NM_201352]	233552	NM_201352	glycerophosphodiester phosphodiesterase domain containing 5	NM_201352	Mm.286317
A_51_P232901	0.028	down	-1.5288178	Cnp	Mus musculus 2',3'-cyclic nucleotide 3' phosphodiesterase (Cnp), transcript variant 2, mRNA [NM_009923]	12799	NM_009923	2',3'-cyclic nucleotide 3' phosphodiesterase	NM_009923	Mm.15711
A_55_P1970826	0.050	down	-1.5299151	Adamts5	Mus musculus a disintegrin-like and metallopeptidase (reprolysin type) with thrombospondin type 1 motif, 5 (aggrecanase-2) (Adamts5), mRNA [NM_011782]	23794	NM_011782	a disintegrin-like and metallopeptidase (reprolysin type) with thrombospondin type 1 motif, 5 (aggrecanase-2)	NM_011782	Mm.112933
A_55_P2072872	0.045	down	-1.530012	Lpin2	lipin 2 [Source:MGI Symbol;Acc:MGI:1891341] [ENSMUST00000156570]	64898	AK033662	lipin 2		Mm.227924
A_51_P514961	0.012	down	-1.5312505	Tiparp	Mus musculus TCDD-inducible poly(ADP-ribose) polymerase (Tiparp), mRNA [NM_178892]	99929	NM_178892	TCDD-inducible poly(ADP-ribose) polymerase	NM_178892	Mm.246398
A_55_P2019327	0.038	down	-1.5322471	Per1	Mus musculus 16 days embryo head cDNA, RIKEN full-length enriched library, clone:C130078P18 product:period homolog (Drosophila), full insert sequence. [AK081813]	18626	AK081813	period circadian clock 1		
A_55_P2082806	0.030	down	-1.5328974	Trib1	Mus musculus tribbles homolog 1 (Drosophila) (Trib1), mRNA [NM_144549]	211770	NM_144549	tribbles homolog 1 (Drosophila)	NM_144549	Mm.40298
A_65_P15245	0.026	down	-1.533504	Nrp2	Mus musculus neuropilin 2 (Nrp2), transcript variant 5, mRNA [NM_001077406]	18187	NM_001077406	neuropilin 2	NM_001077406	Mm.266341
A_55_P2238255	0.029	down	-1.5342028	Wdr7	Mus musculus WD repeat domain 7 (Wdr7), mRNA [NM_001014981]	104082	NM_001014981	WD repeat domain 7	NM_001014981	Mm.30850
A_51_P256093	0.025	down	-1.5392332	Map2k6	Mus musculus mitogen-activated protein kinase kinase 6 (Map2k6), mRNA [NM_011943]	26399	NM_011943	mitogen-activated protein kinase kinase 6	NM_011943	Mm.14487
A_55_P2386587	0.004	down	-1.53937	Cep63	Mus musculus centrosomal protein 63 (Cep63), mRNA [NM_001081122]	28135	NM_001081122	centrosomal protein 63	NM_001081122	Mm.38910
A_52_P183071	0.000	down	-1.539377	Rnf146	Mus musculus ring finger protein 146 (Rnf146), transcript variant 1, mRNA [NM_001110197]	68031	NM_001110197	ring finger protein 146	NM_001110197	Mm.475694
A_51_P371331	0.000	down	-1.5407312	Mpp5	Mus musculus membrane protein, palmitoylated 5 (MAGUK p55 subfamily member 5) (Mpp5), mRNA [NM_019579]	56217	NM_019579	membrane protein, palmitoylated 5 (MAGUK p55 subfamily member 5)	NM_019579	Mm.425777
A_51_P416689	0.047	down	-1.544002	Ext1	Mus musculus exostoses (multiple) 1 (Ext1), mRNA [NM_010162]	14042	NM_010162	exostoses (multiple) 1	NM_010162	Mm.309395
A_51_P258359	0.036	down	-1.5441061	Hus1b	Mus musculus Hus1 homolog b (S. pombe) (Hus1b), mRNA [NM_153072]	210554	NM_153072	Hus1 homolog b (S. pombe)	NM_153072	Mm.450571
A_51_P497171	0.005	down	-1.5451217	Ly9	Mus musculus lymphocyte antigen 9 (Ly9), transcript variant 1, mRNA [NM_008534]	17085	NM_008534	lymphocyte antigen 9	NM_008534	Mm.560
A_55_P2005783	0.005	down	-1.5471117	Ifih1	Mus musculus interferon induced with helicase C domain 1 (Ifih1), transcript variant 1, mRNA [NM_027835]	71586	NM_027835	interferon induced with helicase C domain 1	NM_027835	Mm.136224
A_55_P2016119	0.000	down	-1.5473698	Sgcb	Mus musculus sarcoglycan, beta (dystrophin-associated glycoprotein) (Sgcb), mRNA [NM_011890]	24051	NM_011890	sarcoglycan, beta (dystrophin-associated glycoprotein)	NM_011890	Mm.89310
A_55_P2419514	0.034	down	-1.5489807	Ccny	Mus musculus cyclin Y (Ccny), mRNA [NM_026484]	67974	NM_026484	cyclin Y	NM_026484	Mm.376607
A_52_P443334	0.013	down	-1.5511408	Cd8a	Mus musculus CD8 antigen, alpha chain (Cd8a), transcript variant 1, mRNA [NM_001081110]	12525	NM_001081110	CD8 antigen, alpha chain	NM_001081110	Mm.1858
A_52_P459929	0.015	down	-1.5529405	Itga1	Mus musculus integrin alpha 1 (Itga1), mRNA [NM_001033228]	109700	NM_001033228	integrin alpha 1	NM_001033228	Mm.317280
A_51_P270807	0.007	down	-1.5543815	Tnfrsf17	Mus musculus tumor necrosis factor receptor superfamily, member 17 (Tnfrsf17), mRNA [NM_011608]	21935	NM_011608	tumor necrosis factor receptor superfamily, member 17	NM_011608	Mm.12935
A_51_P195215	0.042	down	-1.5556005	Fam149a	Mus musculus family with sequence similarity 149, member A (Fam149a), mRNA [NM_153535]	212326	NM_153535	family with sequence similarity 149, member A	NM_153535	Mm.51259
A_55_P1978735	0.044	down	-1.5556804	Slx4ip	Mus musculus SLX4 interacting protein (Slx4ip), transcript variant 1, mRNA [NM_028834]	74243	NM_028834	SLX4 interacting protein	NM_028834	Mm.330612
A_66_P127224	0.025	down	-1.5584632	Krt10	Mus musculus keratin 10 (Krt10), mRNA [NM_010660]	16661	NM_010660	keratin 10	NM_010660	Mm.22662
A_55_P1974283	0.000	down	-1.5586705	Rbmx	Mus musculus RNA binding motif protein, X chromosome (Rbmx), transcript variant 3, mRNA [NM_001166623]	19655	NM_001166623	RNA binding motif protein, X chromosome	NM_001166623	Mm.28275
A_51_P343350	0.014	down	-1.5595889	Amn	Mus musculus amnionless (Amn), mRNA [NM_033603]	93835	NM_033603	amnionless	NM_033603	Mm.197639
A_55_P2111533	0.050	down	-1.5603602	Itga10	Mus musculus integrin, alpha 10 (Itga10), mRNA [NM_001081053]	213119	NM_001081053	integrin, alpha 10	NM_001081053	Mm.32741
A_52_P334670	0.011	down	-1.5608453	Clock	Mus musculus circadian locomotor output cycles kaput (Clock), mRNA [NM_007715]	12753	NM_007715	circadian locomotor output cycles kaput	NM_007715	Mm.3552
A_55_P2112560	0.009	down	-1.561405	Ern1	endoplasmic reticulum (ER) to nucleus signalling 1 [Source:MGI Symbol;Acc:MGI:1930134] [ENSMUST00000106800]	78943		endoplasmic reticulum (ER) to nucleus signalling 1		
A_55_P1985433	0.023	down	-1.5614765	Nrg1	Mus musculus neuregulin 1 (Nrg1), mRNA [NM_178591]	211323	NM_178591	neuregulin 1	NM_178591	Mm.153432
A_55_P1956862	0.033	down	-1.5623192	Egfr	Mus musculus epidermal growth factor receptor (Egfr), transcript variant 2, mRNA [NM_007912]	13649	NM_007912	epidermal growth factor receptor	NM_007912	Mm.8534
A_51_P351896	0.050	down	-1.5654434	Fam198b	Mus musculus family with sequence similarity 198, member B (Fam198b), mRNA [NM_133187]	68659	NM_133187	family with sequence similarity 198, member B	NM_133187	Mm.460017

A_55_P2260094	0.049	down	-1.5662489	Tcf7l2	Mus musculus transcription factor 7 like 2, T cell specific, HMG box (Tcf7l2), transcript variant 1, mRNA [NM_001142918]	21416	NM_001142918	transcription factor 7 like 2, T cell specific, HMG box	NM_001142918	Mm.139815
A_51_P297968	0.011	down	-1.5665395	Pdia6	Mus musculus protein disulfide isomerase associated 6 (Pdia6), mRNA [NM_027959]	71853	NM_027959	protein disulfide isomerase associated 6	NM_027959	Mm.222825
A_55_P1988368	0.038	down	-1.5718765	Upp1	Mus musculus uridine phosphorylase 1 (Upp1), transcript variant 1, mRNA [NM_009477]	22271	NM_009477	uridine phosphorylase 1	NM_009477	Mm.4610
A_55_P1961429	0.032	down	-1.573495	Vmn1r131	Mus musculus vomeronasal 1 receptor 131 (Vmn1r131), mRNA [NM_001166839]	1E+08	NM_001166839	vomeronasal 1 receptor 131	NM_001166839	Mm.484991
A_55_P2007070	0.030	down	-1.5735354	Vmn1r194	Mus musculus vomeronasal 1 receptor 194 (Vmn1r194), mRNA [NM_001080972]	626299	NM_001080972	vomeronasal 1 receptor 194	NM_001080972	Mm.436604
A_55_P2072141	0.021	down	-1.5744643	Chic1	Mus musculus cysteine-rich hydrophobic domain 1 (Chic1), mRNA [NM_009767]	12212	NM_009767	cysteine-rich hydrophobic domain 1	NM_009767	Mm.42223
A_51_P280446	0.027	down	-1.5748018	Sdf2l1	Mus musculus stromal cell-derived factor 2-like 1 (Sdf2l1), mRNA [NM_022324]	64136	NM_022324	stromal cell-derived factor 2-like 1	NM_022324	Mm.30222
A_52_P417796	0.011	down	-1.5761262	Tmem164	Mus musculus transmembrane protein 164 (Tmem164), transcript variant 3, mRNA [NM_001199357]	209497	NM_001199357	transmembrane protein 164	NM_001199357	Mm.249648
A_52_P70027	0.001	down	-1.5769452	Cox16	cytochrome c oxidase assembly protein 16 [Source:MGI Symbol;Acc:MGI:1913522] [ENSMUST00000110340]	66272	BC100533	cytochrome c oxidase assembly protein 16		Mm.439970
A_51_P138060	0.002	down	-1.5787227	Rnf13	Mus musculus ring finger protein 13 (Rnf13), transcript variant 2, mRNA [NM_011883]	24017	NM_011883	ring finger protein 13	NM_011883	Mm.274360
A_55_P2039110	0.016	down	-1.5796695	ND2	mitochondrially encoded NADH dehydrogenase 2 [Source:MGI Symbol;Acc:MGI:102500] [ENSMUST00000082396]	17717	AK167184	NADH dehydrogenase subunit 2		Mm.473834
A_55_P2095311	0.001	down	-1.5806626	Ly6g	lymphocyte antigen 6 complex, locus G [Source:MGI Symbol;Acc:MGI:109440] [ENSMUST00000023246]	546644	XM_001475753	lymphocyte antigen 6 complex, locus G	XM_001475753	
A_55_P1973578	0.022	down	-1.5823972	Foxh1	Mus musculus forkhead box H1 (Foxh1), mRNA [NM_007989]	14106	NM_007989	forkhead box H1	NM_007989	Mm.42011
A_66_P113708	0.042	down	-1.5846637	Nespos	Mus musculus neuroendocrine secretory protein opposite strand (Nespos), antisense RNA [NR_002846]	56802	NR_002846	neuroendocrine secretory protein opposite strand	NR_002846	Mm.425113
A_66_P122660	0.022	down	-1.5852066	Mrgpra4	Mus musculus MAS-related GPR, member A4 (Mrgpra4), mRNA [NM_153524]	235854	NM_153524	MAS-related GPR, member A4	NM_153524	Mm.207065
A_55_P2040921	0.002	down	-1.5852424	Ccnt1	cyclin T1 [Source:MGI Symbol;Acc:MGI:1328363] [ENSMUST00000169707]	12455	BY763388	cyclin T1		Mm.488962
A_55_P2178044	0.012	down	-1.5885922	Inppl1	Mus musculus inositol polyphosphate phosphatase-like 1 (Inppl1), transcript variant 1, mRNA [NM_010567]	16332	NM_010567	inositol polyphosphate phosphatase-like 1	NM_010567	Mm.476000
A_55_P2058340	0.028	down	-1.5889167	Prdx4	peroxiredoxin 4 [Source:MGI Symbol;Acc:MGI:1859815] [ENSMUST00000123915]	53381	AK165551	peroxiredoxin 4		Mm.247542
A_51_P399472	0.035	down	-1.5890934	Catsperg2	Mus musculus catsper channel auxiliary subunit gamma 2 (Catsperg2), mRNA [NM_029714]	76718	NM_029714	catsper channel auxiliary subunit gamma 2	NM_029714	Mm.69030
A_55_P1989976	0.003	down	-1.5904948	Ly9	Mus musculus lymphocyte antigen 9 (Ly9), transcript variant 1, mRNA [NM_008534]	17085	NM_008534	lymphocyte antigen 9	NM_008534	Mm.560
A_66_P129111	0.008	down	-1.5910653	Nasp	Mus musculus nuclear autoantigenic sperm protein mRNA, complete cds. [AF034610]	50927	AF034610	nuclear autoantigenic sperm protein (histone-binding)		Mm.257181
A_55_P2086143	0.006	down	-1.5913389	Cyp39a1	Mus musculus cytochrome P450, family 39, subfamily a, polypeptide 1 (Cyp39a1), transcript variant 1, mRNA [NM_018887]	56050	NM_018887	cytochrome P450, family 39, subfamily a, polypeptide 1	NM_018887	Mm.376968
A_51_P212308	0.015	down	-1.5927994	Cxadr	Mus musculus coxsackie virus and adenovirus receptor (Cxadr), transcript variant 2, mRNA [NM_009988]	13052	NM_009988	coxsackie virus and adenovirus receptor	NM_009988	Mm.66222
A_51_P214251	0.012	down	-1.5934497	Rnls	Mus musculus renalase, FAD-dependent amine oxidase (Rnls), transcript variant 1, mRNA [NM_001167818]	67795	NM_001167818	renalase, FAD-dependent amine oxidase	NM_001167818	Mm.204591
A_55_P2055809	0.002	down	-1.5956409	Rab15	Mus musculus RAB15, member RAS oncogene family (Rab15), mRNA [NM_134050]	104886	NM_134050	RAB15, member RAS oncogene family	NM_134050	Mm.172847
A_52_P244637	0.005	down	-1.599071	Fam65b	Mus musculus family with sequence similarity 65, member B (Fam65b), transcript variant 2, mRNA [NM_001080381]	193385	NM_001080381	family with sequence similarity 65, member B	NM_001080381	Mm.217319
A_52_P282741	0.047	down	-1.6001363	Sdc3	Mus musculus syndecan 3 (Sdc3), mRNA [NM_011520]	20970	NM_011520	syndecan 3	NM_011520	Mm.206536
A_55_P1979412	0.001	down	-1.6010518	Olfr615	Mus musculus olfactory receptor 615 (Olfr615), mRNA [NM_147080]	259084	NM_147080	olfactory receptor 615	NM_147080	Mm.377829
A_66_P108770	0.000	down	-1.6028091	Oxct1	Mus musculus 3-oxoacid CoA transferase 1 (Oxct1), mRNA [NM_024188]	67041	NM_024188	3-oxoacid CoA transferase 1	NM_024188	Mm.13445
A_55_P2028734	0.016	down	-1.6041441	Klra16	Mus musculus killer cell lectin-like receptor, subfamily A, member 16 (Klra16), mRNA [NM_013794]	27424	NM_013794	killer cell lectin-like receptor, subfamily A, member 16	NM_013794	Mm.431309
A_55_P2023294	0.009	down	-1.6062374	Il20rb	Mus musculus interleukin 20 receptor beta (Il20rb), mRNA [NM_001033543]	213208	NM_001033543	interleukin 20 receptor beta	NM_001033543	Mm.242896
A_52_P23379	0.033	down	-1.60702	Depdc7	Mus musculus DEP domain containing 7 (Depdc7), mRNA [NM_144804]	211896	NM_144804	DEP domain containing 7	NM_144804	Mm.288805
A_55_P2097913	0.026	down	-1.6079146	Itga10	Mus musculus integrin, alpha 10 (Itga10), mRNA [NM_001081053]	213119	NM_001081053	integrin, alpha 10	NM_001081053	Mm.32741
A_51_P521052	0.044	down	-1.6089573	Ly6k	Mus musculus lymphocyte antigen 6 complex, locus K (Ly6k), mRNA [NM_029627]	76486	NM_029627	lymphocyte antigen 6 complex, locus K	NM_029627	Mm.273319
A_55_P2090025	0.046	down	-1.6115328	Mest	Mus musculus mesoderm specific transcript (Mest), transcript variant 1, mRNA [NM_001252292]	17294	NM_001252292	mesoderm specific transcript	NM_001252292	Mm.335639

A_55_P2085905	0.007	down	-1.6117799	Anpep	Mus musculus alanyl (membrane) aminopeptidase (Anpep), mRNA [NM_008486]	16790	NM_008486	alanyl (membrane) aminopeptidase	NM_008486	Mm.4487
A_55_P2063033	0.047	down	-1.6120396	Phc2	polyhomeotic-like 2 (Drosophila) [Source:MGI Symbol;Acc:MGI:1860454] [ENSMUST00000133439]	54383		polyhomeotic-like 2 (Drosophila)		
A_55_P2181678	0.001	down	-1.6124364	Dennd1b	DENN/MADD domain containing 1B [Source:MGI Symbol;Acc:MGI:2447812] [ENSMUST00000070283]	329260	BC042698	DENN/MADD domain containing 1B		Mm.337604
A_55_P2038752	0.001	down	-1.6125106	Olfr18	Mus musculus olfactory receptor 18 (Olfr18), mRNA [NM_146563]	18315	NM_146563	olfactory receptor 18	NM_146563	Mm.246524
A_51_P379807	0.000	down	-1.6126904	Cyp4f41-ps	Mus musculus cytochrome P450, family 4, subfamily f, polypeptide 41 pseudogene (Cyp4f41-ps), non-coding RNA [NR_033585]	77875	NR_033585	cytochrome P450, family 4, subfamily f, polypeptide 41 pseudogene	NR_033585	Mm.245027
A_55_P1975560	0.011	down	-1.6130244	Ifi204	Mus musculus interferon activated gene 204 (Ifi204), mRNA [NM_008329]	15951	NM_008329	interferon activated gene 204	NM_008329	Mm.261270
A_55_P2119628	0.011	down	-1.6136613	Gnal	Mus musculus guanine nucleotide binding protein, alpha stimulating, olfactory type (Gnal), transcript variant 2, mRNA [NM_177137]	14680	NM_177137	guanine nucleotide binding protein, alpha stimulating, olfactory type	NM_177137	Mm.440746
A_55_P2141479	0.017	down	-1.6167618	Rnu2-10	Mus musculus U2 small nuclear RNA 10 (Rnu2-10), small nuclear RNA [NR_004414]	19848	NR_004414	U2 small nuclear RNA 10	NR_004414	Mm.41449
A_52_P223809	0.012	down	-1.6178868	Dhx58	Mus musculus DEXH (Asp-Glu-X-His) box polypeptide 58 (Dhx58), mRNA [NM_030150]	80861	NM_030150	DEXH (Asp-Glu-X-His) box polypeptide 58	NM_030150	Mm.271830
A_51_P371588	0.041	down	-1.6179796	Olfr1121	Mus musculus olfactory receptor 1121 (Olfr1121), mRNA [NM_146348]	258345	NM_146348	olfactory receptor 1121	NM_146348	Mm.377444
A_55_P2142858	0.002	down	-1.6199783	Parp9	poly (ADP-ribose) polymerase family, member 9 [Source:MGI Symbol;Acc:MGI:1933117] [ENSMUST00000114877]	80285	AK037903	poly (ADP-ribose) polymerase family, member 9		
A_55_P2004031	0.005	down	-1.6213982	Ctrc	Mus musculus chymotrypsin C (caldecrin) (Ctrc), mRNA [NM_001033875]	76701	NM_001033875	chymotrypsin C (caldecrin)	NM_001033875	Mm.489680
A_66_P129976	0.024	down	-1.6218919	Apba1	Mus musculus amyloid beta (A4) precursor protein binding, family A, member 1 (Apba1), mRNA [NM_177034]	319924	NM_177034	amyloid beta (A4) precursor protein binding, family A, member 1	NM_177034	Mm.22879
A_51_P355301	0.050	down	-1.6228426	Cyp3a11	Mus musculus cytochrome P450, family 3, subfamily a, polypeptide 11 (Cyp3a11), mRNA [NM_007818]	13112	NM_007818	cytochrome P450, family 3, subfamily a, polypeptide 11	NM_007818	Mm.332844
A_52_P591166	0.002	down	-1.6230108	Dpysl2	Mus musculus dihydropyrimidinase-like 2 (Dpysl2), mRNA [NM_009955]	12934	NM_009955	dihydropyrimidinase-like 2	NM_009955	Mm.475100
A_55_P2147121	0.001	down	-1.6231387	Ercc5	Mus musculus adult male hypothalamus cDNA, RIKEN full-length enriched library, clone:A230065O19 product:excision repair cross-complementing rodent repair deficiency,complementation group 5, full insert sequence. [AK038822]	22592	AK038822	excision repair cross-complementing rodent repair deficiency, complementation group 5		Mm.2213
A_55_P2167999	0.032	down	-1.6270537	Ldlr	Mus musculus low density lipoprotein receptor (Ldlr), transcript variant 1, mRNA [NM_010700]	16835	NM_010700	low density lipoprotein receptor	NM_010700	Mm.3213
A_51_P128667	0.025	down	-1.6281112	Lynx1	Mus musculus Ly6/neurotoxin 1 (Lynx1), mRNA [NM_011838]	23936	NM_011838	Ly6/neurotoxin 1	NM_011838	Mm.257067
A_55_P2005585	0.026	down	-1.6314385	Trps1	Mus musculus trichorhinophalangeal syndrome I (human) (Trps1), mRNA [NM_032000]	83925	NM_032000	trichorhinophalangeal syndrome I (human)	NM_032000	Mm.30466
A_65_P16750	0.016	down	-1.6325012	Psma8	Mus musculus proteasome (prosome, macropain) subunit, alpha type, 8 (Psma8), mRNA [NM_001163609]	73677	NM_001163609	proteasome (prosome, macropain) subunit, alpha type, 8	NM_001163609	Mm.87277
A_51_P461665	0.018	down	-1.633696	Cxcl9	Mus musculus chemokine (C-X-C motif) ligand 9 (Cxcl9), mRNA [NM_008599]	17329	NM_008599	chemokine (C-X-C motif) ligand 9	NM_008599	Mm.766
A_66_P134501	0.014	down	-1.6344044	Olfm1	Mus musculus olfactomedin 1 (Olfm1), transcript variant 1, mRNA [NM_019498]	56177	NM_019498	olfactomedin 1	NM_019498	Mm.43278
A_51_P207988	0.004	down	-1.6361659	Ptger4	Mus musculus prostaglandin E receptor 4 (subtype EP4) (Ptger4), transcript variant 2, mRNA [NM_008965]	19219	NM_008965	prostaglandin E receptor 4 (subtype EP4)	NM_008965	Mm.18509
A_52_P96159	0.011	down	-1.6372366	Dsg2	Mus musculus desmoglein 2, mRNA (cDNA clone IMAGE:5355674), complete cds. [BC044848]	13511	BC044848	desmoglein 2		Mm.345891
A_52_P473966	0.041	down	-1.6412824	Kdelr3	Mus musculus KDEL (Lys-Asp-Glu-Leu) endoplasmic reticulum protein retention receptor 3 (Kdelr3), mRNA [NM_134090]	105785	NM_134090	KDEL (Lys-Asp-Glu-Leu) endoplasmic reticulum protein retention receptor 3	NM_134090	Mm.298199
A_51_P257934	0.006	down	-1.641312	Tnfsf13b	Mus musculus tumor necrosis factor (ligand) superfamily, member 13b (Tnfsf13b), mRNA [NM_033622]	24099	NM_033622	tumor necrosis factor (ligand) superfamily, member 13b	NM_033622	Mm.28835
A_55_P2170349	0.043	down	-1.6418879	Klra22	Mus musculus killer cell lectin-like receptor subfamily A, member 22 (Klra22), mRNA [NM_053152]	93969	NM_053152	killer cell lectin-like receptor subfamily A, member 22	NM_053152	Mm.333431
A_55_P2071888	0.034	down	-1.6441967	Slc45a1	Mus musculus adult male medulla oblongata cDNA, RIKEN full-length enriched library, clone:6330522A09 product:deleted in neuroblastoma 5, full insert sequence. [AK134677]	242773	AK134677	solute carrier family 45, member 1		Mm.29280
A_55_P1990505	0.035	down	-1.6461345	Masp2	Mus musculus mannan-binding lectin serine peptidase 2 (Masp2), transcript variant 1, mRNA [NM_001003893]	17175	NM_001003893	mannan-binding lectin serine peptidase 2	NM_001003893	Mm.378962
A_51_P128876	0.002	down	-1.652523	Ifitm3	Mus musculus interferon induced transmembrane protein 3 (Ifitm3), mRNA [NM_025378]	66141	NM_025378	interferon induced transmembrane protein 3	NM_025378	Mm.141021
A_55_P2181753	0.017	down	-1.6536663	Bdh1	Mus musculus 3-hydroxybutyrate dehydrogenase, type 1 (Bdh1), transcript variant 2, mRNA [NM_001122683]	71911	NM_001122683	3-hydroxybutyrate dehydrogenase, type 1	NM_001122683	Mm.293470

A_51_P510418	0.001	down	-1.6546342	Aldh1b1	Mus musculus aldehyde dehydrogenase 1 family, member B1 (Aldh1b1), mRNA [NM_028270]	72535	NM_028270	aldehyde dehydrogenase 1 family, member B1	NM_028270	Mm.331583
A_51_P116796	0.020	down	-1.658405	Crygc	Mus musculus crystallin, gamma C (Crygc), transcript variant 1, mRNA [NM_007775]	12966	NM_007775	crystallin, gamma C	NM_007775	Mm.491115
A_55_P2107542	0.042	down	-1.6605468	Pde4b	Mus musculus 9 days embryo whole body cDNA, RIKEN full-length enriched library, clone:D030072C22 product:phosphodiesterase 4B, cAMP specific, full insert sequence. [AK051102]	18578	AK051102	phosphodiesterase 4B, cAMP specific		Mm.406096
A_55_P1982374	0.020	down	-1.661591	Frmd4a	FERM domain containing 4A [Source:MGI Symbol;Acc:MGI:1919850] [ENSMUST00000132710]	209630	AK034706	FERM domain containing 4A		Mm.37932
A_55_P2301312	0.005	down	-1.6628033	Wdr78	Mus musculus WD repeat domain 78 (Wdr78), mRNA [NM_146254]	242584	NM_146254	WD repeat domain 78	NM_146254	Mm.374877
A_55_P2044684	0.026	down	-1.6637483	Rsph1	Mus musculus radial spoke head 1 homolog (Chlamydomonas) (Rsph1), mRNA [NM_025290]	22092	NM_025290	radial spoke head 1 homolog (Chlamydomonas)	NM_025290	Mm.12743
A_55_P2110497	0.012	down	-1.6642548	Ddc	Mus musculus dopa decarboxylase (Ddc), transcript variant 1, mRNA [NM_001190448]	13195	NM_001190448	dopa decarboxylase	NM_001190448	Mm.12906
A_51_P150876	0.038	down	-1.6668277	Lypd3	Mus musculus Ly6/Plaur domain containing 3 (Lypd3), mRNA [NM_133743]	72434	NM_133743	Ly6/Plaur domain containing 3	NM_133743	Mm.281093
A_51_P305052	0.007	down	-1.6698363	Siglecg	Mus musculus sialic acid binding Ig-like lectin G (Siglecg), mRNA [NM_172900]	243958	NM_172900	sialic acid binding Ig-like lectin G	NM_172900	Mm.24444
A_52_P195839	0.007	down	-1.6705474	Ctsc	Mus musculus cathepsin C (Ctsc), mRNA [NM_009982]	13032	NM_009982	cathepsin C	NM_009982	Mm.322945
A_55_P2168383	0.004	down	-1.6747593	Myo18b	Mus musculus myosin XVIIIb (Myo18b), mRNA [NM_028901]	74376	NM_028901	myosin XVIIIb	NM_028901	Mm.441903
A_55_P2108784	0.041	down	-1.6767664	Arhgap22	Mus musculus Rho GTPase activating protein 22 (Arhgap22), mRNA [NM_153800]	239027	NM_153800	Rho GTPase activating protein 22	NM_153800	Mm.318350
A_51_P169693	0.004	down	-1.6768543	Bst2	Mus musculus bone marrow stromal cell antigen 2 (Bst2), mRNA [NM_198095]	69550	NM_198095	bone marrow stromal cell antigen 2	NM_198095	Mm.260325
A_55_P2000172	0.032	down	-1.6791227	Ppp1r16b	Mus musculus protein phosphatase 1, regulatory (inhibitor) subunit 16B (Ppp1r16b), transcript variant 1, mRNA [NM_001159662]	228852	NM_001159662	protein phosphatase 1, regulatory (inhibitor) subunit 16B	NM_001159662	Mm.150540
A_55_P2131020	0.026	down	-1.680552	Nkx1-2	Mus musculus NK1 transcription factor related, locus 2 (Drosophila) (Nkx1-2), mRNA [NM_009123]	20231	NM_009123	NK1 transcription factor related, locus 2 (Drosophila)	NM_009123	Mm.389981
A_52_P526372	0.019	down	-1.680857	Zeb2	Mus musculus zinc finger E-box binding homeobox 2 (Zeb2), transcript variant 2, mRNA [NM_015753]	24136	NM_015753	zinc finger E-box binding homeobox 2	NM_015753	Mm.440702
A_55_P1955483	0.026	down	-1.6836827	Grb14	Mus musculus growth factor receptor bound protein 14 (Grb14), mRNA [NM_016719]	50915	NM_016719	growth factor receptor bound protein 14	NM_016719	Mm.214554
A_55_P1983733	0.002	down	-1.6839554	Aldh1l1	Mus musculus aldehyde dehydrogenase 1 family, member L1 (Aldh1l1), mRNA [NM_027406]	107747	NM_027406	aldehyde dehydrogenase 1 family, member L1	NM_027406	Mm.30035
A_55_P2109439	0.021	down	-1.6839974	Lsmem1	Mus musculus leucine-rich single-pass membrane protein 1 (Lsmem1), mRNA [NM_001033437]	380755	NM_001033437	leucine-rich single-pass membrane protein 1	NM_001033437	Mm.390463
A_52_P155494	0.048	down	-1.6852139	Zfp317	Mus musculus zinc finger protein 317 (Zfp317), mRNA [NM_172918]	244713	NM_172918	zinc finger protein 317	NM_172918	Mm.67366
A_51_P465292	0.001	down	-1.69006	Hnmt	Mus musculus histamine N-methyltransferase (Hnmt), mRNA [NM_080462]	140483	NM_080462	histamine N-methyltransferase	NM_080462	Mm.33120
A_55_P2043509	0.002	down	-1.6920141	Hnmt	Mus musculus histamine N-methyltransferase (Hnmt), mRNA [NM_080462]	140483	NM_080462	histamine N-methyltransferase	NM_080462	Mm.33120
A_55_P1978830	0.003	down	-1.6920464	Camk2d	Mus musculus calcium/calmodulin-dependent protein kinase II, delta (Camk2d), transcript variant 1, mRNA [NM_001025439]	108058	NM_001025439	calcium/calmodulin-dependent protein kinase II, delta	NM_001025439	Mm.255822
A_51_P444290	0.003	down	-1.6943259	Slamf8	Mus musculus SLAM family member 8 (Slamf8), mRNA [NM_029084]	74748	NM_029084	SLAM family member 8	NM_029084	Mm.179812
A_55_P2123502	0.026	down	-1.6959215	Jam2	Mus musculus junction adhesion molecule 2 (Jam2), mRNA [NM_023844]	67374	NM_023844	junction adhesion molecule 2	NM_023844	Mm.41758
A_55_P2002205	0.027	down	-1.6987284	Bre	brain and reproductive organ-expressed protein [Source:MGI Symbol;Acc:MGI:1333875] [ENSMUST00000114514]	107976	AK050695	brain and reproductive organ-expressed protein		Mm.482126
A_51_P163106	0.024	down	-1.7000586	Bdh1	Mus musculus 3-hydroxybutyrate dehydrogenase, type 1 (Bdh1), transcript variant 1, mRNA [NM_175177]	71911	NM_175177	3-hydroxybutyrate dehydrogenase, type 1	NM_175177	Mm.293470
A_51_P214985	0.026	down	-1.7011906	Zfp521	Mus musculus zinc finger protein 521 (Zfp521), mRNA [NM_145492]	225207	NM_145492	zinc finger protein 521	NM_145492	Mm.40325
A_55_P2181191	0.002	down	-1.7022249	Btg1	Mus musculus B cell translocation gene 1, anti-proliferative (Btg1), mRNA [NM_007569]	12226	NM_007569	B cell translocation gene 1, anti-proliferative	NM_007569	Mm.23811
A_51_P332003	0.040	down	-1.7047609	Aste1	Mus musculus asteroid homolog 1 (Drosophila) (Aste1), transcript variant 1, mRNA [NM_025651]	66595	NM_025651	asteroid homolog 1 (Drosophila)	NM_025651	Mm.84941
A_55_P2135986	0.019	down	-1.7058157	Ms4a4c	Mus musculus membrane-spanning 4-domains, subfamily A, member 4C (Ms4a4c), mRNA [NM_029499]	64380	NM_029499	membrane-spanning 4-domains, subfamily A, member 4C	NM_029499	Mm.440715
A_55_P2110136	0.014	down	-1.7059119	Arhgap29	Mus musculus Rho GTPase activating protein 29 (Arhgap29), mRNA [NM_172525]	214137	NM_172525	Rho GTPase activating protein 29	NM_172525	Mm.229287
A_55_P2268221	0.006	down	-1.7067292	Arhgap10	Mus musculus Rho GTPase activating protein 10 (Arhgap10), mRNA [NM_030113]	78514	NM_030113	Rho GTPase activating protein 10	NM_030113	Mm.165974

A_55_P2136827	0.031	down	-1.7145483	Wfdc16	Mus musculus WAP four-disulfide core domain 16 (Wfdc16), mRNA [NM_001012723]	277345	NM_001012723	WAP four-disulfide core domain 16	NM_001012723	Mm.247244
A_51_P153734	0.003	down	-1.719542	Kif23	Mus musculus 13 days embryo head cDNA, RIKEN full-length enriched library, clone:3110001D19 product:kinesin superfamily protein 23, full insert sequence. [AK013939]	71819	AK013939	kinesin family member 23		
A_55_P2014626	0.026	down	-1.7218918	Tcl1b4	Mus musculus T cell leukemia/lymphoma 1B, 4 (Tcl1b4), mRNA [NM_013774]	27380	NM_013774	T cell leukemia/lymphoma 1B, 4	NM_013774	Mm.103652
A_55_P2046812	0.048	down	-1.7286568	Sparc	Mus musculus secreted acidic cysteine rich glycoprotein (Sparc), mRNA [NM_009242]	20692	NM_009242	secreted acidic cysteine rich glycoprotein	NM_009242	Mm.291442
A_51_P124535	0.043	down	-1.7290596	Mest	Mus musculus mesoderm specific transcript (Mest), transcript variant 2, mRNA [NM_008590]	17294	NM_008590	mesoderm specific transcript	NM_008590	Mm.335639
A_55_P2003638	0.044	down	-1.7291865	Stxbp6	Mus musculus syntaxin binding protein 6 (amisyn) (Stxbp6), mRNA [NM_144552]	217517	NM_144552	syntaxin binding protein 6 (amisyn)	NM_144552	Mm.285400
A_55_P2112642	0.037	down	-1.7300104	Tshr	Mus musculus thyroid stimulating hormone receptor (Tshr), transcript variant 1, mRNA [NM_011648]	22095	NM_011648	thyroid stimulating hormone receptor	NM_011648	Mm.173847
A_52_P227937	0.039	down	-1.7345394	Cystm1	cysteine-rich transmembrane module containing 1 [Source:MGI Symbol;Acc:MGI:1913310] [ENSMUST00000050584]	66060	BC028765	cysteine-rich transmembrane module containing 1		Mm.272527
A_52_P54770	0.006	down	-1.7375482	Fam19a4	Mus musculus family with sequence similarity 19, member A4 (Fam19a4), mRNA [NM_177233]	320701	NM_177233	family with sequence similarity 19, member A4	NM_177233	Mm.131700
A_55_P2117818	0.045	down	-1.7413367	Pigu	Mus musculus adult male spinal cord cDNA, RIKEN full-length enriched library, clone:A330029E17 product:PIG-U homolog [Rattus norvegicus], full insert sequence. [AK162913]	228812	AK162913	phosphatidylinositol glycan anchor biosynthesis, class U		Mm.5434
A_52_P378968	0.019	down	-1.7414403	Rgs2	Mus musculus regulator of G-protein signaling 2 (Rgs2), mRNA [NM_009061]	19735	NM_009061	regulator of G-protein signaling 2	NM_009061	Mm.28262
A_51_P413147	0.030	down	-1.7429945	Klk1b3	Mus musculus kallikrein 1-related peptidase b3 (Klk1b3), mRNA [NM_008693]	18050	NM_008693	kallikrein 1-related peptidase b3	NM_008693	Mm.439740
A_55_P2046149	0.008	down	-1.74314	Kremen1	Mus musculus kringle containing transmembrane protein 1 (Kremen1), mRNA [NM_032396]	84035	NM_032396	kringle containing transmembrane protein 1	NM_032396	Mm.209989
A_55_P1959496	0.001	down	-1.7431732	Sbno2	Mus musculus strawberry notch homolog 2 (Drosophila), mRNA (cDNA clone IMAGE:3376209), complete cds. [BC064113]	216161	BC064113	strawberry notch homolog 2 (Drosophila)		Mm.262102
A_55_P2293013	0.012	down	-1.744387	Ces2a	Mus musculus carboxylesterase 2A (Ces2a), transcript variant 1, mRNA [NM_133960]	102022	NM_133960	carboxylesterase 2A	NM_133960	Mm.212983
A_55_P2124153	0.039	down	-1.746984	Rusc2	Mus musculus RUN and SH3 domain containing 2 (Rusc2), transcript variant 2, mRNA [NM_001037709]	100213	NM_001037709	RUN and SH3 domain containing 2	NM_001037709	Mm.235730
A_55_P2032649	0.021	down	-1.7477143	Kif5b	kinesin family member 5B [Source:MGI Symbol;Acc:MGI:1098268] [ENSMUST00000170959]	16573	CJ113196	kinesin family member 5B		Mm.458279
A_55_P2004781	0.002	down	-1.7479306	Ass1	Mus musculus argininosuccinate synthetase 1 (Ass1), mRNA [NM_007494]	11898	NM_007494	argininosuccinate synthetase 1	NM_007494	Mm.3217
A_55_P2144280	0.008	down	-1.7496347	Nnt	Mus musculus nicotinamide nucleotide transhydrogenase (Nnt), transcript variant 1, mRNA [NM_008710]	18115	NM_008710	nicotinamide nucleotide transhydrogenase	NM_008710	Mm.132584
A_51_P352411	0.008	down	-1.752605	Nudcd1	Mus musculus NudC domain containing 1 (Nudcd1), transcript variant 1, mRNA [NM_026149]	67429	NM_026149	NudC domain containing 1	NM_026149	Mm.490404
A_55_P2015687	0.022	down	-1.7526883	Phf11d	Mus musculus PHD finger protein 11D (Phf11d), mRNA [NM_199015]	219132	NM_199015	PHD finger protein 11D	NM_199015	Mm.479285
A_51_P230904	0.004	down	-1.753877	Copz2	Mus musculus coatomer protein complex, subunit zeta 2 (Copz2), mRNA [NM_019877]	56358	NM_019877	coatomer protein complex, subunit zeta 2	NM_019877	Mm.22144
A_52_P651078	0.003	down	-1.7575346	Fyttd1	Mus musculus forty-two-three domain containing 1 (Fyttd1), transcript variant 1, mRNA [NM_027226]	69823	NM_027226	forty-two-three domain containing 1	NM_027226	Mm.12831
A_52_P582374	0.002	down	-1.7581887	Epsti1	Mus musculus epithelial stromal interaction 1 (breast) (Epsti1), transcript variant b, mRNA [NM_178825]	108670	NM_178825	epithelial stromal interaction 1 (breast)	NM_178825	Mm.68134
A_55_P2288117	0.000	down	-1.7584894	Ppp2ca	protein phosphatase 2 (formerly 2A), catalytic subunit, alpha isoform [Source:MGI Symbol;Acc:MGI:1321159] [ENSMUST00000020608]	19052	AK035193	protein phosphatase 2 (formerly 2A), catalytic subunit, alpha isoform		Mm.40874
A_55_P2139019	0.044	down	-1.762433	Hivep3	human immunodeficiency virus type I enhancer binding protein 3 [Source:MGI Symbol;Acc:MGI:106589] [ENSMUST00000123698]	16656	AK013707	human immunodeficiency virus type I enhancer binding protein 3		Mm.302758
A_52_P188261	0.005	down	-1.7637984	Camk2d	Mus musculus calcium/calmodulin-dependent protein kinase II, delta (Camk2d), transcript variant 1, mRNA [NM_001025439]	108058	NM_001025439	calcium/calmodulin-dependent protein kinase II, delta	NM_001025439	Mm.255822
A_52_P428745	0.019	down	-1.765899	Camk2d	Mus musculus calcium/calmodulin-dependent protein kinase II, delta (Camk2d), transcript variant 1, mRNA [NM_001025439]	108058	NM_001025439	calcium/calmodulin-dependent protein kinase II, delta	NM_001025439	Mm.255822
A_55_P1963975	0.003	down	-1.76697	Lrrc16a	Mus musculus leucine rich repeat containing 16A (Lrrc16a), mRNA [NM_026825]	68732	NM_026825	leucine rich repeat containing 16A	NM_026825	Mm.211047
A_51_P290576	0.020	down	-1.7685273	Plk2	Mus musculus polo-like kinase 2 (Plk2), mRNA [NM_152804]	20620	NM_152804	polo-like kinase 2	NM_152804	Mm.380
A_55_P2078710	0.024	down	-1.7690772	Morc4	Mus musculus microrchidia 4 (Morc4), transcript variant B, mRNA [NM_029413]	75746	NM_029413	microrchidia 4	NM_029413	Mm.23269
A_55_P2142072	0.002	down	-1.7727448	Synj2	Mus musculus synaptojanin 2 (Synj2), transcript variant 3, mRNA [NM_011523]	20975	NM_011523	synaptojanin 2	NM_011523	Mm.236068

A_55_P2097808	0.006	down	-1.7753147	P4ha1	Mus musculus procollagen-proline, 2-oxoglutarate 4-dioxygenase (proline 4-hydroxylase), alpha 1 polypeptide (P4ha1), mRNA [NM_011030]	18451	NM_011030	procollagen-proline, 2-oxoglutarate 4-dioxygenase (proline 4-hydroxylase), alpha 1 polypeptide	NM_011030	Mm.2212
A_55_P2033308	0.027	down	-1.7777194	Six2	Mus musculus sine oculis-related homeobox 2 (Six2), mRNA [NM_011380]	20472	NM_011380	sine oculis-related homeobox 2	NM_011380	Mm.5039
A_51_P514256	0.008	down	-1.7778567	Tubb2b	Mus musculus tubulin, beta 2B class IIB (Tubb2b), mRNA [NM_023716]	73710	NM_023716	tubulin, beta 2B class IIB	NM_023716	Mm.379227
A_51_P389396	0.047	down	-1.7784891	Gykl1	Mus musculus glycerol kinase-like 1 (Gykl1), mRNA [NM_010293]	14625	NM_010293	glycerol kinase-like 1	NM_010293	Mm.32242
A_55_P2018697	0.036	down	-1.7796061	Ldhd	Mus musculus lactate dehydrogenase D (Ldhd), mRNA [NM_027570]	52815	NM_027570	lactate dehydrogenase D	NM_027570	Mm.27589
A_51_P464703	0.038	down	-1.7801982	Ccl8	Mus musculus chemokine (C-C motif) ligand 8 (Ccl8), mRNA [NM_021443]	20307	NM_021443	chemokine (C-C motif) ligand 8	NM_021443	Mm.42029
A_55_P1961099	0.000	down	-1.7834481	Atg4c	Mus musculus autophagy related 4C, cysteine peptidase (Atg4c), transcript variant 1, mRNA [NM_175029]	242557	NM_175029	autophagy related 4C, cysteine peptidase	NM_175029	Mm.241663
A_55_P2159585	0.026	down	-1.7846427	Pm20d1	Mus musculus peptidase M20 domain containing 1 (Pm20d1), mRNA [NM_178079]	212933	NM_178079	peptidase M20 domain containing 1	NM_178079	Mm.23844
A_66_P116226	0.006	down	-1.7864703	Tpgs2	Mus musculus tubulin polyglutamylase complex subunit 2 (Tpgs2), transcript variant 1, mRNA [NM_001004361]	66648	NM_001004361	tubulin polyglutamylase complex subunit 2	NM_001004361	Mm.263189
A_55_P2026572	0.028	down	-1.7885219	Fam189a1	Mus musculus family with sequence similarity 189, member A1 (Fam189a1), mRNA [NM_183087]	70638	NM_183087	family with sequence similarity 189, member A1	NM_183087	Mm.441986
A_55_P2406506	0.006	down	-1.7934092	Tuba-rs1	Mouse testicular alpha tubulin mRNA, 3' end. [M19413]	1E+08	M19413	tubulin alpha, related sequence 1		Mm.439689
A_51_P284946	0.011	down	-1.7936845	Rnd3	Mus musculus Rho family GTPase 3 (Rnd3), mRNA [NM_028810]	74194	NM_028810	Rho family GTPase 3	NM_028810	Mm.46497
A_55_P1980730	0.021	down	-1.800656	Plcb1	Mus musculus phospholipase C, beta 1 (Plcb1), transcript variant 1, mRNA [NM_001145830]	18795	NM_001145830	phospholipase C, beta 1	NM_001145830	Mm.330607
A_55_P2106033	0.002	down	-1.8045857	Myo18b	Mus musculus myosin XVIIIb (Myo18b), mRNA [NM_028901]	74376	NM_028901	myosin XVIIIb	NM_028901	Mm.441903
A_52_P28651	0.006	down	-1.8055729	Pvrl1	Mus musculus poliovirus receptor-related 1 (Pvrl1), mRNA [NM_021424]	58235	NM_021424	poliovirus receptor-related 1	NM_021424	Mm.335096
A_55_P2079483	0.028	down	-1.807734	Gbx1	Mus musculus gastrulation brain homeobox 1 (Gbx1), mRNA [NM_015739]	231044	NM_015739	gastrulation brain homeobox 1	NM_015739	Mm.246596
A_55_P2014319	0.008	down	-1.8148062	Armcx6	Mus musculus armadillo repeat containing, X-linked 6 (Armcx6), mRNA [NM_001007578]	278097	NM_001007578	armadillo repeat containing, X-linked 6	NM_001007578	Mm.113272
A_55_P1958140	0.023	down	-1.8201989	B9d1	Mus musculus B9 protein domain 1 (B9d1), mRNA [NM_013717]	27078	NM_013717	B9 protein domain 1	NM_013717	Mm.261226
A_55_P1973046	0.031	down	-1.8214852	Cttnbp2nl	Mus musculus CTTNBP2 N-terminal like (Cttnbp2nl), transcript variant 1, mRNA [NM_030249]	80281	NM_030249	CTTNBP2 N-terminal like	NM_030249	Mm.200327
A_52_P352187	0.041	down	-1.8228921	Acsl6	Mus musculus acyl-CoA synthetase long-chain family member 6 (Acsl6), transcript variant 4, mRNA [NM_001033599]	216739	NM_001033599	acyl-CoA synthetase long-chain family member 6	NM_001033599	Mm.267478
A_51_P262515	0.022	down	-1.8267266	Phf11a	Mus musculus PHD finger protein 11A (Phf11a), mRNA [NM_172603]	219131	NM_172603	PHD finger protein 11A	NM_172603	Mm.254918
A_55_P2159480	0.046	down	-1.8267748	Fam160a1	Mus musculus family with sequence similarity 160, member A1 (Fam160a1), mRNA [NM_172682]	229488	NM_172682	family with sequence similarity 160, member A1	NM_172682	Mm.119926
A_66_P100420	0.012	down	-1.8271178	Olfr1000	Mus musculus olfactory receptor 1000 (Olfr1000), mRNA [NM_001011695]	257899	NM_001011695	olfactory receptor 1000	NM_001011695	Mm.376112
A_55_P1999962	0.023	down	-1.8275826	Podn	Mus musculus podocan (Podn), transcript variant 1, mRNA [NM_001285956]	242608	NM_001285956	podocan	NM_001285956	Mm.74710
A_52_P61520	0.009	down	-1.8297795	Nsun3	Mus musculus NOL1/NOP2/Sun domain family member 3 (Nsun3), mRNA [NM_178925]	106338	NM_178925	NOL1/NOP2/Sun domain family member 3	NM_178925	Mm.26321
A_55_P1975215	0.011	down	-1.8333117	Npas4	Mus musculus neuronal PAS domain protein 4 (Npas4), mRNA [NM_153553]	225872	NM_153553	neuronal PAS domain protein 4	NM_153553	Mm.287867
A_55_P2126950	0.001	down	-1.8407525	Zfp467	Mus musculus zinc finger protein 467 (Zfp467), transcript variant 4, mRNA [NM_001085417]	68910	NM_001085417	zinc finger protein 467	NM_001085417	Mm.2464
A_52_P679101	0.028	down	-1.8476483	Tjp2	Mus musculus tight junction protein 2 (Tjp2), transcript variant 2, mRNA [NM_011597]	21873	NM_011597	tight junction protein 2	NM_011597	Mm.104744
A_55_P2018061	0.029	down	-1.8517597	Cd209a	Mus musculus CD209a antigen (Cd209a), mRNA [NM_133238]	170786	NM_133238	CD209a antigen	NM_133238	Mm.32510
A_52_P384036	0.038	down	-1.8524373	Agbl2	ATP/GTP binding protein-like 2 [Source:MGI Symbol;Acc:MGI:2443254] [ENSMUST00000051831]	271813	AK029744	ATP/GTP binding protein-like 2		Mm.61146
A_55_P2181222	0.007	down	-1.8526036	Pydc3	Mus musculus pyrin domain containing 3 (Pydc3), mRNA [NM_001162938]	1E+08	NM_001162938	pyrin domain containing 3	NM_001162938	Mm.303377
A_55_P2060922	0.047	down	-1.8584301	Unc5a	Mus musculus unc-5 homolog A (C. elegans) (Unc5a), mRNA [NM_153131]	107448	NM_153131	unc-5 homolog A (C. elegans)	NM_153131	Mm.23573
A_51_P464900	0.029	down	-1.8608685	Gabbr1	Mus musculus gamma-aminobutyric acid (GABA) B receptor, 1 (Gabbr1), mRNA [NM_019439]	54393	NM_019439	gamma-aminobutyric acid (GABA) B receptor, 1	NM_019439	Mm.32191
A_66_P131754	0.002	down	-1.8632529	Vwa5a	Mus musculus von Willebrand factor A domain containing 5A (Vwa5a), transcript variant 1, mRNA [NM_172767]	67776	NM_172767	von Willebrand factor A domain containing 5A	NM_172767	Mm.290950
A_51_P147766	0.010	down	-1.8664906	Cpne9	Mus musculus copine family member IX (Cpne9), mRNA [NM_170673]	211232	NM_170673	copine family member IX	NM_170673	Mm.65597

A_66_P106388	0.011	down	-1.8679156	Ms4a4c	Mus musculus membrane-spanning 4-domains, subfamily A, member 4C (Ms4a4c), mRNA [NM_029499]	64380	NM_029499	membrane-spanning 4-domains, subfamily A, member 4C	NM_029499	Mm.440715
A_55_P2038262	0.005	down	-1.8733755	Phf11b	Mus musculus PHD finger protein 11B (Phf11b), mRNA [NM_001164327]	236451	NM_001164327	PHD finger protein 11B	NM_001164327	Mm.247736
A_52_P145539	0.024	down	-1.876305	Tshr	Mus musculus thyroid stimulating hormone receptor (Tshr), transcript variant 1, mRNA [NM_011648]	22095	NM_011648	thyroid stimulating hormone receptor	NM_011648	Mm.173847
A_55_P2367803	0.050	down	-1.8765488	Il2	Mus musculus interleukin 2 (Il2), mRNA [NM_008366]	16183	NM_008366	interleukin 2	NM_008366	Mm.14190
A_51_P177171	0.000	down	-1.8780655	Tie1	Mus musculus tyrosine kinase with immunoglobulin-like and EGF-like domains 1 (Tie1), mRNA [NM_011587]	21846	NM_011587	tyrosine kinase with immunoglobulin-like and EGF-like domains 1	NM_011587	Mm.4345
A_55_P2122604	0.001	down	-1.8830591	Cbr2	Mus musculus carbonyl reductase 2 (Cbr2), mRNA [NM_007621]	12409	NM_007621	carbonyl reductase 2	NM_007621	Mm.21454
A_55_P1973683	0.018	down	-1.8885651	Morn4	Mus musculus MORN repeat containing 4 (Morn4), mRNA [NM_198108]	226123	NM_198108	MORN repeat containing 4	NM_198108	Mm.259441
A_52_P551909	0.030	down	-1.8936373	Arhgap5	Mus musculus Rho GTPase activating protein 5 (Arhgap5), mRNA [NM_009706]	11855	NM_009706	Rho GTPase activating protein 5	NM_009706	Mm.466962
A_55_P1994613	0.041	down	-1.9000003	Itsn1	Mus musculus intersectin 1 (SH3 domain protein 1A) (Itsn1), transcript variant 1, mRNA [NM_010587]	16443	NM_010587	intersectin 1 (SH3 domain protein 1A)	NM_010587	Mm.40546
A_52_P140049	0.016	down	-1.9012287	Hepacam2	Mus musculus HEPACAM family member 2 (Hepacam2), mRNA [NM_178899]	101202	NM_178899	HEPACAM family member 2	NM_178899	Mm.206911
A_55_P2232754	0.003	down	-1.9054596	Smarcal1	Mus musculus SWI/SNF related matrix associated, actin dependent regulator of chromatin, subfamily a-like 1 (Smarcal1), mRNA [NM_018817]	54380	NM_018817	SWI/SNF related matrix associated, actin dependent regulator of chromatin, subfamily a-like 1	NM_018817	Mm.274232
A_65_P01834	0.008	down	-1.9063679	Lima1	Mus musculus LIM domain and actin binding 1 (Lima1), transcript variant a, mRNA [NM_001113545]	65970	NM_001113545	LIM domain and actin binding 1	NM_001113545	Mm.33207
A_55_P2073338	0.006	down	-1.9065619	Syn1	Mus musculus synapsin I (Syn1), transcript variant a, mRNA [NM_013680]	20964	NM_013680	synapsin I	NM_013680	Mm.439844
A_51_P230287	0.014	down	-1.9071076	Sorcs2	Mus musculus sortilin-related VPS10 domain containing receptor 2 (Sorcs2), mRNA [NM_030889]	81840	NM_030889	sortilin-related VPS10 domain containing receptor 2	NM_030889	Mm.34113
A_55_P2106074	0.001	down	-1.9118569	Wfikkn1	Mus musculus WAP, FS, Ig, KU, and NTR-containing protein 1 (Wfikkn1), mRNA [NM_001100454]	215001	NM_001100454	WAP, FS, Ig, KU, and NTR-containing protein 1	NM_001100454	Mm.328871
A_55_P2143070	0.002	down	-1.9120982	Ass1	Mus musculus argininosuccinate synthetase 1 (Ass1), mRNA [NM_007494]	11898	NM_007494	argininosuccinate synthetase 1	NM_007494	Mm.3217
A_51_P149714	0.031	down	-1.9131628	Ms4a6d	Mus musculus membrane-spanning 4-domains, subfamily A, member 6D (Ms4a6d), mRNA [NM_026835]	68774	NM_026835	membrane-spanning 4-domains, subfamily A, member 6D	NM_026835	Mm.290390
A_51_P295237	0.018	down	-1.9160165	Lrp11	Mus musculus low density lipoprotein receptor-related protein 11 (Lrp11), mRNA [NM_172784]	237253	NM_172784	low density lipoprotein receptor-related protein 11	NM_172784	Mm.206759
A_52_P657360	0.007	down	-1.9218575	Tnni1	Mus musculus troponin I, skeletal, slow 1 (Tnni1), transcript variant 1, mRNA [NM_021467]	21952	NM_021467	troponin I, skeletal, slow 1	NM_021467	Mm.44379
A_55_P1994042	0.005	down	-1.9238223	Zbp1	Mus musculus Z-DNA binding protein 1 (Zbp1), transcript variant 2, mRNA [NM_001139519]	58203	NM_001139519	Z-DNA binding protein 1	NM_001139519	Mm.116687
A_55_P2035509	0.003	down	-1.9247584	Pyhin1	Mus musculus pyrin and HIN domain family, member 1 (Pyhin1), mRNA [NM_175026]	236312	NM_175026	pyrin and HIN domain family, member 1	NM_175026	Mm.447
A_52_P145415	0.011	down	-1.9332137	Ptch2	patched homolog 2 [Source:MGI Symbol;Acc:MGI:1095405] [ENSMUST00000030443]	19207	BC058397	patched homolog 2		Mm.287037
A_52_P265965	0.016	down	-1.9337857	Trim36	Mus musculus tripartite motif-containing 36 (Trim36), transcript variant 1, mRNA [NM_178872]	28105	NM_178872	tripartite motif-containing 36	NM_178872	Mm.277382
A_55_P2004541	0.032	down	-1.9394376	Klra7	Mus musculus killer cell lectin-like receptor, subfamily A, member 7 (Klra7), transcript variant 1, mRNA [NM_001110323]	16638	NM_001110323	killer cell lectin-like receptor, subfamily A, member 7	NM_001110323	Mm.441781
A_51_P366672	0.028	down	-1.9400641	Slc36a2	Mus musculus solute carrier family 36 (proton/amino acid symporter), member 2 (Slc36a2), mRNA [NM_153170]	246049	NM_153170	solute carrier family 36 (proton/amino acid symporter), member 2	NM_153170	Mm.291280
A_55_P2034875	0.003	down	-1.9439089	Siglecg	Mus musculus sialic acid binding Ig-like lectin G (Siglecg), mRNA [NM_172900]	243958	NM_172900	sialic acid binding Ig-like lectin G	NM_172900	Mm.24444
A_55_P2066578	0.002	down	-1.9589124	Ifi204	Mus musculus interferon activated gene 204, mRNA (cDNA clone MGC:18551 IMAGE:4018506), complete cds. [BC010546]	15951	BC010546	interferon activated gene 204		Mm.261270
A_55_P1955172	0.022	down	-1.9679773	Camk2d	Mus musculus 13 days embryo stomach cDNA, RIKEN full-length enriched library, clone:D530006I24 product:calcium/calmodulin-dependent protein kinase II, delta, full insert sequence. [AK142435]	108058	AK142435	calcium/calmodulin-dependent protein kinase II, delta		Mm.255822
A_55_P2275057	0.017	down	-1.968386	Nrap	Mus musculus nebulin-related anchoring protein (Nrap), transcript variant 2, mRNA [NM_008733]	18175	NM_008733	nebulin-related anchoring protein	NM_008733	Mm.386769
A_55_P1958554	0.002	down	-1.9690682	Ar	Mus musculus androgen receptor (Ar), mRNA [NM_013476]	11835	NM_013476	androgen receptor	NM_013476	Mm.439657
A_55_P2060097	0.000	down	-1.9742402	Prdm5	Mus musculus PR domain containing 5 (Prdm5), mRNA [NM_027547]	70779	NM_027547	PR domain containing 5	NM_027547	Mm.263355
A_52_P217796	0.005	down	-1.9798316	Ift46	Mus musculus intraflagellar transport 46 (Ift46), mRNA [NM_023831]	76568	NM_023831	intraflagellar transport 46	NM_023831	Mm.278597

A_55_P2156855	0.004	down	-1.9799567	Car13	Mus musculus carbonic anhydrase 13 (Car13), mRNA [NM_024495]	71934	NM_024495	carbonic anhydrase 13	NM_024495	Mm.158776
A_51_P183051	0.013	down	-1.9817835	Upb1	Mus musculus ureidopropionase, beta (Upb1), mRNA [NM_133995]	103149	NM_133995	ureidopropionase, beta	NM_133995	Mm.441195
A_51_P387123	0.006	down	-1.9831499	Oasl2	Mus musculus 2'-5' oligoadenylate synthetase-like 2 (Oasl2), mRNA [NM_011854]	23962	NM_011854	2'-5' oligoadenylate synthetase-like 2	NM_011854	Mm.228363
A_51_P203277	0.010	down	-1.9853113	Vmn1r66	Mus musculus vomeronasal 1 receptor 66 (Vmn1r66), mRNA [NM_134230]	171264	NM_134230	vomeronasal 1 receptor 66	NM_134230	Mm.222637
A_51_P159402	0.001	down	-1.989697	Gcat	Mus musculus glycine C-acetyltransferase (2-amino-3-ketobutyrate-coenzyme A ligase) (Gcat), transcript variant 1, mRNA [NM_013847]	26912	NM_013847	glycine C-acetyltransferase (2-amino-3-ketobutyrate-coenzyme A ligase)	NM_013847	Mm.237085
A_55_P1964183	0.010	down	-1.9917724	Dysf	Mus musculus dysferlin (Dysf), transcript variant 2, mRNA [NM_001077694]	26903	NM_001077694	dysferlin	NM_001077694	Mm.220982
A_55_P2114118	0.010	down	-1.9919354	Cadm1	Mus musculus cell adhesion molecule 1 (Cadm1), transcript variant 1, mRNA [NM_207675]	54725	NM_207675	cell adhesion molecule 1	NM_207675	Mm.234832
A_51_P343517	0.002	down	-1.9922174	Ly6d	Mus musculus lymphocyte antigen 6 complex, locus D (Ly6d), mRNA [NM_010742]	17068	NM_010742	lymphocyte antigen 6 complex, locus D	NM_010742	Mm.878
A_51_P421876	0.008	down	-1.9952458	Irf7	Mus musculus interferon regulatory factor 7 (Irf7), transcript variant 1, mRNA [NM_016850]	54123	NM_016850	interferon regulatory factor 7	NM_016850	Mm.3233
A_52_P381430	0.026	down	-1.9991957	Tbc1d4	Mus musculus TBC1 domain family, member 4 (Tbc1d4), mRNA [NM_001081278]	210789	NM_001081278	TBC1 domain family, member 4	NM_001081278	Mm.320639
A_51_P187461	0.005	down	-2.004529	Rel	Mus musculus reticuloendotheliosis oncogene (Rel), mRNA [NM_009044]	19696	NM_009044	reticuloendotheliosis oncogene	NM_009044	Mm.4869
A_52_P156158	0.000	down	-2.0085785	Grpel1	Mus musculus GrpE-like 1, mitochondrial (Grpel1), mRNA [NM_024478]	17713	NM_024478	GrpE-like 1, mitochondrial	NM_024478	Mm.21535
A_55_P1966731	0.003	down	-2.0105083	Ifi203	Mus musculus interferon activated gene 203 (Ifi203), transcript variant 1, mRNA [NM_001045481]	15950	NM_001045481	interferon activated gene 203	NM_001045481	Mm.472787
A_55_P1954569	0.001	down	-2.017343	Hmgcs1	Mus musculus 13 days embryo male testis cDNA, RIKEN full-length enriched library, clone:6030403N11 product:pre B-cell leukemia transcription factor 1, full insert sequence. [AK031297]	208715	AK031297	3-hydroxy-3-methylglutaryl-Coenzyme A synthase 1		Mm.61526
A_51_P223092	0.007	down	-2.0193841	Zbed3	Mus musculus zinc finger, BED domain containing 3 (Zbed3), mRNA [NM_028106]	72114	NM_028106	zinc finger, BED domain containing 3	NM_028106	Mm.3774
A_51_P382970	0.009	down	-2.0279748	Itga9	Mus musculus integrin alpha 9 (Itga9), transcript variant 1, mRNA [NM_133721]	104099	NM_133721	integrin alpha 9	NM_133721	Mm.335520
A_66_P120507	0.001	down	-2.0375903	Plcd3	Mus musculus phospholipase C, delta 3 (Plcd3), mRNA [NM_152813]	72469	NM_152813	phospholipase C, delta 3	NM_152813	Mm.264743
A_51_P486121	0.020	down	-2.0426779	Aff3	Mus musculus AF4/FMR2 family, member 3 (Aff3), mRNA [NM_010678]	16764	NM_010678	AF4/FMR2 family, member 3	NM_010678	Mm.336679
A_55_P2004652	0.017	down	-2.0526187	Klrc1	Mus musculus killer cell lectin-like receptor subfamily C, member 1 (Klrc1), transcript variant 2, mRNA [NM_010652]	16641	NM_010652	killer cell lectin-like receptor subfamily C, member 1	NM_010652	Mm.56899
A_66_P107818	0.004	down	-2.0528982	Gdf3	Mus musculus growth differentiation factor 3 (Gdf3), mRNA [NM_008108]	14562	NM_008108	growth differentiation factor 3	NM_008108	Mm.299742
A_51_P186487	0.001	down	-2.0580676	Pik3c2b	phosphoinositide-3-kinase, class 2, beta polypeptide [Source:MGI Symbol;Acc:MGI:2685045] [ENSMUST00000124934]	240752	AK049181	phosphoinositide-3-kinase, class 2, beta polypeptide		Mm.387308
A_51_P126067	0.021	down	-2.060553	Cd2	Mus musculus CD2 antigen (Cd2), mRNA [NM_013486]	12481	NM_013486	CD2 antigen	NM_013486	Mm.22842
A_66_P121110	0.008	down	-2.0624528	Pck1	Mus musculus phosphoenolpyruvate carboxykinase 1, cytosolic (Pck1), mRNA [NM_011044]	18534	NM_011044	phosphoenolpyruvate carboxykinase 1, cytosolic	NM_011044	Mm.266867
A_65_P17218	0.001	down	-2.071359	Mndal	Mus musculus myeloid nuclear differentiation antigen like (Mndal), mRNA [NM_001170853]	1E+08	NM_001170853	myeloid nuclear differentiation antigen like	NM_001170853	Mm.463437
A_51_P254855	0.007	down	-2.0753784	Ptgs2	Mus musculus prostaglandin-endoperoxide synthase 2 (Ptgs2), mRNA [NM_011198]	19225	NM_011198	prostaglandin-endoperoxide synthase 2	NM_011198	Mm.292547
A_55_P2037338	0.001	down	-2.0792997	Plcd3	Mus musculus phospholipase C, delta 3 (Plcd3), mRNA [NM_152813]	72469	NM_152813	phospholipase C, delta 3	NM_152813	Mm.264743
A_55_P2033864	0.000	down	-2.0900924	Plekha8	Mus musculus pleckstrin homology domain containing, family A (phosphoinositide binding specific) member 8 (Plekha8), transcript variant 1, mRNA [NM_001164361]	231999	NM_001164361	pleckstrin homology domain containing, family A (phosphoinositide binding specific) member 8	NM_001164361	Mm.35416
A_51_P117794	0.027	down	-2.0970254	Bik	Mus musculus BCL2-interacting killer (Bik), mRNA [NM_007546]	12124	NM_007546	BCL2-interacting killer	NM_007546	Mm.267006
A_55_P2118520	0.044	down	-2.104309	Col1a1	Mus musculus collagen, type I, alpha 1 (Col1a1), mRNA [NM_007742]	12842	NM_007742	collagen, type I, alpha 1	NM_007742	Mm.277735
A_55_P2112185	0.001	down	-2.1213925	Nhsl1	Mus musculus NHS-like 1 (Nhsl1), transcript variant 1, mRNA [NM_173390]	215819	NM_173390	NHS-like 1	NM_173390	Mm.297971
A_55_P2046807	0.050	down	-2.135104	Sparc	Mus musculus secreted acidic cysteine rich glycoprotein (Sparc), mRNA [NM_009242]	20692	NM_009242	secreted acidic cysteine rich glycoprotein	NM_009242	Mm.291442
A_51_P341349	0.016	down	-2.1454515	Mtap	Mus musculus methylthioadenosine phosphorylase (Mtap), mRNA [NM_024433]	66902	NM_024433	methylthioadenosine phosphorylase	NM_024433	Mm.28500
A_51_P162671	0.006	down	-2.1478426	Folr2	Mus musculus folate receptor 2 (fetal) (Folr2), mRNA [NM_008035]	14276	NM_008035	folate receptor 2 (fetal)	NM_008035	Mm.439666

A_52_P138926	0.003	down	-2.1545227	Slc32a1	Mus musculus solute carrier family 32 (GABA vesicular transporter), member 1 (Slc32a1), mRNA [NM_009508]	22348	NM_009508	solute carrier family 32 (GABA vesicular transporter), member 1	NM_009508	Mm.413854
A_55_P2036357	0.004	down	-2.1581376	Pyhin1	Mus musculus pyrin and HIN domain family, member 1 (Pyhin1), mRNA [NM_175026]	236312	NM_175026	pyrin and HIN domain family, member 1	NM_175026	Mm.447
A_55_P1959122	0.035	down	-2.164862	Cd164l2	CD164 sialomucin-like 2 [Source:MGI Symbol;Acc:MGI:1916905] [ENSMUST00000150744]	69655		CD164 sialomucin-like 2		
A_51_P230298	0.045	down	-2.1672788	Hdgfl1	Mus musculus hepatoma derived growth factor-like 1 (Hdgfl1), mRNA [NM_008232]	15192	NM_008232	hepatoma derived growth factor-like 1	NM_008232	Mm.7411
A_52_P201206	0.023	down	-2.1692154	Scrn1	Mus musculus secernin 1 (Scrn1), mRNA [NM_027268]	69938	NM_027268	secernin 1	NM_027268	Mm.41557
A_55_P2077901	0.023	down	-2.1759062	Cd2	Mus musculus CD2 antigen (Cd2), mRNA [NM_013486]	12481	NM_013486	CD2 antigen	NM_013486	Mm.22842
A_55_P2089233	0.026	down	-2.1822374	Pou2af1	Mus musculus POU domain, class 2, associating factor 1 (Pou2af1), mRNA [NM_011136]	18985	NM_011136	POU domain, class 2, associating factor 1	NM_011136	Mm.897
A_51_P456208	0.001	down	-2.183206	Tff3	Mus musculus trefoil factor 3, intestinal (Tff3), mRNA [NM_011575]	21786	NM_011575	trefoil factor 3, intestinal	NM_011575	Mm.4641
A_55_P2086433	0.005	down	-2.1834598	Oasl1	Mus musculus 2'-5' oligoadenylate synthetase-like 1 (Oasl1), mRNA [NM_145209]	231655	NM_145209	2'-5' oligoadenylate synthetase-like 1	NM_145209	Mm.95479
A_51_P327751	0.048	down	-2.1883204	Ifit1	Mus musculus interferon-induced protein with tetratricopeptide repeats 1 (Ifit1), mRNA [NM_008331]	15957	NM_008331	interferon-induced protein with tetratricopeptide repeats 1	NM_008331	Mm.439751
A_51_P154485	0.049	down	-2.192039	Fcrla	Mus musculus Fc receptor-like A (Fcrla), transcript variant 2, mRNA [NM_145141]	98752	NM_145141	Fc receptor-like A	NM_145141	Mm.1850
A_55_P2053923	0.011	down	-2.1941953	Rab17	Mus musculus RAB17, member RAS oncogene family (Rab17), transcript variant 1, mRNA [NM_008998]	19329	NM_008998	RAB17, member RAS oncogene family	NM_008998	Mm.279780
A_55_P1974209	0.037	down	-2.2025807	Kif4-ps	Mus musculus kinesin family member 4, pseudogene (Kif4-ps), non-coding RNA [NR_033653]	74947	NR_033653	kinesin family member 4, pseudogene	NR_033653	Mm.488905
A_51_P514085	0.040	down	-2.2107437	Mx2	Mus musculus myxovirus (influenza virus) resistance 2 (Mx2), transcript variant 1, mRNA [NM_013606]	17858	NM_013606	myxovirus (influenza virus) resistance 2	NM_013606	Mm.14157
A_51_P412926	0.021	down	-2.2109685	Krt27	Mus musculus keratin 27 (Krt27), mRNA [NM_010666]	16675	NM_010666	keratin 27	NM_010666	Mm.440476
A_55_P1955457	0.047	down	-2.2118556	Grb10	Mus musculus growth factor receptor bound protein 10 (Grb10), transcript variant 1, mRNA [NM_010345]	14783	NM_010345	growth factor receptor bound protein 10	NM_010345	Mm.273117
A_52_P519783	0.015	down	-2.2125576	Ltk	Mus musculus leukocyte tyrosine kinase (Ltk), transcript variant 4, mRNA [NM_203345]	17005	NM_203345	leukocyte tyrosine kinase	NM_203345	Mm.1740
A_55_P2082961	0.000	down	-2.2182639	Fnbp1l	Mus musculus formin binding protein 1-like (Fnbp1l), transcript variant 1, mRNA [NM_001114665]	214459	NM_001114665	formin binding protein 1-like	NM_001114665	Mm.209491
A_55_P2126951	0.003	down	-2.2253652	Zfp467	Mus musculus zinc finger protein 467 (Zfp467), transcript variant 4, mRNA [NM_001085417]	68910	NM_001085417	zinc finger protein 467	NM_001085417	Mm.2464
A_55_P2039230	0.027	down	-2.2299294	Pydc4	pyrin domain containing 4 [Source:MGI Symbol;Acc:MGI:3695276] [ENSMUST00000178186]	623121	AK165868	pyrin domain containing 4		Mm.138449
A_55_P1962918	0.000	down	-2.2301056	Mnda	Mus musculus myeloid cell nuclear differentiation antigen (Mnda), mRNA [NM_001033450]	381308	NM_001033450	myeloid cell nuclear differentiation antigen	NM_001033450	Mm.490248
A_55_P2034864	0.006	down	-2.2322278	Tubb2b	Mus musculus tubulin, beta 2B class IIB (Tubb2b), mRNA [NM_023716]	73710	NM_023716	tubulin, beta 2B class IIB	NM_023716	Mm.379227
A_51_P133884	0.016	down	-2.2361448	Cd209a	Mus musculus CD209a antigen (Cd209a), mRNA [NM_133238]	170786	NM_133238	CD209a antigen	NM_133238	Mm.32510
A_51_P245414	0.027	down	-2.2394443	Klk1	Mus musculus kallikrein 1 (Klk1), mRNA [NM_010639]	16612	NM_010639	kallikrein 1	NM_010639	Mm.142722
A_55_P2004536	0.008	down	-2.2661996	Klra4	Mus musculus killer cell lectin-like receptor, subfamily A, member 4 (Klra4), transcript variant 1, mRNA [NM_010649]	16635	NM_010649	killer cell lectin-like receptor, subfamily A, member 4	NM_010649	Mm.333431
A_52_P400831	0.039	down	-2.2677658	Adamts13	Mus musculus a disintegrin-like and metallopeptidase (reprolysin type) with thrombospondin type 1 motif, 13 (Adamts13), mRNA [NM_001001322]	279028	NM_001001322	a disintegrin-like and metallopeptidase (reprolysin type) with thrombospondin type 1 motif, 13	NM_001001322	Mm.330084
A_55_P1977464	0.005	down	-2.2694492	Dab2ip	Mus musculus disabled 2 interacting protein (Dab2ip), transcript variant 1, mRNA [NM_001114125]	69601	NM_001114125	disabled 2 interacting protein	NM_001114125	Mm.29629
A_55_P1974178	0.002	down	-2.2719097	Pyhin1	Mus musculus pyrin and HIN domain family, member 1 (Pyhin1), mRNA [NM_175026]	236312	NM_175026	pyrin and HIN domain family, member 1	NM_175026	Mm.447
A_55_P1966833	0.004	down	-2.2852979	Xaf1	Mus musculus XIAP associated factor 1 (Xaf1), mRNA [NM_001037713]	327959	NM_001037713	XIAP associated factor 1	NM_001037713	Mm.291131
A_55_P2081585	0.030	down	-2.2860372	Dennd5b	Mus musculus DENN/MADD domain containing 5B (Dennd5b), mRNA [NM_177192]	320560	NM_177192	DENN/MADD domain containing 5B	NM_177192	Mm.426874
A_55_P2121558	0.004	down	-2.2871659	Ankfy1	Mus musculus ankyrin repeat and FYVE domain containing 1 (Ankfy1), mRNA [NM_009671]	11736	NM_009671	ankyrin repeat and FYVE domain containing 1	NM_009671	Mm.10313
A_55_P1966838	0.005	down	-2.2883193	Xaf1	Mus musculus XIAP associated factor 1 (Xaf1), mRNA [NM_001037713]	327959	NM_001037713	XIAP associated factor 1	NM_001037713	Mm.291131
A_55_P1999453	0.030	down	-2.290442	Zfp781	Mus musculus zinc finger protein 781 (Zfp781), mRNA [NM_199062]	331188	NM_199062	zinc finger protein 781	NM_199062	Mm.351497

A_55_P2155479	0.014	down	-2.2973633	Eps8	Mus musculus epidermal growth factor receptor pathway substrate 8 (Eps8), transcript variant 1, mRNA [NM_007945]	13860	NM_007945	epidermal growth factor receptor pathway substrate 8	NM_007945	Mm.235346
A_55_P2119633	0.000	down	-2.2992773	Gnal	Mus musculus guanine nucleotide binding protein, alpha stimulating, olfactory type (Gnal), transcript variant 2, mRNA [NM_177137]	14680	NM_177137	guanine nucleotide binding protein, alpha stimulating, olfactory type	NM_177137	Mm.440746
A_52_P219351	0.033	down	-2.3016686	Ssx9	Mus musculus synovial sarcoma, X breakpoint 9 (Ssx9), mRNA [NM_199063]	382206	NM_199063	synovial sarcoma, X breakpoint 9	NM_199063	Mm.391220
A_52_P663413	0.001	down	-2.327122	P4ha2	Mus musculus procollagen-proline, 2-oxoglutarate 4-dioxygenase (proline 4-hydroxylase), alpha II polypeptide (P4ha2), transcript variant 2, mRNA [NM_011031]	18452	NM_011031	procollagen-proline, 2-oxoglutarate 4-dioxygenase (proline 4-hydroxylase), alpha II polypeptide	NM_011031	Mm.3705
A_55_P2150717	0.004	down	-2.3325248	Eomes	Mus musculus eomesodermin homolog (Xenopus laevis) (Eomes), transcript variant 1, mRNA [NM_010136]	13813	NM_010136	eomesodermin homolog (Xenopus laevis)	NM_010136	Mm.200692
A_52_P561161	0.017	down	-2.333651	Dennd5b	Mus musculus DENN/MADD domain containing 5B (Dennd5b), mRNA [NM_177192]	320560	NM_177192	DENN/MADD domain containing 5B	NM_177192	Mm.426874
A_51_P449867	0.009	down	-2.347581	Nyx	Mus musculus nyctalopin (Nyx), mRNA [NM_173415]	236690	NM_173415	nyctalopin	NM_173415	Mm.90233
A_55_P2151638	0.003	down	-2.364333	Klra15	Mus musculus killer cell lectin-like receptor, subfamily A, member 15 (Klra15), mRNA [NM_013793]	27423	NM_013793	killer cell lectin-like receptor, subfamily A, member 15	NM_013793	Mm.24399
A_55_P2002757	0.029	down	-2.3934357	Blnk	Mus musculus B cell linker (Blnk), mRNA [NM_008528]	17060	NM_008528	B cell linker	NM_008528	Mm.9749
A_51_P117226	0.000	down	-2.4025548	Zdhhc2	zinc finger, DHHC domain containing 2 [Source:MGI Symbol;Acc:MGI:1923452] [ENSMUST00000049389]	70546	AK046533	zinc finger, DHHC domain containing 2		Mm.34326
A_66_P139683	0.002	down	-2.4113336	Zbp1	Mus musculus Z-DNA binding protein 1 (Zbp1), transcript variant 1, mRNA [NM_021394]	58203	NM_021394	Z-DNA binding protein 1	NM_021394	Mm.116687
A_52_P536927	0.013	down	-2.4136431	Satb2	Mus musculus special AT-rich sequence binding protein 2 (Satb2), mRNA [NM_139146]	212712	NM_139146	special AT-rich sequence binding protein 2	NM_139146	Mm.145599
A_55_P2160825	0.015	down	-2.417139	Xdh	Mus musculus 10 days lactation, adult female mammary gland cDNA, RIKEN full-length enriched library, clone:D730018C08 product:xanthine dehydrogenase, full insert sequence. [AK164764]	22436	AK164764	xanthine dehydrogenase		Mm.11223
A_55_P2122605	0.000	down	-2.4202843	Cbr2	Mus musculus carbonyl reductase 2 (Cbr2), mRNA [NM_007621]	12409	NM_007621	carbonyl reductase 2	NM_007621	Mm.21454
A_52_P138205	0.000	down	-2.421922	Plekha8	Mus musculus pleckstrin homology domain containing, family A (phosphoinositide binding specific) member 8 (Plekha8), transcript variant 1, mRNA [NM_001164361]	231999	NM_001164361	pleckstrin homology domain containing, family A (phosphoinositide binding specific) member 8	NM_001164361	Mm.35416
A_55_P2156643	0.024	down	-2.4229815	Tas2r130	Mus musculus taste receptor, type 2, member 130 (Tas2r130), mRNA [NM_199156]	387355	NM_199156	taste receptor, type 2, member 130	NM_199156	Mm.377916
A_52_P86693	0.000	down	-2.427836	Ifi27	Mus musculus interferon, alpha-inducible protein 27 (Ifi27), transcript variant 1, mRNA [NM_026790]	52668	NM_026790	interferon, alpha-inducible protein 27	NM_026790	Mm.2121
A_55_P2002933	0.034	down	-2.4476793	Klk1b5	Mus musculus kallikrein 1-related peptidase b5 (Klk1b5), mRNA [NM_008456]	16622	NM_008456	kallikrein 1-related peptidase b5	NM_008456	Mm.443373
A_51_P161086	0.000	down	-2.4964077	Ppic	Mus musculus peptidylprolyl isomerase C (Ppic), mRNA [NM_008908]	19038	NM_008908	peptidylprolyl isomerase C	NM_008908	Mm.4587
A_55_P2154425	0.019	down	-2.5042806	Cd320	Mus musculus CD320 antigen (Cd320), mRNA [NM_019421]	54219	NM_019421	CD320 antigen	NM_019421	Mm.11175
A_55_P2094060	0.010	down	-2.5580933	Gzma	Mus musculus granzyme A (Gzma), mRNA [NM_010370]	14938	NM_010370	granzyme A	NM_010370	Mm.15510
A_55_P2109445	0.049	down	-2.581793	Irf4	Mus musculus interferon regulatory factor 4 (Irf4), mRNA [NM_013674]	16364	NM_013674	interferon regulatory factor 4	NM_013674	Mm.4677
A_55_P2176963	0.001	down	-2.6083279	Hsph1	Mus musculus heat shock 105kDa/110kDa protein 1 (Hsph1), mRNA [NM_013559]	15505	NM_013559	heat shock 105kDa/110kDa protein 1	NM_013559	Mm.270681
A_55_P1961335	0.028	down	-2.608842	Ctsk	Mus musculus cathepsin K (Ctsk), mRNA [NM_007802]	13038	NM_007802	cathepsin K	NM_007802	Mm.272085
A_66_P124179	0.021	down	-2.6151643	Atp6v0d2	Mus musculus ATPase, H+ transporting, lysosomal V0 subunit D2 (Atp6v0d2), mRNA [NM_175406]	242341	NM_175406	ATPase, H+ transporting, lysosomal V0 subunit D2	NM_175406	Mm.19298
A_51_P516323	0.019	down	-2.6250293	Igkv9-129	Mus musculus immunoglobulin kappa light chain mRNA, partial cds. [AY498738]	692182	AY498738	immunoglobulin kappa variable 9-129		Mm.370329
A_55_P2090330	0.002	down	-2.6368647	Kcnmb4	Mus musculus potassium large conductance calcium-activated channel, subfamily M, beta member 4 (Kcnmb4), mRNA [NM_021452]	58802	NM_021452	potassium large conductance calcium-activated channel, subfamily M, beta member 4	NM_021452	Mm.440652
A_55_P2003513	0.005	down	-2.6571896	Hsph1	Mus musculus heat shock 105kDa/110kDa protein 1 (Hsph1), mRNA [NM_013559]	15505	NM_013559	heat shock 105kDa/110kDa protein 1	NM_013559	Mm.270681
A_51_P349546	0.006	down	-2.701737	Cd109	Mus musculus CD109 antigen (Cd109), mRNA [NM_153098]	235505	NM_153098	CD109 antigen	NM_153098	Mm.32955
A_55_P2125663	0.005	down	-2.7496655	Rab11fip4os1	Mus musculus Rab11fib4 opposite strand transcript 1 (Rab11fip4os1), long non-coding RNA [NR_003283]	790912	NR_003283	Rab11fib4 opposite strand transcript 1	NR_003283	Mm.447313
A_52_P398998	0.007	down	-2.7676437	Gfra1	Mus musculus glial cell line derived neurotrophic factor family receptor alpha 1 (Gfra1), transcript variant 1, mRNA [NM_010279]	14585	NM_010279	glial cell line derived neurotrophic factor family receptor alpha 1	NM_010279	Mm.88367

A_55_P2118441	0.003	down	-2.8091288	Mx1	Mus musculus myxovirus (influenza virus) resistance 1 (Mx1), mRNA [NM_010846]	17857	NM_010846	myxovirus (influenza virus) resistance 1	NM_010846	Mm.33996
A_55_P1982454	0.039	down	-2.8746147	Eps8	Mus musculus epidermal growth factor receptor pathway substrate 8 (Eps8), transcript variant 1, mRNA [NM_007945]	13860	NM_007945	epidermal growth factor receptor pathway substrate 8	NM_007945	Mm.235346
A_51_P200517	0.000	down	-2.9009902	Rasgef1a	Mus musculus RasGEF domain family, member 1A (Rasgef1a), mRNA [NM_027526]	70727	NM_027526	RasGEF domain family, member 1A	NM_027526	Mm.87660
A_55_P2126192	0.026	down	-2.908956	Lgr5	Mus musculus leucine rich repeat containing G protein coupled receptor 5 (Lgr5), mRNA [NM_010195]	14160	NM_010195	leucine rich repeat containing G protein coupled receptor 5	NM_010195	Mm.42103
A_51_P445562	0.009	down	-2.919474	Chst10	Mus musculus carbohydrate sulfotransferase 10 (Chst10), mRNA [NM_145142]	98388	NM_145142	carbohydrate sulfotransferase 10	NM_145142	Mm.260054
A_55_P1992234	0.043	down	-2.9504228	Slx	Mus musculus Sycp3 like X-linked (Slx), mRNA [NM_001136476]	664829	NM_001136476	Sycp3 like X-linked	NM_001136476	Mm.489202
A_51_P485161	0.032	down	-2.9796066	Cplx2	Mus musculus complexin 2 (Cplx2), mRNA [NM_009946]	12890	NM_009946	complexin 2	NM_009946	Mm.268902
A_55_P2106800	0.027	down	-2.994774	Bglap	Mus musculus bone gamma carboxyglutamate protein (Bglap), transcript variant 2, mRNA [NM_001037939]	12096	NM_001037939	bone gamma carboxyglutamate protein	NM_001037939	Mm.389459
A_55_P1961625	0.046	down	-3.0287638	Tcl1b5	Mus musculus T cell leukemia/lymphoma 1B, 5 (Tcl1b5), mRNA [NM_013776]	27382	NM_013776	T cell leukemia/lymphoma 1B, 5	NM_013776	Mm.441379
A_55_P2106801	0.020	down	-3.0979645	Bglap3	Mus musculus bone gamma-carboxyglutamate protein 3 (Bglap3), mRNA [NM_031368]	12095	NM_031368	bone gamma-carboxyglutamate protein 3	NM_031368	Mm.482191
A_51_P421538	0.029	down	-3.1383939	Slc9b2	Mus musculus solute carrier family 9, subfamily B (NHA2, cation proton antiporter 2), member 2 (Slc9b2), mRNA [NM_178877]	97086	NM_178877	solute carrier family 9, subfamily B (NHA2, cation proton antiporter 2), member 2	NM_178877	Mm.441764
A_55_P1956370	0.035	down	-3.1505296	Bach2	Mus musculus BTB and CNC homology 2 (Bach2), mRNA [NM_001109661]	12014	NM_001109661	BTB and CNC homology 2	NM_001109661	Mm.491223
A_51_P368496	0.000	down	-3.1752918	Tmem98	Mus musculus transmembrane protein 98 (Tmem98), mRNA [NM_029537]	103743	NM_029537	transmembrane protein 98	NM_029537	Mm.27682
A_52_P68893	0.006	down	-3.2317677	Ifng	Mus musculus interferon gamma (Ifng), mRNA [NM_008337]	15978	NM_008337	interferon gamma	NM_008337	Mm.240327
A_55_P2077083	0.045	down	-3.2710755	Tmem132e	Mus musculus transmembrane protein 132E (Tmem132e), mRNA [NM_023438]	270893	NM_023438	transmembrane protein 132E	NM_023438	Mm.341593
A_55_P2082786	0.000	down	-3.3450563	Trib2	Mus musculus tribbles homolog 2 (Drosophila) (Trib2), mRNA [NM_144551]	217410	NM_144551	tribbles homolog 2 (Drosophila)	NM_144551	Mm.491136
A_51_P209122	0.050	down	-3.729841	Agr2	Mus musculus anterior gradient 2 (Agr2), mRNA [NM_011783]	23795	NM_011783	anterior gradient 2	NM_011783	Mm.7244
A_51_P472867	0.000	down	-3.7368424	Oas3	Mus musculus 2'-5' oligoadenylate synthetase 3 (Oas3), mRNA [NM_145226]	246727	NM_145226	2'-5' oligoadenylate synthetase 3	NM_145226	Mm.204887
A_51_P304397	0.016	down	-4.0262437	Cpm	Mus musculus carboxypeptidase M (Cpm), mRNA [NM_027468]	70574	NM_027468	carboxypeptidase M	NM_027468	Mm.339332
A_51_P487690	0.001	down	-4.1304617	Ifi44	Mus musculus interferon-induced protein 44 (Ifi44), mRNA [NM_133871]	99899	NM_133871	interferon-induced protein 44	NM_133871	Mm.30756
A_51_P304170	0.000	down	-4.1311693	Rtp4	Mus musculus receptor transporter protein 4 (Rtp4), mRNA [NM_023386]	67775	NM_023386	receptor transporter protein 4	NM_023386	Mm.475107
A_51_P359570	0.001	down	-4.1789713	Ifit3	Mus musculus interferon-induced protein with tetratricopeptide repeats 3 (Ifit3), mRNA [NM_010501]	15959	NM_010501	interferon-induced protein with tetratricopeptide repeats 3	NM_010501	Mm.426079
A_55_P2106803	0.016	down	-4.264808	Bglap2	Mus musculus bone gamma-carboxyglutamate protein 2 (Bglap2), mRNA [NM_001032298]	12097	NM_001032298	bone gamma-carboxyglutamate protein 2	NM_001032298	Mm.425160
A_55_P1998942	0.000	down	-4.5850224	Oas1a	Mus musculus 2'-5' oligoadenylate synthetase 1A (Oas1a), mRNA [NM_145211]	246730	NM_145211	2'-5' oligoadenylate synthetase 1A	NM_145211	Mm.14301
A_51_P154842	0.000	down	-4.89793	Oas1f	Mus musculus 2'-5' oligoadenylate synthetase 1F (Oas1f), mRNA [NM_145153]	243262	NM_145153	2'-5' oligoadenylate synthetase 1F	NM_145153	Mm.105369
A_55_P1998943	0.000	down	-5.1482286	Oas1a	Mus musculus 2'-5' oligoadenylate synthetase 1A (Oas1a), mRNA [NM_145211]	246730	NM_145211	2'-5' oligoadenylate synthetase 1A	NM_145211	Mm.14301
A_55_P2019719	0.000	down	-6.9711046	Oas2	Mus musculus 2'-5' oligoadenylate synthetase 2 (Oas2), mRNA [NM_145227]	246728	NM_145227	2'-5' oligoadenylate synthetase 2	NM_145227	Mm.260926
A_52_P90363	0.000	down	-8.661083	Ifi27l2a	Mus musculus interferon, alpha-inducible protein 27 like 2A (Ifi27l2a), transcript variant 1, mRNA [NM_029803]	76933	NM_029803	interferon, alpha-inducible protein 27 like 2A	NM_029803	Mm.271275
A_51_P244504	0.049	up	1.1706135	Oraov1	Mus musculus oral cancer overexpressed 1 (Oraov1), mRNA [NM_028184]	72284	NM_028184	oral cancer overexpressed 1	NM_028184	Mm.389995
A_52_P386837	0.046	up	1.1897619	Trmu	Mus musculus tRNA 5-methylaminomethyl-2-thiouridylate methyltransferase (Trmu), mRNA [NM_028063]	72026	NM_028063	tRNA 5-methylaminomethyl-2-thiouridylate methyltransferase	NM_028063	Mm.158211
A_55_P2173353	0.044	up	1.1936729	Mroh5	Mus musculus maestro heat-like repeat family member 5 (Mroh5), mRNA [NM_001033365]	268816	NM_001033365	maestro heat-like repeat family member 5	NM_001033365	Mm.440635
A_51_P284686	0.048	up	1.1949359	Aak1	Mus musculus AP2 associated kinase 1 (Aak1), transcript variant 1, mRNA [NM_001040106]	269774	NM_001040106	AP2 associated kinase 1	NM_001040106	Mm.221038
A_55_P2015742	0.043	up	1.1956816	Cggbp1	Mus musculus CGG triplet repeat binding protein 1 (Cggbp1), mRNA [NM_178647]	106143	NM_178647	CGG triplet repeat binding protein 1	NM_178647	Mm.10160
A_55_P1954266	0.034	up	1.2024848	Zfp672	Mus musculus zinc finger protein 672 (Zfp672), transcript variant 5, mRNA [NM_001256518]	319475	NM_001256518	zinc finger protein 672	NM_001256518	Mm.72124

A_55_P2175206	0.037	up	1.2028623	Rbbp6	Mus musculus retinoblastoma binding protein 6 (Rbbp6), transcript variant 2, mRNA [NM_175023]	19647	NM_175023	retinoblastoma binding protein 6	NM_175023	Mm.4480
A_51_P333071	0.043	up	1.2086023	Anapc16	Mus musculus anaphase promoting complex subunit 16 (Anapc16), mRNA [NM_025514]	52717	NM_025514	anaphase promoting complex subunit 16	NM_025514	Mm.21793
A_52_P269461	0.028	up	1.2089517	Ddhd2	Mus musculus DDHD domain containing 2 (Ddhd2), mRNA [NM_028102]	72108	NM_028102	DDHD domain containing 2	NM_028102	Mm.246875
A_51_P480073	0.044	up	1.212715	Chad	Mus musculus chondroadherin (Chad), mRNA [NM_007689]	12643	NM_007689	chondroadherin	NM_007689	Mm.440523
A_51_P165914	0.045	up	1.2171824	Zfp770	Mus musculus zinc finger protein 770 (Zfp770), mRNA [NM_175466]	228491	NM_175466	zinc finger protein 770	NM_175466	Mm.132523
A_51_P243207	0.042	up	1.2173404	Zfp574	Mus musculus zinc finger protein 574 (Zfp574), transcript variant 1, mRNA [NM_175477]	232976	NM_175477	zinc finger protein 574	NM_175477	Mm.41568
A_55_P2152387	0.014	up	1.219176	Wwox	Mus musculus WW domain-containing oxidoreductase (Wwox), mRNA [NM_019573]	80707	NM_019573	WW domain-containing oxidoreductase	NM_019573	Mm.440420
A_55_P2016877	0.042	up	1.2210664	Nsd1	Mus musculus nuclear receptor-binding SET-domain protein 1 (Nsd1), mRNA [NM_008739]	18193	NM_008739	nuclear receptor-binding SET-domain protein 1	NM_008739	Mm.168965
A_51_P295192	0.035	up	1.2217946	Nfkbia	Mus musculus nuclear factor of kappa light polypeptide gene enhancer in B cells inhibitor, alpha (Nfkbia), mRNA [NM_010907]	18035	NM_010907	nuclear factor of kappa light polypeptide gene enhancer in B cells inhibitor, alpha	NM_010907	Mm.170515
A_55_P2057994	0.019	up	1.2218629	Mif4gd	Mus musculus MIF4G domain containing (Mif4gd), transcript variant 3, mRNA [NM_001243586]	69674	NM_001243586	MIF4G domain containing	NM_001243586	Mm.390387
A_55_P2185489	0.046	up	1.2228128	Zfp865	Mus musculus zinc finger protein 865 (Zfp865), mRNA [NM_001033383]	319748	NM_001033383	zinc finger protein 865	NM_001033383	Mm.86607
A_55_P1980311	0.037	up	1.2228963	Pcbp2	Mus musculus poly(rC) binding protein 2 (Pcbp2), transcript variant 1, mRNA [NM_001103165]	18521	NM_001103165	poly(rC) binding protein 2	NM_001103165	Mm.236513
A_51_P292368	0.027	up	1.2282636	Tmco6	Mus musculus transmembrane and coiled-coil domains 6 (Tmco6), mRNA [NM_028036]	71983	NM_028036	transmembrane and coiled-coil domains 6	NM_028036	Mm.135188
A_55_P2071952	0.027	up	1.2306263	Wdr92	Mus musculus WD repeat domain 92 (Wdr92), mRNA [NM_178909]	103784	NM_178909	WD repeat domain 92	NM_178909	Mm.298132
A_52_P584415	0.026	up	1.2310164	Cars2	Mus musculus cysteinyl-tRNA synthetase 2 (mitochondrial)(putative) (Cars2), mRNA [NM_024248]	71941	NM_024248	cysteinyl-tRNA synthetase 2 (mitochondrial)(putative)	NM_024248	Mm.196576
A_55_P2109337	0.042	up	1.232147	Gba2	Mus musculus glucosidase beta 2 (Gba2), mRNA [NM_172692]	230101	NM_172692	glucosidase beta 2	NM_172692	Mm.229444
A_51_P468418	0.033	up	1.2339498	Narg2	Mus musculus NMDA receptor-regulated gene 2 (Narg2), mRNA [NM_145618]	93697	NM_145618	NMDA receptor-regulated gene 2	NM_145618	Mm.35911
A_51_P334789	0.041	up	1.2358053	Nudt16	Mus musculus nudix (nucleoside diphosphate linked moiety X)-type motif 16 (Nudt16), mRNA [NM_029385]	75686	NM_029385	nudix (nucleoside diphosphate linked moiety X)-type motif 16	NM_029385	Mm.305810
A_55_P2008417	0.034	up	1.2358162	Mnd1	Mus musculus meiotic nuclear divisions 1 homolog (S. cerevisiae) (Mnd1), mRNA [NM_029797]	76915	NM_029797	meiotic nuclear divisions 1 homolog (S. cerevisiae)	NM_029797	Mm.274829
A_51_P327021	0.033	up	1.2380922	Zbtb25	Mus musculus zinc finger and BTB domain containing 25 (Zbtb25), transcript variant 2, mRNA [NM_028356]	109929	NM_028356	zinc finger and BTB domain containing 25	NM_028356	Mm.41880
A_55_P2122117	0.030	up	1.2396415	Olfr207	Mus musculus olfactory receptor 207 (Olfr207), mRNA [NM_001011792]	257973	NM_001011792	olfactory receptor 207	NM_001011792	Mm.377319
A_55_P2179974	0.020	up	1.2415128	Spsb1	Mus musculus splA/ryanodine receptor domain and SOCS box containing 1 (Spsb1), mRNA [NM_029035]	74646	NM_029035	splA/ryanodine receptor domain and SOCS box containing 1	NM_029035	Mm.30
A_55_P1982727	0.048	up	1.2420528	Dnajb5	Mus musculus DnaJ (Hsp40) homolog, subfamily B, member 5 (Dnajb5), mRNA [NM_019874]	56323	NM_019874	DnaJ (Hsp40) homolog, subfamily B, member 5	NM_019874	Mm.20437
A_55_P2129856	0.042	up	1.244713	Mtfmt	Mus musculus mitochondrial methionyl-tRNA formyltransferase (Mtfmt), mRNA [NM_027134]	69606	NM_027134	mitochondrial methionyl-tRNA formyltransferase	NM_027134	Mm.287956
A_51_P442284	0.041	up	1.2452587	Dnajc12	Mus musculus DnaJ (Hsp40) homolog, subfamily C, member 12 (Dnajc12), transcript variant 1, mRNA [NM_013888]	30045	NM_013888	DnaJ (Hsp40) homolog, subfamily C, member 12	NM_013888	Mm.32550
A_55_P2016712	0.038	up	1.2455252	Msl1	Mus musculus male-specific lethal 1 homolog (Drosophila) (Msl1), mRNA [NM_028722]	74026	NM_028722	male-specific lethal 1 homolog (Drosophila)	NM_028722	Mm.258352
A_52_P144297	0.019	up	1.2461053	Tspyl3	Mus musculus TSPY-like 3 (Tspyl3), mRNA [NM_198617]	241732	NM_198617	TSPY-like 3	NM_198617	Mm.277243
A_66_P120208	0.041	up	1.2468317	Cnnm3	Mus musculus cyclin M3 (Cnnm3), transcript variant 2, mRNA [NM_001039551]	94218	NM_001039551	cyclin M3	NM_001039551	Mm.256323
A_66_P138584	0.048	up	1.2478707	Mnd1	Mus musculus meiotic nuclear divisions 1 homolog (S. cerevisiae) (Mnd1), mRNA [NM_029797]	76915	NM_029797	meiotic nuclear divisions 1 homolog (S. cerevisiae)	NM_029797	Mm.274829
A_52_P610808	0.032	up	1.2482154	Hexdc	Mus musculus hexosaminidase (glycosyl hydrolase family 20, catalytic domain) containing (Hexdc), transcript variant 2, mRNA [NM_001001333]	238023	NM_001001333	hexosaminidase (glycosyl hydrolase family 20, catalytic domain) containing	NM_001001333	Mm.275130
A_55_P2077872	0.028	up	1.2488021	Deaf1	Mus musculus deformed epidermal autoregulatory factor 1 (Drosophila) (Deaf1), transcript variant 4, mRNA [NM_001282076]	54006	NM_001282076	deformed epidermal autoregulatory factor 1 (Drosophila)	NM_001282076	Mm.28392
A_51_P440892	0.043	up	1.2494133	Dph2	Mus musculus DPH2 homolog (S. cerevisiae) (Dph2), mRNA [NM_026344]	67728	NM_026344	DPH2 homolog (S. cerevisiae)	NM_026344	Mm.13823
A_55_P2038237	0.008	up	1.2514842	Rps3	Mus musculus ribosomal protein S3 (Rps3), mRNA [NM_012052]	27050	NM_012052	ribosomal protein S3	NM_012052	Mm.236868
A_55_P2145059	0.048	up	1.2515681	Gbp5	Mus musculus guanylate binding protein 5a (Gbp5) mRNA, complete cds; alternatively spliced. [AF487898]	229898	AF487898	guanylate binding protein 5		
A_55_P1982762	0.049	up	1.2520249	Hsd3b7	Mus musculus hydroxy-delta-5-steroid dehydrogenase, 3 beta- and steroid delta-isomerase 7 (Hsd3b7), transcript variant 1, mRNA [NM_133943]	101502	NM_133943	hydroxy-delta-5-steroid dehydrogenase, 3 beta- and steroid delta-isomerase 7	NM_133943	Mm.157103

A_51_P262340	0.045	up	1.2523875	Rbm3	Mus musculus RNA binding motif protein 3 (Rbm3), transcript variant 2, mRNA [NM_001166409]	19652	NM_001166409	RNA binding motif protein 3	NM_001166409	Mm.360569
A_55_P2047106	0.018	up	1.2555662	Rell2	Mus musculus RELT-like 2 (Rell2), mRNA [NM_153793]	225392	NM_153793	RELT-like 2	NM_153793	Mm.233516
A_51_P376057	0.047	up	1.2563617	Bcorl1	Mus musculus BCL6 co-repressor-like 1 (Bcorl1), mRNA [NM_178782]	320376	NM_178782	BCL6 co-repressor-like 1	NM_178782	Mm.210146
A_55_P2036615	0.044	up	1.256492	Lrif1	Mus musculus ligand dependent nuclear receptor interacting factor 1 (Lrif1), transcript variant 1, mRNA [NM_001039488]	321000	NM_001039488	ligand dependent nuclear receptor interacting factor 1	NM_001039488	Mm.259638
A_55_P2108868	0.036	up	1.2575524	Nufip1	Mus musculus nuclear fragile X mental retardation protein interacting protein 1 (Nufip1), mRNA [NM_013745]	27275	NM_013745	nuclear fragile X mental retardation protein interacting protein 1	NM_013745	Mm.21138
A_51_P490296	0.012	up	1.2593396	Slx4	Mus musculus SLX4 structure-specific endonuclease subunit homolog (S. cerevisiae) (Slx4), mRNA [NM_177472]	52864	NM_177472	SLX4 structure-specific endonuclease subunit homolog (S. cerevisiae)	NM_177472	Mm.29935
A_51_P381321	0.010	up	1.2598284	Ddx17	Mus musculus DEAD (Asp-Glu-Ala-Asp) box polypeptide 17 (Ddx17), transcript variant 1, mRNA [NM_199080]	67040	NM_199080	DEAD (Asp-Glu-Ala-Asp) box polypeptide 17	NM_199080	Mm.29644
A_55_P1977533	0.044	up	1.2602594	Eno3	Mus musculus enolase 3, beta muscle (Eno3), transcript variant 1, mRNA [NM_007933]	13808	NM_007933	enolase 3, beta muscle	NM_007933	Mm.251322
A_52_P363216	0.044	up	1.2608252	Gcnt2	Mus musculus glucosaminyl (N-acetyl) transferase 2, I-branching enzyme (Gcnt2), transcript variant 3, mRNA [NM_133219]	14538	NM_133219	glucosaminyl (N-acetyl) transferase 2, I-branching enzyme	NM_133219	Mm.314757
A_55_P1991440	0.046	up	1.263292	Zscan21	Mus musculus zinc finger and SCAN domain containing 21 (Zscan21), transcript variant 1, mRNA [NM_011757]	22697	NM_011757	zinc finger and SCAN domain containing 21	NM_011757	Mm.2760
A_52_P508317	0.041	up	1.264719	Erlec1	Mus musculus endoplasmic reticulum lectin 1 (Erlec1), mRNA [NM_025745]	66753	NM_025745	endoplasmic reticulum lectin 1	NM_025745	Mm.294641
A_55_P2135039	0.028	up	1.2648722	Hyi	Mus musculus hydroxypyruvate isomerase homolog (E. coli) (Hyi), transcript variant 1, mRNA [NM_026601]	68180	NM_026601	hydroxypyruvate isomerase homolog (E. coli)	NM_026601	Mm.193212
A_55_P2186352	0.035	up	1.2650952	Ttc12	Mus musculus tetratricopeptide repeat domain 12 (Ttc12), mRNA [NM_172770]	235330	NM_172770	tetratricopeptide repeat domain 12	NM_172770	Mm.177413
A_51_P258281	0.039	up	1.2661396	Gal	Mus musculus galanin (Gal), mRNA [NM_010253]	14419	NM_010253	galanin	NM_010253	Mm.4655
A_55_P1986526	0.040	up	1.2662201	Cdh26	Mus musculus cadherin-like 26 (Cdh26), mRNA [NM_198656]	381409	NM_198656	cadherin-like 26	NM_198656	Mm.330860
A_55_P1997977	0.039	up	1.2664095	Rab2b	Mus musculus RAB2B, member RAS oncogene family (Rab2b), mRNA [NM_172601]	76338	NM_172601	RAB2B, member RAS oncogene family	NM_172601	Mm.32870
A_55_P2011220	0.034	up	1.2669866	Armcx1	Mus musculus armadillo repeat containing, X-linked 1 (Armcx1), transcript variant 1, mRNA [NM_001166377]	78248	NM_001166377	armadillo repeat containing, X-linked 1	NM_001166377	Mm.458522
A_55_P2009187	0.036	up	1.2671239	Iffo1	Mus musculus intermediate filament family orphan 1 (Iffo1), transcript variant 1, mRNA [NM_001039669]	320678	NM_001039669	intermediate filament family orphan 1	NM_001039669	Mm.129415
A_55_P1999324	0.025	up	1.2681862	Nudt6	Mus musculus nudix (nucleoside diphosphate linked moiety X)-type motif 6 (Nudt6), mRNA [NM_153561]	229228	NM_153561	nudix (nucleoside diphosphate linked moiety X)-type motif 6	NM_153561	Mm.472818
A_52_P573336	0.049	up	1.268308	Sbsn	Mus musculus suprabasin (Sbsn), transcript variant 1, mRNA [NM_172205]	282619	NM_172205	suprabasin	NM_172205	Mm.250717
A_52_P22781	0.027	up	1.2685411	Zfp866	Mus musculus zinc finger protein 866 (Zfp866), mRNA [NM_177899]	330788	NM_177899	zinc finger protein 866	NM_177899	Mm.41364
A_52_P498985	0.048	up	1.269539	Zfp512	Mus musculus zinc finger protein 512 (Zfp512), mRNA [NM_172993]	269639	NM_172993	zinc finger protein 512	NM_172993	Mm.259127
A_55_P2145611	0.048	up	1.2699789	Krt86	Mus musculus keratin 86 (Krt86), mRNA [NM_010667]	16679	NM_010667	keratin 86	NM_010667	Mm.383183
A_51_P415607	0.017	up	1.2703276	Fkbp7	Mus musculus FK506 binding protein 7 (Fkbp7), mRNA [NM_010222]	14231	NM_010222	FK506 binding protein 7	NM_010222	Mm.24720
A_55_P2065953	0.015	up	1.2710875	Btbd19	Mus musculus BTB (POZ) domain containing 19 (Btbd19), long non-coding RNA [NR_024078]	78611	NR_024078	BTB (POZ) domain containing 19	NR_024078	Mm.446227
A_55_P2130256	0.010	up	1.2717005	Polr2l	Mus musculus polymerase (RNA) II (DNA directed) polypeptide L (Polr2l), mRNA [NM_025593]	66491	NM_025593	polymerase (RNA) II (DNA directed) polypeptide L	NM_025593	Mm.380115
A_55_P2059986	0.020	up	1.2720722	Chst14	Mus musculus carbohydrate (N-acetylgalactosamine 4-0) sulfotransferase 14 (Chst14), mRNA [NM_028117]	72136	NM_028117	carbohydrate (N-acetylgalactosamine 4-0) sulfotransferase 14	NM_028117	Mm.490292
A_55_P2127825	0.046	up	1.2731704	Bcas3	breast carcinoma amplified sequence 3 [Source:MGI Symbol;Acc:MGI:2385848] [ENSMUST00000108061]	192197	AK011603	breast carcinoma amplified sequence 3		Mm.287663
A_51_P270899	0.029	up	1.2739398	Zfp61	Mus musculus zinc finger protein 61 (Zfp61), mRNA [NM_009561]	22719	NM_009561	zinc finger protein 61	NM_009561	Mm.12832
A_55_P2017082	0.045	up	1.2741836	Hnrnpa2b1	Mus musculus heterogeneous nuclear ribonucleoprotein A2/B1 (Hnrnpa2b1), transcript variant 1, mRNA [NM_016806]	53379	NM_016806	heterogeneous nuclear ribonucleoprotein A2/B1	NM_016806	Mm.155896
A_55_P2058746	0.034	up	1.2749372	Mterf1a	Mus musculus mitochondrial transcription termination factor 1a (Mterf1a), transcript variant 1, mRNA [NM_001013023]	545725	NM_001013023	mitochondrial transcription termination factor 1a	NM_001013023	Mm.99593
A_66_P107584	0.041	up	1.2754631	Fam133b	Mus musculus family with sequence similarity 133, member B (Fam133b), mRNA [NM_001042501]	68152	NM_001042501	family with sequence similarity 133, member B	NM_001042501	Mm.240265
A_51_P399960	0.049	up	1.2761472	Mier2	Mus musculus mesoderm induction early response 1, family member 2 (Mier2), mRNA [NM_027422]	70427	NM_027422	mesoderm induction early response 1, family member 2	NM_027422	Mm.334193
A_51_P437478	0.023	up	1.277285	Zfp566	Mus musculus zinc finger protein 566 (Zfp566), mRNA [NM_152814]	72556	NM_152814	zinc finger protein 566	NM_152814	Mm.441512
A_66_P128431	0.037	up	1.2775207	Fam122b	Mus musculus family with sequence similarity 122, member B (Fam122b), transcript variant 3, mRNA [NM_001166583]	78755	NM_001166583	family with sequence similarity 122, member B	NM_001166583	Mm.28559
A_52_P228964	0.049	up	1.277624	Shprh	Mus musculus SNF2 histone linker PHD RING helicase (Shprh), transcript variant 1, mRNA [NM_001077707]	268281	NM_001077707	SNF2 histone linker PHD RING helicase	NM_001077707	Mm.133101
A_55_P2050688	0.034	up	1.2792528	Zbed4	Mus musculus zinc finger, BED domain containing 4 (Zbed4), mRNA [NM_181412]	223773	NM_181412	zinc finger, BED domain containing 4	NM_181412	Mm.396622

A_55_P2063257	0.030	up	1.2810711	Lgals4	Mus musculus lectin, galactose binding, soluble 4 (Lgals4), mRNA [NM_010706]	16855	NM_010706	lectin, galactose binding, soluble 4	NM_010706	Mm.210336
A_55_P1962026	0.049	up	1.2815945	Pigg	Mus musculus phosphatidylinositol glycan anchor biosynthesis, class G (Pigg), mRNA [NM_001081234]	433931	NM_001081234	phosphatidylinositol glycan anchor biosynthesis, class G	NM_001081234	Mm.319881
A_55_P2080248	0.042	up	1.2823884	Cyp2j8	Mus musculus cytochrome P450, family 2, subfamily j, polypeptide 8 (Cyp2j8), mRNA [NM_001104927]	665095	NM_001104927	cytochrome P450, family 2, subfamily j, polypeptide 8	NM_001104927	Mm.331717
A_52_P563340	0.042	up	1.2831993	Smim4	Mus musculus small integral membrane protein 4 (Smim4), long non-coding RNA [NR_024069]	66487	NR_024069	small integral membrane protein 4	NR_024069	Mm.379116
A_51_P100034	0.012	up	1.2846978	Mif4gd	Mus musculus MIF4G domain containing (Mif4gd), transcript variant 1, mRNA [NM_027162]	69674	NM_027162	MIF4G domain containing	NM_027162	Mm.390387
A_55_P2148821	0.047	up	1.2847723	Agtpbp1	Mus musculus ATP/GTP binding protein 1 (Agtpbp1), transcript variant 3, mRNA [NM_001284218]	67269	NM_001284218	ATP/GTP binding protein 1	NM_001284218	Mm.153008
A_52_P98625	0.041	up	1.2857732	Sowahc	Mus musculus sosondowah ankyrin repeat domain family member C (Sowahc), mRNA [NM_172939]	268301	NM_172939	sosondowah ankyrin repeat domain family member C	NM_172939	Mm.3036
A_55_P2093595	0.050	up	1.2876225	Mmd	Mus musculus monocyte to macrophage differentiation-associated (Mmd), mRNA [NM_026178]	67468	NM_026178	monocyte to macrophage differentiation-associated	NM_026178	Mm.277518
A_51_P497100	0.036	up	1.2886292	Lgals4	Mus musculus lectin, galactose binding, soluble 4 (Lgals4), mRNA [NM_010706]	16855	NM_010706	lectin, galactose binding, soluble 4	NM_010706	Mm.210336
A_51_P137388	0.017	up	1.2889398	Zadh2	Mus musculus zinc binding alcohol dehydrogenase, domain containing 2 (Zadh2), mRNA [NM_146090]	225791	NM_146090	zinc binding alcohol dehydrogenase, domain containing 2	NM_146090	Mm.147052
A_55_P2167984	0.042	up	1.2915101	Mmd	monocyte to macrophage differentiation-associated [Source:MGI Symbol;Acc:MGI:1914718] [ENSMUST00000004050]	67468		monocyte to macrophage differentiation-associated		
A_51_P467751	0.025	up	1.2917763	Hgsnat	Mus musculus heparan-alpha-glucosaminide N-acetyltransferase (Hgsnat), mRNA [NM_029884]	52120	NM_029884	heparan-alpha-glucosaminide N-acetyltransferase	NM_029884	Mm.28326
A_55_P2124425	0.015	up	1.2918094	Zfp661	Mus musculus zinc finger protein 661 (Zfp661), transcript variant 2, mRNA [NM_001111029]	72180	NM_001111029	zinc finger protein 661	NM_001111029	Mm.46715
A_51_P438149	0.029	up	1.2919818	Mapre2	Mus musculus microtubule-associated protein, RP/EB family, member 2 (Mapre2), transcript variant 1, mRNA [NM_153058]	212307	NM_153058	microtubule-associated protein, RP/EB family, member 2	NM_153058	Mm.132237
A_51_P208870	0.047	up	1.292627	Zdhhc24	Mus musculus zinc finger, DHHC domain containing 24 (Zdhhc24), transcript variant 1, mRNA [NM_027476]	70605	NM_027476	zinc finger, DHHC domain containing 24	NM_027476	Mm.36885
A_55_P2063646	0.030	up	1.2927897	Krcc1	Mus musculus lysine-rich coiled-coil 1 (Krcc1), mRNA [NM_145568]	57896	NM_145568	lysine-rich coiled-coil 1	NM_145568	Mm.250569
A_51_P281024	0.027	up	1.2929577	Rad9a	Mus musculus RAD9 homolog A (Rad9a), mRNA [NM_011237]	19367	NM_011237	RAD9 homolog A	NM_011237	Mm.277629
A_52_P91891	0.046	up	1.2931098	Ppp4r1l-ps	Mus musculus protein phosphatase 4, regulatory subunit 1-like, pseudogene (Ppp4r1l-ps), non-coding RNA [NR_027957]	1E+08	NR_027957	protein phosphatase 4, regulatory subunit 1-like, pseudogene	NR_027957	Mm.260590
A_52_P543869	0.038	up	1.2933629	Shpk	Mus musculus sedoheptulokinase (Shpk), mRNA [NM_029031]	74637	NM_029031	sedoheptulokinase	NM_029031	Mm.200905
A_55_P2134785	0.028	up	1.2962847	Zscan22	Mus musculus zinc finger and SCAN domain containing 22 (Zscan22), mRNA [NM_001001447]	232878	NM_001001447	zinc finger and SCAN domain containing 22	NM_001001447	Mm.40706
A_55_P2000329	0.045	up	1.296459	Gskip	Mus musculus GSK3B interacting protein (Gskip), mRNA [NM_178613]	66787	NM_178613	GSK3B interacting protein	NM_178613	Mm.248019
A_55_P2096340	0.007	up	1.2967517	Rnf170	Mus musculus ring finger protein 170 (Rnf170), mRNA [NM_029965]	77733	NM_029965	ring finger protein 170	NM_029965	Mm.291906
A_52_P182520	0.004	up	1.2973466	Timm9	Mus musculus translocase of inner mitochondrial membrane 9 homolog (yeast), mRNA (cDNA clone IMAGE:1332217), with apparent retained intron. [BC028435]	30056	BC028435	translocase of inner mitochondrial membrane 9		
A_55_P2081805	0.033	up	1.2974637	Slc46a1	Mus musculus solute carrier family 46, member 1 (Slc46a1), mRNA [NM_026740]	52466	NM_026740	solute carrier family 46, member 1	NM_026740	Mm.131618
A_55_P2049095	0.028	up	1.2991962	Atat1	Mus musculus alpha tubulin acetyltransferase 1 (Atat1), transcript variant 1, mRNA [NM_001142744]	73242	NM_001142744	alpha tubulin acetyltransferase 1	NM_001142744	Mm.273155
A_55_P2060158	0.033	up	1.3008667	Ermn	Mus musculus ermin, ERM-like protein (Ermn), mRNA [NM_029972]	77767	NM_029972	ermin, ERM-like protein	NM_029972	Mm.91712
A_51_P436817	0.034	up	1.3011658	Dos	Mus musculus downstream of Stk11 (Dos), transcript variant 1, mRNA [NM_001195268]	1E+08	NM_001195268	downstream of Stk11	NM_001195268	Mm.485395
A_52_P550218	0.041	up	1.3019598	Trmt12	Mus musculus tRNA methyltranferase 12 (Trmt12), mRNA [NM_026642]	68260	NM_026642	tRNA methyltranferase 12	NM_026642	Mm.278805
A_55_P2129925	0.043	up	1.3030007	Mybl2	Mus musculus myeloblastosis oncogene-like 2 (Mybl2), mRNA [NM_008652]	17865	NM_008652	myeloblastosis oncogene-like 2	NM_008652	Mm.4594
A_55_P2118173	0.050	up	1.3032016	Igf2	Mus musculus insulin-like growth factor 2 (Igf2), transcript variant 1, mRNA [NM_010514]	16002	NM_010514	insulin-like growth factor 2	NM_010514	Mm.3862
A_55_P2033610	0.006	up	1.3041427	Fastkd3	Mus musculus FAST kinase domains 3 (Fastkd3), mRNA [NM_027123]	69577	NM_027123	FAST kinase domains 3	NM_027123	Mm.23670
A_51_P482043	0.020	up	1.3057742	Epm2aip1	Mus musculus EPM2A (laforin) interacting protein 1 (Epm2aip1), mRNA [NM_175266]	77781	NM_175266	EPM2A (laforin) interacting protein 1	NM_175266	Mm.209005
A_55_P2063626	0.038	up	1.3077599	Matk	Mus musculus megakaryocyte-associated tyrosine kinase (Matk), transcript variant 1, mRNA [NM_010768]	17179	NM_010768	megakaryocyte-associated tyrosine kinase	NM_010768	Mm.2918
A_52_P38208	0.048	up	1.3082095	Arpp21	Mus musculus cyclic AMP-regulated phosphoprotein, 21 (Arpp21), transcript variant 1, mRNA [NM_028755]	74100	NM_028755	cyclic AMP-regulated phosphoprotein, 21	NM_028755	Mm.297444
A_55_P2015912	0.041	up	1.3082486	Zfp961	Mus musculus zinc finger protein 961 (Zfp961), mRNA [NM_001164581]	234413	NM_001164581	zinc finger protein 961	NM_001164581	Mm.334607

A_55_P2096933	0.023	up	1.308314	Cntln	Mus musculus centlein, centrosomal protein (Cntln), transcript variant 2, mRNA [NM_177385]	338349	NM_177385	centlein, centrosomal protein	NM_177385	Mm.440562
A_52_P51429	0.014	up	1.3091097	Dennd1c	Mus musculus DENN/MADD domain containing 1C (Dennd1c), mRNA [NM_153551]	70785	NM_153551	DENN/MADD domain containing 1C	NM_153551	Mm.284447
A_51_P466613	0.024	up	1.3099242	Homer3	Mus musculus homer homolog 3 (Drosophila) (Homer3), transcript variant 2, mRNA [NM_011984]	26558	NM_011984	homer homolog 3 (Drosophila)	NM_011984	Mm.440659
A_51_P359137	0.033	up	1.31199	Doc2g	Mus musculus double C2, gamma (Doc2g), mRNA [NM_021791]	60425	NM_021791	double C2, gamma	NM_021791	Mm.266972
A_52_P64763	0.017	up	1.3121759	Gen1	Mus musculus Gen homolog 1, endonuclease (Drosophila) (Gen1), mRNA [NM_177331]	209334	NM_177331	Gen homolog 1, endonuclease (Drosophila)	NM_177331	Mm.215295
A_52_P577729	0.029	up	1.3124323	Rnf169	Mus musculus ring finger protein 169 (Rnf169), mRNA [NM_175388]	108937	NM_175388	ring finger protein 169	NM_175388	Mm.323087
A_52_P53906	0.021	up	1.3132691	Ccnd2	Mus musculus cyclin D2 (Ccnd2), mRNA [NM_009829]	12444	NM_009829	cyclin D2	NM_009829	Mm.333406
A_55_P2114596	0.015	up	1.313315	Zbtb14	Mus musculus zinc finger and BTB domain containing 14 (Zbtb14), mRNA [NM_009547]	22666	NM_009547	zinc finger and BTB domain containing 14	NM_009547	Mm.29434
A_52_P38964	0.033	up	1.3167218	Sap25	Mus musculus sin3 associated polypeptide (Sap25), mRNA [NM_001081962]	751865	NM_001081962	sin3 associated polypeptide	NM_001081962	Mm.200763
A_51_P285413	0.045	up	1.3171716	Rbbp6	Mus musculus retinoblastoma binding protein 6 (Rbbp6), transcript variant 2, mRNA [NM_175023]	19647	NM_175023	retinoblastoma binding protein 6	NM_175023	Mm.4480
A_55_P2016227	0.024	up	1.3173803	Srcrb4d	Mus musculus scavenger receptor cysteine rich domain containing, group B (4 domains) (Srcrb4d), mRNA [NM_001160366]	109267	NM_001160366	scavenger receptor cysteine rich domain containing, group B (4 domains)	NM_001160366	Mm.332750
A_55_P2076288	0.012	up	1.3192103	Zfp661	Mus musculus zinc finger protein 661 (Zfp661), transcript variant 2, mRNA [NM_001111029]	72180	NM_001111029	zinc finger protein 661	NM_001111029	Mm.46715
A_55_P2044252	0.008	up	1.3200214	Znf512b	Mus musculus zinc finger protein 512B (Znf512b), mRNA [NM_001164597]	269401	NM_001164597	zinc finger protein 512B	NM_001164597	Mm.386888
A_55_P1975177	0.024	up	1.3223342	Acyp1	Mus musculus 18-day embryo whole body cDNA, RIKEN full-length enriched library, clone:1110050B14 product:acylphosphatase 1, erythrocyte (common) type, full insert sequence. [AK027969]	66204	AK027969	acylphosphatase 1, erythrocyte (common) type		Mm.311985
A_51_P327232	0.049	up	1.3234628	Lenep	Mus musculus lens epithelial protein (Lenep), mRNA [NM_020517]	57275	NM_020517	lens epithelial protein	NM_020517	Mm.482283
A_55_P2316335	0.025	up	1.3235034	Gfm2	Mus musculus G elongation factor, mitochondrial 2 (Gfm2), transcript variant 2, mRNA [NM_001146043]	320806	NM_001146043	G elongation factor, mitochondrial 2	NM_001146043	Mm.219675
A_55_P2045050	0.028	up	1.324554	Zfp953	Mus musculus zinc finger protein 953 (Zfp953), mRNA [NM_001038651]	629016	NM_001038651	zinc finger protein 953	NM_001038651	Mm.440800
A_55_P2014570	0.016	up	1.3246319	Zfp961	Mus musculus zinc finger protein 961 (Zfp961), mRNA [NM_001164581]	234413	NM_001164581	zinc finger protein 961	NM_001164581	Mm.334607
A_65_P12392	0.015	up	1.3249625	Timp2	Mus musculus tissue inhibitor of metalloproteinase 2 (Timp2), mRNA [NM_011594]	21858	NM_011594	tissue inhibitor of metalloproteinase 2	NM_011594	Mm.206505
A_51_P169516	0.035	up	1.3250974	Ppp1r3d	Mus musculus protein phosphatase 1, regulatory subunit 3D (Ppp1r3d), mRNA [NM_001085501]	228966	NM_001085501	protein phosphatase 1, regulatory subunit 3D	NM_001085501	Mm.472736
A_55_P1981949	0.024	up	1.3257041	Klhl5	Mus musculus kelch-like 5 (Klhl5), mRNA [NM_175174]	71778	NM_175174	kelch-like 5	NM_175174	Mm.10281
A_51_P445841	0.037	up	1.3258518	Deptor	Mus musculus DEP domain containing MTOR-interacting protein (Deptor), transcript variant 1, mRNA [NM_145470]	97998	NM_145470	DEP domain containing MTOR-interacting protein	NM_145470	Mm.295397
A_66_P140886	0.021	up	1.3262718	Prr24	Mus musculus proline rich 24 (Prr24), mRNA [NM_001136270]	66300	NM_001136270	proline rich 24	NM_001136270	Mm.218698
A_55_P2127699	0.017	up	1.326288	Pira2	Mus musculus paired-Ig-like receptor A2 (Pira2), mRNA [NM_011089]	18725	NM_011089	paired-Ig-like receptor A2	NM_011089	Mm.482470
A_55_P2076032	0.011	up	1.3263947	Cacna1f	Mus musculus adult retina cDNA, RIKEN full-length enriched library, clone:A930034B14 product:calcium channel, voltage-dependent, alpha 1F subunit, full insert sequence. [AK044695]	54652	AK044695	calcium channel, voltage-dependent, alpha 1F subunit		Mm.289647
A_55_P2031471	0.044	up	1.330586	Rufy4	Mus musculus RUN and FYVE domain containing 4 (Rufy4), transcript variant 2, mRNA [NM_001034060]	435626	NM_001034060	RUN and FYVE domain containing 4	NM_001034060	Mm.240447
A_55_P2230688	0.013	up	1.3313845	Pigm	Mus musculus phosphatidylinositol glycan anchor biosynthesis, class M (Pigm), mRNA [NM_026234]	67556	NM_026234	phosphatidylinositol glycan anchor biosynthesis, class M	NM_026234	Mm.26612
A_55_P2139753	0.018	up	1.331439	Jade2	Mus musculus jade family PHD finger 2 (Jade2), mRNA [NM_199299]	76901	NM_199299	jade family PHD finger 2	NM_199299	Mm.259996
A_55_P2047703	0.013	up	1.3317056	Serac1	Mus musculus serine active site containing 1 (Serac1), transcript variant 3, mRNA [NM_001111017]	321007	NM_001111017	serine active site containing 1	NM_001111017	Mm.5548
A_52_P586141	0.032	up	1.3333511	Adcy7	Mus musculus adenylate cyclase 7 (Adcy7), transcript variant 3, mRNA [NM_001037724]	11513	NM_001037724	adenylate cyclase 7	NM_001037724	Mm.288206
A_55_P2015782	0.048	up	1.3336397	Bcas1	Mus musculus breast carcinoma amplified sequence 1 (Bcas1), transcript variant 1, mRNA [NM_029815]	76960	NM_029815	breast carcinoma amplified sequence 1	NM_029815	Mm.240850
A_55_P2091446	0.037	up	1.3359863	Trim33	Mus musculus tripartite motif-containing 33 (Trim33), transcript variant 1, mRNA [NM_053170]	94093	NM_053170	tripartite motif-containing 33	NM_053170	Mm.195036
A_51_P331328	0.014	up	1.3359994	Gpihbp1	Mus musculus GPI-anchored HDL-binding protein 1 (Gpihbp1), mRNA [NM_026730]	68453	NM_026730	GPI-anchored HDL-binding protein 1	NM_026730	Mm.46367
A_51_P384318	0.026	up	1.3363585	C1ra	Mus musculus complement component 1, r subcomponent A (C1ra), mRNA [NM_023143]	50909	NM_023143	complement component 1, r subcomponent A	NM_023143	Mm.333375
A_55_P2107447	0.019	up	1.3367457	Rab37	Mus musculus RAB37, member of RAS oncogene family (Rab37), transcript variant 2, mRNA [NM_001163753]	58222	NM_001163753	RAB37, member of RAS oncogene family	NM_001163753	Mm.143789

A_52_P66226	0.030	up	1.3374245	Rab33a	Mus musculus RAB33A, member of RAS oncogene family (Rab33a), mRNA [NM_011228]	19337	NM_011228	RAB33A, member of RAS oncogene family	NM_011228	Mm.2015
A_55_P2259660	0.040	up	1.3378748	AK155734	Mus musculus B6-derived CD11 +ve dendritic cells cDNA, RIKEN full-length enriched library, clone:F730213H03 product:unclassifiable, full insert sequence. [AK155734]	1E+08	AK155734	cDNA sequence AK155734		Mm.446579
A_55_P2009632	0.038	up	1.3393208	S100pbp	Mus musculus 7 days neonate cerebellum cDNA, RIKEN full-length enriched library, clone:A730020N20 product:hypothetical protein, full insert sequence. [AK139097]	74648	AK139097	S100P binding protein		Mm.172064
A_66_P108143	0.009	up	1.3393509	Fbxw10	Mus musculus F-box and WD-40 domain protein 10 (Fbxw10), mRNA [NM_001033669]	213980	NM_001033669	F-box and WD-40 domain protein 10	NM_001033669	Mm.482259
A_52_P661071	0.032	up	1.3399361	Snhg3	Mus musculus small nucleolar RNA host gene (non-protein coding) 3 (Snhg3), long non-coding RNA [NR_003270]	399101	NR_003270	small nucleolar RNA host gene (non-protein coding) 3	NR_003270	Mm.486919
A_52_P206526	0.017	up	1.3400252	Gtf2ird2	Mus musculus GTF2I repeat domain containing 2 (Gtf2ird2), mRNA [NM_053266]	114674	NM_053266	GTF2I repeat domain containing 2	NM_053266	Mm.218744
A_55_P1979768	0.036	up	1.3402649	Hoxa9	Mus musculus homeobox A9 (Hoxa9), transcript variant 1, mRNA [NM_010456]	15405	NM_010456	homeobox A9	NM_010456	Mm.4694
A_55_P2058895	0.047	up	1.3407102	Fktn	Mus musculus fukutin (Fktn), mRNA [NM_139309]	246179	NM_139309	fukutin	NM_139309	Mm.247210
A_55_P2143184	0.046	up	1.3407592	Hrh4	Mus musculus histamine receptor H4 (Hrh4), mRNA [NM_153087]	225192	NM_153087	histamine receptor H4	NM_153087	Mm.207073
A_55_P2041868	0.034	up	1.3409295	Ptafr	Mus musculus platelet-activating factor receptor (Ptafr), mRNA [NM_001081211]	19204	NM_001081211	platelet-activating factor receptor	NM_001081211	Mm.89389
A_55_P2075966	0.043	up	1.3413146	Sptbn4	Mus musculus spectrin beta, non-erythrocytic 4 (Sptbn4), transcript variant sigma1, mRNA [NM_032610]	80297	NM_032610	spectrin beta, non-erythrocytic 4	NM_032610	Mm.459123
A_65_P04542	0.042	up	1.3413422	Zfp207	Mus musculus zinc finger protein 207 (Zfp207), transcript variant 5, non-coding RNA [NR_045038]	22680	NR_045038	zinc finger protein 207	NR_045038	Mm.401332
A_55_P2100065	0.019	up	1.341571	Fsd1l	Mus musculus fibronectin type III and SPRY domain containing 1-like (Fsd1l), transcript variant 2, mRNA [NM_176966]	319636	NM_176966	fibronectin type III and SPRY domain containing 1-like	NM_176966	Mm.103464
A_52_P322535	0.011	up	1.3417866	Mplkip	Mus musculus M-phase specific PLK1 intereacting protein (Mplkip), mRNA [NM_025479]	66308	NM_025479	M-phase specific PLK1 intereacting protein	NM_025479	Mm.291109
A_55_P2000628	0.024	up	1.3418795	Dusp7	Mus musculus dual specificity phosphatase 7, mRNA (cDNA clone IMAGE:4953151), partial cds. [BC025048]	235584	BC025048	dual specificity phosphatase 7		Mm.275584
A_51_P162760	0.049	up	1.3423331	Zfp623	Mus musculus zinc finger protein 623 (Zfp623), mRNA [NM_030199]	78834	NM_030199	zinc finger protein 623	NM_030199	Mm.273264
A_55_P2022293	0.010	up	1.3453954	Zfp579	Mus musculus zinc finger protein 579 (Zfp579), mRNA [NM_026741]	68490	NM_026741	zinc finger protein 579	NM_026741	Mm.76400
A_55_P1977761	0.024	up	1.3470964	Ttc14	Mus musculus tetratricopeptide repeat domain 14 (Ttc14), transcript variant 2, mRNA [NM_025978]	67120	NM_025978	tetratricopeptide repeat domain 14	NM_025978	Mm.275710
A_55_P2168301	0.013	up	1.3475684	Slc46a1	Mus musculus solute carrier family 46, member 1 (Slc46a1), mRNA [NM_026740]	52466	NM_026740	solute carrier family 46, member 1	NM_026740	Mm.131618
A_66_P134481	0.005	up	1.3479135	Skp2	Mus musculus S-phase kinase-associated protein 2 (p45) (Skp2), transcript variant 1, mRNA [NM_013787]	27401	NM_013787	S-phase kinase-associated protein 2 (p45)	NM_013787	Mm.35584
A_55_P2177944	0.044	up	1.3481293	Tmem208	transmembrane protein 208 [Source:MGI Symbol;Acc:MGI:1913570] [ENSMUST00000098453]	66320	AK131833	transmembrane protein 208		Mm.29990
A_55_P2034250	0.033	up	1.3502337	Zscan26	Mus musculus zinc finger and SCAN domain containing 26 (Zscan26), mRNA [NM_001013786]	432731	NM_001013786	zinc finger and SCAN domain containing 26	NM_001013786	Mm.441899
A_55_P2089253	0.027	up	1.3502998	Atg4c	Mus musculus autophagy related 4C, cysteine peptidase (Atg4c), transcript variant 1, mRNA [NM_175029]	242557	NM_175029	autophagy related 4C, cysteine peptidase	NM_175029	Mm.241663
A_55_P2014332	0.009	up	1.350302	Cntln	Mus musculus centlein, centrosomal protein (Cntln), transcript variant 1, mRNA [NM_175275]	338349	NM_175275	centlein, centrosomal protein	NM_175275	Mm.440562
A_51_P446315	0.007	up	1.3508314	Cdc37l1	Mus musculus cell division cycle 37-like 1 (Cdc37l1), mRNA [NM_025950]	67072	NM_025950	cell division cycle 37-like 1	NM_025950	Mm.78875
A_66_P136102	0.015	up	1.3511078	Lefty2	Mus musculus left-right determination factor 2 (Lefty2), mRNA [NM_177099]	320202	NM_177099	left-right determination factor 2	NM_177099	Mm.87078
A_51_P286826	0.044	up	1.3511536	March10	Mus musculus membrane-associated ring finger (C3HC4) 10 (March10), transcript variant 1, mRNA [NM_172568]	632687	NM_172568	membrane-associated ring finger (C3HC4) 10	NM_172568	Mm.121570
A_51_P213030	0.013	up	1.3516818	Macrod1	Mus musculus MACRO domain containing 1 (Macrod1), mRNA [NM_134147]	107227	NM_134147	MACRO domain containing 1	NM_134147	Mm.439977
A_55_P2059254	0.013	up	1.3534228	Hnrnph3	Mus musculus heterogeneous nuclear ribonucleoprotein H3 (Hnrnph3), mRNA [NM_001079824]	432467	NM_001079824	heterogeneous nuclear ribonucleoprotein H3	NM_001079824	Mm.274784
A_55_P2171802	0.019	up	1.3539598	Zfp786	Mus musculus zinc finger protein 786 (Zfp786), mRNA [NM_177882]	330301	NM_177882	zinc finger protein 786	NM_177882	Mm.128149
A_52_P306357	0.042	up	1.3560302	Prok1	Mus musculus prokineticin 1, mRNA (cDNA clone MGC:51604 IMAGE:4975079), complete cds. [BC042707]	246691	BC042707	prokineticin 1		Mm.490697
A_55_P2032714	0.034	up	1.3569219	Fhit	Mus musculus fragile histidine triad gene (Fhit), mRNA [NM_010210]	14198	NM_010210	fragile histidine triad gene	NM_010210	Mm.441926
A_55_P1956687	0.013	up	1.3571334	Rab37	Mus musculus RAB37, member of RAS oncogene family (Rab37), transcript variant 2, mRNA [NM_001163753]	58222	NM_001163753	RAB37, member of RAS oncogene family	NM_001163753	Mm.143789
A_55_P2168426	0.019	up	1.3571519	Pced1b	Mus musculus PC-esterase domain containing 1B (Pced1b), mRNA [NM_172293]	239647	NM_172293	PC-esterase domain containing 1B	NM_172293	Mm.130036
A_66_P116412	0.034	up	1.3606774	Flt3	Mus musculus FMS-like tyrosine kinase 3 (Flt3), mRNA [NM_010229]	14255	NM_010229	FMS-like tyrosine kinase 3	NM_010229	Mm.194
A_52_P671784	0.019	up	1.3606979	Adamts10	Mus musculus a disintegrin-like and metallopeptidase (reprolysin type) with thrombospondin type 1 motif, 10 (Adamts10), transcript variant 1, mRNA [NM_172619]	224697	NM_172619	a disintegrin-like and metallopeptidase (reprolysin type) with thrombospondin type 1 motif, 10	NM_172619	Mm.29304

A_55_P2037717	0.035	up	1.3613436	Slc22a18	Mus musculus solute carrier family 22 (organic cation transporter), member 18 (Slc22a18), transcript variant 2, mRNA [NM_001042760]	18400	NM_001042760	solute carrier family 22 (organic cation transporter), member 18	NM_001042760	Mm.271740
A_52_P157170	0.027	up	1.3616012	Rnf157	Mus musculus ring finger protein 157 (Rnf157), mRNA [NM_027258]	217340	NM_027258	ring finger protein 157	NM_027258	Mm.426344
A_51_P205106	0.026	up	1.3617945	Mlf1	Mus musculus myeloid leukemia factor 1 (Mlf1), transcript variant 2, mRNA [NM_010801]	17349	NM_010801	myeloid leukemia factor 1	NM_010801	Mm.10414
A_51_P490678	0.024	up	1.36337	Trim68	Mus musculus tripartite motif-containing 68 (Trim68), mRNA [NM_198012]	101700	NM_198012	tripartite motif-containing 68	NM_198012	Mm.39043
A_55_P2151685	0.009	up	1.364717	Pira11	Mus musculus paired-Ig-like receptor A11 (Pira11), mRNA [NM_011088]	18724	NM_011088	paired-Ig-like receptor A11	NM_011088	Mm.482470
A_52_P518808	0.004	up	1.3660667	Mmd	monocyte to macrophage differentiation-associated [Source:MGI Symbol;Acc:MGI:1914718] [ENSMUST00000134929]	67468	AK007961	monocyte to macrophage differentiation-associated		Mm.277518
A_52_P20727	0.021	up	1.3661752	Nhlrc1	Mus musculus NHL repeat containing 1 (Nhlrc1), mRNA [NM_175340]	105193	NM_175340	NHL repeat containing 1	NM_175340	Mm.25593
A_55_P2021099	0.026	up	1.3661953	Tmem181a	Mus musculus transmembrane protein 181A (Tmem181a), mRNA [NM_001033178]	77106	NM_001033178	transmembrane protein 181A	NM_001033178	Mm.381893
A_55_P2112464	0.038	up	1.3664732	Grm5	Mus musculus glutamate receptor, metabotropic 5 (Grm5), transcript variant b, mRNA [NM_001143834]	108071	NM_001143834	glutamate receptor, metabotropic 5	NM_001143834	Mm.235018
A_55_P2180909	0.024	up	1.3667463	Ccdc166	Mus musculus coiled-coil domain containing 166 (Ccdc166), transcript variant 2, mRNA [NM_001163518]	223648	NM_001163518	coiled-coil domain containing 166	NM_001163518	Mm.491594
A_55_P1955876	0.013	up	1.366947	Ifitm7	Mus musculus interferon induced transmembrane protein 7 (Ifitm7), mRNA [NM_001270718]	74482	NM_001270718	interferon induced transmembrane protein 7	NM_001270718	Mm.484026
A_51_P496540	0.010	up	1.3673775	Sh2d1b1	Mus musculus SH2 domain protein 1B1 (Sh2d1b1), mRNA [NM_012009]	26904	NM_012009	SH2 domain protein 1B1	NM_012009	Mm.57009
A_52_P598835	0.028	up	1.3681188	Ccdc71l	Mus musculus coiled-coil domain containing 71 like (Ccdc71l), mRNA [NM_001162903]	72123	NM_001162903	coiled-coil domain containing 71 like	NM_001162903	Mm.341352
A_55_P2049567	0.035	up	1.3682386	Pld2	Mus musculus phospholipase D2, mRNA (cDNA clone IMAGE:6310368), complete cds. [BC047268]	18806	BC047268	phospholipase D2		Mm.260177
A_51_P472621	0.039	up	1.3693562	Zfp719	Mus musculus zinc finger protein 719 (Zfp719), mRNA [NM_172482]	210105	NM_172482	zinc finger protein 719	NM_172482	Mm.234422
A_55_P2162890	0.015	up	1.3698398	Zfp780b	Mus musculus zinc finger protein 780B (Zfp780b), mRNA [NM_001081021]	338354	NM_001081021	zinc finger protein 780B	NM_001081021	Mm.389873
A_55_P1977289	0.038	up	1.3705348	Zbtb9	Mus musculus zinc finger and BTB domain containing 9 (Zbtb9), mRNA [NM_001005916]	474156	NM_001005916	zinc finger and BTB domain containing 9	NM_001005916	Mm.328890
A_51_P261999	0.014	up	1.3711456	Ccdc166	Mus musculus coiled-coil domain containing 166 (Ccdc166), transcript variant 2, mRNA [NM_001163518]	223648	NM_001163518	coiled-coil domain containing 166	NM_001163518	Mm.491594
A_51_P120875	0.044	up	1.3712348	Olfr713	Mus musculus olfactory receptor 713 (Olfr713), mRNA [NM_147034]	259036	NM_147034	olfactory receptor 713	NM_147034	Mm.377801
A_52_P72434	0.008	up	1.3713276	Khk	Mus musculus ketohexokinase (Khk), mRNA [NM_008439]	16548	NM_008439	ketohexokinase	NM_008439	Mm.22451
A_51_P293069	0.009	up	1.3715903	Mfsd7b	Mus musculus major facilitator superfamily domain containing 7B (Mfsd7b), mRNA [NM_001081259]	226844	NM_001081259	major facilitator superfamily domain containing 7B	NM_001081259	Mm.134053
A_65_P08864	0.003	up	1.373025	Dph5	Mus musculus 4 days neonate thymus cDNA, RIKEN full-length enriched library, clone:B630008G01 product:Weakly similar to putative diphthine synthase, full insert sequence. [AK046753]	69740	AK046753	DPH5 homolog (S. cerevisiae)		Mm.5915
A_55_P2075070	0.048	up	1.3734726	S1pr5	Mus musculus sphingosine-1-phosphate receptor 5 (S1pr5), mRNA [NM_053190]	94226	NM_053190	sphingosine-1-phosphate receptor 5	NM_053190	Mm.190619
A_55_P1953788	0.043	up	1.3736069	Itk	Mus musculus IL2 inducible T cell kinase (Itk), transcript variant 1, mRNA [NM_001281965]	16428	NM_001281965	IL2 inducible T cell kinase	NM_001281965	Mm.339927
A_51_P396917	0.027	up	1.3741325	Zfyve21	Mus musculus zinc finger, FYVE domain containing 21 (Zfyve21), mRNA [NM_026752]	68520	NM_026752	zinc finger, FYVE domain containing 21	NM_026752	Mm.390497
A_52_P372092	0.045	up	1.3744627	Zfp119a	Mus musculus zinc finger protein 119a (Zfp119a), mRNA [NM_144546]	104349	NM_144546	zinc finger protein 119a	NM_144546	Mm.460659
A_52_P541270	0.017	up	1.3747127	Crebl2	Mus musculus cAMP responsive element binding protein-like 2 (Crebl2), mRNA [NM_177687]	232430	NM_177687	cAMP responsive element binding protein-like 2	NM_177687	Mm.267010
A_51_P343252	0.006	up	1.3750653	Cd151	Mus musculus CD151 antigen (Cd151), transcript variant 1, mRNA [NM_009842]	12476	NM_009842	CD151 antigen	NM_009842	Mm.30246
A_52_P456977	0.002	up	1.3755871	Deaf1	Mus musculus deformed epidermal autoregulatory factor 1 (Drosophila) (Deaf1), transcript variant 1, mRNA [NM_016874]	54006	NM_016874	deformed epidermal autoregulatory factor 1 (Drosophila)	NM_016874	Mm.28392
A_52_P186962	0.031	up	1.3764392	Dennd6a	Mus musculus DENN/MADD domain containing 6A (Dennd6a), transcript variant 2, mRNA [NM_145969]	211922	NM_145969	DENN/MADD domain containing 6A	NM_145969	Mm.260512
A_55_P2006327	0.041	up	1.3779572	Ptprg	Mus musculus protein tyrosine phosphatase, receptor type, G (Ptprg), mRNA [NM_008981]	19270	NM_008981	protein tyrosine phosphatase, receptor type, G	NM_008981	Mm.431266
A_55_P1974827	0.003	up	1.3788881	Pde1b	Mus musculus phosphodiesterase 1B, Ca2+-calmodulin dependent (Pde1b), transcript variant 1, mRNA [NM_008800]	18574	NM_008800	phosphodiesterase 1B, Ca2+-calmodulin dependent	NM_008800	Mm.390792
A_55_P2107972	0.045	up	1.3800664	Smad5	Mus musculus SMAD family member 5 (Smad5), transcript variant 2, mRNA [NM_001164041]	17129	NM_001164041	SMAD family member 5	NM_001164041	Mm.272920
A_51_P512627	0.007	up	1.3805577	Syngr4	Mus musculus synaptogyrin 4 (Syngr4), mRNA [NM_021482]	58867	NM_021482	synaptogyrin 4	NM_021482	Mm.214217
A_55_P2107957	0.022	up	1.3806219	Fam175a	Mus musculus family with sequence similarity 175, member A (Fam175a), mRNA [NM_172405]	70681	NM_172405	family with sequence similarity 175, member A	NM_172405	Mm.486560
A_52_P655285	0.041	up	1.3814371	Zfp462	Mus musculus zinc finger protein 462 (Zfp462), mRNA [NM_172867]	242466	NM_172867	zinc finger protein 462	NM_172867	Mm.102904
A_55_P2068891	0.028	up	1.3817558	Pear1	Mus musculus platelet endothelial aggregation receptor 1 (Pear1), transcript variant 1, mRNA [NM_028460]	73182	NM_028460	platelet endothelial aggregation receptor 1	NM_028460	Mm.158962
A_55_P2125684	0.036	up	1.3820145	Camkk2	Mus musculus calcium/calmodulin-dependent protein kinase kinase 2, beta (Camkk2), transcript variant 2, mRNA [NM_145358]	207565	NM_145358	calcium/calmodulin-dependent protein kinase kinase 2, beta	NM_145358	Mm.289237
A_51_P440210	0.012	up	1.3821106	Prkg1	Mus musculus protein kinase, cGMP-dependent, type I (Prkg1), transcript variant 2, mRNA [NM_011160]	19091	NM_011160	protein kinase, cGMP-dependent, type I	NM_011160	Mm.381170

A_55_P2009767	0.050	up	1.3825997	Mapkapk3	mitogen-activated protein kinase-activated protein kinase 3 [Source:MGI Symbol;Acc:MGI:2143163] [ENSMUST00000141596]	102626	AK078977	mitogen-activated protein kinase-activated protein kinase 3		Mm.445242
A_51_P292073	0.037	up	1.3843076	Haghl	Mus musculus hydroxyacylglutathione hydrolase-like (Haghl), transcript variant 2, mRNA [NM_026897]	68977	NM_026897	hydroxyacylglutathione hydrolase-like	NM_026897	Mm.29230
A_52_P516097	0.005	up	1.3843096	Leng9	Mus musculus leukocyte receptor cluster (LRC) member 9 (Leng9), mRNA [NM_175529]	243813	NM_175529	leukocyte receptor cluster (LRC) member 9	NM_175529	Mm.347643
A_55_P2118799	0.007	up	1.38575	Rmi1	Mus musculus RMI1, RecQ mediated genome instability 1, homolog (S. cerevisiae) (Rmi1), transcript variant 1, mRNA [NM_001168248]	74386	NM_001168248	RMI1, RecQ mediated genome instability 1, homolog (S. cerevisiae)	NM_001168248	Mm.85289
A_55_P2116315	0.029	up	1.3857714	Trim45	Mus musculus tripartite motif-containing 45 (Trim45), transcript variant 1, mRNA [NM_001165953]	229644	NM_001165953	tripartite motif-containing 45	NM_001165953	Mm.189351
A_55_P2040893	0.047	up	1.3858085	Tnni2	Mus musculus troponin I, skeletal, fast 2 (Tnni2), mRNA [NM_009405]	21953	NM_009405	troponin I, skeletal, fast 2	NM_009405	Mm.39469
A_55_P2149209	0.034	up	1.3861519	Camk2a	Mus musculus calcium/calmodulin-dependent protein kinase II alpha (Camk2a), transcript variant 1, mRNA [NM_009792]	12322	NM_009792	calcium/calmodulin-dependent protein kinase II alpha	NM_009792	Mm.131530
A_55_P2011573	0.038	up	1.386352	F2rl3	Mus musculus coagulation factor II (thrombin) receptor-like 3 (F2rl3), mRNA [NM_007975]	14065	NM_007975	coagulation factor II (thrombin) receptor-like 3	NM_007975	Mm.12948
A_51_P391159	0.033	up	1.3869207	Ang	Mus musculus angiogenin, ribonuclease, RNase A family, 5 (Ang), transcript variant 1, mRNA [NM_007447]	11727	NM_007447	angiogenin, ribonuclease, RNase A family, 5	NM_007447	Mm.202665
A_51_P418901	0.036	up	1.3875234	Prss2	Mus musculus protease, serine, 2 (Prss2), mRNA [NM_009430]	22072	NM_009430	protease, serine, 2	NM_009430	Mm.276926
A_66_P104046	0.019	up	1.3880926	Tdp1	Mus musculus adult male epididymis cDNA, RIKEN full-length enriched library, clone:9230109C12 product:hypothetical protein, full insert sequence. [AK079006]	104884	AK079006	tyrosyl-DNA phosphodiesterase 1		Mm.487464
A_55_P2012799	0.046	up	1.3881418	Rtkn	Mus musculus rhotekin (Rtkn), transcript variant 3, mRNA [NM_133641]	20166	NM_133641	rhotekin	NM_133641	Mm.4139
A_55_P1976574	0.008	up	1.3885628	Tmod4	Mus musculus tropomodulin 4 (Tmod4), mRNA [NM_016712]	50874	NM_016712	tropomodulin 4	NM_016712	Mm.71935
A_52_P595717	0.037	up	1.3892868	Smug1	Mus musculus single-strand selective monofunctional uracil DNA glycosylase (Smug1), mRNA [NM_027885]	71726	NM_027885	single-strand selective monofunctional uracil DNA glycosylase	NM_027885	Mm.254820
A_51_P410918	0.036	up	1.3906065	Tor4a	Mus musculus torsin family 4, member A (Tor4a), mRNA [NM_146115]	227612	NM_146115	torsin family 4, member A	NM_146115	Mm.119747
A_66_P115072	0.032	up	1.3906305	Prrt4	Mus musculus proline-rich transmembrane protein 4 (Prrt4), mRNA [NM_001101443]	101359	NM_001101443	proline-rich transmembrane protein 4	NM_001101443	Mm.22818
A_66_P109577	0.015	up	1.3911572	Plcb3	Mus musculus phospholipase C, beta 3 (Plcb3), mRNA [NM_008874]	18797	NM_008874	phospholipase C, beta 3	NM_008874	Mm.273204
A_55_P2016466	0.002	up	1.3922658	Rccd1	Mus musculus RCC1 domain containing 1 (Rccd1), mRNA [NM_173445]	269955	NM_173445	RCC1 domain containing 1	NM_173445	Mm.474502
A_55_P2134004	0.019	up	1.3922914	Gstm2	Mus musculus glutathione S-transferase, mu 2 (Gstm2), mRNA [NM_008183]	14863	NM_008183	glutathione S-transferase, mu 2	NM_008183	Mm.440086
A_55_P2110435	0.050	up	1.3930485	Zscan20	Mus musculus zinc finger and SCAN domains 20 (Zscan20), mRNA [NM_177758]	269585	NM_177758	zinc finger and SCAN domains 20	NM_177758	Mm.153291
A_55_P2178084	0.023	up	1.3947245	Dnmt3a	Mus musculus DNA methyltransferase 3A (Dnmt3a), transcript variant 1, mRNA [NM_007872]	13435	NM_007872	DNA methyltransferase 3A	NM_007872	Mm.5001
A_55_P2135551	0.037	up	1.3951547	Dnase1l2	Mus musculus deoxyribonuclease 1-like 2 (Dnase1l2), mRNA [NM_025718]	66705	NM_025718	deoxyribonuclease 1-like 2	NM_025718	Mm.29017
A_51_P479132	0.042	up	1.3952351	Zcchc3	Mus musculus zinc finger, CCHC domain containing 3 (Zcchc3), mRNA [NM_175126]	67917	NM_175126	zinc finger, CCHC domain containing 3	NM_175126	Mm.391810
A_51_P204103	0.032	up	1.3957094	Olfr1442	Mus musculus olfactory receptor 1442 (Olfr1442), mRNA [NM_146697]	258692	NM_146697	olfactory receptor 1442	NM_146697	Mm.223127
A_55_P1988899	0.011	up	1.3960221	Mblac2	Mus musculus metallo-beta-lactamase domain containing 2 (Mblac2), mRNA [NM_028372]	72852	NM_028372	metallo-beta-lactamase domain containing 2	NM_028372	Mm.31095
A_52_P281033	0.031	up	1.3960584	Socs5	Mus musculus suppressor of cytokine signaling 5 (Socs5), mRNA [NM_019654]	56468	NM_019654	suppressor of cytokine signaling 5	NM_019654	Mm.126885
A_51_P136355	0.016	up	1.3977334	Gng11	Mus musculus guanine nucleotide binding protein (G protein), gamma 11 (Gng11), mRNA [NM_025331]	66066	NM_025331	guanine nucleotide binding protein (G protein), gamma 11	NM_025331	Mm.25547
A_51_P202801	0.025	up	1.3986849	Abcb9	Mus musculus ATP-binding cassette, sub-family B (MDR/TAP), member 9 (Abcb9), mRNA [NM_019875]	56325	NM_019875	ATP-binding cassette, sub-family B (MDR/TAP), member 9	NM_019875	Mm.254839
A_51_P503654	0.023	up	1.3990048	Tmem205	Mus musculus transmembrane protein 205 (Tmem205), transcript variant 2, mRNA [NM_178577]	235043	NM_178577	transmembrane protein 205	NM_178577	Mm.489656
A_52_P15490	0.037	up	1.399932	Pvrl3	Mus musculus poliovirus receptor-related 3 (Pvrl3), transcript variant beta, mRNA [NM_021496]	58998	NM_021496	poliovirus receptor-related 3	NM_021496	Mm.328072
A_55_P2099540	0.003	up	1.4006379	H2afj	Mus musculus H2A histone family, member J (H2afj), mRNA [NM_177688]	232440	NM_177688	H2A histone family, member J	NM_177688	Mm.154564
A_51_P259296	0.030	up	1.4009163	Lpl	Mus musculus lipoprotein lipase (Lpl), mRNA [NM_008509]	16956	NM_008509	lipoprotein lipase	NM_008509	Mm.1514
A_52_P655136	0.017	up	1.401162	Nlrc4	Mus musculus NLR family, CARD domain containing 4 (Nlrc4), mRNA [NM_001033367]	268973	NM_001033367	NLR family, CARD domain containing 4	NM_001033367	Mm.311884
A_52_P154026	0.021	up	1.4014523	Zcwpw1	Mus musculus zinc finger, CW type with PWWP domain 1 (Zcwpw1), mRNA [NM_001005426]	381678	NM_001005426	zinc finger, CW type with PWWP domain 1	NM_001005426	Mm.332765
A_55_P2075485	0.039	up	1.4040363	Ibsp	Mus musculus integrin binding sialoprotein (Ibsp), mRNA [NM_008318]	15891	NM_008318	integrin binding sialoprotein	NM_008318	Mm.4987

A_51_P429865	0.004	up	1.4048287	Msh5	Mus musculus mutS homolog 5 (E. coli), mRNA (cDNA clone MGC:176074 IMAGE:9055725), complete cds. [BC141113]	17687	BC141113	mutS homolog 5 (E. coli)		Mm.24192
A_55_P2010008	0.029	up	1.4053822	Zfp113	Mus musculus zinc finger protein 113 (Zfp113), mRNA [NM_019747]	56314	NM_019747	zinc finger protein 113	NM_019747	Mm.19274
A_55_P2043932	0.018	up	1.4058243	Tmem8b	Mus musculus transmembrane protein 8B (Tmem8b), mRNA [NM_001085508]	242409	NM_001085508	transmembrane protein 8B	NM_001085508	Mm.171489
A_51_P372702	0.022	up	1.4062428	Il16	Mus musculus interleukin 16 (Il16), mRNA [NM_010551]	16170	NM_010551	interleukin 16	NM_010551	Mm.10137
A_51_P490955	0.004	up	1.4075956	Zfp784	Mus musculus zinc finger protein 784 (Zfp784), mRNA [NM_001039532]	654801	NM_001039532	zinc finger protein 784	NM_001039532	Mm.333548
A_55_P2018904	0.035	up	1.4088308	Zbtbd6	Mus musculus kelch repeat and BTB (POZ) domain containing 6 (Zbtbd6), mRNA [NM_001034882]	432879	NM_001034882	kelch repeat and BTB (POZ) domain containing 6	NM_001034882	Mm.308666
A_55_P2069974	0.009	up	1.4091601	Kctd1	Mus musculus potassium channel tetramerisation domain containing 1 (Kctd1), transcript variant 1, mRNA [NM_001142731]	106931	NM_001142731	potassium channel tetramerisation domain containing 1	NM_001142731	Mm.329299
A_51_P430929	0.026	up	1.4105896	Fam20a	Mus musculus family with sequence similarity 20, member A (Fam20a), mRNA [NM_153782]	208659	NM_153782	family with sequence similarity 20, member A	NM_153782	Mm.208662
A_55_P2156425	0.031	up	1.4112008	Upk1a	Mus musculus uroplakin 1A (Upk1a), mRNA [NM_026815]	109637	NM_026815	uroplakin 1A	NM_026815	Mm.25471
A_55_P2345631	0.016	up	1.4119678	Tcrg-V1	Mus musculus 16 days neonate thymus cDNA, RIKEN full-length enriched library, clone:A130095M15 product:unclassifiable, full insert sequence. [AK038325]	21632	AK038325	T cell receptor gamma, variable 1		Mm.210785
A_55_P1984846	0.049	up	1.4125847	Zbtb1	zinc finger and BTB domain containing 1 [Source:MGI Symbol;Acc:MGI:2442326] [ENSMUST00000042779]	268564	BC029005	zinc finger and BTB domain containing 1		Mm.470000
A_55_P2093163	0.016	up	1.412745	Zbtb14	zinc finger and BTB domain containing 14 [Source:MGI Symbol;Acc:MGI:1195345] [ENSMUST00000112674]	22666	AK082051	zinc finger and BTB domain containing 14		Mm.29434
A_55_P2159565	0.026	up	1.4132528	Tas2r131	Mus musculus taste receptor, type 2, member 131 (Tas2r131), mRNA [NM_207030]	387356	NM_207030	taste receptor, type 2, member 131	NM_207030	Mm.377917
A_52_P305279	0.009	up	1.413579	Spata13	Mus musculus spermatogenesis associated 13 (Spata13), mRNA [NM_001033272]	219140	NM_001033272	spermatogenesis associated 13	NM_001033272	Mm.149776
A_51_P392385	0.035	up	1.4143063	Smcp	Mus musculus sperm mitochondria-associated cysteine-rich protein (Smcp), mRNA [NM_008574]	17235	NM_008574	sperm mitochondria-associated cysteine-rich protein	NM_008574	Mm.331192
A_55_P1988488	0.040	up	1.416088	Npc1l1	Mus musculus NPC1-like 1 (Npc1l1), mRNA [NM_207242]	237636	NM_207242	NPC1-like 1	NM_207242	Mm.212492
A_51_P171999	0.012	up	1.4163628	Apoe	Mus musculus apolipoprotein E (Apoe), mRNA [NM_009696]	11816	NM_009696	apolipoprotein E	NM_009696	Mm.305152
A_55_P2363442	0.037	up	1.4163666	Zfp867	Mus musculus zinc finger protein 867 (Zfp867), mRNA [NM_178417]	237775	NM_178417	zinc finger protein 867	NM_178417	Mm.339604
A_51_P364871	0.048	up	1.41665	Olfr361	Mus musculus olfactory receptor 361 (Olfr361), mRNA [NM_146368]	258365	NM_146368	olfactory receptor 361	NM_146368	Mm.377459
A_51_P281089	0.021	up	1.4173571	S100a6	Mus musculus S100 calcium binding protein A6 (calcyclin) (S100a6), mRNA [NM_011313]	20200	NM_011313	S100 calcium binding protein A6 (calcyclin)	NM_011313	Mm.100144
A_55_P2079579	0.004	up	1.4176493	Pira7	Mus musculus paired-Ig-like receptor A7 (Pira7), mRNA [NM_011094]	18730	NM_011094	paired-Ig-like receptor A7	NM_011094	Mm.482470
A_55_P2057777	0.030	up	1.4176927	Fgfr1	Mus musculus fibroblast growth factor receptor 1 (Fgfr1), transcript variant 1, mRNA [NM_010206]	14182	NM_010206	fibroblast growth factor receptor 1	NM_010206	Mm.265716
A_55_P2054409	0.002	up	1.4180219	Pira2	Mus musculus paired-Ig-like receptor A2 (Pira2), mRNA [NM_011089]	18725	NM_011089	paired-Ig-like receptor A2	NM_011089	Mm.482470
A_52_P588771	0.035	up	1.418211	Ntn5	Mus musculus netrin 5 (Ntn5), mRNA [NM_001033356]	243967	NM_001033356	netrin 5	NM_001033356	Mm.291240
A_52_P233441	0.049	up	1.4194889	Gata2	Mus musculus GATA binding protein 2 (Gata2), mRNA [NM_008090]	14461	NM_008090	GATA binding protein 2	NM_008090	Mm.491156
A_51_P342786	0.034	up	1.4199414	Mlh3	Mus musculus mutL homolog 3 (E coli) (Mlh3), mRNA [NM_175337]	217716	NM_175337	mutL homolog 3 (E coli)	NM_175337	Mm.311981
A_55_P2430472	0.007	up	1.4207133	Dido1	Mus musculus death inducer-obliterator 1 (Dido1), transcript variant 1, mRNA [NM_011805]	23856	NM_011805	death inducer-obliterator 1	NM_011805	Mm.253836
A_55_P2032147	0.021	up	1.4208117	Wnt9a	Mus musculus wingless-type MMTV integration site 9A (Wnt9a), mRNA [NM_139298]	216795	NM_139298	wingless-type MMTV integration site 9A	NM_139298	Mm.218794
A_55_P2184339	0.045	up	1.4208729	Mdga1	Mus musculus MAM domain containing glycosylphosphatidylinositol anchor 1 (Mdga1), mRNA [NM_001081160]	74762	NM_001081160	MAM domain containing glycosylphosphatidylinositol anchor 1	NM_001081160	Mm.40901
A_55_P2023146	0.029	up	1.4220188	Nlrp1a	Mus musculus NLR family, pyrin domain containing 1A (Nlrp1a), mRNA [NM_001004142]	195046	NM_001004142	NLR family, pyrin domain containing 1A	NM_001004142	Mm.240227
A_55_P2017590	0.032	up	1.4226778	Exog	Mus musculus endo/exonuclease (5'-3'), endonuclease G-like (Exog), transcript variant 1, mRNA [NM_001172136]	208194	NM_001172136	endo/exonuclease (5'-3'), endonuclease G-like	NM_001172136	Mm.101578
A_55_P2162530	0.007	up	1.4229659	Pex6	Mus musculus peroxisomal biogenesis factor 6 (Pex6), mRNA [NM_145488]	224824	NM_145488	peroxisomal biogenesis factor 6	NM_145488	Mm.299399
A_55_P2151225	0.046	up	1.4232575	Krtap16-1	Mus musculus keratin associated protein 16-1 (Krtap16-1), mRNA [NM_130870]	170651	NM_130870	keratin associated protein 16-1	NM_130870	Mm.196827
A_55_P2059159	0.034	up	1.4234608	Zfp111	Mus musculus zinc finger protein 111 (Zfp111), mRNA [NM_019940]	56707	NM_019940	zinc finger protein 111	NM_019940	Mm.448367
A_66_P123683	0.013	up	1.4234824	Nabp1	Mus musculus nucleic acid binding protein 1 (Nabp1), mRNA [NM_028696]	109019	NM_028696	nucleic acid binding protein 1	NM_028696	Mm.196290
A_55_P2113439	0.045	up	1.4235779	Caln1	Mus musculus calneuron 1 (Caln1), transcript variant 2, mRNA [NM_021371]	140904	NM_021371	calneuron 1	NM_021371	Mm.320206
A_55_P2006615	0.002	up	1.42386	Rbbp9	Mus musculus retinoblastoma binding protein 9 (Rbbp9), mRNA [NM_015754]	26450	NM_015754	retinoblastoma binding protein 9	NM_015754	Mm.440564

A_55_P2122718	0.005	up	1.4249376	Gpr19	Mus musculus G protein-coupled receptor 19 (Gpr19), transcript variant 2, mRNA [NM_001167700]	14760	NM_001167700	G protein-coupled receptor 19	NM_001167700	Mm.4787
A_55_P1959031	0.047	up	1.4250857	Nkain2	Mus musculus Na+/K+ transporting ATPase interacting 2 (Nkain2), transcript variant 2, mRNA [NM_001025286]	432450	NM_001025286	Na+/K+ transporting ATPase interacting 2	NM_001025286	Mm.49665
A_66_P119034	0.009	up	1.4253234	Pla2g7	Mus musculus phospholipase A2, group VII (platelet-activating factor acetylhydrolase, plasma) (Pla2g7), mRNA [NM_013737]	27226	NM_013737	phospholipase A2, group VII (platelet-activating factor acetylhydrolase, plasma)	NM_013737	Mm.9277
A_55_P2038437	0.020	up	1.4272964	Dnah17	Mus musculus dynein, axonemal, heavy chain 17 (Dnah17), mRNA [NM_001167746]	69926	NM_001167746	dynein, axonemal, heavy chain 17	NM_001167746	Mm.473893
A_55_P2112882	0.024	up	1.4307362	Adh6a	Mus musculus alcohol dehydrogenase 6A (class V) (Adh6a), mRNA [NM_026945]	69117	NM_026945	alcohol dehydrogenase 6A (class V)	NM_026945	Mm.46265
A_55_P2083629	0.018	up	1.4309742	Tle2	Mus musculus transducin-like enhancer of split 2, homolog of Drosophila E(spl) (Tle2), transcript variant 2, mRNA [NM_001252401]	21886	NM_001252401	transducin-like enhancer of split 2, homolog of Drosophila E(spl)	NM_001252401	Mm.38608
A_55_P2180769	0.005	up	1.4321121	Gls	Mus musculus glutaminase (Gls), transcript variant 1, mRNA [NM_001081081]	14660	NM_001081081	glutaminase	NM_001081081	Mm.398608
A_52_P172910	0.041	up	1.4326404	Serpinb12	Mus musculus serine (or cysteine) peptidase inhibitor, clade B (ovalbumin), member 12 (Serpinb12), transcript variant 1, mRNA [NM_027971]	71869	NM_027971	serine (or cysteine) peptidase inhibitor, clade B (ovalbumin), member 12	NM_027971	Mm.321740
A_55_P1954116	0.025	up	1.4329189	C8b	Mus musculus complement component 8, beta polypeptide (C8b), mRNA [NM_133882]	110382	NM_133882	complement component 8, beta polypeptide	NM_133882	Mm.87072
A_55_P2003798	0.039	up	1.4342017	Olfr1364	Mus musculus olfactory receptor 1364 (Olfr1364), mRNA [NM_146540]	258533	NM_146540	olfactory receptor 1364	NM_146540	Mm.271445
A_51_P438083	0.012	up	1.4353435	Slc6a13	Mus musculus solute carrier family 6 (neurotransmitter transporter, GABA), member 13 (Slc6a13), mRNA [NM_144512]	14412	NM_144512	solute carrier family 6 (neurotransmitter transporter, GABA), member 13	NM_144512	Mm.258596
A_55_P2140941	0.030	up	1.4354719	Tinagl1	Mus musculus tubulointerstitial nephritis antigen-like 1 (Tinagl1), transcript variant 1, mRNA [NM_023476]	94242	NM_023476	tubulointerstitial nephritis antigen-like 1	NM_023476	Mm.15801
A_51_P296429	0.012	up	1.4357312	Tmem170	Mus musculus transmembrane protein 170 (Tmem170), mRNA [NM_025781]	66817	NM_025781	transmembrane protein 170	NM_025781	Mm.345350
A_55_P2294037	0.025	up	1.4360185	Pura	purine rich element binding protein A [Source:MGI Symbol;Acc:MGI:103079] [ENSMUST00000051301]	19290	AK018157	purine rich element binding protein A		Mm.404138
A_55_P1960989	0.038	up	1.4376222	Nlgn2	Mus musculus neuroligin 2 (Nlgn2), mRNA [NM_198862]	216856	NM_198862	neuroligin 2	NM_198862	Mm.151293
A_52_P473106	0.010	up	1.4380469	Otos	Mus musculus otospiralin (Otos), mRNA [NM_153114]	260301	NM_153114	otospiralin	NM_153114	Mm.219545
A_51_P363801	0.035	up	1.4385947	Pgpep1	Mus musculus pyroglutamyl-peptidase I (Pgpep1), mRNA [NM_023217]	66522	NM_023217	pyroglutamyl-peptidase I	NM_023217	Mm.154906
A_55_P2093705	0.045	up	1.4389411	Meig1	Mus musculus meiosis expressed gene 1 (Meig1), mRNA [NM_008579]	104362	NM_008579	meiosis expressed gene 1	NM_008579	Mm.2688
A_55_P2004876	0.029	up	1.4394647	Srpx2	Mus musculus sushi-repeat-containing protein, X-linked 2 (Srpx2), transcript variant 1, mRNA [NM_026838]	68792	NM_026838	sushi-repeat-containing protein, X-linked 2	NM_026838	Mm.263553
A_55_P2010161	0.006	up	1.4397779	Fastkd5	Mus musculus FAST kinase domains 5 (Fastkd5), transcript variant 1, mRNA [NM_198176]	380601	NM_198176	FAST kinase domains 5	NM_198176	Mm.27090
A_55_P2017075	0.020	up	1.439986	Zfp54	Mus musculus zinc finger protein 54 (Zfp54), mRNA [NM_011760]	22712	NM_011760	zinc finger protein 54	NM_011760	Mm.12916
A_51_P516133	0.026	up	1.4415935	Hist1h1c	Mus musculus histone cluster 1, H1c (Hist1h1c), mRNA [NM_015786]	50708	NM_015786	histone cluster 1, H1c	NM_015786	Mm.193539
A_55_P2111483	0.034	up	1.4416895	Polq	polymerase (DNA directed), theta [Source:MGI Symbol;Acc:MGI:2155399] [ENSMUST00000182163]	77782	AK020790	polymerase (DNA directed), theta		Mm.259114
A_55_P1956284	0.045	up	1.4443433	Zfp60	zinc finger protein 60 [Source:MGI Symbol;Acc:MGI:99207] [ENSMUST00000108336]	22718	AK018216	zinc finger protein 60		Mm.348021
A_52_P114260	0.018	up	1.444602	C1rb	Mus musculus complement component 1, r subcomponent B (C1rb), mRNA [NM_001113356]	667277	NM_001113356	complement component 1, r subcomponent B	NM_001113356	Mm.481484
A_52_P628580	0.046	up	1.4454406	Ggt7	Mus musculus gamma-glutamyltransferase 7 (Ggt7), mRNA [NM_144786]	207182	NM_144786	gamma-glutamyltransferase 7	NM_144786	Mm.41757
A_55_P1975510	0.022	up	1.4470097	H6pd	Mus musculus hexose-6-phosphate dehydrogenase (glucose 1-dehydrogenase) (H6pd), mRNA [NM_173371]	100198	NM_173371	hexose-6-phosphate dehydrogenase (glucose 1-dehydrogenase)	NM_173371	Mm.22183
A_52_P344290	0.041	up	1.4477677	F2r	Mus musculus coagulation factor II (thrombin) receptor (F2r), mRNA [NM_010169]	14062	NM_010169	coagulation factor II (thrombin) receptor	NM_010169	Mm.24816
A_55_P2123912	0.024	up	1.4481014	Tmem200b	Mus musculus transmembrane protein 200B (Tmem200b), mRNA [NM_001201367]	623230	NM_001201367	transmembrane protein 200B	NM_001201367	Mm.421179
A_55_P2077048	0.020	up	1.449842	Itih5	Mus musculus inter-alpha (globulin) inhibitor H5 (Itih5), mRNA [NM_172471]	209378	NM_172471	inter-alpha (globulin) inhibitor H5	NM_172471	Mm.313876
A_52_P418489	0.028	up	1.4515347	Tinagl1	Mus musculus tubulointerstitial nephritis antigen-like 1 (Tinagl1), transcript variant 1, mRNA [NM_023476]	94242	NM_023476	tubulointerstitial nephritis antigen-like 1	NM_023476	Mm.15801
A_55_P1955305	0.011	up	1.4516443	Sirpb1a	Mus musculus signal-regulatory protein beta 1A (Sirpb1a), transcript variant 3, mRNA [NM_001002898]	320832	NM_001002898	signal-regulatory protein beta 1A	NM_001002898	Mm.425126
A_55_P2004016	0.008	up	1.4517514	Crispld2	Mus musculus cysteine-rich secretory protein LCCL domain containing 2 (Crispld2), mRNA [NM_030209]	78892	NM_030209	cysteine-rich secretory protein LCCL domain containing 2	NM_030209	Mm.264680
A_51_P168395	0.002	up	1.4544139	Ttc30a1	Mus musculus tetratricopeptide repeat domain 30A1 (Ttc30a1), mRNA [NM_030188]	78802	NM_030188	tetratricopeptide repeat domain 30A1	NM_030188	Mm.282339
A_66_P133255	0.015	up	1.4552053	Prkch	Mus musculus 10 days neonate skin cDNA, RIKEN full-length enriched library, clone:4732457K18 product:protein kinase C, eta, full insert sequence. [AK076352]	18755	AK076352	protein kinase C, eta		

A_52_P471395	0.006	up	1.4562353	Ifngr2	Mus musculus interferon gamma receptor 2 (Ifngr2), mRNA [NM_008338]	15980	NM_008338	interferon gamma receptor 2	NM_008338	Mm.249364
A_55_P2168732	0.027	up	1.4579287	Polr3e	Mus musculus polymerase (RNA) III (DNA directed) polypeptide E (Polr3e), transcript variant 1, mRNA [NM_025298]	26939	NM_025298	polymerase (RNA) III (DNA directed) polypeptide E	NM_025298	Mm.193632
A_66_P100578	0.042	up	1.458018	Vrtn	Mus musculus vertebrae development associated (Vrtn), transcript variant 1, mRNA [NM_001168588]	432677	NM_001168588	vertebrae development associated	NM_001168588	Mm.311913
A_51_P263591	0.039	up	1.4584277	Pank1	Mus musculus pantothenate kinase 1 (Pank1), transcript variant 2, mRNA [NM_023792]	75735	NM_023792	pantothenate kinase 1	NM_023792	Mm.24742
A_51_P445473	0.011	up	1.4585396	Fut7	Mus musculus fucosyltransferase 7 (Fut7), transcript variant 1, mRNA [NM_013524]	14347	NM_013524	fucosyltransferase 7	NM_013524	Mm.1203
A_55_P2094352	0.049	up	1.460683	Zfp523	Mus musculus NOD-derived CD11c +ve dendritic cells cDNA, RIKEN full-length enriched library, clone:F630106K16 product:zinc finger protein 523, full insert sequence. [AK170523]	224656	AK170523	zinc finger protein 523		Mm.267359
A_51_P415220	0.021	up	1.4611721	Zmat3	Mus musculus zinc finger matrin type 3 (Zmat3), mRNA [NM_009517]	22401	NM_009517	zinc finger matrin type 3	NM_009517	Mm.35705
A_55_P2177578	0.024	up	1.4633515	Atp6v0a1	ATPase, H+ transporting, lysosomal V0 subunit A1 [Source:MGI Symbol;Acc:MGI:103286] [ENSMUST00000139857]	11975	AK166356	ATPase, H+ transporting, lysosomal V0 subunit A1		Mm.340818
A_55_P2087607	0.042	up	1.4634775	Cp	Mus musculus ceruloplasmin (Cp), transcript variant 3, mRNA [NM_001276248]	12870	NM_001276248	ceruloplasmin	NM_001276248	Mm.13787
A_51_P184969	0.014	up	1.4650583	Rilpl1	Mus musculus Rab interacting lysosomal protein-like 1 (Rilpl1), mRNA [NM_021430]	75695	NM_021430	Rab interacting lysosomal protein-like 1	NM_021430	Mm.41180
A_55_P2182675	0.020	up	1.4654614	Fut7	Mus musculus fucosyltransferase 7 (Fut7), transcript variant 1, mRNA [NM_013524]	14347	NM_013524	fucosyltransferase 7	NM_013524	Mm.1203
A_55_P2009077	0.005	up	1.4655515	Sybu	Mus musculus syntabulin (syntaxin-interacting) (Sybu), transcript variant b, mRNA [NM_001032727]	319613	NM_001032727	syntabulin (syntaxin-interacting)	NM_001032727	Mm.207715
A_55_P2048096	0.031	up	1.465821	Mzf1	Mus musculus myeloid zinc finger 1 (Mzf1), mRNA [NM_145819]	109889	NM_145819	myeloid zinc finger 1	NM_145819	Mm.10103
A_55_P1991750	0.029	up	1.4682871	Mtcp1	mature T cell proliferation 1 [Source:MGI Symbol;Acc:MGI:102699] [ENSMUST00000120286]	17763		mature T cell proliferation 1		
A_51_P143190	0.008	up	1.4694821	Lyl1	Mus musculus lymphoblastomic leukemia 1 (Lyl1), mRNA [NM_008535]	17095	NM_008535	lymphoblastomic leukemia 1	NM_008535	Mm.4925
A_55_P2138237	0.048	up	1.4694864	Samd7	Mus musculus sterile alpha motif domain containing 7 (Samd7), transcript variant 1, mRNA [NM_029489]	75953	NM_029489	sterile alpha motif domain containing 7	NM_029489	Mm.308084
A_55_P2079561	0.001	up	1.4713726	Lilra6	Mus musculus leukocyte immunoglobulin-like receptor, subfamily A (with TM domain), member 6 (Lilra6), transcript variant 1, mRNA [NM_011090]	18726	NM_011090	leukocyte immunoglobulin-like receptor, subfamily A (with TM domain), member 6	NM_011090	Mm.440989
A_55_P2035623	0.033	up	1.4714043	Zfp473	Mus musculus zinc finger protein 473 (Zfp473), mRNA [NM_178734]	243963	NM_178734	zinc finger protein 473	NM_178734	Mm.382935
A_52_P226127	0.014	up	1.4715111	Dido1	Mus musculus death inducer-obliterator 1 (Dido1), transcript variant 1, mRNA [NM_011805]	23856	NM_011805	death inducer-obliterator 1	NM_011805	Mm.253836
A_55_P2011390	0.035	up	1.4722017	Tead1	Mus musculus TEA domain family member 1 (Tead1), transcript variant 1, mRNA [NM_001166584]	21676	NM_001166584	TEA domain family member 1	NM_001166584	Mm.24685
A_55_P1959858	0.019	up	1.4730775	Cst10	Mus musculus cystatin 10 (chondrocytes) (Cst10), mRNA [NM_021405]	58214	NM_021405	cystatin 10 (chondrocytes)	NM_021405	Mm.117117
A_65_P13459	0.015	up	1.4736181	Cd300lb	Mus musculus CD300 antigen like family member B (Cd300lb), mRNA [NM_199221]	217304	NM_199221	CD300 antigen like family member B	NM_199221	Mm.185355
A_55_P2329923	0.041	up	1.4738891	Camkk2	Mus musculus calcium/calmodulin-dependent protein kinase kinase 2, beta (Camkk2), transcript variant 1, mRNA [NM_001199676]	207565	NM_001199676	calcium/calmodulin-dependent protein kinase kinase 2, beta	NM_001199676	Mm.289237
A_55_P2016739	0.000	up	1.4739368	Rbm12b2	Mus musculus RNA binding motif protein 12 B2 (Rbm12b2), mRNA [NM_198957]	77604	NM_198957	RNA binding motif protein 12 B2	NM_198957	Mm.474129
A_55_P2053404	0.010	up	1.4740534	Pirb	leukocyte immunoglobulin-like receptor, subfamily B (with TM and ITIM domains), member 3 [Source:MGI Symbol;Acc:MGI:894311] [ENSMUST00000137474]	18733	AK137448	paired Ig-like receptor B		Mm.441334
A_55_P2036538	0.026	up	1.474461	Wbscr27	Mus musculus Williams Beuren syndrome chromosome region 27 (human) (Wbscr27), mRNA [NM_024479]	79565	NM_024479	Williams Beuren syndrome chromosome region 27 (human)	NM_024479	Mm.34235
A_55_P2033041	0.005	up	1.4751258	Sirpb1b	Mus musculus signal-regulatory protein beta 1B (Sirpb1b), mRNA [NM_001173460]	668101	NM_001173460	signal-regulatory protein beta 1B	NM_001173460	Mm.423523
A_52_P176619	0.008	up	1.4756994	Atat1	Mus musculus alpha tubulin acetyltransferase 1 (Atat1), transcript variant 1, mRNA [NM_001142744]	73242	NM_001142744	alpha tubulin acetyltransferase 1	NM_001142744	Mm.273155
A_55_P2173183	0.023	up	1.4762809	Cdk5r1	Mus musculus cyclin-dependent kinase 5, regulatory subunit 1 (p35) (Cdk5r1), mRNA [NM_009871]	12569	NM_009871	cyclin-dependent kinase 5, regulatory subunit 1 (p35)	NM_009871	Mm.142275
A_55_P2034710	0.017	up	1.4776641	Clvs1	Mus musculus clavesin 1 (Clvs1), mRNA [NM_028940]	74438	NM_028940	clavesin 1	NM_028940	Mm.473368
A_55_P2007630	0.008	up	1.4777148	Sez6l2	Mus musculus seizure related 6 homolog like 2 (Sez6l2), transcript variant 1, mRNA [NM_144926]	233878	NM_144926	seizure related 6 homolog like 2	NM_144926	Mm.283926
A_55_P2327177	0.008	up	1.4789286	Exoc8	Mus musculus exocyst complex component 8 (Exoc8), mRNA [NM_198103]	102058	NM_198103	exocyst complex component 8	NM_198103	Mm.347360
A_55_P2110915	0.028	up	1.4802592	Flrt1	Mus musculus fibronectin leucine rich transmembrane protein 1 (Flrt1), mRNA [NM_201411]	396184	NM_201411	fibronectin leucine rich transmembrane protein 1	NM_201411	Mm.386930
A_55_P2159432	0.036	up	1.4804921	Fndc4	Mus musculus fibronectin type III domain containing 4 (Fndc4), mRNA [NM_022424]	64339	NM_022424	fibronectin type III domain containing 4	NM_022424	Mm.292544
A_52_P102248	0.026	up	1.480888	Mex3b	Mus musculus mex3 homolog B (C. elegans) (Mex3b), mRNA [NM_175366]	108797	NM_175366	mex3 homolog B (C. elegans)	NM_175366	Mm.306789
A_52_P105537	0.039	up	1.4816101	Nov	Mus musculus nephroblastoma overexpressed gene (Nov), mRNA [NM_010930]	18133	NM_010930	nephroblastoma overexpressed gene	NM_010930	Mm.5167
A_55_P2137452	0.031	up	1.4817071	Vmn1r207-ps	Mus musculus vomeronasal 1 receptor 207, pseudogene (Vmn1r207-ps), mRNA [NM_001166709]	432735	NM_001166709	vomeronasal 1 receptor 207, pseudogene	NM_001166709	Mm.480282

A_52_P374839	0.004	up	1.4828949	Atp6v1g2	Mus musculus ATPase, H+ transporting, lysosomal V1 subunit G2 (Atp6v1g2), mRNA [NM_023179]	66237	NM_023179	ATPase, H+ transporting, lysosomal V1 subunit G2	NM_023179	Mm.396107
A_55_P2011286	0.014	up	1.4844781	Hopx	Mus musculus HOP homeobox (Hopx), transcript variant 1, mRNA [NM_175606]	74318	NM_175606	HOP homeobox	NM_175606	Mm.181852
A_55_P1964408	0.015	up	1.4858786	Cryab	Mus musculus crystallin, alpha B (Cryab), mRNA [NM_009964]	12955	NM_009964	crystallin, alpha B	NM_009964	Mm.178
A_51_P176156	0.012	up	1.4866377	Sirpb1b	Mus musculus signal-regulatory protein beta 1B (Sirpb1b), mRNA [NM_001173460]	668101	NM_001173460	signal-regulatory protein beta 1B	NM_001173460	Mm.423523
A_52_P487615	0.027	up	1.4874183	Fam105a	Mus musculus family with sequence similarity 105, member A (Fam105a), transcript variant 3, mRNA [NM_198301]	223433	NM_198301	family with sequence similarity 105, member A	NM_198301	Mm.266485
A_55_P2082434	0.017	up	1.4891214	Btbd11	Mus musculus BTB (POZ) domain containing 11 (Btbd11), transcript variant 1, mRNA [NM_028709]	74007	NM_028709	BTB (POZ) domain containing 11	NM_028709	Mm.36544
A_51_P128320	0.035	up	1.4891868	Muc2	Mus musculus mucin 2 (Muc2), mRNA [NM_023566]	17831	NM_023566	mucin 2	NM_023566	Mm.461296
A_55_P2154263	0.000	up	1.4892819	Cbx1	Mus musculus 9.5 days embryo parthenogenote cDNA, RIKEN full-length enriched library, clone:B130005N18 product:chromobox homolog 1 (Drosophila HP1 beta), full insert sequence. [AK157710]	12412	AK157710	chromobox 1		Mm.29055
A_55_P1954718	0.042	up	1.4893869	Cyb561	Mus musculus cytochrome b-561 (Cyb561), mRNA [NM_007805]	13056	NM_007805	cytochrome b-561	NM_007805	Mm.149403
A_55_P2059640	0.001	up	1.4897777	Abhd14b	Mus musculus abhydrolase domain containing 14b (Abhd14b), mRNA [NM_029631]	76491	NM_029631	abhydrolase domain containing 14b	NM_029631	Mm.335427
A_55_P2330545	0.019	up	1.4907813	Zfp81	zinc finger protein 81 [Source:MGI Symbol;Acc:MGI:1890752] [ENSMUST00000054072]	224694	AK011718	zinc finger protein 81		Mm.220824
A_66_P115541	0.005	up	1.4944358	Sox12	Mus musculus SRY-box containing gene 12 (Sox12), mRNA [NM_011438]	20667	NM_011438	SRY-box containing gene 12	NM_011438	Mm.28424
A_55_P2028883	0.025	up	1.4944786	Rnft2	Mus musculus ring finger protein, transmembrane 2 (Rnft2), transcript variant 1, mRNA [NM_172998]	269695	NM_172998	ring finger protein, transmembrane 2	NM_172998	Mm.320042
A_52_P482814	0.009	up	1.4948194	Tmco2	Mus musculus transmembrane and coiled-coil domains 2 (Tmco2), mRNA [NM_001081312]	69469	NM_001081312	transmembrane and coiled-coil domains 2	NM_001081312	Mm.481120
A_55_P2266295	0.006	up	1.4961853	Fzd3	Mus musculus frizzled homolog 3 (Drosophila) (Fzd3), mRNA [NM_021458]	14365	NM_021458	frizzled homolog 3 (Drosophila)	NM_021458	Mm.214687
A_55_P2068862	0.001	up	1.49646	Pigv	Mus musculus phosphatidylinositol glycan anchor biosynthesis, class V (Pigv), transcript variant 1, mRNA [NM_178698]	230801	NM_178698	phosphatidylinositol glycan anchor biosynthesis, class V	NM_178698	Mm.217004
A_66_P119841	0.008	up	1.4972076	Ddx17	Mus musculus DEAD (Asp-Glu-Ala-Asp) box polypeptide 17 (Ddx17), transcript variant 2, mRNA [NM_199079]	67040	NM_199079	DEAD (Asp-Glu-Ala-Asp) box polypeptide 17	NM_199079	Mm.29644
A_55_P2042923	0.036	up	1.4972326	Sgk2	Mus musculus serum/glucocorticoid regulated kinase 2 (Sgk2), mRNA [NM_013731]	27219	NM_013731	serum/glucocorticoid regulated kinase 2	NM_013731	Mm.26462
A_55_P2010003	0.006	up	1.4975216	Zfp113	Mus musculus zinc finger protein 113 (Zfp113), mRNA [NM_019747]	56314	NM_019747	zinc finger protein 113	NM_019747	Mm.19274
A_55_P2025595	0.040	up	1.4981002	Gcnt2	Mus musculus glucosaminyl (N-acetyl) transferase 2, I-branching enzyme (Gcnt2), transcript variant 1, mRNA [NM_008105]	14538	NM_008105	glucosaminyl (N-acetyl) transferase 2, I-branching enzyme	NM_008105	Mm.314757
A_65_P12993	0.043	up	1.4984338	Kif1b	Mus musculus kinesin family member 1B (Kif1b), transcript variant 1, mRNA [NM_008441]	16561	NM_008441	kinesin family member 1B	NM_008441	Mm.402393
A_55_P2083764	0.006	up	1.4985363	Rbm12	RNA binding motif protein 12 [Source:MGI Symbol;Acc:MGI:1922960] [ENSMUST00000109604]	75710	AK020481	RNA binding motif protein 12		Mm.441281
A_55_P2139207	0.033	up	1.498549	Ccdc142	Mus musculus coiled-coil domain containing 142 (Ccdc142), mRNA [NM_001081266]	243510	NM_001081266	coiled-coil domain containing 142	NM_001081266	Mm.436553
A_55_P2076866	0.001	up	1.4986587	Megf6	Mus musculus multiple EGF-like-domains 6 (Megf6), mRNA [NM_001162977]	230971	NM_001162977	multiple EGF-like-domains 6	NM_001162977	Mm.275216
A_55_P2079560	0.015	up	1.498888	Lilra6	Mus musculus leukocyte immunoglobulin-like receptor, subfamily A (with TM domain), member 6 (Lilra6), transcript variant 1, mRNA [NM_011090]	18726	NM_011090	leukocyte immunoglobulin-like receptor, subfamily A (with TM domain), member 6	NM_011090	Mm.440989
A_51_P233727	0.007	up	1.4993619	Sapcd1	Mus musculus suppressor APC domain containing 1 (Sapcd1), transcript variant 1, mRNA [NM_023893]	78376	NM_023893	suppressor APC domain containing 1	NM_023893	Mm.390966
A_55_P2149215	0.035	up	1.5002452	Camk2a	Mus musculus calcium/calmodulin-dependent protein kinase II alpha (Camk2a), transcript variant 1, mRNA [NM_009792]	12322	NM_009792	calcium/calmodulin-dependent protein kinase II alpha	NM_009792	Mm.131530
A_55_P2176792	0.038	up	1.5002561	Sh3gl3	Mus musculus SH3-domain GRB2-like 3 (Sh3gl3), transcript variant 1, mRNA [NM_017400]	20408	NM_017400	SH3-domain GRB2-like 3	NM_017400	Mm.736
A_51_P312035	0.007	up	1.5004423	Cd300e	Mus musculus CD300e antigen (Cd300e), mRNA [NM_172050]	217306	NM_172050	CD300e antigen	NM_172050	Mm.222667
A_55_P2170847	0.023	up	1.5007305	Fbxo27	Mus musculus F-box protein 27 (Fbxo27), transcript variant 1, mRNA [NM_001163702]	233040	NM_001163702	F-box protein 27	NM_001163702	Mm.116116
A_66_P130730	0.011	up	1.500768	Zfp963	Mus musculus zinc finger protein 963 (Zfp963), mRNA [NM_001200023]	620419	NM_001200023	zinc finger protein 963	NM_001200023	Mm.460423
A_55_P2416862	0.022	up	1.5008044	Tmem175	Mus musculus 0 day neonate head cDNA, RIKEN full-length enriched library, clone:4832439D14 product:unclassifiable, full insert sequence. [AK076446]	72392	AK076446	transmembrane protein 175		Mm.258324
A_52_P536494	0.043	up	1.5018535	Mycn	Mus musculus v-myc myelocytomatosis viral related oncogene, neuroblastoma derived (avian) (Mycn), mRNA [NM_008709]	18109	NM_008709	v-myc myelocytomatosis viral related oncogene, neuroblastoma derived (avian)	NM_008709	Mm.16469
A_55_P2052016	0.017	up	1.5019975	Crispld2	Mus musculus cysteine-rich secretory protein LCCL domain containing 2 (Crispld2), mRNA [NM_030209]	78892	NM_030209	cysteine-rich secretory protein LCCL domain containing 2	NM_030209	Mm.264680
A_51_P329490	0.009	up	1.5022004	Slc25a40	Mus musculus solute carrier family 25, member 40 (Slc25a40), mRNA [NM_178766]	319653	NM_178766	solute carrier family 25, member 40	NM_178766	Mm.146262

A_55_P2008895	0.019	up	1.5031004	Tmsb15b1	Mus musculus thymosin beta 15b1 (Tmsb15b1), mRNA [NM_001081983]	666244	NM_001081983	thymosin beta 15b1	NM_001081983	Mm.381904
A_55_P2030491	0.018	up	1.50408	Srsf2	serine/arginine-rich splicing factor 2 [Source:MGI Symbol;Acc:MGI:98284] [ENSMUST00000177429]	20382	AF250135	serine/arginine-rich splicing factor 2		
A_51_P477682	0.033	up	1.5045619	Prss12	Mus musculus protease, serine, 12 neurotrypsin (motopsin) (Prss12), mRNA [NM_008939]	19142	NM_008939	protease, serine, 12 neurotrypsin (motopsin)	NM_008939	Mm.9431
A_55_P2152862	0.030	up	1.5047656	Ints10	Mus musculus 10 days neonate cerebellum cDNA, RIKEN full-length enriched library, clone:B930018M20 product:hypothetical Tetratricopeptide repeat (TPR) structure containing protein, full insert sequence. [AK047093]	70885	AK047093	integrator complex subunit 10		Mm.252421
A_55_P1970788	0.009	up	1.5049702	C5ar2	Mus musculus complement component 5a receptor 2 (C5ar2), transcript variant 1, mRNA [NM_176912]	319430	NM_176912	complement component 5a receptor 2	NM_176912	Mm.239570
A_55_P1953503	0.032	up	1.5052683	Lrrc4b	Mus musculus leucine rich repeat containing 4B (Lrrc4b), mRNA [NM_198250]	272381	NM_198250	leucine rich repeat containing 4B	NM_198250	Mm.44173
A_51_P386503	0.027	up	1.5071293	Abhd15	Mus musculus abhydrolase domain containing 15 (Abhd15), mRNA [NM_026185]	67477	NM_026185	abhydrolase domain containing 15	NM_026185	Mm.432526
A_55_P2096942	0.010	up	1.5073389	Nrn1l	Mus musculus neuritin 1-like (Nrn1l), mRNA [NM_175024]	234700	NM_175024	neuritin 1-like	NM_175024	Mm.133990
A_55_P2043668	0.002	up	1.5073901	Zfp617	Mus musculus zinc finger protein 617 (Zfp617), mRNA [NM_133358]	170938	NM_133358	zinc finger protein 617	NM_133358	Mm.480419
A_52_P88793	0.003	up	1.5091544	Zfp933	Mus musculus zinc finger protein 933 (Zfp933), mRNA [NM_198619]	242747	NM_198619	zinc finger protein 933	NM_198619	Mm.332053
A_52_P194464	0.004	up	1.5100888	Ercc6l2	Mus musculus excision repair cross-complementing rodent repair deficiency, complementation group 6 like 2 (Ercc6l2), transcript variant 2, mRNA [NM_023507]	76251	NM_023507	excision repair cross-complementing rodent repair deficiency, complementation group 6 like 2	NM_023507	Mm.186610
A_51_P456957	0.005	up	1.5114971	Pex11a	Mus musculus peroxisomal biogenesis factor 11 alpha (Pex11a), mRNA [NM_011068]	18631	NM_011068	peroxisomal biogenesis factor 11 alpha	NM_011068	Mm.20615
A_55_P2008126	0.021	up	1.511554	Rad51	RAD51 homolog [Source:MGI Symbol;Acc:MGI:97890] [ENSMUST00000110828]	19361	AK045050	RAD51 homolog		Mm.330492
A_51_P300506	0.020	up	1.5119703	Cox6b2	Mus musculus cytochrome c oxidase subunit VIb polypeptide 2 (Cox6b2), transcript variant 1, mRNA [NM_183405]	333182	NM_183405	cytochrome c oxidase subunit VIb polypeptide 2	NM_183405	Mm.29625
A_52_P625624	0.020	up	1.5125315	Stau2	Mus musculus staufen (RNA binding protein) homolog 2 (Drosophila) (Stau2), transcript variant 3, mRNA [NM_025303]	29819	NM_025303	staufen (RNA binding protein) homolog 2 (Drosophila)	NM_025303	Mm.216257
A_52_P481686	0.021	up	1.5133067	Wtip	Mus musculus WT1-interacting protein (Wtip), mRNA [NM_207212]	101543	NM_207212	WT1-interacting protein	NM_207212	Mm.422738
A_55_P2142738	0.006	up	1.5135951	Lrrc48	Mus musculus leucine rich repeat containing 48 (Lrrc48), mRNA [NM_029044]	74665	NM_029044	leucine rich repeat containing 48	NM_029044	Mm.133570
A_55_P2104583	0.002	up	1.5142299	C87436	Mus musculus expressed sequence C87436 (C87436), transcript variant 4, mRNA [NM_001243742]	232196	NM_001243742	expressed sequence C87436	NM_001243742	Mm.404040
A_55_P2024155	0.002	up	1.5155388	Zbtb16	Mus musculus zinc finger and BTB domain containing 16 (Zbtb16), mRNA [NM_001033324]	235320	NM_001033324	zinc finger and BTB domain containing 16	NM_001033324	Mm.457803
A_55_P2076846	0.039	up	1.5157826	Tead2	Mus musculus TEA domain family member 2 (Tead2), transcript variant 1, mRNA [NM_001285498]	21677	NM_001285498	TEA domain family member 2	NM_001285498	Mm.3019
A_65_P19112	0.013	up	1.516645	Zfp397	Mus musculus zinc finger protein 397 (Zfp397), mRNA [NM_027007]	69256	NM_027007	zinc finger protein 397	NM_027007	Mm.218343
A_55_P2023617	0.003	up	1.5176531	Kiss1r	Mus musculus KISS1 receptor (Kiss1r), mRNA [NM_053244]	114229	NM_053244	KISS1 receptor	NM_053244	Mm.191035
A_51_P215438	0.007	up	1.5190463	Prodh	Mus musculus proline dehydrogenase (Prodh), mRNA [NM_011172]	19125	NM_011172	proline dehydrogenase	NM_011172	Mm.28456
A_51_P165082	0.001	up	1.5192257	Tmem191c	Mus musculus transmembrane protein 191C (Tmem191c), mRNA [NM_177473]	224019	NM_177473	transmembrane protein 191C	NM_177473	Mm.246388
A_51_P235415	0.039	up	1.5198421	Olfm2	Mus musculus olfactomedin 2 (Olfm2), mRNA [NM_173777]	244723	NM_173777	olfactomedin 2	NM_173777	Mm.293552
A_55_P2064995	0.025	up	1.5199355	Capzb	Mus musculus capping protein (actin filament) muscle Z-line, beta (Capzb), transcript variant 1, mRNA [NM_001037761]	12345	NM_001037761	capping protein (actin filament) muscle Z-line, beta	NM_001037761	Mm.2945
A_52_P512575	0.008	up	1.5211166	Hopx	Mus musculus HOP homeobox (Hopx), transcript variant 1, mRNA [NM_175606]	74318	NM_175606	HOP homeobox	NM_175606	Mm.181852
A_55_P1963198	0.025	up	1.5221595	Chchd7	coiled-coil-helix-coiled-coil-helix domain containing 7 [Source:MGI Symbol;Acc:MGI:1913683] [ENSMUST00000121210]	66433		coiled-coil-helix-coiled-coil-helix domain containing 7		
A_55_P1969615	0.008	up	1.5222158	Pnck	Mus musculus pregnancy upregulated non-ubiquitously expressed CaM kinase (Pnck), transcript variant 1, mRNA [NM_001199351]	93843	NM_001199351	pregnancy upregulated non-ubiquitously expressed CaM kinase	NM_001199351	Mm.89564
A_52_P199776	0.012	up	1.5228201	Snhg7	Mus musculus small nucleolar RNA host gene (non-protein coding) 7 (Snhg7), long non-coding RNA [NR_024068]	72091	NR_024068	small nucleolar RNA host gene (non-protein coding) 7	NR_024068	Mm.27103
A_51_P397468	0.011	up	1.5251408	Rundc3a	Mus musculus RUN domain containing 3A (Rundc3a), transcript variant 1, mRNA [NM_016759]	51799	NM_016759	RUN domain containing 3A	NM_016759	Mm.2560
A_55_P1975155	0.009	up	1.5265101	Galnt6	Mus musculus UDP-N-acetyl-alpha-D-galactosamine:polypeptide N-acetylgalactosaminyltransferase 6 (Galnt6), transcript variant 1, mRNA [NM_001161767]	207839	NM_001161767	UDP-N-acetyl-alpha-D-galactosamine:polypeptide N-acetylgalactosaminyltransferase 6	NM_001161767	Mm.22969
A_52_P55772	0.034	up	1.5266297	Tbxa2r	Mus musculus thromboxane A2 receptor (Tbxa2r), transcript variant 1, mRNA [NM_009325]	21390	NM_009325	thromboxane A2 receptor	NM_009325	Mm.4545
A_52_P891775	0.040	up	1.5278579	Cdr2l	Mus musculus cerebellar degeneration-related protein 2-like (Cdr2l), mRNA [NM_001080929]	237988	NM_001080929	cerebellar degeneration-related protein 2-like	NM_001080929	Mm.102872

A_55_P1982563	0.003	up	1.5287867	Rhbdf1	Mus musculus rhomboid family 1 (Drosophila) (Rhbdf1), mRNA [NM_010117]	13650	NM_010117	rhomboid family 1 (Drosophila)	NM_010117	Mm.11545
A_51_P117236	0.005	up	1.5290183	Zfp354a	Mus musculus zinc finger protein 354A (Zfp354a), mRNA [NM_009329]	21408	NM_009329	zinc finger protein 354A	NM_009329	Mm.4792
A_52_P608132	0.004	up	1.5307223	Snx32	Mus musculus sorting nexin 32 (Snx32), mRNA [NM_001024560]	225861	NM_001024560	sorting nexin 32	NM_001024560	Mm.20071
A_52_P151528	0.020	up	1.5332603	Catsperg1	Mus musculus catsper channel auxiliary subunit gamma 1 (Catsperg1), mRNA [NM_001164658]	320225	NM_001164658	catsper channel auxiliary subunit gamma 1	NM_001164658	Mm.349382
A_51_P369426	0.028	up	1.5350566	Mss51	Mus musculus MSS51 mitochondrial translational activator (Mss51), mRNA [NM_029104]	74843	NM_029104	MSS51 mitochondrial translational activator	NM_029104	Mm.295241
A_55_P1999760	0.008	up	1.535076	Lrrc51	Mus musculus leucine rich repeat containing 51 (Lrrc51), transcript variant 3, mRNA [NM_001162974]	69358	NM_001162974	leucine rich repeat containing 51	NM_001162974	Mm.214841
A_55_P1955308	0.002	up	1.5355425	Sirpb1a	Mus musculus signal-regulatory protein beta 1A (Sirpb1a), transcript variant 3, mRNA [NM_001002898]	320832	NM_001002898	signal-regulatory protein beta 1A	NM_001002898	Mm.425126
A_55_P2149575	0.015	up	1.5363148	Sdr39u1	Mus musculus short chain dehydrogenase/reductase family 39U, member 1 (Sdr39u1), mRNA [NM_001082975]	654795	NM_001082975	short chain dehydrogenase/reductase family 39U, member 1	NM_001082975	Mm.486218
A_51_P478952	0.004	up	1.5369042	N4bp2l1	Mus musculus NEDD4 binding protein 2-like 1 (N4bp2l1), mRNA [NM_133898]	100637	NM_133898	NEDD4 binding protein 2-like 1	NM_133898	Mm.279932
A_52_P283524	0.010	up	1.5370286	Rcan3	Mus musculus regulator of calcineurin 3 (Rcan3), mRNA [NM_022980]	53902	NM_022980	regulator of calcineurin 3	NM_022980	Mm.331970
A_51_P487668	0.008	up	1.538463	H2afy3	Mus musculus H2A histone family, member Y3 (H2afy3), non-coding RNA [NR_003523]	67552	NR_003523	H2A histone family, member Y3	NR_003523	Mm.484027
A_52_P500274	0.035	up	1.539653	Ntrk3	Mus musculus neurotrophic tyrosine kinase, receptor, type 3 (Ntrk3), transcript variant 2, mRNA [NM_182809]	18213	NM_182809	neurotrophic tyrosine kinase, receptor, type 3	NM_182809	Mm.33496
A_52_P385594	0.019	up	1.5408483	Tal2	Mus musculus T cell acute lymphocytic leukemia 2 (Tal2), mRNA [NM_009317]	21350	NM_009317	T cell acute lymphocytic leukemia 2	NM_009317	Mm.56955
A_52_P257041	0.040	up	1.5409498	Nudt6	Mus musculus nudix (nucleoside diphosphate linked moiety X)-type motif 6 (Nudt6), mRNA [NM_153561]	229228	NM_153561	nudix (nucleoside diphosphate linked moiety X)-type motif 6	NM_153561	Mm.472818
A_55_P2053032	0.032	up	1.541605	Xkr8	Mus musculus X Kell blood group precursor related family member 8 homolog (Xkr8), mRNA [NM_201368]	381560	NM_201368	X Kell blood group precursor related family member 8 homolog	NM_201368	Mm.44558
A_55_P1989539	0.042	up	1.5416757	Cklf	Mus musculus chemokine-like factor (Cklf), transcript variant 4, mRNA [NM_001037841]	75458	NM_001037841	chemokine-like factor	NM_001037841	Mm.269219
A_55_P2065866	0.023	up	1.5425735	Cygb	Mus musculus cytoglobin (Cygb), mRNA [NM_030206]	114886	NM_030206	cytoglobin	NM_030206	Mm.34598
A_51_P250058	0.026	up	1.5432932	Epas1	Mus musculus endothelial PAS domain protein 1 (Epas1), mRNA [NM_010137]	13819	NM_010137	endothelial PAS domain protein 1	NM_010137	Mm.1415
A_51_P379660	0.011	up	1.5437572	Mllt11	Mus musculus myeloid/lymphoid or mixed-lineage leukemia (trithorax homolog, Drosophila); translocated to, 11 (Mllt11), mRNA [NM_019914]	56772	NM_019914	myeloid/lymphoid or mixed-lineage leukemia (trithorax homolog, Drosophila); translocated to, 11	NM_019914	Mm.331208
A_55_P2089223	0.003	up	1.5466073	Ak4	Mus musculus adenylate kinase 4 (Ak4), transcript variant 1, mRNA [NM_001177602]	11639	NM_001177602	adenylate kinase 4	NM_001177602	Mm.42040
A_55_P1952882	0.000	up	1.547333	Cyp4f18	Mus musculus cytochrome P450 CYP4F18 mRNA, complete cds. [AF233647]	72054	AF233647	cytochrome P450, family 4, subfamily f, polypeptide 18		Mm.160020
A_55_P2067518	0.008	up	1.5473729	Slc13a3	Mus musculus solute carrier family 13 (sodium-dependent dicarboxylate transporter), member 3 (Slc13a3), mRNA [NM_054055]	114644	NM_054055	solute carrier family 13 (sodium-dependent dicarboxylate transporter), member 3	NM_054055	Mm.250738
A_55_P2091831	0.013	up	1.5482485	Krtap1-4	Mus musculus keratin associated protein 1-4 (Krtap1-4), mRNA [NM_001039502]	629873	NM_001039502	keratin associated protein 1-4	NM_001039502	Mm.423246
A_66_P140508	0.030	up	1.5494934	Krtap31-2	Mus musculus keratin associated protein 31-2 (Krtap31-2), mRNA [NM_001025244]	432602	NM_001025244	keratin associated protein 31-2	NM_001025244	Mm.246878
A_55_P2031481	0.002	up	1.5513638	Fam131a	Mus musculus family with sequence similarity 131, member A (Fam131a), mRNA [NM_133778]	78408	NM_133778	family with sequence similarity 131, member A	NM_133778	Mm.196512
A_55_P2156155	0.026	up	1.5516629	Gadl1	Mus musculus glutamate decarboxylase-like 1 (Gadl1), mRNA [NM_028638]	73748	NM_028638	glutamate decarboxylase-like 1	NM_028638	Mm.485018
A_55_P2071059	0.037	up	1.5519704	Nab2	Mus musculus Ngfi-A binding protein 2 (Nab2), transcript variant 1, mRNA [NM_008668]	17937	NM_008668	Ngfi-A binding protein 2	NM_008668	Mm.336898
A_51_P308347	0.011	up	1.5526282	Dact2	Mus musculus dapper homolog 2, antagonist of beta-catenin (xenopus) (Dact2), mRNA [NM_172826]	240025	NM_172826	dapper homolog 2, antagonist of beta-catenin (xenopus)	NM_172826	Mm.79993
A_55_P2041708	0.007	up	1.5546508	Gas2	Mus musculus growth arrest specific 2 (Gas2), mRNA [NM_008087]	14453	NM_008087	growth arrest specific 2	NM_008087	Mm.207360
A_51_P449824	0.011	up	1.5550592	Exoc3l2	exocyst complex component 3-like 2 [Source:MGI Symbol;Acc:MGI:1921713] [ENSMUST00000011407]	74463	XM_001471750	exocyst complex component 3-like 2	XM_001471750	Mm.333647
A_55_P2083411	0.000	up	1.5559179	Cyp4f18	Mus musculus cytochrome P450, family 4, subfamily f, polypeptide 18 (Cyp4f18), mRNA [NM_024444]	72054	NM_024444	cytochrome P450, family 4, subfamily f, polypeptide 18	NM_024444	Mm.160020
A_51_P201490	0.021	up	1.5561322	Olfr242	Mus musculus olfactory receptor 242 (Olfr242), mRNA [NM_010974]	406175	NM_010974	olfactory receptor 242	NM_010974	Mm.246532
A_52_P609120	0.006	up	1.5568955	Pdxp	Mus musculus pyridoxal (pyridoxine, vitamin B6) phosphatase (Pdxp), mRNA [NM_020271]	57028	NM_020271	pyridoxal (pyridoxine, vitamin B6) phosphatase	NM_020271	Mm.263169
A_55_P2022604	0.006	up	1.5570291	Exoc3l4	Mus musculus exocyst complex component 3-like 4 (Exoc3l4), mRNA [NM_028807]	74190	NM_028807	exocyst complex component 3-like 4	NM_028807	Mm.248640
A_55_P1968153	0.016	up	1.5575699	Pcgf2	Mus musculus polycomb group ring finger 2 (Pcgf2), transcript variant 1, mRNA [NM_009545]	22658	NM_009545	polycomb group ring finger 2	NM_009545	Mm.2418

A_51_P184484	0.037	up	1.5584984	Mmp13	Mus musculus matrix metallopeptidase 13 (Mmp13), mRNA [NM_008607]	17386	NM_008607	matrix metallopeptidase 13	NM_008607	Mm.5022
A_55_P2003199	0.001	up	1.5587971	Setd1b	SET domain containing 1B [Source:MGI Symbol;Acc:MGI:2652820] [ENSMUST00000100731]	208043	AK141316	SET domain containing 1B		Mm.250391
A_55_P2134616	0.002	up	1.5590839	Med12l	Mus musculus mRNA for mKIAA3007 protein. [AK129475]	329650	AK129475	mediator of RNA polymerase II transcription, subunit 12 homolog (yeast)-like		Mm.240059
A_52_P104091	0.043	up	1.5607264	Slc25a27	Mus musculus solute carrier family 25, member 27 (Slc25a27), mRNA [NM_028711]	74011	NM_028711	solute carrier family 25, member 27	NM_028711	Mm.288697
A_52_P488623	0.049	up	1.5609661	Fam169a	Mus musculus family with sequence similarity 169, member A (Fam169a), transcript variant 1, mRNA [NM_001100458]	320557	NM_001100458	family with sequence similarity 169, member A	NM_001100458	Mm.458280
A_55_P2000958	0.009	up	1.5627816	Sdr39u1	Mus musculus short chain dehydrogenase/reductase family 39U, member 1 (Sdr39u1), mRNA [NM_001082975]	654795	NM_001082975	short chain dehydrogenase/reductase family 39U, member 1	NM_001082975	Mm.486218
A_55_P2003414	0.049	up	1.5634348	Kng1	Mus musculus kininogen 1 (Kng1), transcript variant 2, mRNA [NM_023125]	16644	NM_023125	kininogen 1	NM_023125	Mm.2160
A_55_P2066103	0.004	up	1.5646662	Hoxa9	Mus musculus homeobox A9 (Hoxa9), transcript variant 1, mRNA [NM_010456]	15405	NM_010456	homeobox A9	NM_010456	Mm.4694
A_55_P2147703	0.029	up	1.5671086	Vmn2r56	Mus musculus vomeronasal 2, receptor 56 (Vmn2r56), mRNA [NM_001104648]	629079	NM_001104648	vomeronasal 2, receptor 56	NM_001104648	Mm.469869
A_55_P2078960	0.003	up	1.5682069	Tdh	Mus musculus L-threonine dehydrogenase (Tdh), mRNA [NM_021480]	58865	NM_021480	L-threonine dehydrogenase	NM_021480	Mm.354229
A_55_P2367843	0.031	up	1.569252	Efcab4a	Mus musculus 10 days neonate cerebellum cDNA, RIKEN full-length enriched library, clone:B930011K17 product:unclassifiable, full insert sequence. [AK140493]	213573	AK140493	EF-hand calcium binding domain 4A		Mm.386851
A_51_P244303	0.019	up	1.5694579	Smc1b	Mus musculus structural maintenance of chromosomes 1B (Smc1b), mRNA [NM_080470]	140557	NM_080470	structural maintenance of chromosomes 1B	NM_080470	Mm.182737
A_52_P295104	0.017	up	1.5695626	Smim5	Mus musculus small integral membrane protein 5 (Smim5), mRNA [NM_183259]	66528	NM_183259	small integral membrane protein 5	NM_183259	Mm.275740
A_55_P2040011	0.014	up	1.5708767	Itga7	Mus musculus integrin alpha 7 (Itga7), mRNA [NM_008398]	16404	NM_008398	integrin alpha 7	NM_008398	Mm.179747
A_55_P2045040	0.018	up	1.5711335	F7	Mus musculus coagulation factor VII (F7), mRNA [NM_010172]	14068	NM_010172	coagulation factor VII	NM_010172	Mm.4827
A_52_P686785	0.034	up	1.5717871	Lyve1	Mus musculus lymphatic vessel endothelial hyaluronan receptor 1 (Lyve1), mRNA [NM_053247]	114332	NM_053247	lymphatic vessel endothelial hyaluronan receptor 1	NM_053247	Mm.396078
A_51_P210286	0.004	up	1.5719697	Cacng1	Mus musculus calcium channel, voltage-dependent, gamma subunit 1 (Cacng1), mRNA [NM_007582]	12299	NM_007582	calcium channel, voltage-dependent, gamma subunit 1	NM_007582	Mm.57093
A_51_P351923	0.042	up	1.5731944	Chd3os	Mus musculus Chd3 opposite strand transcript (Chd3os), long non-coding RNA [NR_027827]	80515	NR_027827	Chd3 opposite strand transcript	NR_027827	Mm.5324
A_52_P270145	0.008	up	1.5734469	Zfp329	Mus musculus zinc finger protein 329 (Zfp329), mRNA [NM_026046]	67230	NM_026046	zinc finger protein 329	NM_026046	Mm.333574
A_55_P1956593	0.009	up	1.5742955	Plekha4	Mus musculus pleckstrin homology domain containing, family A (phosphoinositide binding specific) member 4 (Plekha4), mRNA [NM_148927]	69217	NM_148927	pleckstrin homology domain containing, family A (phosphoinositide binding specific) member 4	NM_148927	Mm.274158
A_55_P1973553	0.036	up	1.5745028	Bbs12	Mus musculus Bardet-Biedl syndrome 12 (human) (Bbs12), transcript variant 2, mRNA [NM_001255992]	241950	NM_001255992	Bardet-Biedl syndrome 12 (human)	NM_001255992	Mm.32171
A_55_P2156937	0.038	up	1.575196	Sytl3	Mus musculus synaptotagmin-like 3 (Sytl3), transcript variant 1, mRNA [NM_031395]	83672	NM_031395	synaptotagmin-like 3	NM_031395	Mm.442201
A_52_P221756	0.012	up	1.5754011	Spock2	Mus musculus sparc/osteonectin, cwcv and kazal-like domains proteoglycan 2 (Spock2), mRNA [NM_052994]	94214	NM_052994	sparc/osteonectin, cwcv and kazal-like domains proteoglycan 2	NM_052994	Mm.153429
A_55_P2136657	0.003	up	1.5755699	Thbs3	Mus musculus thrombospondin 3 (Thbs3), mRNA [NM_013691]	21827	NM_013691	thrombospondin 3	NM_013691	Mm.2114
A_55_P2025820	0.009	up	1.5759348	Plcb4	Mus musculus adult female vagina cDNA, RIKEN full-length enriched library, clone:9930022F01 product:unclassifiable, full insert sequence. [AK036896]	18798	AK036896	phospholipase C, beta 4		Mm.38009
A_55_P2095859	0.044	up	1.5763127	Rdh18-ps	Mus musculus retinol dehydrogenase 18, pseudogene (Rdh18-ps), non-coding RNA [NR_037604]	380674	NR_037604	retinol dehydrogenase 18, pseudogene	NR_037604	Mm.384871
A_55_P1985001	0.020	up	1.5785563	Cirbp	cold inducible RNA binding protein [Source:MGI Symbol;Acc:MGI:893588] [ENSMUST00000054666]	12696	BU840680	cold inducible RNA binding protein		Mm.487902
A_52_P337427	0.012	up	1.5793294	Izumo4	Mus musculus IZUMO family member 4 (Izumo4), mRNA [NM_027829]	71564	NM_027829	IZUMO family member 4	NM_027829	Mm.35802
A_52_P342620	0.006	up	1.57961	Csrnp2	Mus musculus cysteine-serine-rich nuclear protein 2 (Csrnp2), mRNA [NM_153407]	207785	NM_153407	cysteine-serine-rich nuclear protein 2	NM_153407	Mm.294969
A_55_P1989956	0.037	up	1.57978	Lphn2	Mus musculus latrophilin 2 (Lphn2), mRNA [NM_001081298]	99633	NM_001081298	latrophilin 2	NM_001081298	Mm.9776
A_55_P2027112	0.027	up	1.5798206	Rgs3	regulator of G-protein signaling 3 [Source:MGI Symbol;Acc:MGI:1354734] [ENSMUST00000142684]	50780	AK041392	regulator of G-protein signaling 3		Mm.286753
A_52_P506461	0.010	up	1.5802481	Arhgap27	Mus musculus Rho GTPase activating protein 27 (Arhgap27), transcript variant 3, mRNA [NM_183288]	544817	NM_183288	Rho GTPase activating protein 27	NM_183288	Mm.259113
A_55_P1958921	0.013	up	1.5802972	Ankrd29	Mus musculus ankyrin repeat domain 29 (Ankrd29), mRNA [NM_001190371]	225187	NM_001190371	ankyrin repeat domain 29	NM_001190371	Mm.53865
A_51_P381618	0.004	up	1.5806237	Pla1a	Mus musculus phospholipase A1 member A (Pla1a), mRNA [NM_134102]	85031	NM_134102	phospholipase A1 member A	NM_134102	Mm.279805
A_55_P2114143	0.001	up	1.5814	Rsph3a	Mus musculus cDNA clone IMAGE:5371116. [BC110424]	66832	BC110424	radial spoke 3A homolog (Chlamydomonas)		Mm.359991

A_55_P1994447	0.007	up	1.5814736	Sowahd	Mus musculus sosondowah ankyrin repeat domain family member D (Sowahd), mRNA [NM_173779]	245381	NM_173779	sosondowah ankyrin repeat domain family member D	NM_173779	Mm.123697
A_55_P1992889	0.015	up	1.5845213	Serping1	Mus musculus serine (or cysteine) peptidase inhibitor, clade G, member 1 (Serping1), mRNA [NM_009776]	12258	NM_009776	serine (or cysteine) peptidase inhibitor, clade G, member 1	NM_009776	Mm.38888
A_51_P435817	0.024	up	1.5849888	Zbtb8a	Mus musculus zinc finger and BTB domain containing 8a (Zbtb8a), mRNA [NM_028603]	73680	NM_028603	zinc finger and BTB domain containing 8a	NM_028603	Mm.32997
A_55_P2010622	0.005	up	1.5859756	Sec16b	Mus musculus SEC16 homolog B (S. cerevisiae) (Sec16b), transcript variant 1, mRNA [NM_033354]	89867	NM_033354	SEC16 homolog B (S. cerevisiae)	NM_033354	Mm.87114
A_55_P2048119	0.003	up	1.5876263	Slc29a4	Mus musculus solute carrier family 29 (nucleoside transporters), member 4 (Slc29a4), mRNA [NM_146257]	243328	NM_146257	solute carrier family 29 (nucleoside transporters), member 4	NM_146257	Mm.125942
A_51_P268094	0.008	up	1.5882511	Serpine2	Mus musculus serine (or cysteine) peptidase inhibitor, clade E, member 2 (Serpine2), mRNA [NM_009255]	20720	NM_009255	serine (or cysteine) peptidase inhibitor, clade E, member 2	NM_009255	Mm.3093
A_55_P2096324	0.009	up	1.5886954	Camk4	Mus musculus calcium/calmodulin-dependent protein kinase IV (Camk4), mRNA [NM_009793]	12326	NM_009793	calcium/calmodulin-dependent protein kinase IV	NM_009793	Mm.222329
A_55_P2074499	0.013	up	1.589041	Kcp	Mus musculus kielin/chordin-like protein (Kcp), mRNA [NM_001029985]	333088	NM_001029985	kielin/chordin-like protein	NM_001029985	Mm.332961
A_52_P231232	0.003	up	1.5894305	Nanos1	Mus musculus nanos homolog 1 (Drosophila) (Nanos1), mRNA [NM_178421]	332397	NM_178421	nanos homolog 1 (Drosophila)	NM_178421	Mm.186390
A_55_P2180764	0.004	up	1.5903126	Gls	Mus musculus glutaminase (Gls), transcript variant 1, mRNA [NM_001081081]	14660	NM_001081081	glutaminase	NM_001081081	Mm.398608
A_55_P2012815	0.014	up	1.5913291	Acvr2b	Mus musculus activin receptor IIB (Acvr2b), mRNA [NM_007397]	11481	NM_007397	activin receptor IIB	NM_007397	Mm.390239
A_51_P304125	0.010	up	1.5925467	Slc29a2	Mus musculus solute carrier family 29 (nucleoside transporters), member 2 (Slc29a2), mRNA [NM_007854]	13340	NM_007854	solute carrier family 29 (nucleoside transporters), member 2	NM_007854	Mm.4930
A_51_P193189	0.043	up	1.5931162	Slc26a8	solute carrier family 26, member 8 [Source:MGI Symbol;Acc:MGI:2385046] [ENSMUST00000145224]	224661	BC027076	solute carrier family 26, member 8		
A_55_P2077956	0.036	up	1.5937	Klrb1	Mus musculus killer cell lectin-like receptor subfamily B member 1 (Klrb1), mRNA [NM_001099918]	1E+08	NM_001099918	killer cell lectin-like receptor subfamily B member 1	NM_001099918	Mm.66911
A_55_P1953540	0.021	up	1.5940403	Orly	Mus musculus adult male testis cDNA, RIKEN full-length enriched library, clone:4930529H12 product:RIKEN cDNA 4930529H12 gene, full insert sequence. [AK015935]	75204	AK015935	oppositely-transcribed, rearranged locus on the Y		Mm.463004
A_51_P411757	0.004	up	1.5945728	Ankrd13b	Mus musculus ankyrin repeat domain 13b (Ankrd13b), mRNA [NM_172945]	268445	NM_172945	ankyrin repeat domain 13b	NM_172945	Mm.39296
A_51_P319460	0.045	up	1.5946622	Osmr	Mus musculus oncostatin M receptor (Osmr), mRNA [NM_011019]	18414	NM_011019	oncostatin M receptor	NM_011019	Mm.10760
A_51_P140237	0.003	up	1.5955256	Fhl2	Mus musculus four and a half LIM domains 2 (Fhl2), mRNA [NM_010212]	14200	NM_010212	four and a half LIM domains 2	NM_010212	Mm.6799
A_51_P386562	0.048	up	1.5973159	Ebf2	Mus musculus early B cell factor 2 (Ebf2), transcript variant 2, mRNA [NM_010095]	13592	NM_010095	early B cell factor 2	NM_010095	Mm.319947
A_55_P2035255	0.029	up	1.5987228	Skint6	Mus musculus selection and upkeep of intraepithelial T cells 6 (Skint6), mRNA [NM_001103199]	230622	NM_001103199	selection and upkeep of intraepithelial T cells 6	NM_001103199	Mm.291107
A_55_P2325378	0.006	up	1.5989857	Pura	purine rich element binding protein A [Source:MGI Symbol;Acc:MGI:103079] [ENSMUST00000051301]	19290	AK039484	purine rich element binding protein A		Mm.44643
A_51_P284486	0.010	up	1.5994326	Gstm2	Mus musculus glutathione S-transferase, mu 2 (Gstm2), mRNA [NM_008183]	14863	NM_008183	glutathione S-transferase, mu 2	NM_008183	Mm.440086
A_55_P2274592	0.026	up	1.6015161	Wnt7b	Mus musculus wingless-related MMTV integration site 7B (Wnt7b), transcript variant 2, mRNA [NM_001163634]	22422	NM_001163634	wingless-related MMTV integration site 7B	NM_001163634	Mm.306946
A_55_P2044932	0.016	up	1.6016846	Gpr84	Mus musculus G protein-coupled receptor 84 (Gpr84), mRNA [NM_030720]	80910	NM_030720	G protein-coupled receptor 84	NM_030720	Mm.160369
A_51_P395473	0.046	up	1.6019158	Tenc1	Mus musculus tensin like C1 domain-containing phosphatase (Tenc1), mRNA [NM_153533]	209039	NM_153533	tensin like C1 domain-containing phosphatase	NM_153533	Mm.29389
A_55_P2021029	0.001	up	1.6038224	Tigd3	Mus musculus tigger transposable element derived 3 (Tigd3), mRNA [NM_198634]	332359	NM_198634	tigger transposable element derived 3	NM_198634	Mm.329642
A_51_P382512	0.037	up	1.6039506	Zfp418	Mus musculus zinc finger protein 418 (Zfp418), mRNA [NM_146179]	232854	NM_146179	zinc finger protein 418	NM_146179	Mm.184857
A_55_P1977766	0.008	up	1.6041479	Ttc14	Mus musculus tetratricopeptide repeat domain 14 (Ttc14), transcript variant 2, mRNA [NM_025978]	67120	NM_025978	tetratricopeptide repeat domain 14	NM_025978	Mm.275710
A_55_P2000798	0.002	up	1.6043315	Ccdc144b	Mus musculus coiled-coil domain containing 144B (Ccdc144b), mRNA [NM_178418]	241943	NM_178418	coiled-coil domain containing 144B	NM_178418	Mm.269018
A_55_P2100928	0.002	up	1.6051271	Ptgds	Mus musculus prostaglandin D2 synthase (brain) (Ptgds), mRNA [NM_008963]	19215	NM_008963	prostaglandin D2 synthase (brain)	NM_008963	Mm.1008
A_55_P1973945	0.026	up	1.6067803	Krt75	Mus musculus keratin 75 (Krt75), mRNA [NM_133357]	109052	NM_133357	keratin 75	NM_133357	Mm.106868
A_51_P173678	0.033	up	1.6078887	Slc10a6	Mus musculus solute carrier family 10 (sodium/bile acid cotransporter family), member 6 (Slc10a6), mRNA [NM_029415]	75750	NM_029415	solute carrier family 10 (sodium/bile acid cotransporter family), member 6	NM_029415	Mm.7446
A_66_P138289	0.002	up	1.607957	Rdh5	Mus musculus retinol dehydrogenase 5 (Rdh5), mRNA [NM_134006]	19682	NM_134006	retinol dehydrogenase 5	NM_134006	Mm.439856
A_55_P2040245	0.008	up	1.6100805	Piezo2	Mus musculus piezo-type mechanosensitive ion channel component 2 (Piezo2), mRNA [NM_001039485]	667742	NM_001039485	piezo-type mechanosensitive ion channel component 2	NM_001039485	Mm.158720
A_51_P339540	0.003	up	1.6106753	Cdkn1c	Mus musculus cyclin-dependent kinase inhibitor 1C (P57) (Cdkn1c), transcript variant 2, mRNA [NM_009876]	12577	NM_009876	cyclin-dependent kinase inhibitor 1C (P57)	NM_009876	Mm.168789
A_55_P2097630	0.035	up	1.6107734	Csmd1	Mus musculus 16 days embryo head cDNA, RIKEN full-length enriched library, clone:C130029B03 product:unclassifiable, full insert sequence. [AK048002]	94109	AK048002	CUB and Sushi multiple domains 1		
A_51_P285097	0.005	up	1.6108631	Wdr38	WD repeat domain 38 [Source:MGI Symbol;Acc:MGI:1923896] [ENSMUST00000039535]	76646	AK077027	WD repeat domain 38		Mm.358777

A_51_P323531	0.011	up	1.6110903	Fam71e1	Mus musculus family with sequence similarity 71, member E1 (Fam71e1), mRNA [NM_028169]	75538	NM_028169	family with sequence similarity 71, member E1	NM_028169	Mm.258841
A_55_P2162204	0.026	up	1.6111579	Kctd15	Mus musculus potassium channel tetramerisation domain containing 15 (Kctd15), mRNA [NM_146188]	233107	NM_146188	potassium channel tetramerisation domain containing 15	NM_146188	Mm.486198
A_55_P2146500	0.039	up	1.6118026	Ccdc107	coiled-coil domain containing 107 [Source:MGI Symbol;Acc:MGI:1913423] [ENSMUST00000107922]	622404		coiled-coil domain containing 107		
A_55_P2071691	0.010	up	1.6119266	Kank3	Mus musculus KN motif and ankyrin repeat domains 3 (Kank3), mRNA [NM_030697]	80880	NM_030697	KN motif and ankyrin repeat domains 3	NM_030697	Mm.196330
A_51_P308597	0.033	up	1.6126976	Gcm2	Mus musculus glial cells missing homolog 2 (Drosophila) (Gcm2), mRNA [NM_008104]	107889	NM_008104	glial cells missing homolog 2 (Drosophila)	NM_008104	Mm.1399
A_55_P2420873	0.002	up	1.6149215	Igip	Mus musculus IgA inducing protein (Igip), mRNA [NM_001267796]	109169	NM_001267796	IgA inducing protein	NM_001267796	Mm.426423
A_55_P1956534	0.005	up	1.6156214	Dach2	Mus musculus dachshund 2 (Drosophila) (Dach2), transcript variant 2, mRNA [NM_033605]	93837	NM_033605	dachshund 2 (Drosophila)	NM_033605	Mm.79760
A_55_P2080531	0.040	up	1.6165721	Poln	Mus musculus DNA polymerase N (Poln), mRNA [NM_181857]	272158	NM_181857	DNA polymerase N	NM_181857	Mm.319286
A_51_P115178	0.013	up	1.6165777	Scara3	Mus musculus scavenger receptor class A, member 3 (Scara3), mRNA [NM_172604]	219151	NM_172604	scavenger receptor class A, member 3	NM_172604	Mm.344095
A_52_P188678	0.013	up	1.617176	Pvrl2	Mus musculus poliovirus receptor-related 2 (Pvrl2), transcript variant 2, mRNA [NM_001159724]	19294	NM_001159724	poliovirus receptor-related 2	NM_001159724	Mm.4341
A_51_P252859	0.021	up	1.6190504	Cyr61	Mus musculus cysteine rich protein 61 (Cyr61), mRNA [NM_010516]	16007	NM_010516	cysteine rich protein 61	NM_010516	Mm.1231
A_51_P250123	0.018	up	1.6198678	Zfp763	Mus musculus zinc finger protein 763 (Zfp763), mRNA [NM_028543]	73451	NM_028543	zinc finger protein 763	NM_028543	Mm.129453
A_55_P1981869	0.034	up	1.6202233	Ercc6l2	Mus musculus excision repair cross-complementing rodent repair deficiency, complementation group 6 like 2 (Ercc6l2), transcript variant 2, mRNA [NM_023507]	76251	NM_023507	excision repair cross-complementing rodent repair deficiency, complementation group 6 like 2	NM_023507	Mm.186610
A_51_P302249	0.011	up	1.6207916	Armc12	Mus musculus armadillo repeat containing 12 (Armc12), mRNA [NM_026290]	67645	NM_026290	armadillo repeat containing 12	NM_026290	Mm.46170
A_51_P308844	0.046	up	1.621572	Nrn1	Mus musculus neuritin 1 (Nrn1), mRNA [NM_153529]	68404	NM_153529	neuritin 1	NM_153529	Mm.232930
A_55_P1952628	0.049	up	1.6217355	Dpys	Mus musculus dihydropyrimidinase (Dpys), transcript variant 1, mRNA [NM_022722]	64705	NM_022722	dihydropyrimidinase	NM_022722	Mm.275974
A_55_P2063686	0.008	up	1.6223044	Pdxp	Mus musculus pyridoxal (pyridoxine, vitamin B6) phosphatase (Pdxp), mRNA [NM_020271]	57028	NM_020271	pyridoxal (pyridoxine, vitamin B6) phosphatase	NM_020271	Mm.263169
A_55_P2078955	0.015	up	1.6227481	Aqp8	Mus musculus aquaporin 8 (Aqp8), transcript variant 1, mRNA [NM_007474]	11833	NM_007474	aquaporin 8	NM_007474	Mm.273175
A_55_P2043237	0.011	up	1.6231254	Rpe65	Mus musculus retinal pigment epithelium 65 (Rpe65), mRNA [NM_029987]	19892	NM_029987	retinal pigment epithelium 65	NM_029987	Mm.131708
A_52_P108447	0.009	up	1.6238179	Glis2	Mus musculus GLIS family zinc finger 2 (Glis2), mRNA [NM_031184]	83396	NM_031184	GLIS family zinc finger 2	NM_031184	Mm.134072
A_55_P2124641	0.042	up	1.6243048	Gxylt2	glucoside xylosyltransferase 2 [Source:MGI Symbol;Acc:MGI:2682940] [ENSMUST00000141333]	232313	AK144790	glucoside xylosyltransferase 2		Mm.272037
A_51_P463860	0.018	up	1.6244735	Fam161b	Mus musculus family with sequence similarity 161, member B (Fam161b), mRNA [NM_172581]	217705	NM_172581	family with sequence similarity 161, member B	NM_172581	Mm.259468
A_55_P2173188	0.015	up	1.6245418	Kank3	Mus musculus KN motif and ankyrin repeat domains 3 (Kank3), mRNA [NM_030697]	80880	NM_030697	KN motif and ankyrin repeat domains 3	NM_030697	Mm.196330
A_55_P1963863	0.013	up	1.6271733	Arhgef17	Mus musculus Rho guanine nucleotide exchange factor (GEF) 17 (Arhgef17), mRNA [NM_001081116]	207212	NM_001081116	Rho guanine nucleotide exchange factor (GEF) 17	NM_001081116	Mm.29954
A_55_P2075884	0.024	up	1.6293719	Senp8	Mus musculus SUMO/sentrin specific peptidase 8 (Senp8), transcript variant 1, mRNA [NM_001172068]	71599	NM_001172068	SUMO/sentrin specific peptidase 8	NM_001172068	Mm.279070
A_55_P2128511	0.012	up	1.6294115	Ociad2	Mus musculus OCIA domain containing 2 (Ociad2), mRNA [NM_026950]	433904	NM_026950	OCIA domain containing 2	NM_026950	Mm.274892
A_51_P392214	0.024	up	1.6296799	Pcnxl4	Mus musculus pecanex-like 4 (Drosophila) (Pcnxl4), mRNA [NM_026327]	67708	NM_026327	pecanex-like 4 (Drosophila)	NM_026327	Mm.11233
A_52_P559975	0.006	up	1.6298984	Cxcr2	Mus musculus chemokine (C-X-C motif) receptor 2 (Cxcr2), mRNA [NM_009909]	12765	NM_009909	chemokine (C-X-C motif) receptor 2	NM_009909	Mm.234466
A_51_P331827	0.009	up	1.6313721	Slc25a41	Mus musculus solute carrier family 25, member 41 (Slc25a41), mRNA [NM_175333]	103775	NM_175333	solute carrier family 25, member 41	NM_175333	Mm.33647
A_52_P391110	0.012	up	1.632166	Ryr1	Mus musculus ryanodine receptor 1, skeletal muscle (Ryr1), mRNA [NM_009109]	20190	NM_009109	ryanodine receptor 1, skeletal muscle	NM_009109	Mm.439745
A_55_P2010681	0.041	up	1.6324892	Kif17	kinesin family member 17 [Source:MGI Symbol;Acc:MGI:1098229] [ENSMUST00000105820]	16559	AK046890	kinesin family member 17		
A_65_P10029	0.041	up	1.6335613	Prdm2	Mus musculus PR domain containing 2, with ZNF domain (Prdm2), transcript variant 1, mRNA [NM_001081355]	110593	NM_001081355	PR domain containing 2, with ZNF domain	NM_001081355	Mm.332020
A_55_P2107025	0.002	up	1.6336709	Flad1	RFad1, flavin adenine dinucleotide synthetase, homolog (yeast) [Source:MGI Symbol;Acc:MGI:2443030] [ENSMUST00000050398]	319945	AK044501	RFad1, flavin adenine dinucleotide synthetase, homolog (yeast)		Mm.258142
A_51_P475378	0.020	up	1.637103	Echdc3	Mus musculus enoyl Coenzyme A hydratase domain containing 3 (Echdc3), mRNA [NM_024208]	67856	NM_024208	enoyl Coenzyme A hydratase domain containing 3	NM_024208	Mm.38342
A_55_P1973159	0.004	up	1.6382275	Serpinf1	Mus musculus serine (or cysteine) peptidase inhibitor, clade F, member 1 (Serpinf1), mRNA [NM_011340]	20317	NM_011340	serine (or cysteine) peptidase inhibitor, clade F, member 1	NM_011340	Mm.2044
A_52_P651948	0.049	up	1.638657	Fam229b	Mus musculus family with sequence similarity 229, member B (Fam229b), mRNA [NM_183254]	66337	NM_183254	family with sequence similarity 229, member B	NM_183254	Mm.45187

A_55_P2153783	0.015	up	1.6388525	Fmo1	Mus musculus flavin containing monooxygenase 1 (Fmo1), mRNA [NM_010231]	14261	NM_010231	flavin containing monooxygenase 1	NM_010231	Mm.976
A_51_P250001	0.008	up	1.6390827	H1fnt	Mus musculus H1 histone family, member N, testis-specific (H1fnt), mRNA [NM_027304]	70069	NM_027304	H1 histone family, member N, testis-specific	NM_027304	Mm.107718
A_51_P376238	0.020	up	1.6394316	Serping1	Mus musculus serine (or cysteine) peptidase inhibitor, clade G, member 1 (Serping1), mRNA [NM_009776]	12258	NM_009776	serine (or cysteine) peptidase inhibitor, clade G, member 1	NM_009776	Mm.38888
A_51_P169255	0.032	up	1.6395781	Olfr190	Mus musculus olfactory receptor 190 (Olfr190), mRNA [NM_146397]	258392	NM_146397	olfactory receptor 190	NM_146397	Mm.377476
A_51_P271153	0.029	up	1.6398549	Olfr166	Mus musculus olfactory receptor 166 (Olfr166), mRNA [NM_147068]	259071	NM_147068	olfactory receptor 166	NM_147068	Mm.377822
A_55_P2167461	0.002	up	1.6401871	Homer3	Mus musculus NOD-derived CD11c +ve dendritic cells cDNA, RIKEN full-length enriched library, clone:F630230H03 product:unclassifiable, full insert sequence. [AK155463]	26558	AK155463	homer homolog 3 (Drosophila)		Mm.440659
A_55_P2147126	0.015	up	1.6410222	Zfp712	Mus musculus zinc finger protein 712 (Zfp712), mRNA [NM_001166218]	78251	NM_001166218	zinc finger protein 712	NM_001166218	Mm.297247
A_51_P103222	0.007	up	1.6426587	Slc39a4	Mus musculus solute carrier family 39 (zinc transporter), member 4 (Slc39a4), mRNA [NM_028064]	72027	NM_028064	solute carrier family 39 (zinc transporter), member 4	NM_028064	Mm.276829
A_51_P347529	0.042	up	1.6469682	Nek5	Mus musculus NIMA (never in mitosis gene a)-related expressed kinase 5 (Nek5), mRNA [NM_177898]	330721	NM_177898	NIMA (never in mitosis gene a)-related expressed kinase 5	NM_177898	Mm.113940
A_51_P232399	0.003	up	1.6471031	Acy3	Mus musculus aspartoacylase (aminoacylase) 3 (Acy3), mRNA [NM_027857]	71670	NM_027857	aspartoacylase (aminoacylase) 3	NM_027857	Mm.29735
A_55_P2043627	0.042	up	1.6487694	Fam89a	Mus musculus family with sequence similarity 89, member A (Fam89a), mRNA [NM_001081120]	69627	NM_001081120	family with sequence similarity 89, member A	NM_001081120	Mm.271600
A_55_P2089799	0.004	up	1.6531175	Atp6v1g2	ATPase, H+ transporting, lysosomal V1 subunit G2 [Source:MGI Symbol;Acc:MGI:1913487] [ENSMUST00000143289]	66237	AK077802	ATPase, H+ transporting, lysosomal V1 subunit G2		Mm.300795
A_51_P158814	0.006	up	1.6532267	Marveld1	Mus musculus adult male corpus striatum cDNA, RIKEN full-length enriched library, clone:C030005I21 product:HYPOTHETICAL 18.9 KDA PROTEIN homolog [Homo sapiens], full insert sequence. [AK081212]	277010	AK081212	MARVEL (membrane-associating) domain containing 1		Mm.206250
A_52_P587948	0.003	up	1.6543875	Zfp11	Mus musculus zinc finger protein 11 (Zfp11), mRNA [NM_172462]	22648	NM_172462	zinc finger protein 11	NM_172462	Mm.135067
A_55_P1968170	0.022	up	1.6551336	Vmn1r139	Mus musculus vomeronasal 1 receptor 139 (Vmn1r139), mRNA [NM_001166748]	667444	NM_001166748	vomeronasal 1 receptor 139	NM_001166748	Mm.484972
A_55_P2079855	0.035	up	1.655453	Prl6a1	Mus musculus prolactin family 6, subfamily a, member 1 (Prl6a1), mRNA [NM_011166]	19111	NM_011166	prolactin family 6, subfamily a, member 1	NM_011166	Mm.7512
A_51_P313503	0.026	up	1.6556039	Olfr577	Mus musculus olfactory receptor 577 (Olfr577), mRNA [NM_147109]	259113	NM_147109	olfactory receptor 577	NM_147109	Mm.377854
A_52_P466147	0.003	up	1.6560128	Rarres2	Mus musculus retinoic acid receptor responder (tazarotene induced) 2 (Rarres2), mRNA [NM_027852]	71660	NM_027852	retinoic acid receptor responder (tazarotene induced) 2	NM_027852	Mm.28231
A_55_P2101696	0.006	up	1.6580591	Gnat2	Mus musculus guanine nucleotide binding protein, alpha transducing 2 (Gnat2), mRNA [NM_008141]	14686	NM_008141	guanine nucleotide binding protein, alpha transducing 2	NM_008141	Mm.439652
A_52_P650387	0.020	up	1.6583284	Ccnjl	Mus musculus cyclin J-like (Ccnjl), mRNA [NM_001045530]	380694	NM_001045530	cyclin J-like	NM_001045530	Mm.247595
A_52_P550932	0.003	up	1.658878	H1f0	Mus musculus H1 histone family, member 0 (H1f0), mRNA [NM_008197]	14958	NM_008197	H1 histone family, member 0	NM_008197	Mm.24350
A_55_P2057821	0.003	up	1.6590683	Golga1	Mus musculus golgi autoantigen, golgin subfamily a, 1 (Golga1), mRNA [NM_029793]	76899	NM_029793	golgi autoantigen, golgin subfamily a, 1	NM_029793	Mm.131098
A_55_P2090429	0.005	up	1.6598822	Dync1i1	Mus musculus dynein cytoplasmic 1 intermediate chain 1 (Dync1i1), transcript variant 1, mRNA [NM_010063]	13426	NM_010063	dynein cytoplasmic 1 intermediate chain 1	NM_010063	Mm.20893
A_55_P1965989	0.015	up	1.6612097	Zfp329	Mus musculus zinc finger protein 329 (Zfp329), mRNA [NM_026046]	67230	NM_026046	zinc finger protein 329	NM_026046	Mm.333574
A_55_P2102941	0.042	up	1.6617292	Olfr351	Mus musculus olfactory receptor 351 (Olfr351), mRNA [NM_146942]	258944	NM_146942	olfactory receptor 351	NM_146942	Mm.377763
A_55_P2122075	0.014	up	1.6628006	Pdcd1lg2	Mus musculus butyrophilin-like protein (Btdc) mRNA, complete cds. [AF142780]	58205	AF142780	programmed cell death 1 ligand 2		Mm.116737
A_55_P2015485	0.007	up	1.6631039	Ccdc19	Mus musculus coiled-coil domain containing 19 (Ccdc19), mRNA [NM_027972]	71870	NM_027972	coiled-coil domain containing 19	NM_027972	Mm.78373
A_55_P2084177	0.010	up	1.6638758	Klhl34	Mus musculus kelch-like 34 (Klhl34), mRNA [NM_001081667]	245683	NM_001081667	kelch-like 34	NM_001081667	Mm.295846
A_55_P2325663	0.010	up	1.6641272	Cers3	Mus musculus ceramide synthase 3 (Cers3), mRNA [NM_001164201]	545975	NM_001164201	ceramide synthase 3	NM_001164201	Mm.380104
A_55_P2033490	0.022	up	1.664834	Ece2	Mus musculus adult male medulla oblongata cDNA, RIKEN full-length enriched library, clone:6330509A19 product:ENDOTHELIN CONVERTING ENZYME-2 homolog [Mus musculus], full insert sequence. [AK031951]	107522	AK031951	endothelin converting enzyme 2		Mm.263319
A_55_P2109122	0.008	up	1.6661425	Hist1h2bc	Mus musculus histone cluster 1, H2bc (Hist1h2bc), mRNA [NM_023422]	68024	NM_023422	histone cluster 1, H2bc	NM_023422	Mm.261676
A_55_P2149500	0.044	up	1.6663383	Kifc2	Mus musculus kinesin family member C2 (Kifc2), mRNA [NM_010630]	16581	NM_010630	kinesin family member C2	NM_010630	Mm.378950
A_55_P1981719	0.018	up	1.6683728	Rreb1	Mus musculus ras responsive element binding protein 1 (Rreb1), transcript variant 5, non-coding RNA [NR_033615]	68750	NR_033615	ras responsive element binding protein 1	NR_033615	Mm.491109
A_55_P1976200	0.008	up	1.6707114	Lmtk3	Mus musculus lemur tyrosine kinase 3 (Lmtk3), mRNA [NM_001005511]	381983	NM_001005511	lemur tyrosine kinase 3	NM_001005511	Mm.44928
A_55_P1962084	0.009	up	1.6712534	Hist2h2aa2	Mus musculus histone cluster 2, H2aa2 (Hist2h2aa2), mRNA [NM_178212]	319192	NM_178212	histone cluster 2, H2aa2	NM_178212	Mm.5220
A_55_P1960298	0.002	up	1.6761812	Tmem80	Mus musculus transmembrane protein 80 (Tmem80), transcript variant 1, mRNA [NM_027797]	71448	NM_027797	transmembrane protein 80	NM_027797	Mm.248960
A_51_P234864	0.001	up	1.6765678	Sfxn5	Mus musculus sideroflexin 5 (Sfxn5), mRNA [NM_178639]	94282	NM_178639	sideroflexin 5	NM_178639	Mm.121485

A_55_P2056958	0.007	up	1.681662	Cul9	Mus musculus cullin 9 (Cul9), mRNA [NM_001081335]	78309	NM_001081335	cullin 9	NM_001081335	Mm.329076
A_66_P139698	0.016	up	1.6820395	Slc25a53	Mus musculus solute carrier family 25, member 53 (Slc25a53), transcript variant 1, mRNA [NM_001082412]	67062	NM_001082412	solute carrier family 25, member 53	NM_001082412	Mm.276518
A_52_P647408	0.006	up	1.6863321	Gspt2	G1 to S phase transition 2 [Source:MGI Symbol;Acc:MGI:1316727] [ENSMUST00000137033]	14853	AK050859	G1 to S phase transition 2		Mm.487061
A_51_P268069	0.008	up	1.6866584	Six1	Mus musculus sine oculis-related homeobox 1 (Six1), mRNA [NM_009189]	20471	NM_009189	sine oculis-related homeobox 1	NM_009189	Mm.4645
A_51_P484054	0.003	up	1.6882596	F7	Mus musculus coagulation factor VII (F7), mRNA [NM_010172]	14068	NM_010172	coagulation factor VII	NM_010172	Mm.4827
A_55_P2060502	0.007	up	1.6884923	Zfp563	Mus musculus zinc finger protein 563 (Zfp563), mRNA [NM_001024950]	240068	NM_001024950	zinc finger protein 563	NM_001024950	Mm.121392
A_55_P2031671	0.012	up	1.689844	Gstm6	Mus musculus glutathione S-transferase, mu 6 (Gstm6), mRNA [NM_008184]	14867	NM_008184	glutathione S-transferase, mu 6	NM_008184	Mm.458154
A_51_P494822	0.002	up	1.6926062	Stag3	Mus musculus stromal antigen 3 (Stag3), mRNA [NM_016964]	50878	NM_016964	stromal antigen 3	NM_016964	Mm.285609
A_55_P1971734	0.038	up	1.6941823	Reck	Mus musculus reversion-inducing-cysteine-rich protein with kazal motifs (Reck), mRNA [NM_016678]	53614	NM_016678	reversion-inducing-cysteine-rich protein with kazal motifs	NM_016678	Mm.331573
A_51_P323812	0.001	up	1.6947131	Slc6a12	Mus musculus solute carrier family 6 (neurotransmitter transporter, betaine/GABA), member 12 (Slc6a12), mRNA [NM_133661]	14411	NM_133661	solute carrier family 6 (neurotransmitter transporter, betaine/GABA), member 12	NM_133661	Mm.274506
A_51_P173601	0.045	up	1.6951581	Bcam	Mus musculus basal cell adhesion molecule (Bcam), mRNA [NM_020486]	57278	NM_020486	basal cell adhesion molecule	NM_020486	Mm.29236
A_52_P347942	0.015	up	1.6967124	Olfr1384	Mus musculus olfactory receptor 1384 (Olfr1384), mRNA [NM_146472]	258464	NM_146472	olfactory receptor 1384	NM_146472	Mm.271389
A_55_P2183508	0.023	up	1.6975597	Hist1h1d	Mus musculus histone cluster 1, H1d (Hist1h1d), mRNA [NM_145713]	14957	NM_145713	histone cluster 1, H1d	NM_145713	Mm.247046
A_55_P2094494	0.005	up	1.6985368	Tmem150b	Mus musculus transmembrane protein 150B (Tmem150b), transcript variant 1, mRNA [NM_001142792]	330460	NM_001142792	transmembrane protein 150B	NM_001142792	Mm.361851
A_55_P2078633	0.011	up	1.7002507	C4b	Mus musculus complement component 4B (Chido blood group) (C4b), mRNA [NM_009780]	12268	NM_009780	complement component 4B (Chido blood group)	NM_009780	Mm.477109
A_51_P514913	0.031	up	1.7012333	Rsad1	Mus musculus radical S-adenosyl methionine domain containing 1 (Rsad1), mRNA [NM_001013381]	237926	NM_001013381	radical S-adenosyl methionine domain containing 1	NM_001013381	Mm.342275
A_55_P2006525	0.007	up	1.7013103	Adamtsl4	Mus musculus ADAMTS-like 4 (Adamtsl4), mRNA [NM_144899]	229595	NM_144899	ADAMTS-like 4	NM_144899	Mm.490574
A_51_P164630	0.035	up	1.7018064	Fitm1	Mus musculus fat storage-inducing transmembrane protein 1 (Fitm1), mRNA [NM_026808]	68680	NM_026808	fat storage-inducing transmembrane protein 1	NM_026808	Mm.36758
A_51_P422335	0.005	up	1.7022303	Zfp420	Mus musculus zinc finger protein 420 (Zfp420), mRNA [NM_172740]	233058	NM_172740	zinc finger protein 420	NM_172740	Mm.297657
A_55_P2031547	0.041	up	1.7044076	Vmn2r121	Mus musculus vomeronasal 2, receptor 121 (Vmn2r121), mRNA [NM_001100616]	1E+08	NM_001100616	vomeronasal 2, receptor 121	NM_001100616	Mm.388852
A_51_P446085	0.002	up	1.7049844	Map10	Mus musculus microtubule-associated protein 10 (Map10), mRNA [NM_028908]	74393	NM_028908	microtubule-associated protein 10	NM_028908	Mm.41709
A_55_P2153560	0.000	up	1.7062864	Zkscan3	Mus musculus adult male liver tumor cDNA, RIKEN full-length enriched library, clone:C730004D03 product:SCAN-KRAB-zinc finger gene 1, full insert sequence. [AK050028]	72739	AK050028	zinc finger with KRAB and SCAN domains 3		Mm.296071
A_51_P114462	0.019	up	1.706544	Ccl17	Mus musculus chemokine (C-C motif) ligand 17 (Ccl17), mRNA [NM_011332]	20295	NM_011332	chemokine (C-C motif) ligand 17	NM_011332	Mm.41988
A_55_P2139501	0.038	up	1.706737	Alk	Mus musculus anaplastic lymphoma kinase (Alk), mRNA [NM_007439]	11682	NM_007439	anaplastic lymphoma kinase	NM_007439	Mm.311854
A_51_P163173	0.000	up	1.7092553	Rbm12b1	Mus musculus RNA binding motif protein 12 B1 (Rbm12b1), mRNA [NM_028226]	72397	NM_028226	RNA binding motif protein 12 B1	NM_028226	Mm.430709
A_51_P394814	0.008	up	1.7105867	Svep1	Mus musculus sushi, von Willebrand factor type A, EGF and pentraxin domain containing 1 (Svep1), mRNA [NM_022814]	64817	NM_022814	sushi, von Willebrand factor type A, EGF and pentraxin domain containing 1	NM_022814	Mm.261024
A_55_P2052281	0.013	up	1.7131	Rnf208	Mus musculus ring finger protein 208 (Rnf208), mRNA [NM_176834]	68846	NM_176834	ring finger protein 208	NM_176834	Mm.24777
A_51_P509263	0.001	up	1.7143456	Hoxa7	Mus musculus homeobox A7 (Hoxa7), mRNA [NM_010455]	15404	NM_010455	homeobox A7	NM_010455	Mm.294826
A_52_P300730	0.001	up	1.7146486	Hmga2	Mus musculus high mobility group AT-hook 2 (Hmga2), mRNA [NM_010441]	15364	NM_010441	high mobility group AT-hook 2	NM_010441	Mm.157190
A_52_P668478	0.017	up	1.7155323	Rhbdd2	Mus musculus rhomboid domain containing 2 (Rhbdd2), mRNA [NM_146002]	215160	NM_146002	rhomboid domain containing 2	NM_146002	Mm.295438
A_51_P283563	0.001	up	1.7168102	Zbtb26	Mus musculus zinc finger and BTB domain containing 26 (Zbtb26), mRNA [NM_199025]	320633	NM_199025	zinc finger and BTB domain containing 26	NM_199025	Mm.383299
A_55_P2108943	0.019	up	1.7201277	Ccr6	Mus musculus chemokine (C-C motif) receptor 6 (Ccr6), transcript variant 1, mRNA [NM_009835]	12458	NM_009835	chemokine (C-C motif) receptor 6	NM_009835	Mm.8007
A_55_P2394308	0.029	up	1.7202802	Fst	Mus musculus follistatin (Fst), mRNA [NM_008046]	14313	NM_008046	follistatin	NM_008046	Mm.4913
A_52_P114620	0.015	up	1.7205883	Elmo3	Mus musculus engulfment and cell motility 3 (Elmo3), mRNA [NM_172760]	234683	NM_172760	engulfment and cell motility 3	NM_172760	Mm.237966
A_51_P353882	0.000	up	1.7209328	Zfp580	Mus musculus zinc finger protein 580 (Zfp580), mRNA [NM_026900]	68992	NM_026900	zinc finger protein 580	NM_026900	Mm.41484
A_55_P2008889	0.000	up	1.721267	Tmsb15b1	Mus musculus thymosin beta 15b1 (Tmsb15b1), mRNA [NM_001081983]	666244	NM_001081983	thymosin beta 15b1	NM_001081983	Mm.381904
A_55_P2005060	0.004	up	1.7216449	Ddx43	Mus musculus DEAD (Asp-Glu-Ala-Asp) box polypeptide 43 (Ddx43), mRNA [NM_001191044]	1E+08	NM_001191044	DEAD (Asp-Glu-Ala-Asp) box polypeptide 43	NM_001191044	Mm.158166
A_55_P2106434	0.038	up	1.7219436	Zfp114	Mus musculus zinc finger protein 114 (Zfp114), mRNA [NM_001029933]	232966	NM_001029933	zinc finger protein 114	NM_001029933	Mm.435485
A_52_P390913	0.043	up	1.7243291	Ccdc96	Mus musculus coiled-coil domain containing 96 (Ccdc96), mRNA [NM_025725]	66717	NM_025725	coiled-coil domain containing 96	NM_025725	Mm.42368
A_55_P2074766	0.032	up	1.7272311	Mug2	Mus musculus murinoglobulin 2 (Mug2), mRNA [NM_008646]	17837	NM_008646	murinoglobulin 2	NM_008646	Mm.479223

A_65_P09657	0.000	up	1.7308793	Zfp213	Mus musculus zinc finger protein 213 (Zfp213), mRNA [NM_001033496]	449521	NM_001033496	zinc finger protein 213	NM_001033496	Mm.268582
A_55_P2043921	0.032	up	1.7336086	Hmga2	Mus musculus high mobility group AT-hook 2 (Hmga2), mRNA [NM_010441]	15364	NM_010441	high mobility group AT-hook 2	NM_010441	Mm.157190
A_66_P120995	0.024	up	1.7345619	Htr2a	Mus musculus 5-hydroxytryptamine (serotonin) receptor 2A (Htr2a), mRNA [NM_172812]	15558	NM_172812	5-hydroxytryptamine (serotonin) receptor 2A	NM_172812	Mm.214351
A_55_P1953836	0.010	up	1.7373196	Pld5	Mus musculus phospholipase D family, member 5 (Pld5), transcript variant 1, mRNA [NM_176916]	319455	NM_176916	phospholipase D family, member 5	NM_176916	Mm.338001
A_51_P344461	0.010	up	1.7435691	Prrt2	Mus musculus proline-rich transmembrane protein 2 (Prrt2), mRNA [NM_001102563]	69017	NM_001102563	proline-rich transmembrane protein 2	NM_001102563	Mm.392047
A_51_P219385	0.002	up	1.7441994	Six5	Mus musculus sine oculis-related homeobox 5 (Six5), mRNA [NM_011383]	20475	NM_011383	sine oculis-related homeobox 5	NM_011383	Mm.3410
A_51_P337708	0.000	up	1.7468349	Ovgp1	Mus musculus oviductal glycoprotein 1 (Ovgp1), mRNA [NM_007696]	12659	NM_007696	oviductal glycoprotein 1	NM_007696	Mm.431947
A_51_P317181	0.043	up	1.7471954	Pcdhb21	Mus musculus protocadherin beta 21 (Pcdhb21), mRNA [NM_053146]	93892	NM_053146	protocadherin beta 21	NM_053146	Mm.348053
A_51_P372099	0.003	up	1.7499465	Tarm1	Mus musculus T cell-interacting, activating receptor on myeloid cells 1 (Tarm1), mRNA [NM_177363]	245126	NM_177363	T cell-interacting, activating receptor on myeloid cells 1	NM_177363	Mm.245747
A_51_P490700	0.013	up	1.7505082	Ccdc40	Mus musculus coiled-coil domain containing 40 (Ccdc40), mRNA [NM_175430]	207607	NM_175430	coiled-coil domain containing 40	NM_175430	Mm.189885
A_55_P2239317	0.000	up	1.7525784	Afap1l1	Mus musculus actin filament associated protein 1-like 1 (Afap1l1), mRNA [NM_178928]	106877	NM_178928	actin filament associated protein 1-like 1	NM_178928	Mm.26632
A_55_P2075213	0.027	up	1.7539331	Kbtbd11	Mus musculus kelch repeat and BTB (POZ) domain containing 11 (Kbtbd11), mRNA [NM_029116]	74901	NM_029116	kelch repeat and BTB (POZ) domain containing 11	NM_029116	Mm.46675
A_51_P232748	0.017	up	1.7561619	Plxnb3	Mus musculus plexin B3 (Plxnb3), mRNA [NM_019587]	140571	NM_019587	plexin B3	NM_019587	Mm.275600
A_51_P419117	0.002	up	1.7575226	Rab15	Mus musculus RAB15, member RAS oncogene family (Rab15), mRNA [NM_134050]	104886	NM_134050	RAB15, member RAS oncogene family	NM_134050	Mm.172847
A_51_P431636	0.013	up	1.7604516	F2rl3	Mus musculus coagulation factor II (thrombin) receptor-like 3 (F2rl3), mRNA [NM_007975]	14065	NM_007975	coagulation factor II (thrombin) receptor-like 3	NM_007975	Mm.12948
A_55_P2051787	0.035	up	1.7613535	Gbx1	Mus musculus gastrulation brain homeobox 1 (Gbx1), mRNA [NM_015739]	231044	NM_015739	gastrulation brain homeobox 1	NM_015739	Mm.246596
A_55_P1972099	0.007	up	1.7622433	Acsm3	Mus musculus adult male kidney cDNA, RIKEN full-length enriched library, clone:F530003P09 product:SA rat hypertension-associated homolog, full insert sequence. [AK143946]	20216	AK143946	acyl-CoA synthetase medium-chain family member 3		Mm.334199
A_55_P2088765	0.036	up	1.7701905	Sox5	SRY-box containing gene 5 [Source:MGI Symbol;Acc:MGI:98367] [ENSMUST00000111746]	20678	AK015212	SRY-box containing gene 5		
A_55_P2054285	0.015	up	1.7752758	Fam178b	Mus musculus family with sequence similarity 178, member B (Fam178b), transcript variant C, mRNA [NM_201365]	381337	NM_201365	family with sequence similarity 178, member B	NM_201365	Mm.17413
A_51_P321341	0.004	up	1.777934	Sult1a1	Mus musculus sulfotransferase family 1A, phenol-preferring, member 1 (Sult1a1), mRNA [NM_133670]	20887	NM_133670	sulfotransferase family 1A, phenol-preferring, member 1	NM_133670	Mm.368982
A_51_P122452	0.035	up	1.7792557	Olfr1511	Mus musculus olfactory receptor 1511 (Olfr1511), mRNA [NM_146271]	258268	NM_146271	olfactory receptor 1511	NM_146271	Mm.377402
A_55_P2179378	0.000	up	1.7838362	Zkscan8	Mus musculus zinc finger with KRAB and SCAN domains 8 (Zkscan8), transcript variant 1, mRNA [NM_139141]	93681	NM_139141	zinc finger with KRAB and SCAN domains 8	NM_139141	Mm.290601
A_51_P285077	0.013	up	1.7839278	Hhatl	Mus musculus hedgehog acyltransferase-like (Hhatl), transcript variant 1, mRNA [NM_029095]	74770	NM_029095	hedgehog acyltransferase-like	NM_029095	Mm.44195
A_66_P130100	0.009	up	1.7850319	Rab23	Mus musculus RAB23, member RAS oncogene family (Rab23), transcript variant 1, mRNA [NM_008999]	19335	NM_008999	RAB23, member RAS oncogene family	NM_008999	Mm.86744
A_51_P136441	0.015	up	1.7872701	Nudt12	Mus musculus nudix (nucleoside diphosphate linked moiety X)-type motif 12 (Nudt12), mRNA [NM_026497]	67993	NM_026497	nudix (nucleoside diphosphate linked moiety X)-type motif 12	NM_026497	Mm.36507
A_52_P255849	0.027	up	1.7875633	Fam57a	family with sequence similarity 57, member A [Source:MGI Symbol;Acc:MGI:2151840] [ENSMUST00000169560]	116972	AK160183	family with sequence similarity 57, member A		Mm.188413
A_66_P139703	0.029	up	1.7894886	Wnt4	Mus musculus wingless-related MMTV integration site 4 (Wnt4), mRNA [NM_009523]	22417	NM_009523	wingless-related MMTV integration site 4	NM_009523	Mm.20355
A_55_P2005475	0.001	up	1.7910314	Sult1a1	Mus musculus sulfotransferase family 1A, phenol-preferring, member 1 (Sult1a1), mRNA [NM_133670]	20887	NM_133670	sulfotransferase family 1A, phenol-preferring, member 1	NM_133670	Mm.368982
A_55_P1958320	0.050	up	1.7956915	Vmn2r113	Mus musculus vomeronasal 2, receptor 113 (Vmn2r113), mRNA [NM_001104578]	434701	NM_001104578	vomeronasal 2, receptor 113	NM_001104578	Mm.469850
A_51_P333274	0.013	up	1.7993836	Gzmb	Mus musculus granzyme B (Gzmb), mRNA [NM_013542]	14939	NM_013542	granzyme B	NM_013542	Mm.14874
A_55_P2020128	0.000	up	1.7995454	Dhrs3	Mus musculus dehydrogenase/reductase (SDR family) member 3 (Dhrs3), transcript variant 1, mRNA [NM_011303]	20148	NM_011303	dehydrogenase/reductase (SDR family) member 3	NM_011303	Mm.14063
A_55_P2136776	0.048	up	1.8022532	Sult1b1	Mus musculus sulfotransferase family 1B, member 1 (Sult1b1), mRNA [NM_019878]	56362	NM_019878	sulfotransferase family 1B, member 1	NM_019878	Mm.23502
A_55_P2284497	0.016	up	1.803745	Smim18	Mus musculus small integral membrane protein 18 (Smim18), mRNA [NM_001206849]	72632	NM_001206849	small integral membrane protein 18	NM_001206849	Mm.443483
A_55_P2029319	0.013	up	1.8055224	Cd70	Mus musculus CD70 antigen (Cd70), mRNA [NM_011617]	21948	NM_011617	CD70 antigen	NM_011617	Mm.42228
A_55_P1979575	0.003	up	1.806537	Shroom2	Mus musculus shroom family member 2 (Shroom2), mRNA [NM_172441]	110380	NM_172441	shroom family member 2	NM_172441	Mm.40796
A_55_P2166212	0.040	up	1.8083241	Vmn2r111	Mus musculus vomeronasal 2, receptor 111 (Vmn2r111), mRNA [NM_001104573]	210876	NM_001104573	vomeronasal 2, receptor 111	NM_001104573	Mm.484955
A_55_P2015541	0.037	up	1.8089881	Hif3a	Mus musculus hypoxia inducible factor 3, alpha subunit (Hif3a), transcript variant 2, mRNA [NM_016868]	53417	NM_016868	hypoxia inducible factor 3, alpha subunit	NM_016868	Mm.135110
A_51_P356265	0.001	up	1.809259	Acrbp	Mus musculus proacrosin binding protein (Acrbp), transcript variant 1, mRNA [NM_016845]	54137	NM_016845	proacrosin binding protein	NM_016845	Mm.347910

A_55_P1961720	0.038	up	1.8099843	Kcnt1	Mus musculus potassium channel, subfamily T, member 1 (Kcnt1), transcript variant 1, mRNA [NM_175462]	227632	NM_175462	potassium channel, subfamily T, member 1	NM_175462	Mm.119462
A_52_P84901	0.022	up	1.8157022	Slc44a1	Mus musculus solute carrier family 44, member 1 (Slc44a1), transcript variant 1, mRNA [NM_133891]	100434	NM_133891	solute carrier family 44, member 1	NM_133891	Mm.482207
A_51_P220162	0.004	up	1.8161702	Notch3	Mus musculus notch 3 (Notch3), mRNA [NM_008716]	18131	NM_008716	notch 3	NM_008716	Mm.439741
A_51_P334979	0.001	up	1.817494	Apoc2	Mus musculus apolipoprotein C-II (Apoc2), mRNA [NM_001277944]	11813	NM_001277944	apolipoprotein C-II	NM_001277944	Mm.28394
A_55_P2172678	0.033	up	1.8180676	Tm4sf19	Mus musculus transmembrane 4 L six family member 19 (Tm4sf19), mRNA [NM_001160402]	277203	NM_001160402	transmembrane 4 L six family member 19	NM_001160402	Mm.484240
A_51_P322265	0.002	up	1.8210135	Grap	Mus musculus GRB2-related adaptor protein (Grap), mRNA [NM_027817]	71520	NM_027817	GRB2-related adaptor protein	NM_027817	Mm.202760
A_52_P312102	0.002	up	1.8214622	Sema3g	sema domain, immunoglobulin domain (Ig), short basic domain, secreted, (semaphorin) 3G [Source:MGI Symbol;Acc:MGI:3041242] [ENSMUST00000090180]	218877	AK129018	sema domain, immunoglobulin domain (Ig), short basic domain, secreted, (semaphorin) 3G		Mm.27812
A_52_P365660	0.028	up	1.8226581	Lrrc4c	Mus musculus leucine rich repeat containing 4C (Lrrc4c), mRNA [NM_178725]	241568	NM_178725	leucine rich repeat containing 4C	NM_178725	Mm.241682
A_51_P340747	0.018	up	1.8296181	Hspa1l	Mus musculus heat shock protein 1-like (Hspa1l), mRNA [NM_013558]	15482	NM_013558	heat shock protein 1-like	NM_013558	Mm.14287
A_51_P128075	0.030	up	1.8320402	Tescl	Mus musculus tescalcin-like (Tescl), mRNA [NM_001163810]	69301	NM_001163810	tescalcin-like	NM_001163810	Mm.144202
A_55_P2007210	0.044	up	1.8323786	Abca15	Mus musculus ATP-binding cassette, sub-family A (ABC1), member 15 (Abca15), mRNA [NM_177213]	320631	NM_177213	ATP-binding cassette, sub-family A (ABC1), member 15	NM_177213	Mm.78330
A_55_P2213438	0.014	up	1.8346751	Hnrnpr	Mus musculus heterogeneous nuclear ribonucleoprotein R (Hnrnpr), transcript variant 1, mRNA [NM_028871]	74326	NM_028871	heterogeneous nuclear ribonucleoprotein R	NM_028871	Mm.31051
A_55_P2094499	0.021	up	1.8352761	Tmem150b	Mus musculus transmembrane protein 150B (Tmem150b), transcript variant 2, mRNA [NM_177887]	330460	NM_177887	transmembrane protein 150B	NM_177887	Mm.361851
A_51_P415225	0.000	up	1.835348	Zfp105	Mus musculus zinc finger protein 105 (Zfp105), mRNA [NM_009544]	22646	NM_009544	zinc finger protein 105	NM_009544	Mm.290595
A_55_P2039532	0.005	up	1.8370528	Pax8	Mus musculus paired box gene 8 (Pax8), mRNA [NM_011040]	18510	NM_011040	paired box gene 8	NM_011040	Mm.2533
A_66_P116998	0.046	up	1.8375669	Tro	Mus musculus trophinin (Tro), transcript variant 1, mRNA [NM_001002272]	56191	NM_001002272	trophinin	NM_001002272	Mm.3597
A_55_P2321004	0.043	up	1.8429159	Zfp882	Mus musculus zinc finger protein 882 (Zfp882), mRNA [NM_001166645]	382019	NM_001166645	zinc finger protein 882	NM_001166645	Mm.251482
A_52_P467675	0.005	up	1.8462454	Ankrd63	Mus musculus ankyrin repeat domain 63 (Ankrd63), mRNA [NM_001081971]	383787	NM_001081971	ankyrin repeat domain 63	NM_001081971	Mm.35758
A_55_P2074843	0.001	up	1.8490628	Ccdc122	Mus musculus coiled-coil domain containing 122 (Ccdc122), mRNA [NM_175369]	108811	NM_175369	coiled-coil domain containing 122	NM_175369	Mm.102168
A_55_P2117187	0.007	up	1.8507985	Olfr1243	Mus musculus olfactory receptor 1243 (Olfr1243), mRNA [NM_146969]	258971	NM_146969	olfactory receptor 1243	NM_146969	Mm.389308
A_66_P114641	0.009	up	1.8521498	Cdh18	cadherin 18 [Source:MGI Symbol;Acc:MGI:1344366] [ENSMUST00000040427]	320865	AK137430	cadherin 18		Mm.241965
A_52_P469956	0.003	up	1.8526036	Zc3h6	Mus musculus zinc finger CCCH type containing 6 (Zc3h6), mRNA [NM_178404]	78751	NM_178404	zinc finger CCCH type containing 6	NM_178404	Mm.26377
A_55_P2144886	0.004	up	1.8530318	Robo3	Mus musculus roundabout homolog 3 (Drosophila) (Robo3), mRNA [NM_001164767]	19649	NM_001164767	roundabout homolog 3 (Drosophila)	NM_001164767	Mm.212826
A_55_P1962906	0.039	up	1.8537352	Als2cl	Mus musculus ALS2 C-terminal like (Als2cl), transcript variant 1, mRNA [NM_001146060]	235633	NM_001146060	ALS2 C-terminal like	NM_001146060	Mm.86338
A_55_P1992227	0.009	up	1.8538612	Zkscan2	Mus musculus zinc finger with KRAB and SCAN domains 2 (Zkscan2), mRNA [NM_001081329]	210162	NM_001081329	zinc finger with KRAB and SCAN domains 2	NM_001081329	Mm.135397
A_51_P169714	0.044	up	1.8550558	Shc2	Mus musculus SHC (Src homology 2 domain containing) transforming protein 2 (Shc2), mRNA [NM_001024539]	216148	NM_001024539	SHC (Src homology 2 domain containing) transforming protein 2	NM_001024539	Mm.39424
A_55_P2031979	0.021	up	1.8635205	Sspn	Mus musculus sarcospan (Sspn), mRNA [NM_010656]	16651	NM_010656	sarcospan	NM_010656	Mm.49689
A_52_P675617	0.006	up	1.8640912	Agbl3	ATP/GTP binding protein-like 3 [Source:MGI Symbol;Acc:MGI:1923473] [ENSMUST00000115014]	76223	BC087897	ATP/GTP binding protein-like 3		Mm.150047
A_55_P2117425	0.006	up	1.866801	Syt5	Mus musculus synaptotagmin V (Syt5), mRNA [NM_016908]	53420	NM_016908	synaptotagmin V	NM_016908	Mm.358663
A_52_P645632	0.014	up	1.8729057	Senp8	Mus musculus SUMO/sentrin specific peptidase 8 (Senp8), transcript variant 1, mRNA [NM_001172068]	71599	NM_001172068	SUMO/sentrin specific peptidase 8	NM_001172068	Mm.279070
A_55_P2030025	0.008	up	1.876024	Plag1	Mus musculus pleiomorphic adenoma gene 1 (Plag1), mRNA [NM_019969]	56711	NM_019969	pleiomorphic adenoma gene 1	NM_019969	Mm.331467
A_55_P2058550	0.043	up	1.8770075	Raet1c	Mus musculus retinoic acid early transcript gamma (Raet1c), mRNA [NM_009018]	19370	NM_009018	retinoic acid early transcript gamma	NM_009018	Mm.439724
A_55_P2040678	0.046	up	1.877459	Susd2	Mus musculus sushi domain containing 2 (Susd2), transcript variant 1, mRNA [NM_027890]	71733	NM_027890	sushi domain containing 2	NM_027890	Mm.247956
A_55_P1984690	0.012	up	1.8782495	Ptprr	Mus musculus protein tyrosine phosphatase, receptor type, R (Ptprr), transcript variant 1, mRNA [NM_011217]	19279	NM_011217	protein tyrosine phosphatase, receptor type, R	NM_011217	Mm.336316
A_51_P317740	0.019	up	1.8791159	Pcgf2	Mus musculus polycomb group ring finger 2 (Pcgf2), transcript variant 1, mRNA [NM_009545]	22658	NM_009545	polycomb group ring finger 2	NM_009545	Mm.2418
A_51_P329818	0.021	up	1.8793976	Dpep3	Mus musculus dipeptidase 3 (Dpep3), mRNA [NM_027960]	71854	NM_027960	dipeptidase 3	NM_027960	Mm.173395
A_51_P420547	0.000	up	1.8813074	Clic5	Mus musculus chloride intracellular channel 5 (Clic5), mRNA [NM_172621]	224796	NM_172621	chloride intracellular channel 5	NM_172621	Mm.37666

A_55_P1959283	0.034	up	1.881801	Rbm44	Mus musculus RNA binding motif protein 44 (Rbm44), mRNA [NM_001033408]	329207	NM_001033408	RNA binding motif protein 44	NM_001033408	Mm.295940
A_66_P121312	0.039	up	1.8854207	Olfr1251	Mus musculus olfactory receptor 1251 (Olfr1251), mRNA [NM_001011529]	259145	NM_001011529	olfactory receptor 1251	NM_001011529	Mm.376637
A_55_P2106459	0.005	up	1.8856729	Zfhx3	Mus musculus zinc finger homeobox 3 (Zfhx3), mRNA [NM_007496]	11906	NM_007496	zinc finger homeobox 3	NM_007496	Mm.416972
A_51_P342031	0.007	up	1.889735	Mkrn3	Mus musculus makorin, ring finger protein, 3 (Mkrn3), mRNA [NM_011746]	22652	NM_011746	makorin, ring finger protein, 3	NM_011746	Mm.146
A_55_P1986233	0.034	up	1.8909578	Tubgcp4	Mus musculus 12 days embryo spinal ganglion cDNA, RIKEN full-length enriched library, clone:D130054F11 product:SIMILAR TO GAMMA TUBULIN RING COMPLEX PROTEIN (76P GENE) homolog [Homo sapiens], full insert sequence. [AK051517]	51885	AK051517	tubulin, gamma complex associated protein 4		Mm.383499
A_51_P216496	0.037	up	1.8931653	Tfpi2	Mus musculus tissue factor pathway inhibitor 2 (Tfpi2), mRNA [NM_009364]	21789	NM_009364	tissue factor pathway inhibitor 2	NM_009364	Mm.25612
A_55_P1995337	0.043	up	1.8979139	Btnl5-ps	Mus musculus butyrophilin-like 5, pseudogene (Btnl5-ps), non-coding RNA [NR_004051]	81497	NR_004051	butyrophilin-like 5, pseudogene	NR_004051	Mm.484231
A_55_P1964193	0.013	up	1.9046198	Map1b	Mus musculus microtubule-associated protein 1B (Map1b), mRNA [NM_008634]	17755	NM_008634	microtubule-associated protein 1B	NM_008634	Mm.4173
A_55_P2060532	0.004	up	1.9051384	Mtl5	Mus musculus metallothionein-like 5, testis-specific (tesmin) (Mtl5), transcript variant 2, mRNA [NM_001039658]	17771	NM_001039658	metallothionein-like 5, testis-specific (tesmin)	NM_001039658	Mm.209941
A_55_P2363030	0.039	up	1.9065063	Cmtm4	Mus musculus CKLF-like MARVEL transmembrane domain containing 4 (Cmtm4), mRNA [NM_153582]	97487	NM_153582	CKLF-like MARVEL transmembrane domain containing 4	NM_153582	Mm.29658
A_51_P324651	0.014	up	1.9095253	Lphn1	Mus musculus latrophilin 1 (Lphn1), mRNA [NM_181039]	330814	NM_181039	latrophilin 1	NM_181039	Mm.260733
A_52_P628212	0.007	up	1.9103866	Esrrb	Mus musculus adult retina cDNA, RIKEN full-length enriched library, clone:A930008D01 product:estrogen related receptor, beta, full insert sequence. [AK044339]	26380	AK044339	estrogen related receptor, beta		
A_52_P35048	0.000	up	1.9106913	Serpinf1	Mus musculus serine (or cysteine) peptidase inhibitor, clade F, member 1 (Serpinf1), mRNA [NM_011340]	20317	NM_011340	serine (or cysteine) peptidase inhibitor, clade F, member 1	NM_011340	Mm.2044
A_51_P317941	0.013	up	1.9109895	Perp	Mus musculus PERP, TP53 apoptosis effector (Perp), mRNA [NM_022032]	64058	NM_022032	PERP, TP53 apoptosis effector	NM_022032	Mm.28209
A_51_P419759	0.007	up	1.9124916	Tha1	Mus musculus threonine aldolase 1 (Tha1), mRNA [NM_027919]	71776	NM_027919	threonine aldolase 1	NM_027919	Mm.3200
A_55_P2032538	0.001	up	1.912745	Pde3b	Mus musculus phosphodiesterase 3B, cGMP-inhibited (Pde3b), mRNA [NM_011055]	18576	NM_011055	phosphodiesterase 3B, cGMP-inhibited	NM_011055	Mm.430730
A_51_P181891	0.001	up	1.9162463	Amer2	Mus musculus APC membrane recruitment 2 (Amer2), transcript variant 1, mRNA [NM_028113]	72125	NM_028113	APC membrane recruitment 2	NM_028113	Mm.275499
A_55_P2187141	0.006	up	1.9171697	Pdcd1lg2	Mus musculus programmed cell death 1 ligand 2 (Pdcd1lg2), mRNA [NM_021396]	58205	NM_021396	programmed cell death 1 ligand 2	NM_021396	Mm.116737
A_55_P2208041	0.022	up	1.9175113	Stra8	Mus musculus stimulated by retinoic acid gene 8 (Stra8), mRNA [NM_009292]	20899	NM_009292	stimulated by retinoic acid gene 8	NM_009292	Mm.5171
A_52_P58359	0.035	up	1.9189745	Tlx1	Mus musculus T cell leukemia, homeobox 1 (Tlx1), mRNA [NM_021901]	21908	NM_021901	T cell leukemia, homeobox 1	NM_021901	Mm.391203
A_51_P450549	0.008	up	1.9214536	Padi3	Mus musculus peptidyl arginine deiminase, type III (Padi3), mRNA [NM_011060]	18601	NM_011060	peptidyl arginine deiminase, type III	NM_011060	Mm.20851
A_51_P458008	0.041	up	1.9244955	Wscd1	WSC domain containing 1 [Source:MGI Symbol;Acc:MGI:2448493] [ENSMUST00000138339]	216881	AK049137	WSC domain containing 1		Mm.48648
A_51_P511899	0.001	up	1.9249582	Mthfsd	Mus musculus methenyltetrahydrofolate synthetase domain containing (Mthfsd), transcript variant 2, mRNA [NM_172761]	234814	NM_172761	methenyltetrahydrofolate synthetase domain containing	NM_172761	Mm.31690
A_51_P248335	0.028	up	1.9257879	Zfp618	Mus musculus 10, 11 days embryo whole body cDNA, RIKEN full-length enriched library, clone:2810031P15 product:hypothetical Zinc finger, C2H2 type containing protein, full insert sequence. [AK012848]	72701	AK012848	zinc finger protein 618		Mm.482416
A_66_P128442	0.000	up	1.9275675	Rimbp3	Mus musculus RIMS binding protein 3 (Rimbp3), mRNA [NM_001033338]	239731	NM_001033338	RIMS binding protein 3	NM_001033338	Mm.86671
A_55_P2016237	0.037	up	1.9343227	Hand2	Mus musculus heart and neural crest derivatives expressed transcript 2 (Hand2), mRNA [NM_010402]	15111	NM_010402	heart and neural crest derivatives expressed transcript 2	NM_010402	Mm.430844
A_55_P1955627	0.029	up	1.9377273	Chrdl1	Mus musculus chordin-like 1 (Chrdl1), transcript variant 1, mRNA [NM_001114385]	83453	NM_001114385	chordin-like 1	NM_001114385	Mm.157697
A_55_P2054315	0.012	up	1.9386445	Mcpt2	Mus musculus mast cell protease 2 (Mcpt2), mRNA [NM_008571]	17225	NM_008571	mast cell protease 2	NM_008571	Mm.4409
A_55_P1952449	0.028	up	1.9396259	Dyx1c1	Mus musculus dyslexia susceptibility 1 candidate 1 homolog (human) (Dyx1c1), transcript variant 1, mRNA [NM_026314]	67685	NM_026314	dyslexia susceptibility 1 candidate 1 homolog (human)	NM_026314	Mm.31817
A_55_P2014427	0.002	up	1.9403299	Il17re	Mus musculus interleukin 17 receptor E (Il17re), transcript variant 3, mRNA [NM_001034031]	57890	NM_001034031	interleukin 17 receptor E	NM_001034031	Mm.131781
A_51_P309733	0.029	up	1.9420327	Cyp11a1	Mus musculus cytochrome P450, family 11, subfamily a, polypeptide 1 (Cyp11a1), mRNA [NM_019779]	13070	NM_019779	cytochrome P450, family 11, subfamily a, polypeptide 1	NM_019779	Mm.302865
A_55_P2087008	0.038	up	1.948847	Dsg3	Mus musculus 10 days neonate skin cDNA, RIKEN full-length enriched library, clone:4732482D04 product:desmoglein 3, full insert sequence. [AK029018]	13512	AK029018	desmoglein 3		Mm.106811
A_51_P472829	0.004	up	1.9506315	Aif1l	Mus musculus allograft inflammatory factor 1-like (Aif1l), mRNA [NM_145144]	108897	NM_145144	allograft inflammatory factor 1-like	NM_145144	Mm.24838
A_52_P213889	0.035	up	1.952057	Tmc7	Mus musculus transmembrane channel-like gene family 7 (Tmc7), mRNA [NM_172476]	209760	NM_172476	transmembrane channel-like gene family 7	NM_172476	Mm.69380
A_55_P2125982	0.007	up	1.9524266	Lrrc4b	Mus musculus leucine rich repeat containing 4B (Lrrc4b), mRNA [NM_198250]	272381	NM_198250	leucine rich repeat containing 4B	NM_198250	Mm.44173
A_52_P468068	0.011	up	1.9530383	Tchh	Mus musculus trichohyalin (Tchh), mRNA [NM_001163098]	99681	NM_001163098	trichohyalin	NM_001163098	Mm.316671

A_55_P2064446	0.000	up	1.9539217	Pced1a	Mus musculus PC-esterase domain containing 1A (Pced1a), transcript variant 1, mRNA [NM_178762]	319513	NM_178762	PC-esterase domain containing 1A	NM_178762	Mm.482539
A_55_P2137941	0.006	up	1.9547102	Fxyd2	Mus musculus FXYD domain-containing ion transport regulator 2 (Fxyd2), transcript variant b, mRNA [NM_052823]	11936	NM_052823	FXYD domain-containing ion transport regulator 2	NM_052823	Mm.22742
A_55_P1954086	0.007	up	1.9558233	Postn	Mus musculus periostin, osteoblast specific factor (Postn), transcript variant 3, mRNA [NM_001198766]	50706	NM_001198766	periostin, osteoblast specific factor	NM_001198766	Mm.236067
A_55_P2120581	0.046	up	1.9698569	Mei4	Mus musculus meiosis-specific, MEI4 homolog (S. cerevisiae) (Mei4), mRNA [NM_175213]	75033	NM_175213	meiosis-specific, MEI4 homolog (S. cerevisiae)	NM_175213	Mm.335329
A_51_P352303	0.011	up	1.9713678	Homer2	Mus musculus homer homolog 2 (Drosophila) (Homer2), transcript variant 1, mRNA [NM_011983]	26557	NM_011983	homer homolog 2 (Drosophila)	NM_011983	Mm.228
A_55_P2053181	0.024	up	1.9726171	Nefh	Mus musculus neurofilament, heavy polypeptide (Nefh), mRNA [NM_010904]	380684	NM_010904	neurofilament, heavy polypeptide	NM_010904	Mm.298283
A_55_P2026982	0.003	up	1.9746056	Tdrp	Mus musculus testis development related protein (Tdrp), mRNA [NM_173744]	72148	NM_173744	testis development related protein	NM_173744	Mm.5727
A_66_P101132	0.000	up	1.9756824	Hist1h2bp	Mus musculus histone cluster 1, H2bp (Hist1h2bp), mRNA [NM_178202]	319188	NM_178202	histone cluster 1, H2bp	NM_178202	Mm.264645
A_55_P2014882	0.001	up	1.9793595	Dhrs9	Mus musculus dehydrogenase/reductase (SDR family) member 9 (Dhrs9), mRNA [NM_175512]	241452	NM_175512	dehydrogenase/reductase (SDR family) member 9	NM_175512	Mm.211655
A_51_P213359	0.018	up	1.9808099	Has2	Mus musculus hyaluronan synthase 2 (Has2), mRNA [NM_008216]	15117	NM_008216	hyaluronan synthase 2	NM_008216	Mm.5148
A_55_P1967727	0.005	up	1.9824344	Ceacam19	Mus musculus carcinoembryonic antigen-related cell adhesion molecule 19 (Ceacam19), mRNA [NM_177036]	319930	NM_177036	carcinoembryonic antigen-related cell adhesion molecule 19	NM_177036	Mm.215839
A_55_P2134645	0.012	up	1.9838777	Fam227a	family with sequence similarity 227, member A [Source:MGI Symbol;Acc:MGI:1922979] [ENSMUST00000046816]	75729	AK039547	family with sequence similarity 227, member A		Mm.78946
A_52_P342131	0.036	up	1.9900807	Zfp532	Mus musculus zinc finger protein 532 (Zfp532), mRNA [NM_207255]	328977	NM_207255	zinc finger protein 532	NM_207255	Mm.286232
A_55_P2018191	0.005	up	1.9929366	Ccm2l	Mus musculus cerebral cavernous malformation 2-like (Ccm2l), mRNA [NM_145536]	228788	NM_145536	cerebral cavernous malformation 2-like	NM_145536	Mm.299979
A_55_P2318584	0.008	up	1.9937034	Aqp8	Mus musculus aquaporin 8 (Aqp8), transcript variant 1, mRNA [NM_007474]	11833	NM_007474	aquaporin 8	NM_007474	Mm.273175
A_51_P159453	0.041	up	1.9950365	Serpina3n	Mus musculus serine (or cysteine) peptidase inhibitor, clade A, member 3N (Serpina3n), mRNA [NM_009252]	20716	NM_009252	serine (or cysteine) peptidase inhibitor, clade A, member 3N	NM_009252	Mm.482074
A_51_P469252	0.004	up	1.9978261	Zfp647	Mus musculus zinc finger protein 647 (Zfp647), transcript variant 2, mRNA [NM_172817]	239546	NM_172817	zinc finger protein 647	NM_172817	Mm.183650
A_55_P2082653	0.018	up	1.999795	Zfp711	Mus musculus zinc finger protein 711 (Zfp711), mRNA [NM_177747]	245595	NM_177747	zinc finger protein 711	NM_177747	Mm.259846
A_55_P2123673	0.000	up	18.445747	Trnp1	Mus musculus TMF1-regulated nuclear protein 1 (Trnp1), mRNA [NM_001081156]	69539	NM_001081156	TMF1-regulated nuclear protein 1	NM_001081156	Mm.151594
A_55_P2119009	0.000	up	2.0000684	Foxe3	Mus musculus forkhead box E3 (Foxe3), mRNA [NM_015758]	30923	NM_015758	forkhead box E3	NM_015758	Mm.377122
A_66_P124659	0.026	up	2.0001068	Mark1	Mus musculus MAP/microtubule affinity-regulating kinase 1 (Mark1), mRNA [NM_145515]	226778	NM_145515	MAP/microtubule affinity-regulating kinase 1	NM_145515	Mm.7445
A_55_P1987414	0.004	up	2.0038753	Nbeal1	Mus musculus neurobeachin like 1 (Nbeal1), mRNA [NM_173444]	269198	NM_173444	neurobeachin like 1	NM_173444	Mm.404295
A_52_P357335	0.016	up	2.0116699	Tmco1	Mus musculus transmembrane and coiled-coil domains 1 (Tmco1), mRNA [NM_001039483]	68944	NM_001039483	transmembrane and coiled-coil domains 1	NM_001039483	Mm.330045
A_55_P2162935	0.046	up	2.014917	Ntn1	Mus musculus netrin 1 (Ntn1), mRNA [NM_008744]	18208	NM_008744	netrin 1	NM_008744	Mm.39095
A_52_P309890	0.026	up	2.0174844	Mcc	Mus musculus mutated in colorectal cancers (Mcc), transcript variant 1, mRNA [NM_001085373]	328949	NM_001085373	mutated in colorectal cancers	NM_001085373	Mm.312511
A_55_P1983643	0.017	up	2.0177875	Vmn1r45	Mus musculus vomeronasal 1 receptor 45 (Vmn1r45), mRNA [NM_011684]	22297	NM_011684	vomeronasal 1 receptor 45	NM_011684	Mm.431981
A_55_P2096435	0.005	up	2.024964	Zc3h6	Mus musculus zinc finger CCCH type containing 6 (Zc3h6), mRNA [NM_178404]	78751	NM_178404	zinc finger CCCH type containing 6	NM_178404	Mm.26377
A_55_P1967761	0.032	up	2.0269754	Foxc1	Mus musculus forkhead box C1 (Foxc1), mRNA [NM_008592]	17300	NM_008592	forkhead box C1	NM_008592	Mm.12949
A_55_P2006345	0.008	up	2.0270424	Ccdc85b	Mus musculus coiled-coil domain containing 85B (Ccdc85b), transcript variant 1, mRNA [NM_198616]	240514	NM_198616	coiled-coil domain containing 85B	NM_198616	Mm.329657
A_55_P2156712	0.013	up	2.0287015	Gpr1	Mus musculus G protein-coupled receptor 1 (Gpr1), mRNA [NM_146250]	241070	NM_146250	G protein-coupled receptor 1	NM_146250	Mm.103354
A_55_P2042612	0.000	up	2.0381217	C1s	Mus musculus complement component 1, s subcomponent (C1s), transcript variant 1, mRNA [NM_144938]	50908	NM_144938	complement component 1, s subcomponent	NM_144938	Mm.219527
A_52_P239320	0.021	up	2.0386348	Syngr1	Mus musculus synaptogyrin 1 (Syngr1), transcript variant 1a, mRNA [NM_207708]	20972	NM_207708	synaptogyrin 1	NM_207708	Mm.230301
A_51_P161485	0.040	up	2.0451949	Wbp2nl	Mus musculus WBP2 N-terminal like (Wbp2nl), mRNA [NM_029066]	74716	NM_029066	WBP2 N-terminal like	NM_029066	Mm.159315
A_51_P258690	0.001	up	2.0453398	Scrg1	Mus musculus scrapie responsive gene 1 (Scrg1), mRNA [NM_009136]	20284	NM_009136	scrapie responsive gene 1	NM_009136	Mm.12886
A_52_P537852	0.043	up	2.0470283	Fam120b	Mus musculus family with sequence similarity 120, member B (Fam120b), transcript variant 1, mRNA [NM_024203]	67544	NM_024203	family with sequence similarity 120, member B	NM_024203	Mm.35039
A_51_P458451	0.002	up	2.0526762	Adipoq	Mus musculus adiponectin, C1Q and collagen domain containing (Adipoq), mRNA [NM_009605]	11450	NM_009605	adiponectin, C1Q and collagen domain containing	NM_009605	Mm.3969
A_51_P324838	0.010	up	2.0533931	Evpl	Mus musculus envoplakin (Evpl), mRNA [NM_025276]	14027	NM_025276	envoplakin	NM_025276	Mm.293683
A_55_P1969015	0.010	up	2.058425	Asb18	Mus musculus ankyrin repeat and SOCS box-containing 18 (Asb18), mRNA [NM_139152]	208372	NM_139152	ankyrin repeat and SOCS box-containing 18	NM_139152	Mm.215164
A_55_P2137314	0.023	up	2.0622404	Kctd21	Mus musculus potassium channel tetramerisation domain containing 21 (Kctd21), mRNA [NM_001039039]	622320	NM_001039039	potassium channel tetramerisation domain containing 21	NM_001039039	Mm.334029

A_55_P2170454	0.021	up	2.0723915	Gsta2	Mus musculus glutathione S-transferase, alpha 2 (Yc2) (Gsta2), mRNA [NM_008182]	14858	NM_008182	glutathione S-transferase, alpha 2 (Yc2)	NM_008182	Mm.422778
A_55_P2169586	0.000	up	2.0730395	Ccdc80	Mus musculus coiled-coil domain containing 80 (Ccdc80), mRNA [NM_026439]	67896	NM_026439	coiled-coil domain containing 80	NM_026439	Mm.181074
A_51_P142896	0.041	up	2.0780373	Cd59a	Mus musculus CD59a antigen (Cd59a), transcript variant 2, mRNA [NM_007652]	12509	NM_007652	CD59a antigen	NM_007652	Mm.247265
A_55_P2023523	0.004	up	2.078601	Aloxe3	Mus musculus arachidonate lipoxygenase 3 (Aloxe3), mRNA [NM_011786]	23801	NM_011786	arachidonate lipoxygenase 3	NM_011786	Mm.41989
A_55_P2028698	0.009	up	2.083565	Dmc1	Mus musculus DMC1 dosage suppressor of mck1 homolog, meiosis-specific homologous recombination (Dmc1), transcript variant 1, mRNA [NM_010059]	13404	NM_010059	DMC1 dosage suppressor of mck1 homolog, meiosis-specific homologous recombination	NM_010059	Mm.2524
A_55_P1969356	0.004	up	2.08482	Ccm2l	Mus musculus cerebral cavernous malformation 2-like (Ccm2l), mRNA [NM_145536]	228788	NM_145536	cerebral cavernous malformation 2-like	NM_145536	Mm.299979
A_55_P1994128	0.022	up	2.0868535	Tmem184a	Mus musculus transmembrane protein 184a (Tmem184a), transcript variant 1, mRNA [NM_001161548]	231832	NM_001161548	transmembrane protein 184a	NM_001161548	Mm.482655
A_55_P1981836	0.002	up	2.0911195	Ccm2l	Mus musculus cerebral cavernous malformation 2-like (Ccm2l), mRNA [NM_145536]	228788	NM_145536	cerebral cavernous malformation 2-like	NM_145536	Mm.299979
A_55_P2164977	0.002	up	2.091777	Tcf24	Mus musculus transcription factor 24 (Tcf24), mRNA [NM_001285425]	1E+08	NM_001285425	transcription factor 24	NM_001285425	
A_51_P176352	0.000	up	2.1125038	Ndrg2	Mus musculus N-myc downstream regulated gene 2 (Ndrg2), transcript variant 1, mRNA [NM_013864]	29811	NM_013864	N-myc downstream regulated gene 2	NM_013864	Mm.26722
A_51_P305628	0.000	up	2.1192434	Atp8a2	Mus musculus ATPase, aminophospholipid transporter-like, class I, type 8A, member 2 (Atp8a2), mRNA [NM_015803]	50769	NM_015803	ATPase, aminophospholipid transporter-like, class I, type 8A, member 2	NM_015803	Mm.319599
A_52_P177271	0.001	up	2.1278737	Mtl5	Mus musculus metallothionein-like 5, testis-specific (tesmin) (Mtl5), transcript variant 1, mRNA [NM_001039657]	17771	NM_001039657	metallothionein-like 5, testis-specific (tesmin)	NM_001039657	Mm.209941
A_52_P659470	0.002	up	2.128472	Rgs9bp	Mus musculus regulator of G-protein signalling 9 binding protein (Rgs9bp), mRNA [NM_145840]	243923	NM_145840	regulator of G-protein signalling 9 binding protein	NM_145840	Mm.222680
A_52_P281702	0.001	up	2.1443672	Igfbp5	Mus musculus insulin-like growth factor binding protein 5 (Igfbp5), mRNA [NM_010518]	16011	NM_010518	insulin-like growth factor binding protein 5	NM_010518	Mm.405761
A_55_P2024720	0.037	up	2.146502	Olfr390	Mus musculus olfactory receptor 390 (Olfr390), mRNA [NM_146347]	258344	NM_146347	olfactory receptor 390	NM_146347	Mm.390356
A_51_P418116	0.001	up	2.1505885	Tmem119	Mus musculus transmembrane protein 119 (Tmem119), mRNA [NM_146162]	231633	NM_146162	transmembrane protein 119	NM_146162	Mm.41681
A_55_P2172096	0.040	up	2.1526587	Mc1r	Mus musculus melanocortin 1 receptor (Mc1r), mRNA [NM_008559]	17199	NM_008559	melanocortin 1 receptor	NM_008559	Mm.324942
A_55_P1979814	0.005	up	2.1538892	Abcc8	Mus musculus ATP-binding cassette, sub-family C (CFTR/MRP), member 8 (Abcc8), mRNA [NM_011510]	20927	NM_011510	ATP-binding cassette, sub-family C (CFTR/MRP), member 8	NM_011510	Mm.259076
A_52_P93066	0.011	up	2.1563122	Klhl42	Mus musculus kelch-like 42 (Klhl42), mRNA [NM_001081237]	232539	NM_001081237	kelch-like 42	NM_001081237	Mm.491206
A_55_P2027386	0.044	up	2.1572435	Gpr156	Mus musculus G protein-coupled receptor 156 (Gpr156), mRNA [NM_153394]	239845	NM_153394	G protein-coupled receptor 156	NM_153394	Mm.228018
A_55_P1973501	0.000	up	2.1720433	Ceacam16	Mus musculus carcinoembryonic antigen-related cell adhesion molecule 16 (Ceacam16), mRNA [NM_001033419]	330483	NM_001033419	carcinoembryonic antigen-related cell adhesion molecule 16	NM_001033419	Mm.260192
A_51_P234692	0.000	up	2.1724956	Neat1	Mus musculus nuclear paraspeckle assembly transcript 1 (non-protein coding) (Neat1), long non-coding RNA [NR_003513]	66961	NR_003513	nuclear paraspeckle assembly transcript 1 (non-protein coding)	NR_003513	Mm.281895
A_55_P1991841	0.017	up	2.1755493	Slc22a27	Mus musculus solute carrier family 22, member 27 (Slc22a27), mRNA [NM_134256]	171405	NM_134256	solute carrier family 22, member 27	NM_134256	Mm.326517
A_55_P2072453	0.016	up	2.175969	Marc1	Mus musculus mitochondrial amidoxime reducing component 1 (Marc1), mRNA [NM_001081361]	66112	NM_001081361	mitochondrial amidoxime reducing component 1	NM_001081361	Mm.272457
A_55_P2095563	0.021	up	2.190837	Slc8a2	Mus musculus solute carrier family 8 (sodium/calcium exchanger), member 2 (Slc8a2), mRNA [NM_148946]	110891	NM_148946	solute carrier family 8 (sodium/calcium exchanger), member 2	NM_148946	Mm.241147
A_55_P2095103	0.041	up	2.1946611	Gucy1a2	Mus musculus guanylate cyclase 1, soluble, alpha 2 (Gucy1a2), mRNA [NM_001033322]	234889	NM_001033322	guanylate cyclase 1, soluble, alpha 2	NM_001033322	Mm.91240
A_51_P336599	0.004	up	2.1955385	Kcne3	Mus musculus potassium voltage-gated channel, Isk-related subfamily, gene 3 (Kcne3), transcript variant 2, mRNA [NM_020574]	57442	NM_020574	potassium voltage-gated channel, Isk-related subfamily, gene 3	NM_020574	Mm.282386
A_55_P1972039	0.011	up	2.2128415	Nox4	Mus musculus NADPH oxidase 4 (Nox4), transcript variant 2, mRNA [NM_001285833]	50490	NM_001285833	NADPH oxidase 4	NM_001285833	Mm.31748
A_52_P136782	0.003	up	2.2180758	Rgs5	Mus musculus regulator of G-protein signaling 5 (Rgs5), mRNA [NM_009063]	19737	NM_009063	regulator of G-protein signaling 5	NM_009063	Mm.20954
A_51_P237585	0.000	up	2.2360969	Btnl9	Mus musculus butyrophilin-like 9 (Btnl9), mRNA [NM_172793]	237754	NM_172793	butyrophilin-like 9	NM_172793	Mm.130787
A_52_P613643	0.000	up	2.238098	Zfp3	Mus musculus zinc finger protein 3 (Zfp3), mRNA [NM_177565]	193043	NM_177565	zinc finger protein 3	NM_177565	Mm.40966
A_55_P1975370	0.000	up	2.243443	Apoc1	Mus musculus apolipoprotein C-I (Apoc1), transcript variant 1, mRNA [NM_007469]	11812	NM_007469	apolipoprotein C-I	NM_007469	Mm.182440

A_55_P2046877	0.007	up	2.2456934	Foxq1	Mus musculus forkhead box Q1 (Foxq1), mRNA [NM_008239]	15220	NM_008239	forkhead box Q1	NM_008239	Mm.44235
A_55_P2039359	0.003	up	2.247934	Tnfsf11	Mus musculus tumor necrosis factor (ligand) superfamily, member 11 (Tnfsf11), mRNA [NM_011613]	21943	NM_011613	tumor necrosis factor (ligand) superfamily, member 11	NM_011613	Mm.249221
A_55_P1974487	0.002	up	2.2501159	Atp8b5	Mus musculus ATPase, class I, type 8B, member 5 (Atp8b5), mRNA [NM_177195]	320571	NM_177195	ATPase, class I, type 8B, member 5	NM_177195	Mm.74465
A_55_P2183208	0.000	up	2.2527063	Prl2c1	Mus musculus Prolactin family 2, subfamily c, member 1 (Prl2c1), mRNA [NM_001045532]	666317	NM_001045532	Prolactin family 2, subfamily c, member 1	NM_001045532	Mm.432060
A_52_P399095	0.027	up	2.2543855	Mettl24	Mus musculus methyltransferase like 24 (Mettl24), mRNA [NM_177793]	327747	NM_177793	methyltransferase like 24	NM_177793	Mm.80407
A_55_P2025514	0.036	up	2.2550771	Pnpla3	Mus musculus patatin-like phospholipase domain containing 3 (Pnpla3), mRNA [NM_054088]	116939	NM_054088	patatin-like phospholipase domain containing 3	NM_054088	Mm.390817
A_55_P2167530	0.036	up	2.2555797	Scube3	signal peptide, CUB domain, EGF-like 3 [Source:MGI Symbol;Acc:MGI:3045253] [ENSMUST00000043503]	268935		signal peptide, CUB domain, EGF-like 3		
A_55_P2174982	0.002	up	2.2560532	Cngb1	Mus musculus cyclic nucleotide gated channel beta 1 (Cngb1), transcript variant 1, mRNA [NM_001195413]	333329	NM_001195413	cyclic nucleotide gated channel beta 1	NM_001195413	Mm.484049
A_55_P2078335	0.001	up	2.2579236	Mapk4	Mus musculus mitogen-activated protein kinase 4 (Mapk4), mRNA [NM_172632]	225724	NM_172632	mitogen-activated protein kinase 4	NM_172632	Mm.254517
A_55_P2056846	0.021	up	2.2621684	Olfr525	Mus musculus olfactory receptor 525 (Olfr525), mRNA [NM_146956]	258958	NM_146956	olfactory receptor 525	NM_146956	Mm.347648
A_51_P325501	0.019	up	2.281968	Crip3	Mus musculus cysteine-rich protein 3 (Crip3), transcript variant TLP-B, mRNA [NM_053250]	114570	NM_053250	cysteine-rich protein 3	NM_053250	Mm.25168
A_55_P2065074	0.050	up	2.2863803	Hdac9	Mus musculus histone deacetylase 9 (Hdac9), transcript variant 1, mRNA [NM_001271386]	79221	NM_001271386	histone deacetylase 9	NM_001271386	Mm.310551
A_51_P503822	0.028	up	2.3015265	Slitrk6	Mus musculus SLIT and NTRK-like family, member 6 (Slitrk6), mRNA [NM_175499]	239250	NM_175499	SLIT and NTRK-like family, member 6	NM_175499	Mm.49728
A_51_P143031	0.007	up	2.3096523	Eps8l3	Mus musculus EPS8-like 3 (Eps8l3), mRNA [NM_133867]	99662	NM_133867	EPS8-like 3	NM_133867	Mm.108491
A_55_P2036452	0.032	up	2.3278494	Ckap4	Mus musculus 13 days embryo head cDNA, RIKEN full-length enriched library, clone:3110096E07 product:cytoskeleton-associated protein 4, full insert sequence. [AK132136]	216197	AK132136	cytoskeleton-associated protein 4		Mm.334999
A_55_P2108708	0.004	up	2.3468862	Kcne3	Mus musculus potassium voltage-gated channel, Isk-related subfamily, gene 3 (Kcne3), transcript variant 4, mRNA [NM_001190870]	57442	NM_001190870	potassium voltage-gated channel, Isk-related subfamily, gene 3	NM_001190870	Mm.282386
A_51_P224042	0.012	up	2.3540678	Ppil6	Mus musculus peptidylprolyl isomerase (cyclophilin)-like 6 (Ppil6), mRNA [NM_028430]	73075	NM_028430	peptidylprolyl isomerase (cyclophilin)-like 6	NM_028430	Mm.359762
A_55_P2067632	0.007	up	2.359352	Ush1c	Mus musculus Usher syndrome 1C (Ush1c), transcript variant b4, mRNA [NM_001163733]	72088	NM_001163733	Usher syndrome 1C	NM_001163733	Mm.119709
A_55_P2011445	0.001	up	2.363846	Capn13	Mus musculus calpain 13 (Capn13), mRNA [NM_001033444]	381122	NM_001033444	calpain 13	NM_001033444	Mm.81496
A_55_P2207055	0.000	up	2.3650346	Btnl9	butyrophilin-like 9 [Source:MGI Symbol;Acc:MGI:2442439] [ENSMUST00000046522]	237754	AA510905	butyrophilin-like 9		Mm.268294
A_55_P2128869	0.006	up	2.3683	Ccdc80	Mus musculus coiled-coil domain containing 80 (Ccdc80), mRNA [NM_026439]	67896	NM_026439	coiled-coil domain containing 80	NM_026439	Mm.181074
A_55_P1956837	0.000	up	2.370863	Rnf157	ring finger protein 157 [Source:MGI Symbol;Acc:MGI:2442484] [ENSMUST00000149682]	217340	AK081001	ring finger protein 157		Mm.483447
A_52_P708507	0.003	up	2.3712249	Myadml2	Mus musculus myeloid-associated differentiation marker-like 2 (Myadml2), transcript variant 1, mRNA [NM_026751]	68515	NM_026751	myeloid-associated differentiation marker-like 2	NM_026751	Mm.194594
A_55_P2021572	0.011	up	2.3716471	C87414	Mus musculus expressed sequence C87414 (C87414), transcript variant 1, mRNA [NM_001164284]	381654	NM_001164284	expressed sequence C87414	NM_001164284	Mm.326591
A_51_P499698	0.000	up	2.3746903	Asprv1	Mus musculus aspartic peptidase, retroviral-like 1 (Asprv1), mRNA [NM_026414]	67855	NM_026414	aspartic peptidase, retroviral-like 1	NM_026414	Mm.183043
A_51_P508838	0.009	up	2.375248	Kcne4	Mus musculus potassium voltage-gated channel, Isk-related subfamily, gene 4 (Kcne4), mRNA [NM_021342]	57814	NM_021342	potassium voltage-gated channel, Isk-related subfamily, gene 4	NM_021342	Mm.24386
A_51_P474701	0.000	up	2.3828967	Fbp1	Mus musculus fructose bisphosphatase 1 (Fbp1), mRNA [NM_019395]	14121	NM_019395	fructose bisphosphatase 1	NM_019395	Mm.423078
A_51_P237865	0.026	up	2.3839142	Il4	Mus musculus interleukin 4 (Il4), transcript variant 1, mRNA [NM_021283]	16189	NM_021283	interleukin 4	NM_021283	Mm.276360
A_55_P2105944	0.010	up	2.388309	Olfr224	Mus musculus olfactory receptor 224 (Olfr224), mRNA [NM_207695]	258198	NM_207695	olfactory receptor 224	NM_207695	Mm.480307
A_52_P527625	0.004	up	2.3963645	Colec12	Mus musculus collectin sub-family member 12 (Colec12), mRNA [NM_130449]	140792	NM_130449	collectin sub-family member 12	NM_130449	Mm.218571
A_55_P2113758	0.040	up	2.411344	Gria1	Mus musculus glutamate receptor, ionotropic, AMPA1 (alpha 1) (Gria1), transcript variant 1, mRNA [NM_001113325]	14799	NM_001113325	glutamate receptor, ionotropic, AMPA1 (alpha 1)	NM_001113325	Mm.4920
A_52_P481957	0.003	up	2.4273162	Grem1	Mus musculus gremlin 1 (Grem1), mRNA [NM_011824]	23892	NM_011824	gremlin 1	NM_011824	Mm.166318
A_52_P1042732	0.005	up	2.4292486	Akap5	Mus musculus A kinase (PRKA) anchor protein 5 (Akap5), mRNA [NM_001101471]	238276	NM_001101471	A kinase (PRKA) anchor protein 5	NM_001101471	Mm.311452
A_52_P622850	0.000	up	2.4325588	Hes5	Mus musculus hairy and enhancer of split 5 (Drosophila) (Hes5), mRNA [NM_010419]	15208	NM_010419	hairy and enhancer of split 5 (Drosophila)	NM_010419	Mm.137268
A_55_P1967643	0.000	up	2.434276	Plb1	Mus musculus phospholipase B1 (Plb1), transcript variant 1, mRNA [NM_001081407]	665270	NM_001081407	phospholipase B1	NM_001081407	Mm.160067
A_66_P118772	0.019	up	2.4432647	Tmem136	Mus musculus transmembrane protein 136 (Tmem136), mRNA [NM_001034863]	235300	NM_001034863	transmembrane protein 136	NM_001034863	Mm.261326

A_52_P343617	0.003	up	2.4555347	Bpifa2	Mus musculus BPI fold containing family A, member 2 (Bpifa2), mRNA [NM_008953]	19194	NM_008953	BPI fold containing family A, member 2	NM_008953	Mm.29197
A_55_P2065562	0.003	up	2.4724917	Aldh3a1	Mus musculus aldehyde dehydrogenase family 3, subfamily A1 (Aldh3a1), transcript variant 1, mRNA [NM_007436]	11670	NM_007436	aldehyde dehydrogenase family 3, subfamily A1	NM_007436	Mm.4257
A_51_P397673	0.002	up	2.4767466	Pcsk9	Mus musculus proprotein convertase subtilisin/kexin type 9 (Pcsk9), mRNA [NM_153565]	100102	NM_153565	proprotein convertase subtilisin/kexin type 9	NM_153565	Mm.133268
A_52_P343627	0.009	up	2.5899904	Rbp7	Mus musculus retinol binding protein 7, cellular (Rbp7), mRNA [NM_022020]	63954	NM_022020	retinol binding protein 7, cellular	NM_022020	Mm.46023
A_55_P2052929	0.001	up	2.5948367	Dyrk4	Mus musculus dual-specificity tyrosine-(Y)-phosphorylation regulated kinase 4 (Dyrk4), mRNA [NM_207210]	101320	NM_207210	dual-specificity tyrosine-(Y)-phosphorylation regulated kinase 4	NM_207210	Mm.44896
A_55_P2200029	0.001	up	2.6086812	Dleu2	Mus musculus adult male cortex cDNA, RIKEN full-length enriched library, clone:7730401J12 product:inferred: deleted in lymphocytic leukemia, 2, full insert sequence. [AK033078]	668253	AK033078	deleted in lymphocytic leukemia, 2		Mm.483605
A_52_P197402	0.001	up	2.6174166	Tbc1d30	Mus musculus TBC1 domain family, member 30 (Tbc1d30), mRNA [NM_029057]	74694	NM_029057	TBC1 domain family, member 30	NM_029057	Mm.233833
A_52_P265051	0.032	up	2.6536973	Sertm1	Mus musculus serine rich and transmembrane domain containing 1 (Sertm1), mRNA [NM_177854]	329641	NM_177854	serine rich and transmembrane domain containing 1	NM_177854	Mm.51187
A_51_P267933	0.009	up	2.6822252	Snhg11	Mus musculus small nucleolar RNA host gene 11 (Snhg11), mRNA [NM_175692]	319317	NM_175692	small nucleolar RNA host gene 11	NM_175692	Mm.294494
A_51_P408649	0.000	up	2.684844	Shisa2	Mus musculus shisa homolog 2 (Xenopus laevis) (Shisa2), mRNA [NM_145463]	219134	NM_145463	shisa homolog 2 (Xenopus laevis)	NM_145463	Mm.275409
A_52_P22640	0.001	up	2.720891	Slc26a8	PREDICTED: Mus musculus solute carrier family 26, member 8 (Slc26a8), mRNA [XM_003085148]	224661	XM_003085148	solute carrier family 26, member 8	XM_003085148	Mm.219530
A_51_P289131	0.003	up	2.7214544	Dmc1	Mus musculus DMC1 dosage suppressor of mck1 homolog, meiosis-specific homologous recombination (Dmc1), transcript variant 1, mRNA [NM_010059]	13404	NM_010059	DMC1 dosage suppressor of mck1 homolog, meiosis-specific homologous recombination	NM_010059	Mm.2524
A_66_P140742	0.003	up	2.7353187	Adc	Mus musculus arginine decarboxylase (Adc), mRNA [NM_172875]	242669	NM_172875	arginine decarboxylase	NM_172875	Mm.215814
A_52_P111845	0.020	up	2.7679987	Nxph2	Mus musculus neurexophilin 2 (Nxph2), mRNA [NM_008752]	18232	NM_008752	neurexophilin 2	NM_008752	Mm.131616
A_55_P1976953	0.044	up	2.7738624	Tmprss9	Mus musculus transmembrane protease, serine 9 (Tmprss9), mRNA [NM_001081688]	432478	NM_001081688	transmembrane protease, serine 9	NM_001081688	Mm.235302
A_55_P1994385	0.001	up	2.7892606	B3gnt6	Mus musculus UDP-GlcNAc:betaGal beta-1,3-N-acetylglucosaminyltransferase 6 (core 3 synthase) (B3gnt6), mRNA [NM_001081167]	272411	NM_001081167	UDP-GlcNAc:betaGal beta-1,3-N-acetylglucosaminyltransferase 6 (core 3 synthase)	NM_001081167	Mm.98333
A_55_P2144641	0.002	up	2.8521152	Sdk1	Mus musculus sidekick homolog 1 (chicken) (Sdk1), mRNA [NM_177879]	330222	NM_177879	sidekick homolog 1 (chicken)	NM_177879	Mm.151931
A_51_P301998	0.000	up	2.8717546	Fmo2	Mus musculus flavin containing monooxygenase 2 (Fmo2), mRNA [NM_018881]	55990	NM_018881	flavin containing monooxygenase 2	NM_018881	Mm.10929
A_55_P1966749	0.000	up	2.8901708	Cyp2s1	Mus musculus cytochrome P450, family 2, subfamily s, polypeptide 1 (Cyp2s1), mRNA [NM_028775]	74134	NM_028775	cytochrome P450, family 2, subfamily s, polypeptide 1	NM_028775	Mm.275188
A_55_P2147066	0.002	up	2.9176462	Dmc1	Mus musculus DMC1 dosage suppressor of mck1 homolog, meiosis-specific homologous recombination (Dmc1), transcript variant 1, mRNA [NM_010059]	13404	NM_010059	DMC1 dosage suppressor of mck1 homolog, meiosis-specific homologous recombination	NM_010059	Mm.2524
A_55_P2009817	0.032	up	2.9393775	Ssxb6	Mus musculus synovial sarcoma, X member B, breakpoint 6 (Ssxb6), mRNA [NM_001205108]	668976	NM_001205108	synovial sarcoma, X member B, breakpoint 6	NM_001205108	Mm.298030
A_55_P2012899	0.001	up	2.9438763	Vsig4	Mus musculus V-set and immunoglobulin domain containing 4 (Vsig4), mRNA [NM_177789]	278180	NM_177789	V-set and immunoglobulin domain containing 4	NM_177789	Mm.26781
A_52_P629895	0.012	up	2.9505317	Adh1	Mus musculus alcohol dehydrogenase 1 (class I) (Adh1), mRNA [NM_007409]	11522	NM_007409	alcohol dehydrogenase 1 (class I)	NM_007409	Mm.2409
A_51_P212741	0.000	up	2.9677176	Scn2b	Mus musculus sodium channel, voltage-gated, type II, beta (Scn2b), mRNA [NM_001014761]	72821	NM_001014761	sodium channel, voltage-gated, type II, beta	NM_001014761	Mm.491371
A_55_P2033110	0.002	up	2.9709585	Crisp3	Mus musculus cysteine-rich secretory protein 3 (Crisp3), mRNA [NM_009639]	11572	NM_009639	cysteine-rich secretory protein 3	NM_009639	Mm.14138
A_52_P508920	0.001	up	2.9786494	Abca6	Mus musculus ATP-binding cassette, sub-family A (ABC1), member 6 (Abca6), transcript variant 1, mRNA [NM_147218]	76184	NM_147218	ATP-binding cassette, sub-family A (ABC1), member 6	NM_147218	Mm.159704
A_55_P1989563	0.004	up	3.023255	Cd163l1	Mus musculus CD163 molecule-like 1 (Cd163l1), mRNA [NM_172909]	244233	NM_172909	CD163 molecule-like 1	NM_172909	Mm.19283
A_55_P2116310	0.007	up	3.0334435	Trim45	Mus musculus tripartite motif-containing 45 (Trim45), transcript variant 3, mRNA [NM_001165952]	229644	NM_001165952	tripartite motif-containing 45	NM_001165952	Mm.189351
A_55_P2131190	0.001	up	3.0380576	Echdc3	enoyl Coenzyme A hydratase domain containing 3 [Source:MGI Symbol;Acc:MGI:1915106] [ENSMUST00000114941]	67856		enoyl Coenzyme A hydratase domain containing 3		
A_55_P1961576	0.027	up	3.0457091	Ypel1	Mus musculus yippee-like 1 (Drosophila) (Ypel1), mRNA [NM_023249]	106369	NM_023249	yippee-like 1 (Drosophila)	NM_023249	Mm.237941
A_55_P2046563	0.008	up	3.0738263	Cym	Mus musculus chymosin (Cym), mRNA [NM_001111143]	229697	NM_001111143	chymosin	NM_001111143	Mm.297051
A_52_P87843	0.007	up	3.1173623	Aldh1a3	Mus musculus aldehyde dehydrogenase family 1, subfamily A3 (Aldh1a3), mRNA [NM_053080]	56847	NM_053080	aldehyde dehydrogenase family 1, subfamily A3	NM_053080	Mm.140988

A_55_P1983418	0.004	up	3.205319	Amy1	Mus musculus amylase 1, salivary (Amy1), transcript variant 1, mRNA [NM_007446]	11722	NM_007446	amylase 1, salivary	NM_007446	Mm.439727
A_55_P2131453	0.000	up	3.5777566	Otogl	Mus musculus otogelin-like (Otogl), mRNA [NM_001177567]	628870	NM_001177567	otogelin-like	NM_001177567	Mm.336090
A_55_P2013823	0.001	up	3.839323	Gal3st1	Mus musculus galactose-3-O-sulfotransferase 1 (Gal3st1), transcript variant 1, mRNA [NM_016922]	53897	NM_016922	galactose-3-O-sulfotransferase 1	NM_016922	Mm.103414
A_55_P2160456	0.000	up	4.3050933	Defb40	Mus musculus defensin beta 40 (Defb40), mRNA [NM_183039]	360217	NM_183039	defensin beta 40	NM_183039	Mm.271560
A_66_P100496	0.010	up	4.764042	Speer4b	Mus musculus spermatogenesis associated glutamate (E)-rich protein 4b (Speer4b), mRNA [NM_028561]	73526	NM_028561	spermatogenesis associated glutamate (E)-rich protein 4b	NM_028561	Mm.389608
A_55_P1990373	0.000	up	5.49998	Trnp1	Mus musculus TMF1-regulated nuclear protein 1 (Trnp1), mRNA [NM_001081156]	69539	NM_001081156	TMF1-regulated nuclear protein 1	NM_001081156	Mm.151594
A_52_P222696	0.000	up	6.2484856	Poteg	Mus musculus POTE ankyrin domain family, member G (Poteg), transcript variant 1, mRNA [NM_026256]	70952	NM_026256	POTE ankyrin domain family, member G	NM_026256	Mm.158160
A_52_P398925	0.000	up	6.309071	Stfa2l1	Mus musculus stefin A2 like 1 (Stfa2l1), mRNA [NM_173869]	268885	NM_173869	stefin A2 like 1	NM_173869	Mm.187847
A_66_P139618	0.000	up	7.057383	Stfa2	Mus musculus stefin A2 (Stfa2), mRNA [NM_001082545]	20862	NM_001082545	stefin A2	NM_001082545	Mm.359610
A_51_P504815	0.000	up	7.6945486	Stfa3	Mus musculus stefin A3 (Stfa3), mRNA [NM_025288]	20863	NM_025288	stefin A3	NM_025288	Mm.136573
A_55_P1963017	0.000	up	9.664193	Stfa1	Mus musculus stefin A1 (Stfa1), mRNA [NM_001082543]	20861	NM_001082543	stefin A1	NM_001082543	Mm.327618
A_51_P191909	0.032		-2.281672	Rem2	Mus musculus rad and gem related GTP binding protein 2 (Rem2), mRNA [NM_080726]	140743	NM_080726	rad and gem related GTP binding protein 2	NM_080726	Mm.274727

Supplemental Table S3: Effect of high sucrose (HS) diet on rat body, fat pads and muscle weight and glucose tolerance.

	Control (n = 15)	HS (n = 14)	P
Body weight (g)	559 ± 11	609 ± 18	0.0258
Mesenteric AT (mg/g bw)	12.3 ± 0.5	16.1 ± 0.7	< 0.0001
Epididymal AT (mg/g bw)	11.9 ± 0.4	16.5 ± 0.6	< 0.0001
Abdominal subcutaneous AT (mg/g bw)	33.9 ± 1.8	56.6 ± 2.9	< 0.0001
Gemmelic muscle (mg/g bw)	5.54 ± 0.09	4.62 ± 0.09	< 0.0001
AUC blood glucose during GTT (mg/dL/min)	156 ± 3	174 ± 4	0.0005

bw: body weight; AT: adipose tissue; AUC: area under the curve; GTT: glucose tolerance test. Data are mean ± SEM.

```
mStfa1 MSLGGVSEASRATPEIQMIANKVRPQLEAKTNKKYEKFEAVEYKTQVVAGENIFIKMDVG
hCSTA  MIPGGLSEAKPATPEIQEIVDKVKPQLEEKTNETYGKLEAVQYKTQVVAGTNYYIKVRAG
       *  **:***. ****** *.:**:**** ***:.* *:***:******** * :**: .*
   HGCFIHIKVFNGPTGKDN-YELHGYQTDKTMDEELTYF
   DNKYMHLKVFKSLPGQNEDLVLTGYQVDKNKDDELTGF
   .. ::*:***:. .*::   * ***.**. *:*** *

mStfa2 MTEYTIEIIGGLSEARPATSEIQEIADKVRPLLEEKTNEKYEKFKAIEYKVQVVQGLNYF
hCSTA        MIPGGLSEAKPATPEIQEIVDKVKPQLEEKTNETYGKLEAVQYKTQVVAGTNYY
             * ******:***.*****.***:* *******.* *::*::**.*** * **:
   IKMNVGRGCYLHINVLSGISSEND-LELTGYQTNKAKNDELTYF
   IKVRAGDNKYMHLKVFKSLPGQNEDLVLTGYQVDKNKDDELTGF
   **:..* . *:*::*:..:..:*: * *****.:* *:**** *

mStfa3 MSQENLKIKGGLSEARPATPEIQMIADKVRPLLEEQTNEKYEKFEAVEYKSQVVAGQNLF
hCSTA        MIPGGLSEAKPATPEIQEIVDKVKPQLEEKTNETYGKLEAVQYKTQVVAGTNYY
             * ******:******* *.***:* ***:***.* *:***:**:***** * :
   IKIDVGNGCFLHMKVFRGLSGEDD-LKLKGYQTNKTKTDELTSM
   IKVRAGDNKYMHLKVFKSLPGQNEDLVLTGYQVDKNKDDELTGF
   **: .*:. ::*:***:.*.*::: * *.***.:*.* ****.:
```

Supplemental Figure S4 : Sequences alignments showing homologies between hCSTA and Stefina 1-3 proteins.

CSTA is the human ortholog of the mouse Stefin (Stfa) 1-3 with 56-59 % protein identity (data are from Ensembl). These molecules have a known conserved structural fold, and present a significantly conserved amino acid segment involved in the binding of target enzymes, the cystatin motif, QXVXG (shaded area).

Supplemental Table S5: General characteristics, anthropometric and metabolic indexes of the patients studied before and after bariatric surgery (BS).

	Lean control	Obese/T2D				Obese/Non-T2D			
	n = 10	n	Presurgery	Postsurgery	P	n	Presurgery	Postsurgery	P
Weight (Kg)	60.1 ± 10.1	16	120 ± 19	90 ± 33	0.0001	18	122 ± 20	83 ± 17	0.0001
BMI (kg/m²)	22.9 ± 1.6	16	44 ± 6	33 ± 5	0.0001	18	43 ± 4	30 ± 4	0.0001
Fasting glycemia (g/L)	0.94 ± 0.02	16	1.4 ± 0.5	1.3 ± 0.5	0.0001	18	1 ± 0.1	0.8 ± 0.1	0.002
HbA1c (%)	N/A	11	8 ± 2.7	6.6 ± 2.3	0.03		N/A	N/A	
Fasting insulinemia (µU/mL)	N/A	12	23 ± 15	9 ± 4	0.002	15	26.7 ± 17.5	6.8 ± 4	0.0001
QUIKI	N/A	12	0.3 ± 0.03	0.4 ± 0.02	0.002	15	0.3 ± 0.03	0.3 ± 0.04	0.0001
HOMA-IR	N/A	12	7.8 ± 6	2.5 ± 1.2	0.002	15	6.6 ± 4.5	1.5 ± 0.9	0.0001
Total cholesterol (g/L)	2.22 ± 0.28	16	1.8 ± 0.3	1.7 ± 0.3	0.5	18	1.8 ± 0.3	1.8 ± 0.5	0.6
HDL cholesterol (g/L)	0.70 ± 0.10	14	0.4 ± 0.02	0.4 ± 0.07	0.1	15	0.4 ± 0.1	0.4 ± 0.1	0.8
LDL cholesterol (g/L)	1.32 ± 0.23	14	1.2 ± 0.2	1 ± 0.3	0.2	15	1.2 ± 0.2	1.2 ± 0.5	0.4
Triglycerides (g/L)	0.80 ± 0.22	16	1.5 ± 06	1 ± 0.4	0.006	18	1 ± 0.6	0.8 ± 0.3	0.04
GGT (UI/L)	16.9 ± 8.6	14	45 ± 66	27 ± 21	0.004	15	29 ± 19	14 ± 10	0.0001
Uric acid (µmol/L)	243 ± 74	15	347 ± 81	287 ± 79	0.02	15	344 ± 67	282 ± 80	0.005
Fibrinogen (g/L)	3.10 ± 0.71	14	3.7 ± 0.8	3.7 ± 0.8	0.3	16	3.8 ± 0.6	3.5 ± 0.7	0.1
CRP (mg/L)	N/A	15	9.0 ± 7.0	3.6 ± 1.4	0.001	14	8.3 ± 5.8	2.7 ± 2	0.002

Data are mean ± SD. BMI: body mass index; QUIKI: quantitative insulin sensitivity check index; HOMA-IR: homeostasis model assessment insulin resistance; CRP: c reactive protein; N/A: not available

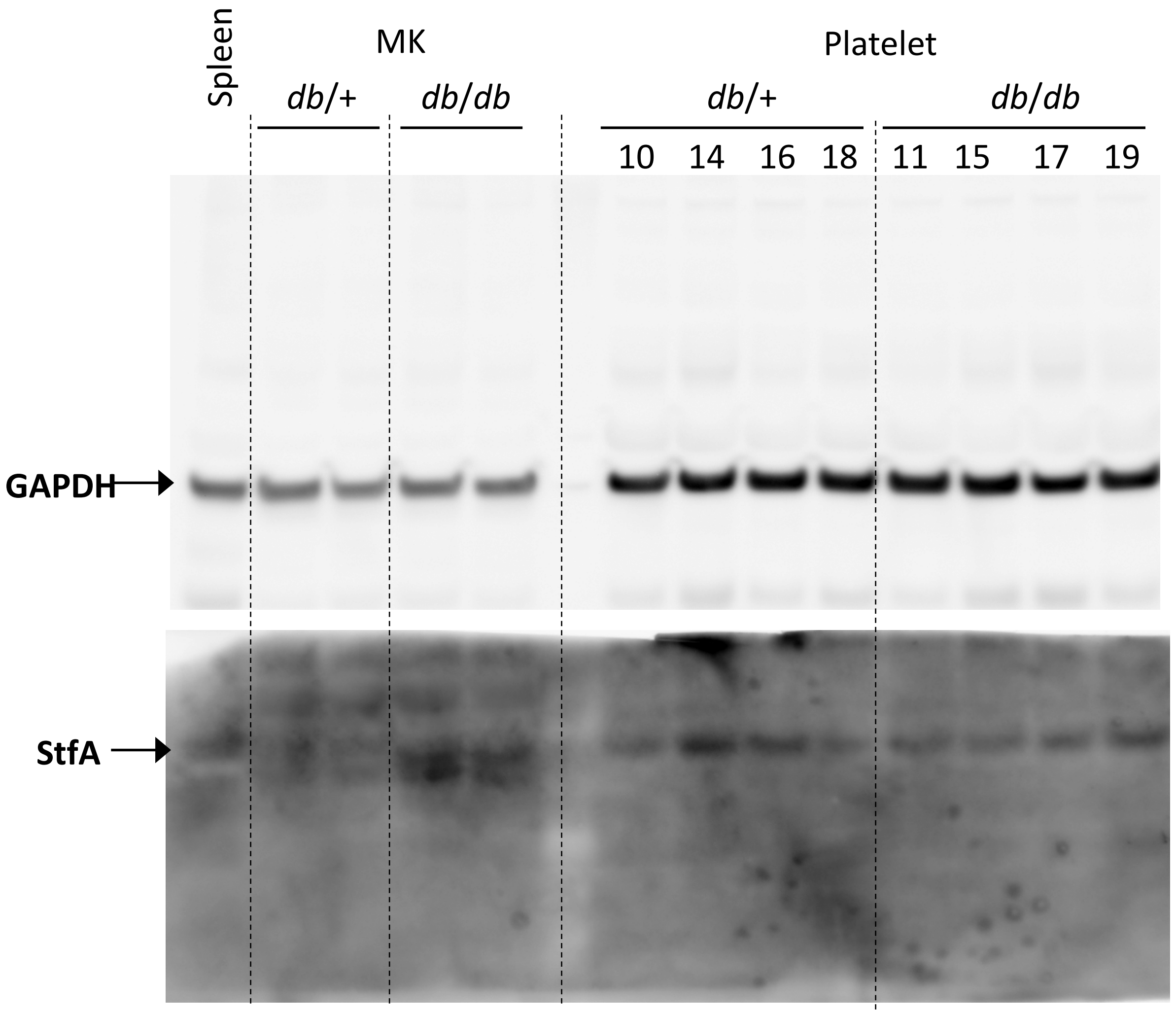

Supplementary Figure S6. Uncropped images of immunoblot presented in Figure 2b.

Representative Western blot of stfA expression of pooled (3 mice) MKs or platelets cellular lysate obtained from *db/+* or *db/db* mice (*n* = 2 and 4/group, respectively). Results were normalized to GAPDH. The illustration resulted from a single gel. Immunoblots with an exposure time allowing to visualize the position of the proteins of interest as well as the background inherent to their detections are shown. Samples are identified by the number indicated above the blot.

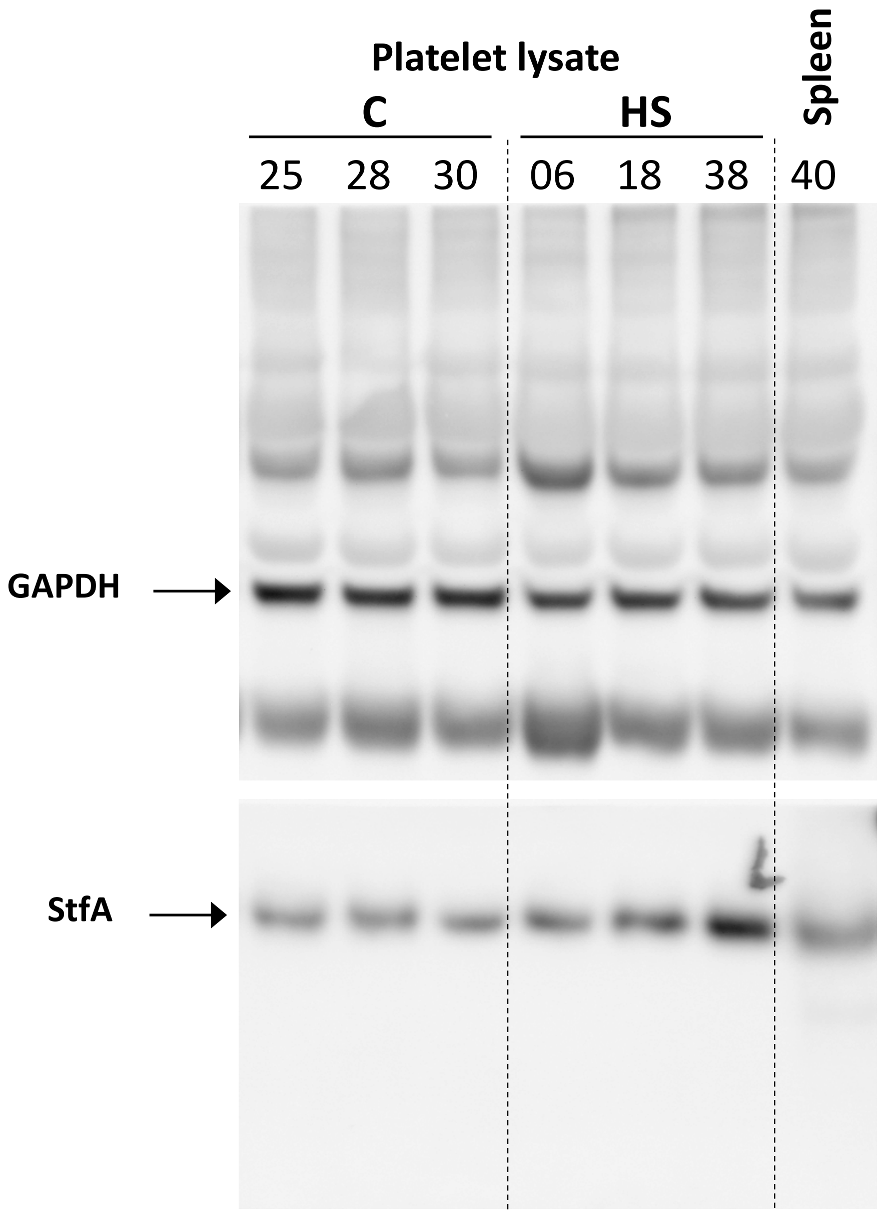

Supplementary Figure S7. Uncropped images of immunoblot presented in Figure 2c.

Representative image of StfA immunoreactivity in platelets lysates obtained from control (C, *n* = 15) or HS-fed rats (n = 14) normalized against GAPDH. Rat spleen extract was loaded as StfA positive control. Immunoblots with an exposure time allowing to visualize the position of the proteins of interest as well as the background inherent to their detections are shown. Samples are identified by the number indicated above the blot.

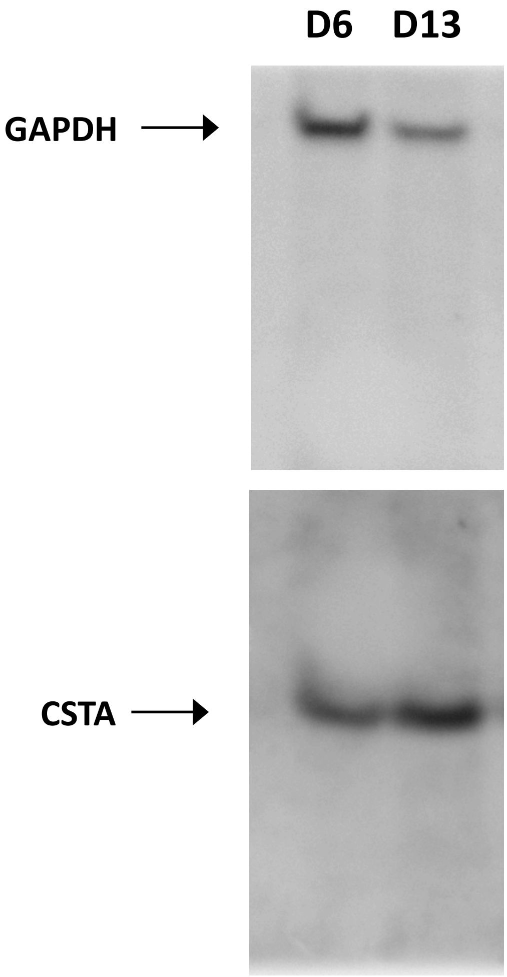

Supplementary Figure S8. Uncropped images of immunoblot presented in Figure 3b.

Time course analysis of CSTA levels during in vitro differentiation of peripheral blood CD34^{+} cells into MKs. Representative results from 2 independent experiments are showed. The immunoblot shows CSTA and GAPDH Western blot at D6 and D13.
